# Molecular and Cellular Hallmarks of Age‐Related Vestibular Hair Cell Degeneration

**DOI:** 10.1002/advs.76340

**Published:** 2026-06-26

**Authors:** Samadhi Kulasooriya, Huizhan Liu, Sarath Vijayakumar, Celia Bloom, Zhenhang Xu, Shu Tu, Benjamin J. Borgmeier, Mi Zhou, Litao Tao, Bechara Kachar, David Z. He

**Affiliations:** ^1^ Department of Biomedical Science School of Medicine Creighton University Omaha Nebraska USA; ^2^ Laboratory of Cell Structure and Dynamics National Institute of Deafness and Other Communication Disorders National Institutes of Health Bethesda Maryland USA

**Keywords:** age‐related hearing loss, aging‐associated diseases, cochlea, hair cell, mechanotransduction, neuroscience, proteostasis, senescence, transcriptome, vestibular system

## Abstract

Age‐related vestibular dysfunction (ARVD) is a prevalent and debilitating condition among the elderly, yet its etiology and underlying molecular mechanisms remain poorly understood. We focused on mechanosensitive hair cells (HCs), which are widely recognized as susceptible to aging. Using single‐cell RNA‐seq transcriptomic analysis of young and old mice, we show that old vestibular HCs exhibit conserved transcriptomic hallmarks of cellular aging, including cellular senescence, mitochondrial dysfunction, and impaired proteostasis, along with prominent cell type‐specific changes linked to hair bundle architecture and the mechanotransduction machinery. Consistent with these transcriptomic findings, imaging and electrophysiological recordings from old vestibular sensory epithelia reveal hair bundle degeneration and reduced mechanotransduction activity. Importantly, this structural and functional deterioration precedes HC loss, underscoring impaired hair bundle function as a key driver of ARVD. Furthermore, our comparative analysis identifies both shared and distinct aging signatures in vestibular and cochlear HCs, providing broader insight into the mechanisms that may underlie their different rates of age‐related degeneration.

## Graphical abstract

This study utilizes single‐cell RNA‐seq transcriptomes, advanced imaging, and electrophysiology to examine universal and cell‐type‐specific aging signatures of vestibular hair cells. The study shows that impaired hair bundle function is a key driver of age‐related vestibular dysfunction. Furthermore, the study highlights shared and distinct aging signatures between cochlear and vestibular hair cells.

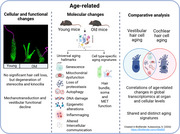

## 1 Introduction

Aging research has advanced rapidly in recent years, revealing that evolutionarily conserved genetic and molecular programs may, in part, govern the pace of aging [1]. Aging is the progressive deterioration of physiological integrity, leading to impaired function and enhanced vulnerability to disease and death. At the molecular level, aging is characterized by conserved hallmarks, including increased cellular senescence, genomic instability, epigenetic alterations, mitochondrial dysfunction, loss of proteostasis, and inflammaging [1, 2]. The interconnected and interdependent nature of these processes leads to slow structural changes and functional decline over time. Similar to other sensory organs, aging negatively affects the inner ear, leading to age‐related hearing loss (ARHL) and vestibular dysfunction (ARVD). While numerous studies have focused on ARHL, ARVD remains relatively understudied, partially due to its multifaceted etiology [3, 4, 5, 6, 7, 8]. ARVD is characterized by the gradual loss of bilateral vestibular function accompanied by interruptions in visual and proprioceptive inputs. The damaging impact of ARVD manifests itself in an exponential increase in geriatric dizziness and injurious or fatal falls [9]. According to the National Center for Health Statistics and the National Institutes of Health, the prevalence of ARVD in the US is 75.3%. It is the sixth leading cause of death and accounts for 50% of all accidental deaths among the elderly population [5, 10].

The vestibular system plays a crucial role in sensing the direction, acceleration, and spatial orientation of the head in response to gravity, thereby maintaining the upright posture and balance of the body. It consists of three cristae ampullaris that detect the angular motions and two otolith organs (utricle and saccule) that detect linear motions of the head [7]. Vestibular sensory epithelia contain two types of mechanosensitive hair cells (HCs), type I and type II. HCs convert mechanical stimuli into electrical signals. Mechanotransduction, the first key step in the vestibular signal processing, is mediated by the mechanotransduction apparatus in the hair bundle comprising tightly linked stereocilia and a kinocilium [11, 12]. Damage or degeneration of the hair bundles facilitates vestibular functional decline. While previous studies have examined physiological and morphological changes in the vestibular end organs of old animals and humans, age‐related molecular changes are poorly understood [9, 13, 14]. Although other cell types in the vestibular end organs also undergo aging, we focused on HCs as they are widely recognized for their susceptibility to aging due to accumulated damage from ototoxic drugs, noise, genetic predispositions, and comorbidities. Interestingly, studies in human and murine models indicate that the auditory sensory epithelium exhibits an earlier onset and accelerated progression of morphological and functional decline compared to the vestibular system [14]. However, the transcriptional similarities and differences associated with the aging of vestibular (vHCs) and cochlear HCs (cHCs) remain unknown. This gap in our understanding of the molecular basis of HC aging remains a significant barrier to developing targeted therapeutic interventions to mitigate ARHL and ARVD [15].

In the current study, we examined the age‐related decline of the vestibular end organs, focusing on functional, morphological, and molecular changes in HCs. Transcriptomic studies have been at the forefront of efforts to decipher the molecular mechanisms driving cellular aging in model organisms and humans. Studies from various tissues, including the mouse brain and cochlea, reveal that aging not only induces a universal program but also drives distinct transcriptional courses in different cell populations [16, 17]. Utilizing single‐cell RNA sequencing (scRNA‐seq), we examined universal and cell‐type‐specific aging signatures in vHCs from young and old mice. While vHCs exhibit shared transcriptional hallmarks of aging with other cell types, distinct cell‐type‐specific transcriptional signatures are associated with structural and functional deterioration of hair bundles and mechanotransduction apparatus. Consistent with these transcriptomic findings, imaging and electrophysiological recordings from old vestibular sensory epithelia revealed hair bundle degeneration and reduced mechanotransduction activity. Importantly, this structural and functional deterioration precedes HC loss, highlighting impaired hair bundle function as a key driver of ARVD. Moreover, comparative analysis of vHCs and cHCs reveals broad similarities and subtle differences in age‐related molecular and cellular changes. Finally, our dataset provides a foundational transcriptomic resource to advance studies of inner ear aging and support the development of targeted therapeutic strategies.

## 2 Results

### 2.1 Age‐Related Functional and Morphological Changes in Vestibular HCs

**FIGURE 1:**
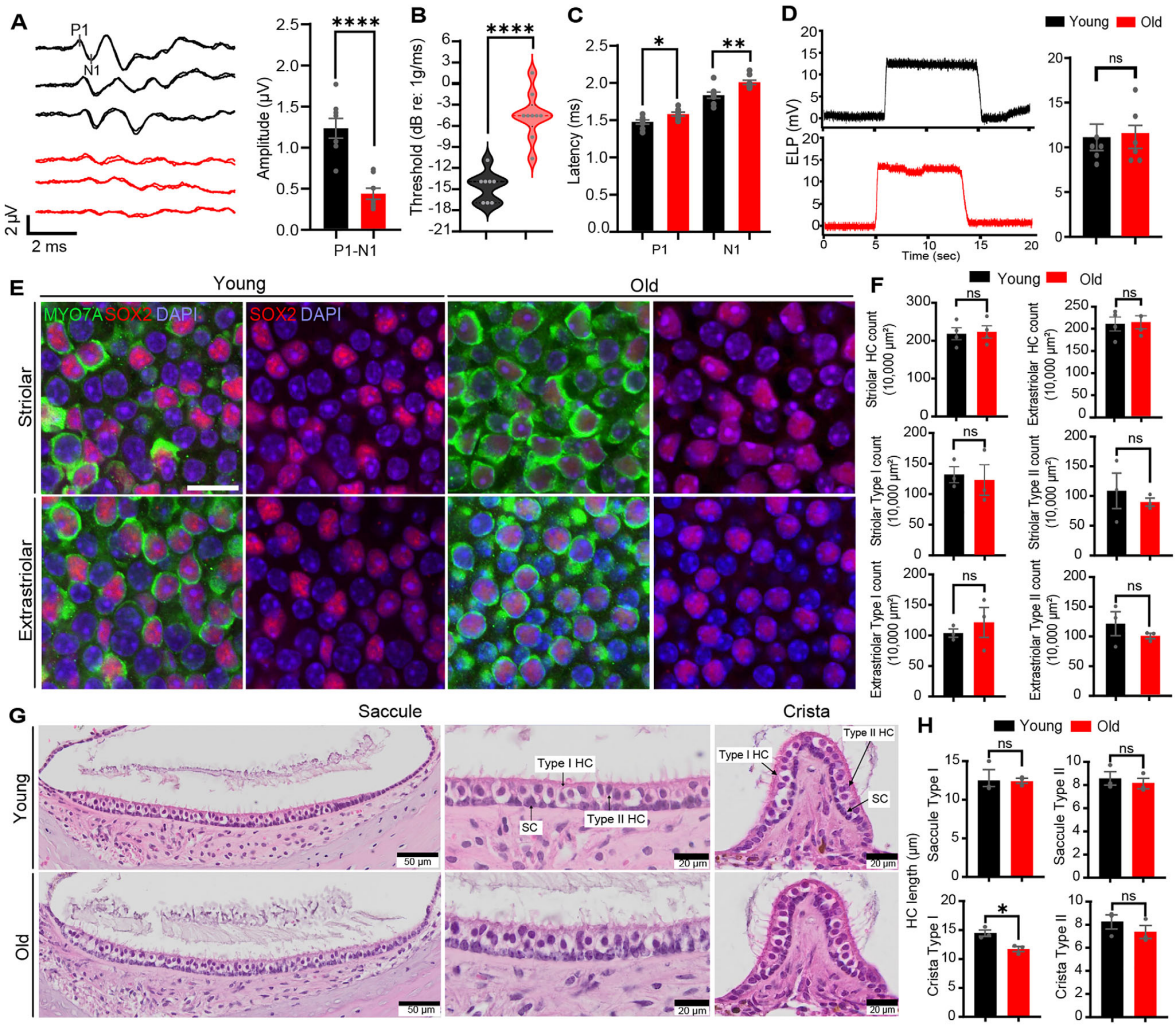
Age‐related functional and cellular changes in the vestibular system. (A–C). Measurements of VsEP from the young and old mice (*n* = 8 per age group). (A) Three representative waveforms from each age group and quantification depicting the reduction in response amplitude, (B) elevation of threshold in the old mice compared to the young, and (C) increased latency in the old mice compared to the young. P1 and N1 indicate the first positive and negative peaks. (D) Vestibular endolymphatic potential (ELP) waveforms and quantification from young and old mice (*n* = 6 per age group). (E) Representative images of young and old striolar and extrastriolar sensory epithelia (cropped region) from utricle whole mounts immunolabeled with anti‐MYO7A and anti‐SOX2, nuclei were stained with DAPI. Scale bar, 10 µm. (F) Quantifications of total HC density (*n* = 4 per age group), as well as type I and II HC density in striolar and extrastriolar regions (*n* = 3 per age group). (G) Representative images of H&E sections from young and old mice indicating type I and II HCs from the saccule and cristae (*n* = 3 per age group). (H) Quantification of type I and II HC length in young and old mice. Data are shown as mean ± SEM, individual data points represent independent biological replicates, *p < 0.05, **p < 0.01, ****p < 0.0001, ns – non‐significant by unpaired Student's *t*‐test.

Prior studies have reported various age‐related structural and functional changes in the vestibular end organs in mice and humans [5, 6, 18, 19]. In this study, we utilized CBA/J mice, which develop progressive age‐related changes in the inner ear later in life, mirroring key features of human inner ear aging [20, 21]. To examine age‐related functional decline in the vestibular system, we measured vestibular sensory evoked potentials (VsEPs) generated by the vestibular sensory epithelia in response to rapid linear head accelerations from 2‐ to 2.5‐month‐old (young) and 22‐to 24‐month‐old (old) mice. This test evaluates sensitivity (threshold), strength of the response (amplitude), and time taken by the signal to reach vestibular neurons after stimulus onset (latency) [22, 23]. Consistent with previous studies, we observed an age‐related decline in vestibular function, as evidenced by a significant decrease in response amplitude and increases in threshold and latency (Figure 1A–C). Endolymphatic potential is a driving force for mechanotransduction, and reduction in endocochlear potential is thought to be a key contributor to ARHL [24, 25]. Therefore, we measured endolymphatic potential in the utricle and found no age‐related decline in the magnitude, thus excluding reduction of endolymphatic potential as a contributor to ARVD (Figure 1D).

Next, we examined the age‐related morphological changes in old vHCs. Some studies have reported an age‐related loss of vHCs in both mice and humans [6, 26, 27], with greater loss of type I than type II HCs [6, 28], whereas other studies report no significant HC loss [29]. We examined HC status in the young and old utricles by immunolabeling with MYO7A (HC‐specific marker) and SOX2 (type II HC and supporting cell‐specific marker). Similar to previous studies [30], MYO7A‐positive/SOX2‐negative cells were identified as type I HCs, whereas MYO7A/SOX2‐positive cells were identified as type II HCs. Hair bundles were labeled with phalloidin. The otolith organs have two distinct functional zones, the striolar and extrastriolar regions, defined by reversal of hair bundle orientation. Z‐stack images were acquired from these two regions for HC quantifications. In some experiments, oncomodulin was also used to mark type I HCs in the striolar region (Figure S1) [31]. Our findings showed no significant reduction of type I and II HC density in the striolar and extrastriolar regions (Figure 1E,F and Figure S1). Changes in cell length leading to cellular hypertrophy and atrophy are commonly observed in aging cells, including cHCs and spiral ganglion neurons [17, 32]. To assess age‐related cytological changes, we measured the length of type I and II HCs in the saccule and crista ampullaris using H&E‐stained thin sections. Except for a significant cellular atrophy observed in type I HCs in crista, no significant changes in cell length were observed in other HCs (Figure 1G,H).

**FIGURE 2:**
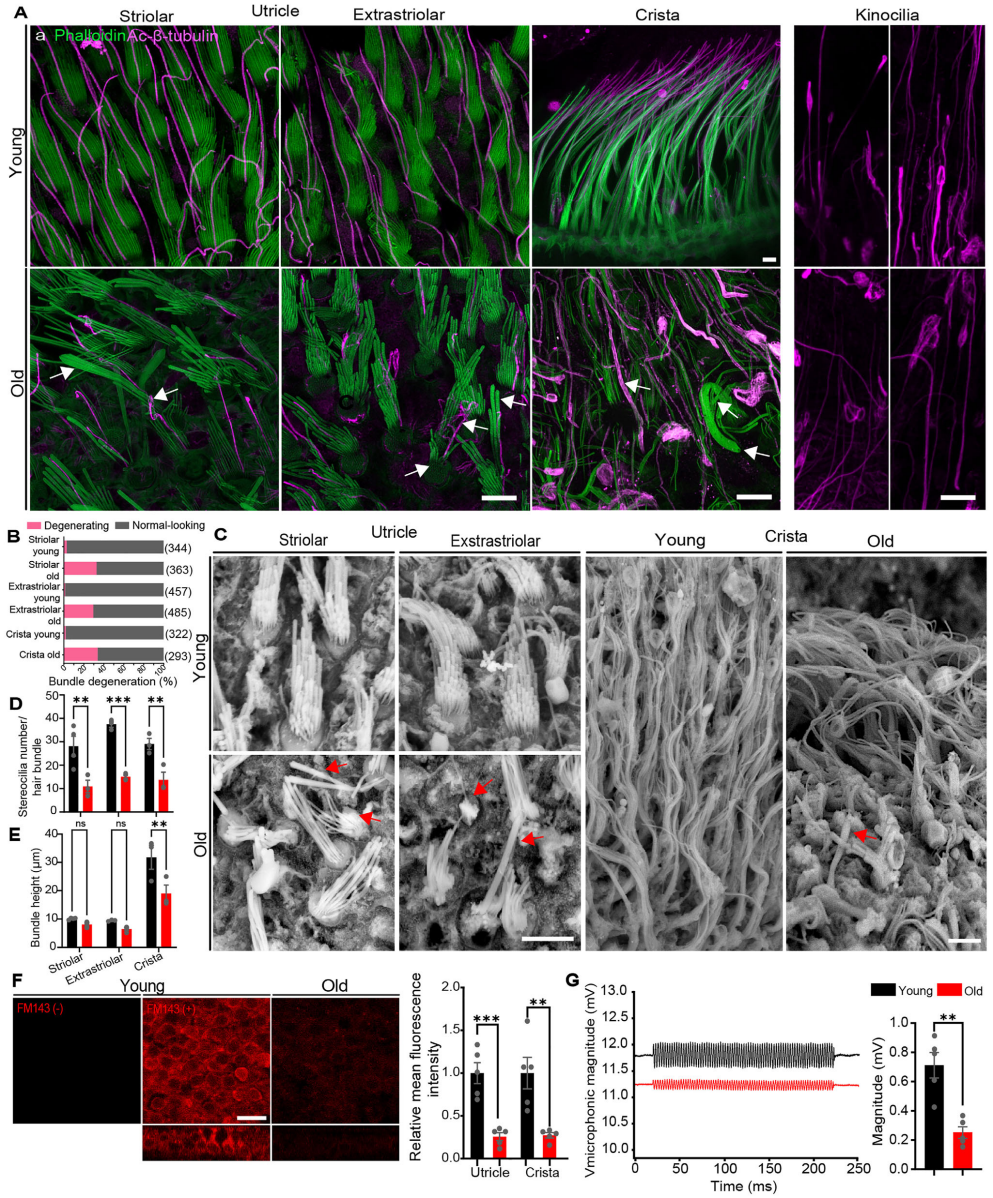
Age‐related changes in the vestibular hair bundles. (A) High‐resolution confocal microscopic images of young and old utricles and cristae, indicating stereocilia and kinocilia degeneration with aging (white arrows). Arrowheads indicate degenerative signs, such as stereocilia fusion, stereocilia loss leading to thin or short bundles, and kinocilia abnormalities, including shortening, fusion, looping, knotting, and bulb formation (left). Representative images of old kinocilia are shown in the right panel. Scale bars, 5 µm. (B) The number of bundles quantified is indicated to the right of each bar. A total of 801 HCs were counted from young utricles, 848 from old utricles, 322 from young cristae, and 293 from old cristae from *n* = 4–5 per age group. (C) Representative SEM images of hair bundles from young and old utricle and cristae, indicating signs of bundle degeneration and loss (red arrows). Scale bars, 5 µm. The representative images are taken from regions with obvious signs of bundle degeneration. (D) Quantification of stereocilia per hair bundle and (E) overall bundle height in young and old utricle and crista. Stereocilia number and bundle height were quantified from a total of 46 young and 53 old HCs from utricles, 21 young and 22 old HCs from crista, with *n* = 3–4 per age group. Data are shown as SEM, **p < 0.01, ***p < 0.001, ns – non‐significant by two‐way ANOVA Sidak's multiple comparisons test. (F) Representative images (utricle) and quantification of relative mean fluorescence intensity of FM1‐43 dye uptake in young and old HCs in the utricle and cristae (*n* = 5 per age group). Scale bar, 10 µm. (G) Representative images of vestibular microphonic measurements from the young and old macula of the utricle (*n* = 5 per age group). Data are shown as mean ± SEM, individual data points represent independent biological replicates, **p < 0.01 by unpaired Student's *t*‐test.

We utilized high‐resolution confocal imaging to examine changes in hair bundles of HCs in the striolar and extrastriolar regions of the utricle and in the crista ampullaris. Stereocilia were labeled with phalloidin, and kinocilia were immunolabeled with anti‐acetylated β‐tubulin. The most obvious signs of stereocilia degeneration observed were loss and fusion, whereas signs of kinociliary degeneration included fusion (fuses with neighboring kinocilia or stereocilia), shortening, bulb formation, aberrant looping, and knotting (Figure 2A). We observed signs of degeneration in 32.8%, 29.5%, and 33.8% of cells in the old striolar region, extrastriolar region, and crista, respectively, compared with 2.9%, 1.3%, and 1.8% in the young tissues (Figure 2B). We also utilized scanning electron microscopy (SEM) to examine hair bundle ultrastructure in the striolar and extrastriolar regions of the utricle and crista. Similar degenerative features were observed (Figure 2C). We quantified stereocilia loss and bundle length and observed a significant reduction in the number of stereocilia per hair bundle in the striolar and extrastriolar regions of both the utricle and crista. While a significant reduction in overall bundle length was observed in the crista, no significant change in bundle length was detected in the utricle (Figure 2D,E). Collectively, these findings indicate that the most striking age‐related changes in HCs are associated with hair bundles.

Next, we assessed whether these degenerative changes in old vestibular hair bundles compromise mechanotransduction. HC mechanotransduction is often assessed using FM1‐43 styryl dye uptake in vitro. This fluorescent dye rapidly enters HCs via mechano‐transduction channels. Degeneration of hair bundles and loss of mechanotransduction channel function result in reduced dye uptake by HCs [33]. We observed a significant reduction in FM1‐43 uptake by HCs in the old utricle and cristae (Figure 2F) compared to the young tissues, indicating reduced mechanotransduction with aging. Vestibular microphonic (VM) response is a direct measurement of the electrical signal produced by vHCs in response to mechanical stimuli [34]. To evaluate mechanotransduction and HC function in vivo, we recorded microphonic responses from young and old mice. A significant reduction in the magnitude of the microphonic response was observed in old mice compared to the young mice (Figure 2G), indicating that aging compromises both mechanotransduction and overall HC performance.

### 2.2 Transcriptional Profiles Associated With Universal Hallmarks of Aging in vHCs

Previous studies have identified universal and cell‐type‐specific genes and molecular processes associated with cellular aging [16]. We separately isolated the sensory epithelia from the vestibular end organs and the cochlea from young and old CBA/J mice and performed scRNA‐seq to identify these genes and molecular pathways. Sequencing reads were aligned to the mouse reference genome (mm10), followed by standard quality control and downstream processing steps, including dimensional reduction and unsupervised clustering [35]. Clusters were annotated based on expressions of known marker genes. The vestibular epithelia encompass type I and II HCs, supporting cells, and neuronal, epithelial, blood, and immune cell populations. HCs were identified by their expression of markers such as *Myo6*, *Myo7a, Pou4f3, Tmc1, and Kncn* [36, 37], and type I and II HC types were further differentiated using *Adam11* and *Sox2* [31, 38, 39]. Based on these markers, 698 type I and 129 type II HCs from young mice and 1651 type I and 230 type II HCs from old mice were identified for further analysis (Figure S2).

We aggregated single‐cell raw gene counts to generate pseudobulk gene expression profiles of young and old vHCs (Table S1). Principal component analysis was used to examine the overall age‐driven changes in the gene expression between young and old HCs across the biological replicates (Figure S3A). We observed that the most substantial differences occur between young and old groups, as evidenced by the 96% variance of principal component 1. We also assessed the number of unique and shared genes expressed in young and old type I and II HCs. We found that, irrespective of cell type or age, most genes (∼61%) are shared among HCs (Figure S3B).

**FIGURE 3:**
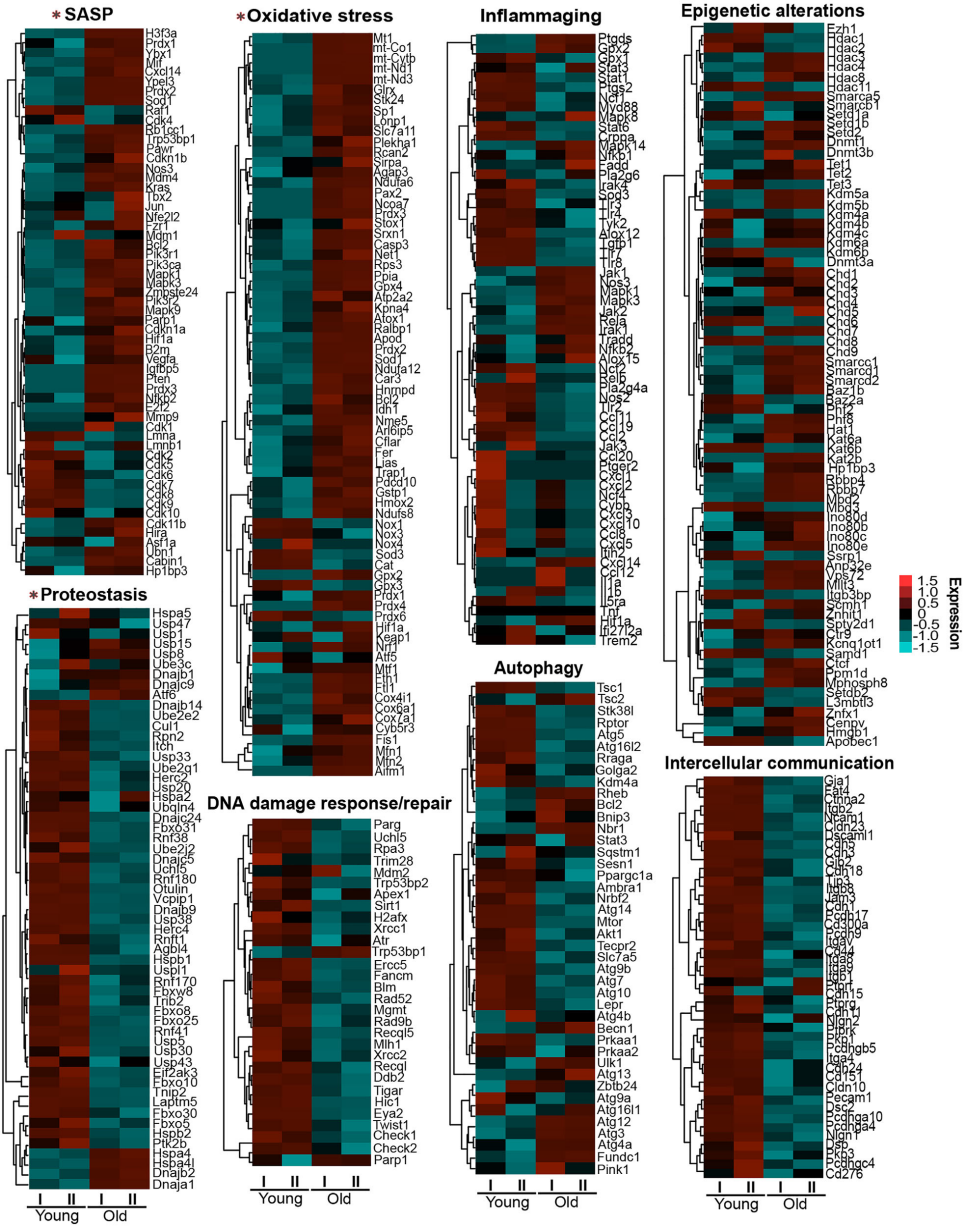
Universal hallmarks of aging in vestibular type I and II HCs. Complex heatmaps indicating the genes associated with universal hallmarks of aging, including senescence and SASP, proteostasis, oxidative stress, autophagy, inflammaging, DNA damage repair, intercellular communication, and epigenetic alterations in the old vestibular type I and II HCs compared to type I and II young HCs. Single‐cell counts were aggregated. Pseudo‐bulk gene expression values were then scaled and log2‐transformed. Expression values were centered by Cluster 3.0, and heatmaps were generated using JAVA TreeView. The color scale indicates the expression level from high (red) to low (cyan). Asterisks indicate universal pathways that were validated in Figure 4.

Cellular aging typically follows universal aging programs [1, 2, 15]. We used the pseudobulk expression matrices of young and old vHCs to examine gene expression changes associated with universal hallmarks (Figure 3). Cellular senescence is recognized as a hallmark of cellular aging. It is characterized by permanent cessation of the cell cycle, and cells adopt the characteristic senescence‐associated secretory phenotypes (SASP), which exert deleterious effects on cellular function over time [1, 2, 40]. In post‐mitotic cells, stress can induce a senescence‐like state with aging. Our data indicated a decrease in expression of cell cycle regulators (*Cdk2/4/6*), accompanied by an increase in cyclin‐dependent kinase inhibitors (*Cdkn1a/1b*), and degradation of cyclins (*Fzr1*). Moreover, we noted an increase in senescence induction (*Ypel3*), a decrease in laminins (*Lmna, Lmnb1*), and enrichment of various SASPs that are involved in inflammation (*Cxcl14, Nfkb2, B2m*), senescence‐associated heterochromatic foci (*H3f3a, Hira, Ubn1*), hypoxia, and angiogenesis (*Vegfa, Hif1a, Nos3*), indicating increased senescence in old vHCs.

The imbalance between the production and elimination of reactive oxygen species (ROS) results in oxidative stress [1, 2]. We observed an increased expression of genes involved in mitochondrial respiration, which is a major source of ROS (*Cox4i1/6a1, mt‐Co1, Ndufs2*), antioxidant response and detoxification (*Mt1, Sod1, Prdx1, Gpx4, Keap1, Nrf1*), mitochondrial fission and fusion (*Fis1, Mfn1/2*), stress response (*Bcl2, Cflar, Hif1a, Atf5*) and mitochondrial quality control (*Lonp1, Trap1*), suggesting an age‐related increase in oxidative stress followed by a compensatory increase in antioxidant mechanisms in old vHCs. In addition, key genes involved in hydrogen peroxide detoxification (*Cat*) were downregulated, suggesting impaired ROS clearance.

Age‐related increase in chronic low‐grade inflammation is termed inflammaging [1, 2]. We noticed an increase in some pro‐inflammatory genes (*Ptgds, Cxcl14, Ccl12, Il1a, Nfkb1, Jak1, Stat3*) and downregulation of other genes (*Tgfb1, Tlr3/4, Myd88, Irak4, Ccl2/5/11*), indicating potential maintenance of a prolonged low‐grade inflammatory state followed by reduced immune surveillance and response, consistent with inflammaging observed in the old utricles [41]. Epigenetic dysregulation increases with aging [42]. Old vHCs displayed a reduced expression of genes related to global heterochromatin (*Kdm5a/6a, Setdb2, Smarcb1*), nucleosome remodeling (*Smarca1, Ino80b, Vps72, Phf8, Chd4/5, Baz2a*), changes in histone marks (*Mll1/3, Setd2, Kdm4a, Ctcf, Cbx3*), global DNA hypomethylation, and CpG hypermethylation (*Dnmt1/3b, Tet1, Ezh2*). Age‐driven dysregulation of mechanisms involved in protein synthesis, folding, and turnover leads to impaired protein homeostasis, followed by the accumulation of misfolded protein aggregates. We noted decreased expression of genes involved in unfolded protein response (*Hspb1/2, Dnajb5/14, Dnajc5/24*) and protein turnover, indicating signs of impaired proteostasis in old vHCs.

Reduced DNA repair capability accumulates DNA damage, leading to genome instability with aging [1, 2, 43]. We observed a decrease in the expression of genes involved in DNA damage response and repair mechanisms (*Rad52, Xrcc1/2, Atr, Mgmt, Apex1, Recql*), indicating diminished DNA repair in old vHCs. Autophagy is crucial to recycling damaged organelles to maintain cellular homeostasis [1, 2]. We observed an enrichment of key genes related to autophagy initiation, autophagosome formation, and progression (*Becn1, Atg12/13/Atg16l1, Map1lc3b, Ulk1, Fundc1, Pink1, Sqstm1, Tsc2*). However, we also noted the downregulation of key genes (*Atg5/7/9a/9b/10/14, Prkaa1/2, Ambra1, Nrbf2, Ppargc1a*) involved in autophagosome elongation, autophagy progression, and energy sensing. This may suggest active regulation of autophagy and dysregulation in autophagosome formation and energy sensing. Aging also impacts extracellular matrix (ECM) dynamics and intercellular communication [1, 2]. We observed a reduction in genes involved in cell‐cell adhesion (*Cdh1/3/15, Pcdh9/17, Pcdhga4, Ptprk, Ptprf*), intercellular communication (*Gja1, Gjb2*), ECM interactions, and diminished tissue integrity (*Itgb1, Itga8, Tjp3, Cldn10/23*) in old vHCs.

**FIGURE 4:**
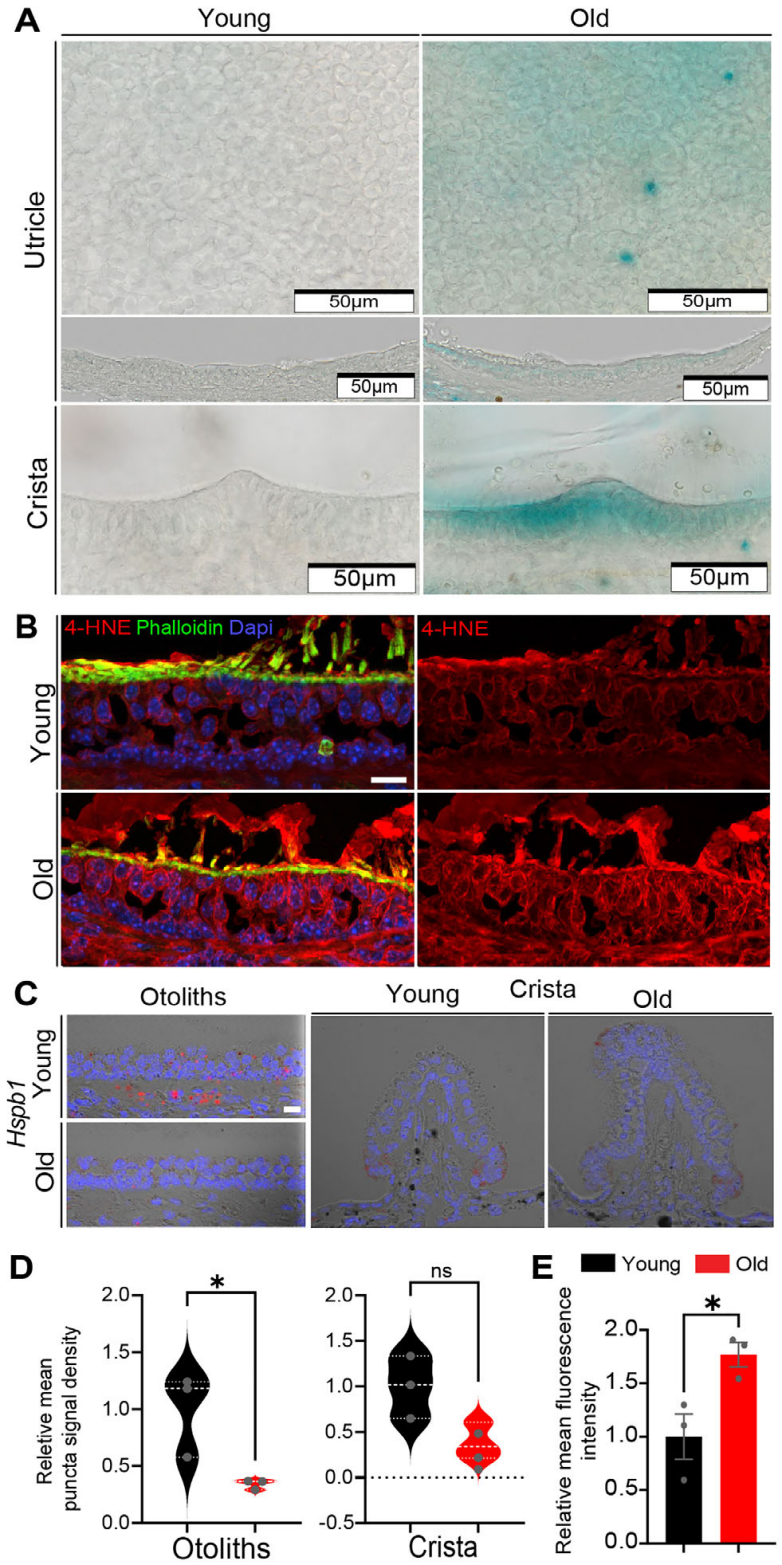
Age‐related changes in expression of markers associated with universal aging hallmarks. (A) Senescence‐associated β‐galactosidase activity (pH 6.0) in young and old vestibular HCs, shown in whole mounts and sections. Scale bar, 20 µm. (B) Immunostaining of 4‐Hydroxynonenal (4‐HNE) in young and old vestibular HCs. Scale bar, 10 µm. (C) RNAscope in situ hybridization (chromogenic red signals) of *Hspb1* (proteostasis) in young and old otolith organs and crista. Scale bar, 10 µm. (D and E) The quantifications of the relative mean puncta density of Hspb1 and the relative mean fluorescence intensity of 4‐HNE. *n* = 3 per age group, data are shown as mean ± SEM, individual data points represent independent biological replicates, *p < 0.05, ns – non‐significant by unpaired Student's *t*‐test.

To validate some of the observations from our transcriptomic data, we used immunostaining and RNAscope in situ *hy*bridization assays, as well as senescence‐associated‐β‐galactosidase (SA‐β‐gal) activity (pH 6.0) and accumulation of 4‐Hydroxynonenal (4‐HNE) to assess cellular senescence and oxidative stress [44, 45]. We observed an elevated expression of both SA‐β‐gal and 4‐HNE in old vHCs (Figure 4A,B,E). Additionally, RNAscope confirmed the age‐related downregulation of the key proteostasis marker *Hspb1* (Figure 4C,D and Figure S4), which is also implicated in ARHL. These data are consistent with the observed trends in gene expression and the universal pathways identified in our transcriptomic analysis.

### 2.3 Transcriptional Profiles Associated With Cell‐Type‐Specific Aging Signatures in vHCs

**FIGURE 5:**
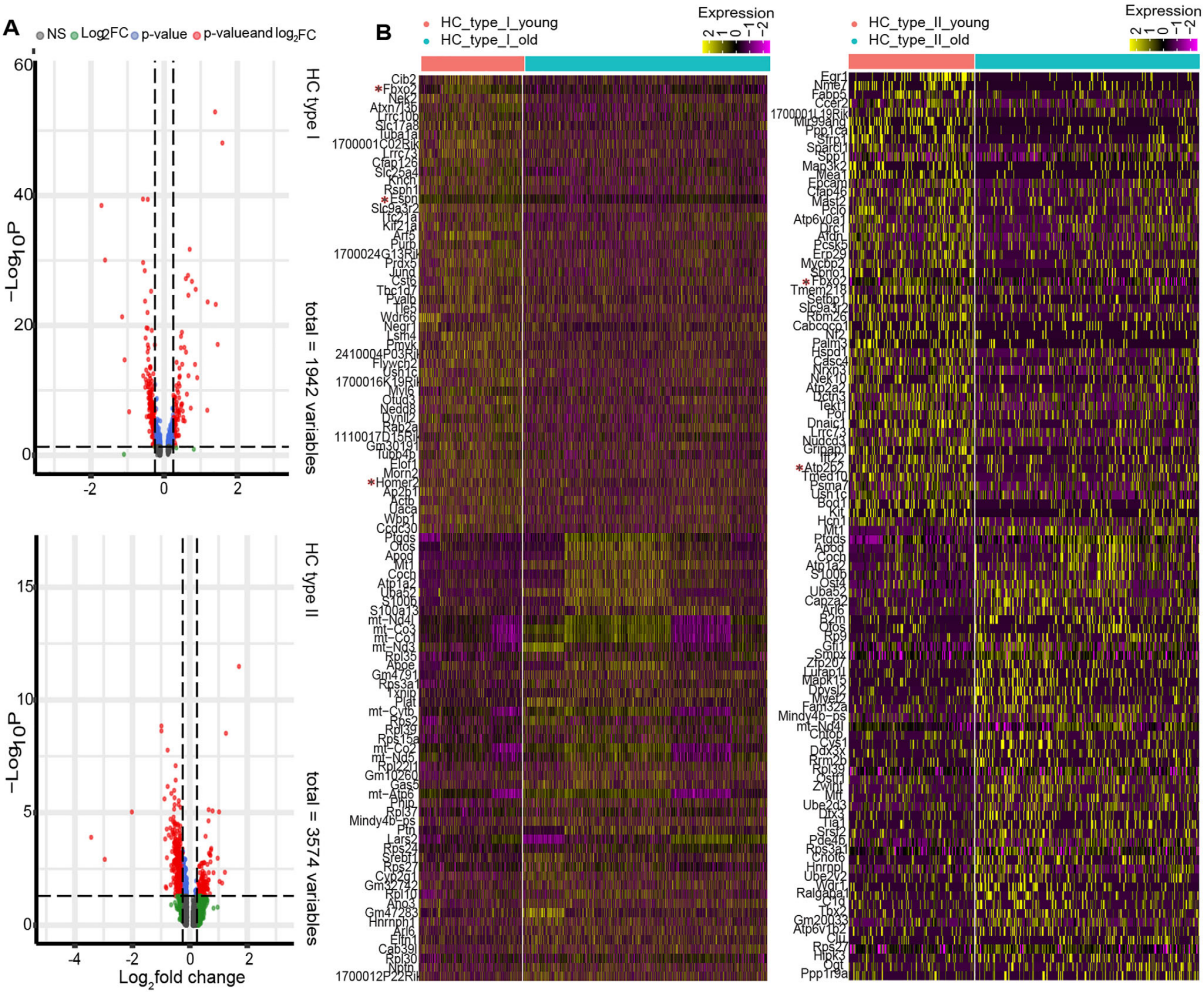
Cell type‐specific aging signatures in vestibular HCs. (A) Volcano plots of young and old type I and II HCs, *p* < 0.05, FCcutoff – 0.25, logFC threshold – 0.1. The x‐axis represents the log2 fold‐change values (avg_log2FC). The y‐axis represents the raw *p*‐values (p_val). Wilcoxon rank‐sum test with Bonferroni correction for false discovery FDR<0.01 was used to analyze DEGs. (B) Heatmaps of young and old type I and II HCs indicating the top 50 DEGs. Rows represent genes, columns represent single cells, with expression levels ranging from high (purple) to low (yellow), *p* < 0.05, FCcutoff – 0.25. Asterisks indicate genes that were validated in Figure 7.

While all cells exhibit universal hallmarks of aging, each cell type also displays distinct, cell‐type‐specific aging signatures that are often related to its unique morphology and specialized function [16, 47, 48, 49]. We examined cell‐type‐specific molecular profiles associated with aging by computing differentially expressed genes (DEGs) between young and old type I and type II HCs (Figure 5A,B). While many of our top enriched DEGs were linked to universal hallmarks, we primarily focused on DEGs associated with cell‐type‐specific aging signatures. We observed an age‐related downregulation of some key genes related to hair bundle structure and HC function, including, *Espn, Ush1c, Cib2, Kncn, Slc17a8, Tuba1a, Tubb4b, Dynll2, Kif21a, Rsph1, Cfap126, Cfap46, Drc1, Dnaic1, Dctn3, Tekt1, Ttc21a, Ift22, Nme7, Nudcd3, Ccdc30, Morn2 and Atp2b2*, many of which have been implicated in hearing loss and genetic balance disorders [50, 51, 52, 53, 54, 55].

**FIGURE 6:**
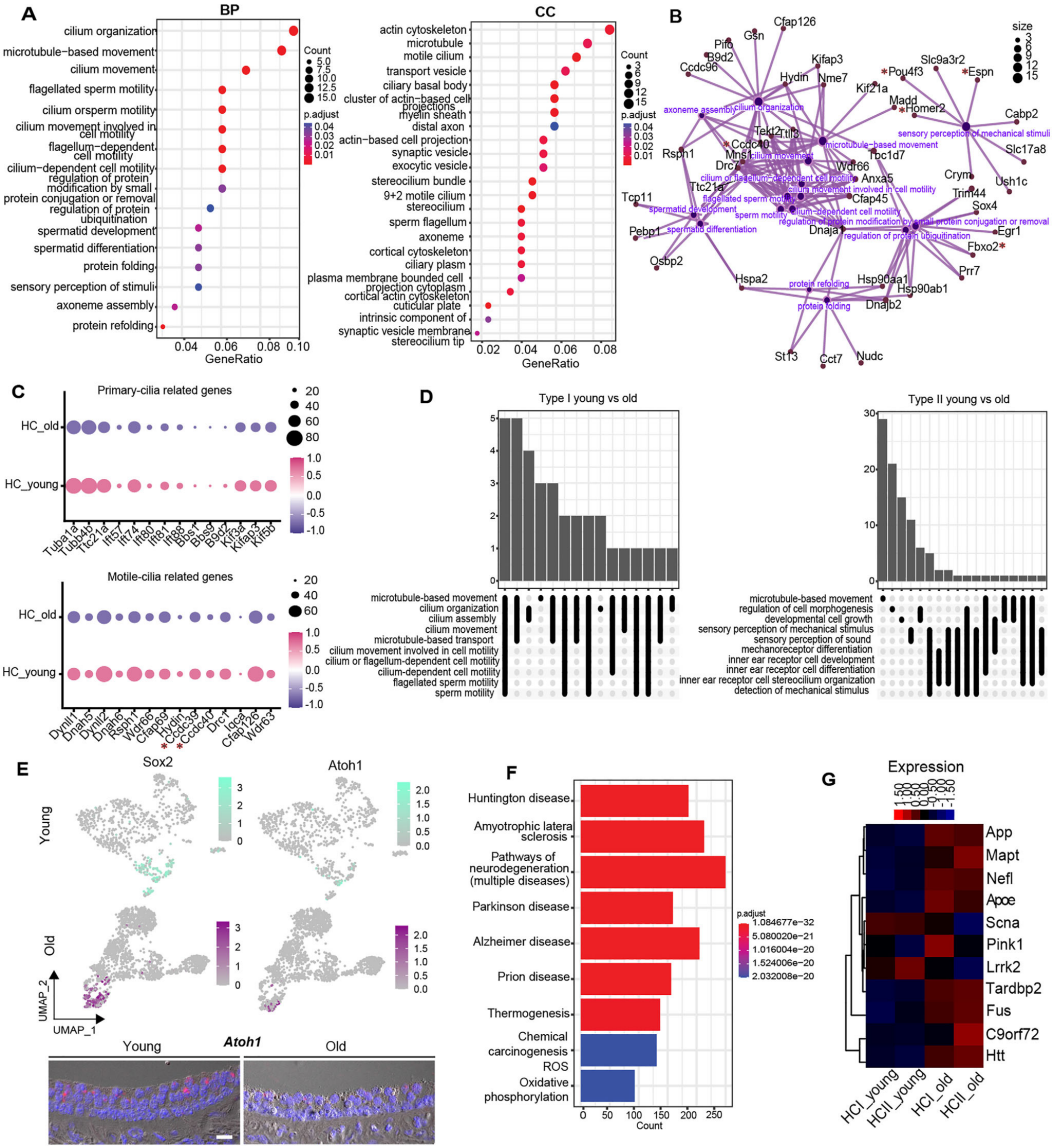
Biological processes associated with old vestibular HCs. (A) GO enrichment analysis of the biological process (BP) (left) and cellular component (CC) (right). The Hypergeometric test with the Benjamini–Hochberg adjustment was used. Pairwise semantic similarity was computed for clustering the GO terms, *p*‐value < 0.05. (B) Circular network (CNET) plot illustrates genes associated with the downregulated biological processes. (C) Dot plots indicate the primary and motile cilia‐related genes associated with kinocilia. The dot size represents the percentage of cells expressing the genes from the HC cluster, whereas the color indicates the expression level. Expression levels are normalized via z‐score normalization. Thus, the average expression is zero, and positive or negative values indicate expression above or below average. Asterisks indicate genes that were validated in Figure 7. (D) GO terms downregulated in type I young vs. old and type II young vs. old, respectively. *p*‐value < 0.05. (E) Feature plots indicating *Sox2* and *Atoh1* expression in the young and old type II HC cluster, and RNAscope in situ hybridization validation of *Atoh1* in young and old vestibular HCs. (F) KEGG pathway analysis of old vestibular HCs. The Hypergeometric test with the Benjamini–Hochberg p adjustment was used, *p*‐value < 0.05. (G) Enrichment of AD, PD, HD, ALS, and dementia susceptibility genes in vestibular HCs.

Next, we performed gene ontology (GO) enrichment and Kyoto Encyclopedia of Genes and Genomes (KEGG) analyses to examine the biological relevance of the DEGs. We observed a significant downregulation of GO terms and genes related to i) HC structure and functions, including sensory perception of stimuli, stereocilia bundle, organization, and actin cytoskeleton (*Espn, Ush1c, Pou4f3, Homer2*), ii) proteostasis‐related processes such as regulation of protein ubiquitination, proper protein folding, and refolding (*Fbxo2*), iii) synapses (Figure 6A–C). Interestingly, we observed the downregulation of genes and GO terms related to kinocilia structure and motility (Figure 6A–C). The downregulation of many kinociliary genes (Figure 6B,C), such as *Ccdc39, Ccdc40, Ccdc96, Cfap126, Hydin, Rpsh1, Ttll3, Ttc21a*, and *Cafp45, is* associated with ciliopathies [55, 56].

Previous studies have suggested that type I HCs are more vulnerable to aging than type II HCs [9]. However, we did not observe differential HC loss in our morphological assessments (Figure 1E,F). Consistent with our morphological studies, we did not observe significant differences in universal and cell‐type‐specific aging signatures between the two HC types, except for GO terms associated with mechanoreceptor differentiation and development, which were downregulated in old type II vHCs (Figure 6D). *Atoh1* is one of the genes associated with these GO terms and was downregulated in the old type II HCs (Figure 6E).

ARVD is increasingly recognized as a key player in neurodegenerative diseases and cognitive decline [57, 58]. Our KEGG pathway analysis of old vHCs revealed significant enrichment of signaling pathways linked to various neurodegenerative diseases, including Alzheimer's (AD), Parkinson's (PD), Huntington's (HD), and amyotrophic lateral sclerosis (ALS), along with an elevated expression of AD/PD (*Apoe, App, Mapt*), HD (*Htt*), ALS, and dementia (*Nefl, Fus, Tardbp, C9orf72*) susceptible genes (Figures 6F,G) [59, 60, 61, 62]. Moreover, our analyses showed downregulation of many genes and processes related to proteostasis. Age‐driven dysregulation of proteostasis and subsequent accumulation of protein aggregates lead to the onset of various diseases, including neurodegenerative diseases [63]. This suggests that neurons and vHCs exhibit some shared aging patterns.

**FIGURE 7:**
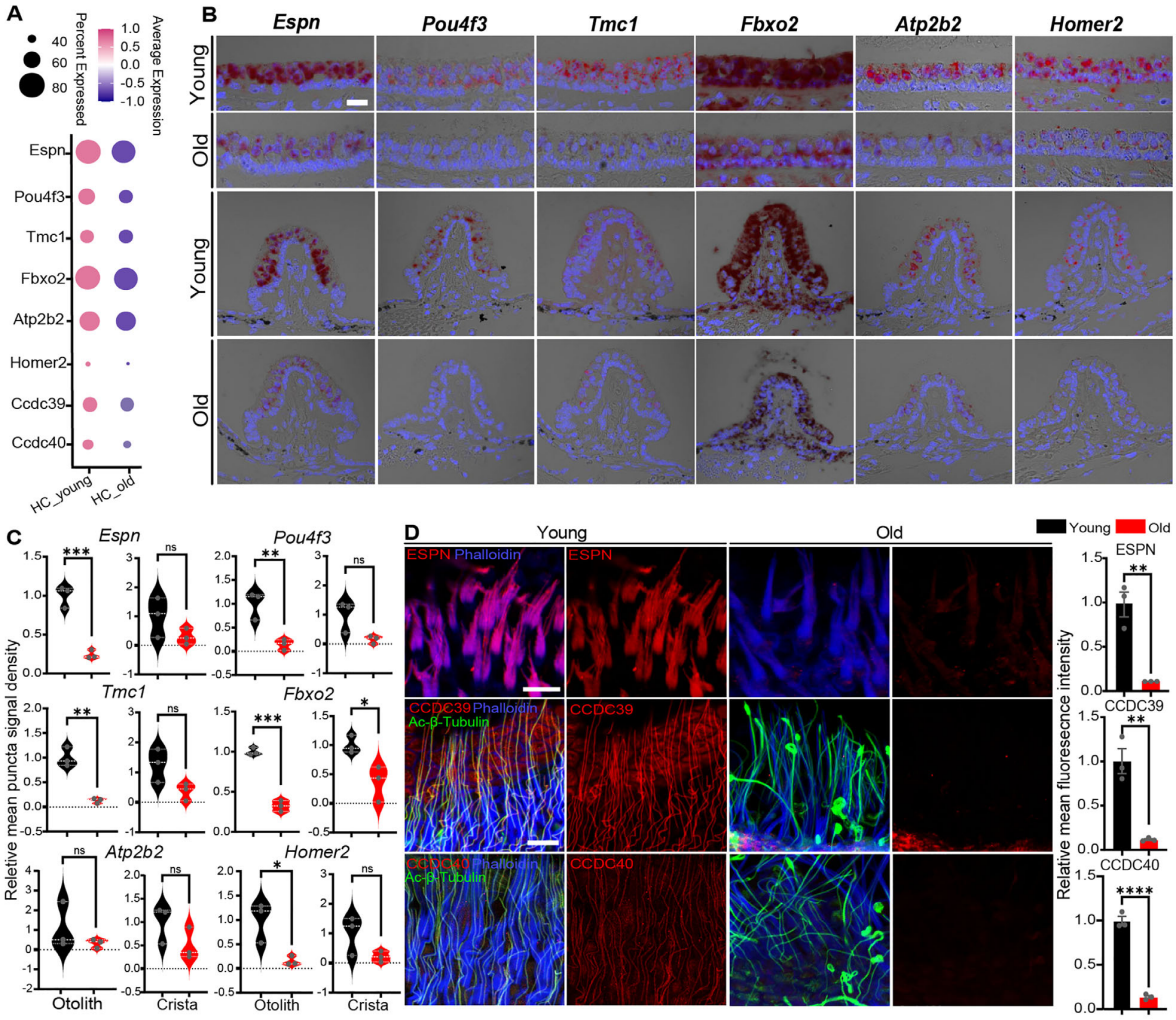
Age‐related changes in expression of cell‐type‐specific markers. (A) Dot plot shows the average gene expression levels of markers identified in DEG and GO analyses. (B) RNAscope in situ hybridization (chromogenic red signals) of key HC genes identified from DEG and GO analysis in young and old otolith organs and crista. Scale bar, 10 µm. (C) Quantifications of RNAscope in situ hybridization from otolith organs and crista (*n* = 3 per age group). (D) Representative images depicting age‐related reduction in stereocilia‐related (ESPN) and kinocilia‐related (CCDC39, CCDC40) markers (red) and quantifications of relative mean fluorescence intensity (*n* = 3 per age group). Scale bar, 10 µm. Data are shown as mean ± SEM, individual data points represent independent biological replicates, *p < 0.05, **p < 0.01, ***p < 0.001, ****p < 0.0001 by unpaired Student's *t*‐test.

To validate key markers identified from our analyses, we performed RNAscope in situ hybridization. The dot plot illustrates average gene expression of these validated markers in the young and old vHCs (Figure 7A). Representative RNAscope images of gene expressions in young and old utricles and cristae, along with quantifications, demonstrate an age‐related downregulation of these key genes (Figure 7B,C and Figure S4). Furthermore, we examined the expression of ESPN, CCDC39, and CCDC40 using immunostaining. ESPN is a multifunctional actin‐bundling protein essential for stabilizing parallel actin bundles in the stereocilia [51], while CCDC39 and CCDC40 are the molecular rulers that organize the axonemal structure in the 96‐nm repeating interactome in kinocilia and are required for ciliary motility [39]. We observed a significant age‐related downregulation of these proteins (Figure 7D), aligning with the trend observed in transcriptomic findings and the functional decline of mechanotransduction.

### 2.4 Comparison of Aging Signatures Between vHCs and cHCs

The cochlea and vestibular system exhibit a differential pace of aging [9, 29, 64]. We measured auditory brainstem response (ABR), distortion product otoacoustic emissions (DPOAE), and endocochlear potential to assess auditory functional decline in our animal cohorts in addition to VsEP (Figure S5A–C). Consistent with previous studies [14, 23], we observed a significant elevation in ABR and DPOAE thresholds in old mice. However, unlike gerbils and some mouse strains, in which endocochlear potential magnitude decreases with age [25], we did not observe a reduction in endocochlear potential magnitude in our old mice. Next, we assessed the age‐related morphological changes in the cochlea. The cochlea contains inner and outer HCs (IHCs and OHCs), which are tonotopically organized along its length. Consistent with previous studies [17], we observed significant loss of cHCs, especially OHCs in the apical and basal regions (Figure S5D), together with age‐related hypertrophy and atrophy (Figure S5E).

**FIGURE 8:**
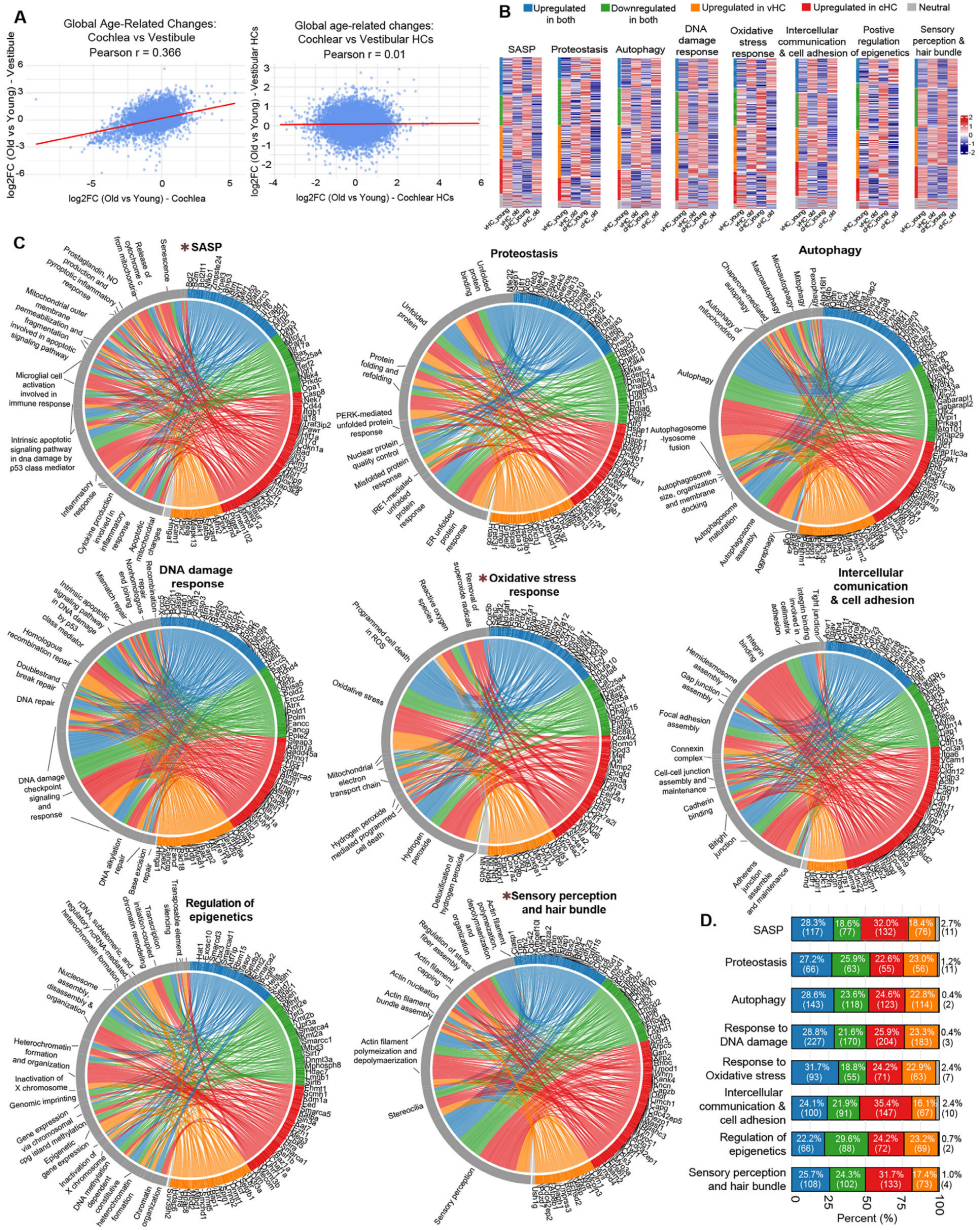
Age‐related transcriptomic changes in cochlear and vestibular HCs. (A) Scatter plots showing organ‐level linear correlation of age‐related global transcriptomic changes in the cochlea and the vestibular system (left) and global transcriptomic changes in vHCs and cHCs (right). Each dot indicates a gene plotted by log2 fold change (old vs young) in the cochlea/cHCs (x‐axis) versus vestibule/vHCs (y‐axis). The red line shows the linear regression fit. (B) Complex heatmap indicating log2‐transformed pseudobulk expression of genes associated with the indicated pathways across young and old vHCs and cHCs. Expression values were z‐score normalized per gene to highlight relative changes across groups. Color annotation reflects the shared and distinct gene sets in vHCs and cHCs. This color annotation applies to Figures B–D. Scale bar indicates expression level from high (red) to low (blue). (C) Circos chord diagrams indicating age‐related transcriptional responses of cHCs and vHCs, highlighting links between genes and Gene Ontology processes in each aging pathway. Asterisks indicate pathways that were validated in Figure S8. (D) Stacked bar plots indicating the number and percentage of genes shown in the circos chord diagrams.

Since aging signatures associated with universal and IHC‐ and OHC‐specific processes have been previously examined [17, 47], we focused on age‐related transcriptional differences between cHCs and vHCs. We identified 983 young and 149 old cHCs based on known marker genes (Figure S6) [65]. We examined the organ‐level linear correlation of age‐related global transcriptomic changes in the cochlea and the vestibular system, and observed a weak positive correlation, indicating partially shared aging programs in the two organs. Next, we assessed the cell types driving these differences and found that HCs, immune cells, neural and glial cells primarily contribute to the organ‐specific differences (Figure S7A). We further evaluated the linear correlation between cHCs and vHCs and observed no significant correlation between the two HC types (Figure 8A and Figure S7B). Finally, we compared changes in gene expression patterns and identified both shared and unique gene sets (color‐coded) associated with universal and cell‐type‐specific hallmarks in cHCs and vHCs (Figure 8B–D). The results are presented in detail in Table S2, while key biological insights are summarized below.

#### 2.4.1 Shared and Unique Genes Associated With Universal Hallmarks Between cHCs and vHCs

We observed shared upregulation of genes associated with p53‐mediated DNA damage response (*Trp53, Atm*), mitochondrial outer membrane permeabilization priming for intrinsic apoptosis (*Bok, Bbc3, Casp9*), SASP‐mediated inflammaging (*Nfkb1, B2m, Il16, Ccl5, Cxcl12*), and other core SASP regulators (*Suv39h1, Zfp277, Ypel3*), indicating persistent genotoxic stress and senescence in both cHCs and vHCs [66, 67, 68, 69]. Shared downregulated genes included checkpoint regulators (*Nek4, Prkdc*), telomere‐protective factors (*Sirt6, Terf2*), antioxidant response, and mitochondrial dynamics, indicating impaired genome stability, telomere attrition, diminished ROS detoxification, and capacity to clear dysfunctional mitochondria, along with a compensatory increase in pro‐survival genes (Sirt1, Bcl2l12, Akt3) [69, 70]. In contrast to vHCs, the major difference observed in old cHCs is the strong induction of genes related to SASP‐inflammatory mediators, inflammasome activation, immune cell activation, surveillance, and pyroptosis, consistent with cochlear inflammaging implicated in ARHL [71].

We observed a broad upregulation of genes related to ETC components (Mt‐Nd3, *Mt‐Co3*, *Cox7a1*), stress sensing, and antioxidant defenses (Sod1, Prdx1, Glrx, Gpx7, Nfe2l2). Despite this compensatory upregulation, several key genes involved in ROS detoxification (*Cat, Sod2, Gpx1, Nqo1*), ETC genes (*Ndufa8, Ndufv1, Cox5a*), and mtDNA maintenance (*Dguok*) were downregulated in both cHCs and vHCs. Declines in ETC function and mtDNA maintenance have been reported in the aging cochlea and in ARHL. These gene signatures suggest impaired ROS clearance and progressive dysregulation of mitochondrial function, priming for intrinsic apoptosis [72, 73]. We noted that cHCs were enriched for extracellular ROS detoxification genes (*Sod3, Oxr1*), stress‐responsive transcription factors (*Foxo3, Sin3a)*, hypoxia‐inducible factor *Hif1a*, and hypoxia‐adaptive ETC subunits (*Cox4i2, Cox7a2l*), indicating chronic intracellular and extracellular ROS exposure, and microvascular stress [74, 75]. In contrast, vHCs were enriched in critical players of ETC, mtDNA copy number and integrity (*Mpv17*, *Pnpt1, Ogg1*), and redox homeostasis (*Txn1, G6pdx*), suggesting enhanced efforts to maintain ETC efficiency, preserve mtDNA integrity, and increase resistance to ROS in vHCs [76, 77].

Genes related to core autophagy machinery (*Atg16l1*), autolysosome formation, lysosomal repair and biogenesis, pexophagy (*Pex2/10*), and aggrephagy (*Hspa8, Ubqln1/2*), mitophagy (*Bnip3, Prkn, Nipsnap2*) were upregulated in both cHCs and vHCs. However, we also noted the upregulation of *Zkscan3*, an energy‐sensitive master transcriptional repressor of autophagy genes, and downregulation of many genes that are involved in key steps of energy sensing (*Prkaa1*/*2*), autophagy initiation (*Atg101, Ulk2)*, membrane nucleation, phagophore formation and expansion, autophagosome formation and maturation (*Atg3, Gabarapl1/2*), and autolysosome formation. These findings suggest diminished energy sensing and dysregulation of autophagy in both HC types with aging, despite the increased effort to induce autophagy [78, 79, 80, 81]. In contrast to vHCs, cHCs show a unique upregulation of the master transcriptional activator of autophagy and lysosomal genes (*Tfeb*), and genes related to stress‐induced upstream activators of autophagy, such as hypoxia‐mediated, inflammation‐mediated, oxidative stress‐mediated, metabolic stress‐mediated, and integrated stress response‐mediated autophagy, indicating a potential stress‐adaptive hyperactivation due to chronic stress in cHCs. Moreover, we observed upregulation of genes involved in autophagic cell death (*Dapk1*) in cHCs [82].

Both cHCs and vHCs exhibit increased expression of genes associated with heat shock factors/co‐chaperones (*Dnajb12, Hspa8/b8*), ER‐mediated unfolded‐protein response (UPR) (*Eif2ak3, Atf4/6b, Xbp1*), and ER‐associated degradation (ERAD) factors (*Selenos*, *Derl2/3, Edem1*), mitochondrial proteostasis, and ubiquitin‐proteasome system. This was accompanied by downregulation of key ER/mitochondrial molecular chaperones (*Hspa5, Hspd1, Dnajb6)*, ER‐UPR (*Ern1, Hspa5, Ddit3*), and ubiquitin‐proteasome system. Although not tightly regulated, these gene signatures suggest ER stress, chronic ER‐UPR activation (ATF6‐mediated, IRE1‐mediated, PERK‐mediated), and mitochondrial‐UPR activation, indicating increased proteotoxic stress. Previous studies have reported that ER‐stress and UPR dysregulation are associated with apoptosis, necroptosis, chronic inflammation, connexin degeneration linked to sensorineural hearing loss, and ARHL [83, 84, 85]. While shared gene signatures indicate dysregulation of proteostasis in both HC types, we observed a unique upregulation of several genes related to heat‐shock chaperones (*Hsf1, Hspb1, Hsp90aa1/ab1*), co‐chaperones/aggrephagy receptors (*Dnajb1, Bag3, Cct2/3*), mitochondrial proteostasis (*Clpx*), and ER stress/apoptosis regulators (*Casp12, Daxx, Creb3l4*) in cHCs. *Bag3*, along with *Hspa/Hspb* family, is involved in chaperone‐assisted selective autophagy (CASA) complex formation, while CCTs are involved in the t‐complex polypeptide 1 ring complex (TRiC) formation, which recognizes mechanically damaged actin and tubulin for degradation [86]. Upregulation of *Daxx* and *Casp12* indicates heightened ER‐stress‐linked cell death [87]. Overall, these gene signatures indicate a high burden of unfolded/misfolded proteins in cHCs despite the increase in compensatory mechanisms, while also priming the cells for ER stress‐induced apoptosis.

We observed a broad concurrent upregulation and downregulation of genes related to DNA damage response (DDR) activation and various repair mechanisms encompassing homologous recombination, non‐homologous end joining, nucleotide‐excision repair, base‐excision repair, mismatch repair, telomere stability, and replication stress. Overall, these gene signatures suggest that both cHCs and vHCs experience heavy genotoxic stress, characterized by increased checkpoint signaling, replication fork failure, increased double‐stranded breaks, transcription‐stalling lesions, oxidative and alkylation damage, etc. These changes align well with the observed increased oxidative stress and mitochondrial dysfunction, which is known to accumulate nuclear and mitochondrial DNA damage, activating DDR, promoting HC senescence and apoptosis implicated in ARHL [45].

We observed a shared upregulation of genes related to facultative and constitutive heterochromatinization (*Suv39h1, Ehmt2*), chromatin remodelers (*Smarcad1*), polycomb/non‐polycomb repression, DNA methylation, genomic imprinting, histone variants, nucleosome assembly, and X‐chromosome inactivation. We also noted downregulation of many genes related to chromatin accessibility & nucleosome remodeling (*Smarca4, Smarce1*), chromatin marks, DNA methylation (*Dnmt3a*), polycomb repression, chromatin DDR, RNA‐mediated chromatin regulation. These gene signatures suggest H3K9 methylation, recruitment of HP1/PRC1, and heterochromatin formation, which reinforce chromatin condensation, genome stability, suppression of transposable elements, and reduced transcriptional plasticity, indicating an adaptive stress‐resistant state [42, 88]. Moreover, recent studies suggest that increased X‐chromosome inactivation with aging is associated with an increased risk of adverse diseases [89]. cHC‐specific gene signatures are reflective of additional genes related to chromatin repression and damage/repair‐associated epigenetic remodeling. In contrast, vHC‐specific gene signatures suggest tight epigenetic silencing, maintenance of methylation (*Dnmt1, Mbd1/2*), 3D genome (*Ctcf*), and nuclear architecture.

We observed upregulated and downregulated genes associated with cell–cell adhesion, tight junction, polarity, cell‐ECM adhesion, actin cytoskeleton, and intercellular signaling in cHCs and vHCs. Among the shared upregulated genes, we noted key genes (*Cldn11, Cdh23, Cdc42, Panx1*) whose loss or disruption is associated with deafness, impaired endocochlear potential, stereocilia defects, and SNHL in mice and humans [90, 91, 92]. Thus, transcriptional upregulation may be indicative of a compensatory attempt to reinforce apical junctions, epithelial barriers, and actin mechanics in response to age‐related degenerative changes. Among the shared downregulated genes, we observed key genes involved in hearing loss (*Cldn14, Cib2, Myh9*). Moreover, recent studies have demonstrated that alterations in the ECM and matrisome contribute to ARHL [93, 94, 95]. In contrast to vHCs, cHCs exhibited strong transcriptional remodeling characterized by upregulation of cadherins, claudins, tight junction, integrin‐ECM anchoring, and cytoskeletal/stereocilia/actin genes, including established deafness related genes (*Whrn, Grhl2, Actg1, Actb, Cldn9, Marveld2*), suggesting an increased effort to maintain HC integrity and barrier function in the remaining cHCs [96, 97].

#### 2.4.2 Shared and Unique Genes Associated With Cell Type‐Specific Signatures Between cHCs and vHCs 

We observed a shared upregulation of genes related to stereocilia structure and actin dynamics (*Tprn, Espnl, Ptprq, Pcdh15, Cdh23, Eps8, Capza1/2, Arpin, Cfl2*), kinocilia/ciliary‐trafficking, planar cell polarity, and maintenance of ionic homeostasis, along with a shared downregulation of core genes involved in MET machinery and actin/hair bundle (*Strc, Espn, Tmie, Triobp, Pls1, Grxcr2, Cib2, Ush1c, Loxhd1, Twf2, Fscn2, Tmod2/3, Arpc2/3*), kinociliary intraflagellar transport, planar cell polarity (*Ift27, Kif3a*), and other HC functions (*Atp2b2*, *Homer2*). Compared to vHCs, cHCs exhibited a robust upregulation of many genes involved in processes related to actin dynamics, such as actin nucleation (*Actr3/5, Wasf1, Washc3*), polymerization and depolymerization (*Pfn1, Cfl1*), capping (*Tmod1, Capz*), bundling (*Pls3, Xirp2*), contractility regulators (*Myo3a, Myo1c, Limch*), HC function‐related genes (*Otof, Kncn, Chrna9*). Interestingly, a recent study demonstrates that proteins involved in stereocilia elongation during development are required to preserve stereocilia height and organization throughout adult life, and any disruptions may impair mechanotransduction [98]. Collectively, these signatures indicate that actin remodeling is an adaptive stress response to maintain stereocilia structure via transcriptional remodeling in response to age‐related bundle degeneration. Despite these efforts, observed downregulation of core genes involved in mechanotransduction and stereocilia maintenance is consistent with morphological changes in the hair bundle and diminished mechanotransduction [17, 39, 99, 100]. We validated the age‐related downregulation of four genes (*Espn, Atp2b2, Tmc1, Homer2*) in cHCs using RNAscope and observed the same trend of downregulation of these four genes. We also assessed cellular senescence and oxidative stress in young and old cHCs. Compared to young vHCs, we noted increased SA‐β‐gal accumulation in young cHCs, as well as significantly elevated 4‐HNE accumulation in old cHCs compared to age‐matched old vHCs from the same animals, indicating a potential earlier onset and progressive age‐related changes in the cHCs (Figure S8).

## 3 Discussion

Despite its high prevalence, the molecular basis of ARVD remains poorly understood, hindering the development of targeted treatments [5, 9]. In this study, we performed comprehensive transcriptomic, functional, and morphological assessments to examine age‐related alterations in murine vestibular end organs. We identified genes and molecular processes associated with both universal and cell‐type‐specific aging signatures in HCs. Additionally, we uncovered tissue‐specific molecular differences in aging between cHCs and vHCs, providing insights into differences in age‐related processes within these two mechanosensory systems.

Aging cells, including post‐mitotic neurons, photoreceptor cells, and cHCs, exhibit universal aging hallmarks [17, 47, 101]. We demonstrated that vHCs also conform to these hallmarks. We observed heightened senescence, marked by a decrease in CDKs and an increase in CDK kinase inhibitors alongside alterations in SASP genes related to laminins, heterochromatic foci, apoptosis, hypoxia, and angiogenesis in old vHCs. We also observed signatures of increased oxidative stress and low‐grade inflammation, and impaired autophagy. We found that genes involved in DNA repair mechanisms were downregulated in old vHCs, indicating diminished DNA damage repair efficiency with age. Moreover, we saw significant epigenetic alterations, including alterations in histone marks, DNA methylation, global heterochromatin, and nucleosome remodeling in old vHCs. Genes involved in ECM, intercellular adhesion, and communication, including cadherins, integrins, and protocadherins, were downregulated in the old vHCs. These genes and pathways can be potential targets for delaying the onset and progression of ARVD. Genes and molecular processes related to universal pathways have been targeted for slowing down aging in animal models.

Our analysis indicated that the most prominent changes in genes and biological processes specific to HCs were associated with stereocilia bundles and the mechanotransduction apparatus. We also observed downregulation of axoneme‐related genes associated with vestibular kinocilia, a phenomenon not previously reported. Reduced expression of these genes is highly consistent with the hair bundle degeneration observed at the morphological level. We speculate that reduced expression of genes involved in actin cytoskeleton and intercellular communication, including cadherins and protocadherins, may also contribute to the observed degenerative changes in the hair bundles of old vHCs. Many of these downregulated genes are associated with congenital hearing loss and balance disorders [50, 51, 52, 55]. We conjecture that the vulnerability of hair bundles to aging is likely due to the wear‐and‐tear theory [102], as the hair bundles are constantly exposed to mechanical stress. Each stereocilium contains a paracrystalline actin core composed of uniformly polarized, parallel, cross‐linked actin filaments. During development, the establishment and maintenance of stereocilia length are thought to involve an actin treadmilling mechanism, with continuous polymerization at the barbed ends and depolymerization at the pointed ends [103]. In contrast, in mature hair bundles, actin turnover becomes markedly restricted and is largely confined to the distal tip region [104, 105]. This spatially limited renewal capacity likely constrains the ability of stereocilia to compensate for cumulative, lifelong mechanical wear and tear.

We noticed that the ratio of type I to type II HCs sequenced was approximately 5.4 (698 vs. 129) in young mice and 7.2 (1651 vs. 230) in old mice, substantially higher than the ratio typically observed in vivo. The lower yield ratio of type II HCs in old samples compared to young samples may suggest that type II HCs become more fragile with aging and/or enzymatic digestion. The yield of solitary cells depends on multiple factors, including cellular architecture, extracellular matrix composition, and cell‐cell junction strength. It is important to note that in the vestibular sensory epithelia, type I HCs connect directly to SCs at their apical neck through tight and adherens junctions, while calyces enwrap their basolateral body, forming a synaptic cleft [106]. In contrast, type II HCs form tight connections with neighboring type II HCs via large basolateral processes and with supporting cells [107]. This increased cellular connectivity in type II HCs may contribute to lower dissociation of solitary type II HCs compared with type I HCs. While both biological and technical factors may contribute to differences in the ratio of type I and type II HCs recovered, it is difficult to conclusively distinguish between these possibilities.

In contrast to the cochlea, where HC loss, especially OHC loss, is as high as 70%–85% in the apical and basal ends of the cochlea at 24 months [108], we observed no significant vHC loss, consistent with some previous studies [14, 29]. Moreover, we did not observe substantial age‐related changes in genes related to key Ca^2+^ and K^+^ channels expressed in vHCs. Recent reports show no significant loss of synapses [3] and vestibular neurons [29]. Thus, the vestibular functional decline at this age may not result from significant loss of HCs, synapses, and disruptions to ionic homeostasis, but rather from degeneration of the hair bundles and mechanotransduction machinery, highlighting impaired mechanotransduction as a key feature of vHC aging and a major contributor to ARVD. Interestingly, degeneration of hair bundles without substantial HC loss has been observed in previous studies following noise exposure, implicating the role of hair bundle degeneration in noise‐induced hearing loss [109, 110].

Numerous studies have identified diverse molecular changes across sensory organs during aging, reflecting inherent differences in cellular dynamics that regulate the degree and pace of aging [48, 49]. Our comparative analysis revealed shared and distinct transcriptional aging signatures between cHCs and vHCs. We observed that many genes implicated in ARHL also play a role in ARVD. Consistent with the observed profound morphological changes and cHC loss, we noted an overall pattern of heightened transcriptomic reprogramming and stress‐adaptive mechanisms in cHCs compared to vHCs. Moreover, relative to young vHCs, we noted increased SA‐β‐gal in the young cHCs, as well as significantly elevated 4‐HNE accumulation in old cHCs compared to old vHCs from the same animals, indicating a potential earlier onset and progressive age‐related changes in the cHCs (Figure S8). While our data provides insights into subtle differences in aging signatures, it remains challenging to pinpoint a single driver underlying the differential pace of aging between auditory and vestibular end organs. In humans, differences between vHCs and cHCs likely reflect a combination of intrinsic and extrinsic factors associated with differences in lifespan, sensory stimulation, cumulative wear and tear, and other environmental exposures.

Overall, the present study characterizes universal and cell‐type‐specific aging signatures in vHCs, providing insights into previously unrecognized aspects of vestibular aging. Our findings suggest that molecular deterioration may begin early, leading to diminished homeostatic capacity and promoting the accumulation of degenerative changes in vHCs that precede overt HC loss. Our data highlight hair bundle degeneration and the associated reduction in mechanotransduction as a key contributor to ARVD. The broader transcriptional landscape presented here also offers mechanistic insight into the molecular underpinnings of age‐related degeneration in vHCs and cHCs and establishes a foundation for developing targeted strategies to mitigate ARVD and ARHL.

## 4 Methods

### 4.1 Experimental Animals

Care and use of the animals in this study were approved by the Institutional Animal Care and Use Committee of Creighton University (protocol #2301045). Male and female CBA/J mice were purchased from the Jackson Laboratory (Stock #:000656) and bred and aged in the Animal Facility of Creighton University under standard conditions. The acoustic environment of the animals was examined by a sound level meter (CEL‐500, Casella Cel, UK). The sound levels were 68 dB SPL over 98% of the time, and 75 dB SPL 1%–2% of the time.

### 4.2 Vestibular Sensory Evoked Potential (VsEP)

Animal preparation and VsEP testing were performed as previously described [111]. Briefly, mice were anesthetized with a combination of Ketamine (126 mg/kg)/Xylazine (14 mg/kg) injected intraperitoneally and supplemented as needed to maintain the anesthesia. Body temperature was maintained at 37°C ± 1°C via a homeothermic heating blanket. The recording electrodes were placed anterior to the nuchal crest (non‐inverting), behind the pinna (inverting), and over the hip (ground) subcutaneously. A custom head clip was used to secure the head to the mechanical shaker (ET‐132 Labworks Inc.). The head translation was induced along the naso‐occipital axis at a rate of 17 linear pulses per second with alternating polarity and stimulus intensities ranging from +6 to −18 dB relative to 1.0 g/ms (1.0 g = 9.8 m/s^2^) adjusted in 3 dB intervals. A broad‐band forward masker (50–50 000 Hz, 92 dB SPL) was presented to avoid auditory responses. 128 responses from each of the two polarities (upward and downward head translations) were averaged to yield the mean VsEP responses. First positive (P1) and negative (N1) response peaks correspond to the compound action potential from the vestibular nerve. Thus, the amplitude and latency of these peaks were quantified. The threshold was defined as the mid‐level of the lowest stimulus intensity that elicited a detectable response and the next lowest level that did not produce a detectable response. VsEP thresholds, P1‐N1 amplitude, and latency of the young and old groups were analyzed using an unpaired Student's *t*‐test, respectively, in GraphPad Prism.

### 4.3 Auditory Brainstem Response (ABR) and Distortion Product Otoacoustic Emissions (DPOAE)

ABRs were recorded from young and old mice in a soundproof chamber as previously described [17]. The mice were anesthetized with a mixture of ketamine/xylazine, and the body temperature was maintained using a heating pad as mentioned in the VsEP procedure. Platinum recording electrodes were placed at the vertex (non‐inverting), mastoid prominence (inverting), and leg (ground) subcutaneously. Tone bursts ranging from 4 to 50 kHz were used as the stimuli, and ABRs were amplified (100, 000x), filtered, and recorded by the TDT RZ6 (Tucker‐Davis Technologies, Alachua, FL). 200 stimulus repetitions were used to yield the averaged responses. The lowest sound pressure level (dB) at which any wave (wave I–IV) was visibly detected and reproducible above the noise level was regarded as the ABR threshold. The DPOAE at 2f1‐2f2 was recorded in response to f1 and f2, with f1/f2 = 1.2, and the f2 level was set 10 dB lower than f1. The sound signal was recorded from the inner ear canal via a microphone, amplified, and the fast Fourier transforms were computed from the averaged waveforms obtained from the inner ear sound signal. The f1 sound pressure required to produce a response above the noise level at the frequency of 2f1‐f2 was considered the DPOAE threshold. ABR and DPOAE thresholds corresponding to tested frequencies in young and old groups were analyzed using a two‐way ANOVA Sidak's multiple comparisons test in GraphPad Prism.

### 4.4 Endolymphatic Potential (ELP) and Endocochlear Potential (EP)

ELP and EP measurements from the young and old mice were performed as described previously [112]. Post‐anesthesia, tracheotomy was performed in the ventral position without providing artificial respiration. Tissue and musculature overlying the tympanic bulla were removed, and the bulla was opened to access the cochlea. A fine drill was used to make small holes in the lateral wall in the apical and the basal turns of the cochlea. A glass capillary microelectrode (5–8 MΩ) filled with 150 mm KCl was mounted on the Leica micromanipulator. A stable positive DC potential was observed when the microelectrode entered the scala media. The responses were amplified (high‐pass filter at 1 kHz) using an Axopatch 200B amplifier (Molecular Probe, Sunnyvale, CA, USA) under current‐clamp mode and acquired by software pClamp 10 running on an IBM‐compatible computer with 16‐bit A/D converter (Digidata 1440A). The voltage changes during the penetration were recorded under the gap‐free mode via Clampex in the pClamp software (version 10, Molecular Devices) with a sampling frequency of 1 kHz. ELP in the utricle was recorded by inserting the microelectrode through the round window, advancing across scala media, and scala vestibuli to the utricle. The tip of the microelectrode was loaded with fluorescent phalloidin (Invitrogen #565227) diluted in 150 mM KCl (1:100) and injected into the utricle to confirm that the recording was made from the utricle. Post‐recording utricle was processed and observed under a confocal microscope. EP and ELP magnitudes of the young and old groups were compared using an unpaired Student's *t*‐test, respectively, in GraphPad Prism.

### 4.5 Recording of Vestibular Microphonic From Utricle Maculae

The vestibular microphonic was recorded as described previously [34]. Vestibular microphonic from the macula of the utricle was evoked by driving the inner ear fluid. The stimulus was a 390 Hz sinusoid with a duration of 300 ms and a rise‐time of 1 ms from a Burleigh PZ‐150 M Driver. The piezo actuator was attached to the stapes foot through a tapered glass rod (1.5 cm in length and 0.8 mm in diameter at the stapes foot) after the ossicular chain was severed. The peak‐to‐peak magnitude of the piezo motion was preset at 3 µm, sufficient to drive a saturated response. We utilized a sharp microelectrode to record the extracellular potential from the utricular maculae. A microelectrode with a tip size of ∼0.5 m should pick up local response with minimal contribution from the distant electrical activities in the organ of Corti. Moreover, the specificity of this technique for vestibular but not cochlear potentials was confirmed in a mutant mouse model with significant loss of OHCs and cochlear microphonics. The microphonic signal from the microelectrode was amplified and high‐pass filtered (at 1 kHz) under current‐clamp mode using an Axopatch 200B amplifier and acquired by software pClamp 10 running on an IBM‐compatible computer with a 16‐bit A/D converter (Digidata 1440A). The sampling frequency was 10 kHz. Responses from 20 presentations were averaged for each recording. VM magnitude of the young and old groups was compared using an unpaired Student's *t*‐test in GraphPad Prism.

### 4.6 Scanning Electron Microscopy (SEM)

Utricle and cristae from the two age groups were dissected and fixed in 2% glutaraldehyde and 4% PFA in 0.1 M sodium cacodylate buffer (pH 7.4) overnight. The fixed samples were washed with 0.1 M phosphate buffer. After removing the otolithic membrane from the utricle, the samples were treated with 1% Osmium tetroxide and washed with 0.1 M sodium cacodylate buffer. The samples were then dehydrated using a series of ethanol, followed by critical point drying using carbon dioxide via a critical point dryer (EMS 850) and mounted onto the SEM stubs. The morphology of the stereocilia bundle was visualized, and the images were acquired via Phenom Pharos desktop scanning electron microscope (Thermo Fisher Scientific). SEM images were used to quantify the number of stereocilia per hair bundle and to measure bundle height using FIJI. Only bundles with proper orientation and a clear staircase arrangement were measured, as some bundles were obscured by residual gelatinous mass of the otolithic membrane or cupula. Bundle height measurements were taken from the cuticular plate to the tallest stereocilia. We averaged the measurements taken from HCs per biological replicate. Comparison was made between young and old groups using a 2‐way ANOVA Sidak's multiple comparisons test in GraphPad Prism.

### 4.7 Cell Dissociation, cDNA Library Preparation, and Single‐Cell RNA Sequencing

Male and female CBA/J mice aged 2‐2.5 months and 22–24 months were used for scRNA‐seq. Whole cochlear and vestibular organs were microdissected from the inner ears separately in petri dishes containing cold L‐15 media (Gibco #11320033). The sensory epithelia were subjected to enzymatic digestion by transferring them into 1.5 mL Eppendorf tubes containing 1 mL/mg collagenase IV (Sigma) in L‐15 medium for 10 min at room temperature. Then the cells were resuspended in 400 µL of enzyme‐free DMEM containing 10% fetal bovine serum. Single‐cell suspensions were obtained by trituration using a trimmed 200 µL pipette tip and filtered with a 40 µm strainer. The cells were pelleted by centrifuging at 300 × g for 5 min at 4°C, then resuspended in media and subsequently quantified the number of cells. 7–8 mice were used for each biological replicate, and 4–6 biological replicates were obtained for the cochlea and vestibule for the two age groups, respectively. Single‐cell capture and library preparation were performed using droplet‐based microfluidic technology, following the manufacturer's instructions for the Chromium Next GEM Single Cell 3ʹ v3.1 dual index kit. The quality of the obtained libraries was assessed via high‐sensitivity DNA kits (Agilent Technologies) using Agilent 4200 TapeStation before sequencing with Illumina NovaSeq 6000, acquiring 50,000 reads per cell.

### 4.8 Raw Data Processing and Quality Control

Standard 10x Genomics CellRanger pipeline (version 6.1.2) was used for demultiplexing, alignment (10x mouse reference genome mm10), barcode processing, and UMI counting to obtain the filtered count matrices. Processing and visualization of the scRNA‐seq data from cochlea and vestibule were performed in R (version 4.2.3). Filtered count matrices obtained from biological replicates from cochlear and vestibular samples were loaded into R, converted to Seurat Objects, and pre‐processed to eliminate the poor‐quality cells using parameters such as counts, features, percentage of mitochondria, ribosomes, and blood gene content. Cells with at least 200 features, 1000 UMI counts, and less than 25% mitochondrial genes were included for the downstream analysis. Individual Seurat Objects were integrated into a single Seurat Object for each age group, resulting in two main Seurat Objects for young and old samples in both cochlea and vestibule, respectively, creating four Seurat Objects in total.

### 4.9 Normalization, Scaling, Integration, Dimensional Reduction, and Cluster Annotation

Integrated objects were then normalized using log‐normalization and scaled using the ‘NormalizeData’ and ‘ScaleData’ functions, followed by unsupervised principal component analysis (PCA) and dimensional reduction. The first 30 PCs were used for the dimensional reduction. The ‘FindNeighbors’ was used to calculate pairwise similarities and the ‘FindClusters’ function, which uses the Louvain algorithm, was applied with the optimal cluster resolution for all four Seurat Objects, respectively. The top markers of each cluster were identified using the ‘Findmarkers’ function. Samples were then visualized via uniform manifold approximation and projection (UMAP). This dimensional reduction method uses k‐nearest neighbors and Euclidean distance algorithms, preserving the local and global structure of the data. A total of 33 and 27 distinct clusters were identified from the initial clustering of young and old vestibular samples, while 31 and 33 clusters were identified from young and old cochlear samples, respectively. Previously reported cell‐type‐specific pan markers were used to classify the cell‐type distribution in young and old vestibular and cochlear samples. Based on the known marker genes, we identified many cell types, including vestibular type I and II HCs as well as cochlear IHCs and OHCs. Vestibular and cochlear HC clusters from the two age groups were then subsetted out and reclustered for the downstream analysis to investigate age‐related molecular changes.

### 4.10 Pseudobulk Gene Expression Analysis

Subsetted out young and old type I and II HCs were used to extract raw count data from single cells and aggregated them by averaging counts across young and old biological replicates to generate a pseudobulk data frame that contains summed expression values for the 4 HC groups (young and old type I and II). Expression values were then scaled and log2‐normalized (Table S1). Genes associated with universal aging pathways were gathered from multiple publicly available resources, such as Aging Atlas [113], and literature. Then, the general trends in the age‐related expression changes were evaluated using genes associated with each aging pathway using the ‘ComplexHeatMap’ function. Expression data were extracted and centered using Cluster 3.0 (version 1.59), and heatmaps were generated using JAVA TreeView (version 1.2.1).

### 4.11 Differentially Expressed Gene (DEG) Analysis

Principal component analysis (PCA) was performed to assess the variance among young and old vestibular datasets by using the ‘DESeq2’ package in Seurat. The default statistical test in the ‘DESeq2’ package is the Wald test with Benjamini–Hochberg correction for multiple testing. DESeq2 uses a generalized linear model (GLM) to identify DEGs within multiple groups, and variance stabilizing transformation (VST) is used to assess the variance, revealing the biological patterns in the data. DESeq2 pseudo bulk differential expression pipeline was used to generate Venn diagram via ggplot2‐based ‘ggVennDiagram’ package. The Wilcoxon rank‐sum test with Bonferroni correction in Seurat was used to assess the differentially expressed genes in young and old samples and subsequently visualized as heatmaps and volcano plots. The top 50 DEGs were identified using the ‘FindMarkers’ function, using young HCs as the reference and old HCs as the test group to generate heatmaps using the ‘DoHeatmap’ function. The genes at least expressed in 25% of cells in the cluster were considered for this analysis and ordered according to Log2 fold change (Log_2_FC). The ‘EnhancedVolcano’ function was used to generate the volcano plots using a fold‐change cut‐off (FCcutoff) value of 0.25 and a *p*‐value (pCutoff) of 0.05.

### 4.12 Functional Enrichment Analysis

The biological relevance of the top DEGs in the old samples compared to the young samples was assessed using gene ontology (GO) enrichment and over‐representation analysis. EnrichGO in the ‘clusterProfiler’ package in Seurat was used to conduct this analysis. This algorithm uses the Hypergeometric test with the Benjamini‐Hochberg method for multiple testing corrections. The top genes that were downregulated with aging (Log_2_FC<0) were used to assess the biological processes (BP) and cellular components (CC). Pairwise semantic similarity was computed for clustering the GO terms, and data were visualized as dot plots, emap plots, and cnet plots. Kyoto Encyclopedia of Genes and Genomes (KEGG) analysis was performed to assess the pathways enriched in old vestibular HCs (Log_2_FC<0). DESeq2 was used to generate a pseudobulk data matrix as described above in the DEG analysis. The ‘enrichKEGG’ function in the ‘clusterProfiler’ package was used for this analysis. This algorithm also uses the Hypergeometric test with the Benjamini‐Hochberg adjustment.

### 4.13 Comparative Pathway Analysis of Vestibular and Cochlear HCs

Young and old vestibular and cochlear HCs were subsetted based on the known marker genes, and raw gene counts were aggregated per biological replicate to generate pseudobulk matrices. Genes were aligned across conditions, variance stabilized, and normalized via DESeq2. Genes and their corresponding Gene Ontology terms related to aging pathways were obtained from the Gene Ontology Browser of the Mouse Genome Informatics. These curated pathway‐specific gene lists were used to compute Pearson correlation coefficients to assess the relationship between age‐related transcriptomic changes in cochlear and vestibular HCs. Each pathway‐specific correlation was visualized via scatter plots with overlaid linear regression lines illustrating the direction and strength of the association. Since there was no significant linear correlation, indicating differential transcriptomic response to aging, hierarchical clustering was subsequently performed to compare the age‐related gene expression patterns in cochlear and vestibular HCs. The gene‐pathway relationships across the groups were visualized via circos chord diagrams to highlight shared and unique transcriptomic changes associated with each aging pathway.

### 4.14 Histology

Temporal bones were isolated from the young and old mice, and a hole was made in the apical cochlea, followed by overnight fixation with 4% PFA (Electron Microscopy Science, Cat: 15710), followed by decalcification with EDTA (E671001‐0500; Sangon Biotech). Tissues were thoroughly washed with 1x PBS, cleared with xylene, and rehydrated with a series of ethanol. Then, tissues were embedded in paraffin wax, and sections 8 µm thick were made using a microtome. H&E staining was performed using the Fisher Scientific Gemini AS automated slide stainer. The slides were loaded onto the Gemini AS basket and immersed in xylene for 3 min (x3), followed by a series of ethanol washes (100%, 95%, and 70%), 2 min each. Next, the slides were immersed in hematoxylin for 2 min, rinsed with water, and clarifier two was added (20 s) followed by 1 min rinse with water. Blueing reagent was added for 1 min, followed by 1 min water rinse. Slides were then submerged in 70% and 95% ethanol (2 min each). Then the slides were immersed with counterstain eosin for 1 min, and the slides were then immersed in 100% ethanol (2 min x3) followed by incubation in xylene (2 min x3) and mounting. Images were taken using Olympus VS120 virtual slide scanner. Individual cell length measurements were taken along the sensory epithelia from two technical replicates per biological replicate using VS120‐ASW software. Measurements of individual cells per technical replicate were averaged, and the values from the two technical replicates were then averaged for each biological replicate. Measurements from young and old groups were analyzed using an unpaired Student's *t*‐test in GraphPad Prism.

### 4.15 Immunostaining and Confocal Imaging

Temporal bones were isolated from the young and old mice, and a hole was made in the apical cochlea, followed by overnight fixation with 4% PFA (Electron Microscopy Science, Cat: 15710) at 4°C, followed by decalcification with EDTA (E671001‐0500; Sangon Biotech). For the whole mount preparations, the vestibule and cochlea were microdissected from the temporal bones, then permeabilized in 1x PBST (0.3% Triton‐X‐100 in PBS) for 30 min, followed by blocking with 10% normal goat serum (Sera care, Cat: 5560‐0007) for 1 h at room temperature. Tissues were incubated with primary antibodies overnight at 4°C on a rocker. The samples were thoroughly washed with 1x PBST for 30 min and incubated for 40 min at room temperature with secondary antibodies and counterstains such as DAPI and Phalloidin. The tissues were subsequently washed with 1x PBST for 40 min and carefully mounted with Fluoromount mounting media. For cryosections, the tissues were fixed overnight with 4% PFA in PBS solution at 4°C and decalcified in EDTA (E671001‐0500; Sangon Biotech). Tissues were washed with PBS post‐fixing and cryoprotected in a sucrose gradient of 15% and 30%. The sections were then embedded in OCT (Fisher Healthcare, Cat: 4585) and kept on dry ice. 6 µm‐thick sections were made using Cryostat (Leica CM 1950). We used primary antibodies to immunolabel the following markers: anti‐4HNE, anti‐ESPN, anti‐CCDC39, anti‐CCDC40, anti‐MYO6, anti‐MYO7A, anti‐acetylated‐β‐Tubulin, anti‐SOX2, anti‐OCM. Goat anti‐rabbit Fluor 568, donkey anti‐mouse 568, goat anti‐rabbit Fluor 488, goat anti‐mouse Fluor 488 secondary antibodies, and Phalloidin Flour 488, Phalloidin Flour Plus 405, DAPI counterstains were used (Table S3). For cochlear and vestibular comparative experiments, samples were processed and imaged at the same time. Confocal images were taken via Zeiss LSM 700 upright microscope, Zeiss LSM 700 inverted microscope, Zeiss LSM 980 inverted confocal microscope, and Nikon‐AXR confocal microscope. All the confocal z‐stack images were processed using FIJI (2.16.0). Cell counts were obtained from 10,000 µm^2^ region of interest (ROI), and manual cell counting was performed based on the marker expression of type I (MYO7A+/SOX2‐/DAPI+) and type II HCs (MYO7A+/SOX2+/DAPI+) as described in the results. For quantification of the percentage of degenerative changes in hair bundles, confocal images from young and old utricles and cristae were used. Total HCs and the HCs with obvious degenerative signs, such as fusion or stereocilia loss, were counted and presented as a percentage in a bar graph. Individual HC bundles counted are also presented in the figure and the legend. For ESPN, mean fluorescence intensities were obtained from 10,000 µm^2^ ROIs, background corrected, and normalized to the young group to obtain the relative mean intensity. The statistical analysis was performed using an unpaired Student's t‐test in GraphPad Prism. For CCDC39 and CCDC40, intensity measurements were taken along the kinocilia and averaged per sample. The values were then normalized to the young group to obtain relative mean intensity, and the statistical analysis was performed using an unpaired Student's t‐test in GraphPad Prism. For 4‐HNE, the HC layer in sections was selected using freehand selection tools in FIJI, and fluorescence intensities were obtained, background corrected, and values were normalized to the young group. Statistical analysis was performed in GraphPad Prism using an unpaired Student's t‐test.

### 4.16 RNAscope In Situ Hybridization

Temporal bones were isolated from the young and old mice, and a hole was made in the apical cochlea, followed by overnight fixation with 4% PFA (Electron Microscopy Science, Cat: 15710), followed by decalcification with EDTA (E671001‐0500; Sangon Biotech). Tissues were thoroughly washed with 1x PBS, cleared with xylene, and rehydrated with a series of ethanol. Then, tissues were embedded in paraffin wax, and sections 6‐8 µm thick were made using a microtome. Gene expression validations were performed using RNAscope in situ hybridization following the manufacturer's instructions for RNAscope 2.5 HD Red Assay from Advanced Cell Diagnostics (ACD Bio, Cat: 322360). In brief, paraffin‐embedded tissue sections were baked in the HybEZ oven at 60°C followed by 10 min incubation with xylene and ethanol to dewax and dehydrate, respectively. Sections were then subjected to hydrogen peroxide and protease treatment before incubation with probes for 2 h at 40°C. Subsequently, amplification, detection, and counterstaining with DAPI were carried out before the samples were mounted. Images were acquired via Zeiss 700 inverted confocal microscope, and the z‐stacks obtained were presented in maximum‐intensity projection. Following this procedure, 9 probes were used to assess the gene expression, including *Espn*, *Tmc1*, *Atp2b2*, *Fbxo2, Pou4f3*, *Homer2*, *Hspb1*, *Atoh1*, and *DapB* (negative control). Confocal z‐stacks were projected using maximum intensity projection using FIJI. The HC layer was manually defined as a ROI using the freehand selection tool. RNAscope red signals were thresholded, and the total ROI area and the signal area were measured to obtain signal density, which was expressed as an area fraction (signal area divided by total ROI area). Values were then normalized to the young group, and statistical analyses were performed using unpaired Student's t‐test in GraphPad Prism for each gene.

### 4.17 FM1‐43FX (*N*‐(3‐Triethylammoniumpropyl)‐4‐(4‐(Dibutylamino) Styryl) Pyridinium Dibromide) Dye Uptake Assay

As described previously [33], the inner ears were harvested from the young and old mice and transferred to DMEM supplemented with 10% FBS, and the utricle and cristae were microdissected efficiently. The tissues were immersed in 10 µM FM1‐43 (Invitrogen, Cat: F35355) for 1 min 30 s, followed by washes with DMEM. The tissues were fixed with 4% PFA for 20 min, washed with PBS, and mounted. The images were acquired using Zeiss 980 confocal microscope. Images were processed using FIJI, and optical sections were made using the Orthoslicer tool in Imaris. Intensity measurements were taken from two 5000 µm^2^ ROIs of utricles and cristae using FIJI, respectively. Intensity values were background corrected and averaged per biological replicate and normalized to the young group to obtain the relative fold change. Young and old groups were analyzed using unpaired Student's *t*‐test in GraphPad Prism.

### 4.18 Senescence‐Associated β‐Galactosidase (SA‐β‐Gal) Staining and Imaging

According to the user manual, senescence‐associated β‐gal (SA‐β‐gal) Staining was performed using senescence‐associated β‐gal (Cell Signaling #9860) kit. The temporal bones were fixed in the 1X fixative solution provided in the kit for 30 min, as previously described [45]. Then the cochlea and the vestibular system were microdissected. According to the manufacturer's protocol, 1x staining solution, X‐Gal, and β‐galactosidase staining solutions were prepared. The cochlear and vestibular whole mounts were incubated in the staining solution overnight at 37°C. Samples were mounted and imaged using an Olympus VS120 virtual slide scanner. The whole mounts were then cryo‐sectioned and imaged utricular sensory epithelia using an Olympus VS120 virtual slide scanner.

### 4.19 Statistical Analysis

All statistical analyses and data visualizations were performed in GraphPad Prism (version 10.1.2), R Studio (version 4.2.3), Cluster 3.0 (version 1.59), and JAVA TreeView (version 1.2.1). Data are presented as mean ± standard error of the mean (SEM). Detailed data preprocessing, sample sizes (*n*), and biological replicates are provided in the figure legends or methods section. Statistical significance was calculated by two‐tailed unpaired Student's *t*‐test, or two‐way ANOVA Sidak's multiple comparisons test, where applicable. A *p*‐value of <0.05 is considered statistically significant. The statistical significance of data was denoted on graphs by asterisks (*p < 0.05, **p < 0.01, ***p < 0.001, ****p < 0.0001) or ns (not significant).

## Author Contributions

**Samadhi Kulasooriya**: conceptualization, methodology, investigation, visualization, writing – original draft, funding acquisition, writing – review and editing, formal analysis, data curation. **David Z. He**: conceptualization, supervision, funding acquisition, writing – review and editing, investigation, methodology. **Celia Bloom**: investigation, writing – review and editing. **Zhenhang Xu**: investigation. Shu Tu: investigation. **Mi Zhou**: investigation. **Benjamin J. Borgmeier**: investigation, writing – review and editing. **Huizhan Liu**: investigation, methodology. **Bechara Kachar**: supervision, writing – review and editing. **Sarath Vijayakumar**: investigation. **Litao Tao**: supervision, resources.

## Funding

This work was supported by the National Institute on Deafness and Other Communication Disorders (NIDCD), National Institutes of Health (R01 DC016807) to D.H., National Institute on Deafness and Other Communication Disorders (NIDCD), National Institutes of Health (IRP fund Z01‐DC000002) to B.K., and Dr. Richard. J. Bellucci Pre‐doctoral research award to S.K. by Bellucci De Paoli Family Foundation and Creighton University School of Medicine.

## Ethics Approval

All experimental procedures involving animals were reviewed and approved by the Institutional Animal Care and Use Committee of Creighton University (protocol # 2301045).

## Conflicts of Interest

All authors declare no conflicts of interest.

## Supporting information




**Supporting File 1**: advs76340‐sup‐0001‐SuppMat.pdf.

**Supporting File 2**: advs76340‐sup‐0002‐Table S1.xlsx.

**Supporting File 3**: advs76340‐sup‐0003‐Table S2.xlsx.

**Supporting File 4**: advs76340‐sup‐0004‐Data.zip.

## Data Availability

All relevant data are included in this paper and the supporting materials. The single‐cell RNA sequencing data generated in this study have been deposited in the NCBI Gene Expression Omnibus (GEO) under the accession numbers GSE283534 (young CBA/J) and GSE283708 (old CBA/J). Publicly available software, standard packages, and algorithms were used for the transcriptomic analysis, including Cell Ranger (RRID: SCR_017344), Seurat, and built‐in tools (RRID: SCR_016341). No custom code or algorithms were generated.
