# Supplementary material for: Molecular and Cellular Hallmarks of Age‐Related Vestibular Hair Cell Degeneration
Source: Adv Sci (Weinh). 2026 Jun 26;13(53):e76340. doi: 10.1002/advs.76340 (PMC13337082; doi:10.1002/advs.76340)
Supplement: Supplementary file 1 [file ADVS-13-e76340-s004.pdf]

Supporting information for

Molecular and Cellular Hallmarks of Age-Related Vestibular Hair Cell Degeneration

Samadhi Kulasooriya et al.
Corresponding author:
David Z. He (DavidHe@creighton.edu)

This PDF file includes:

Figures S1 to S8
Supplementary figure legends
Table S3

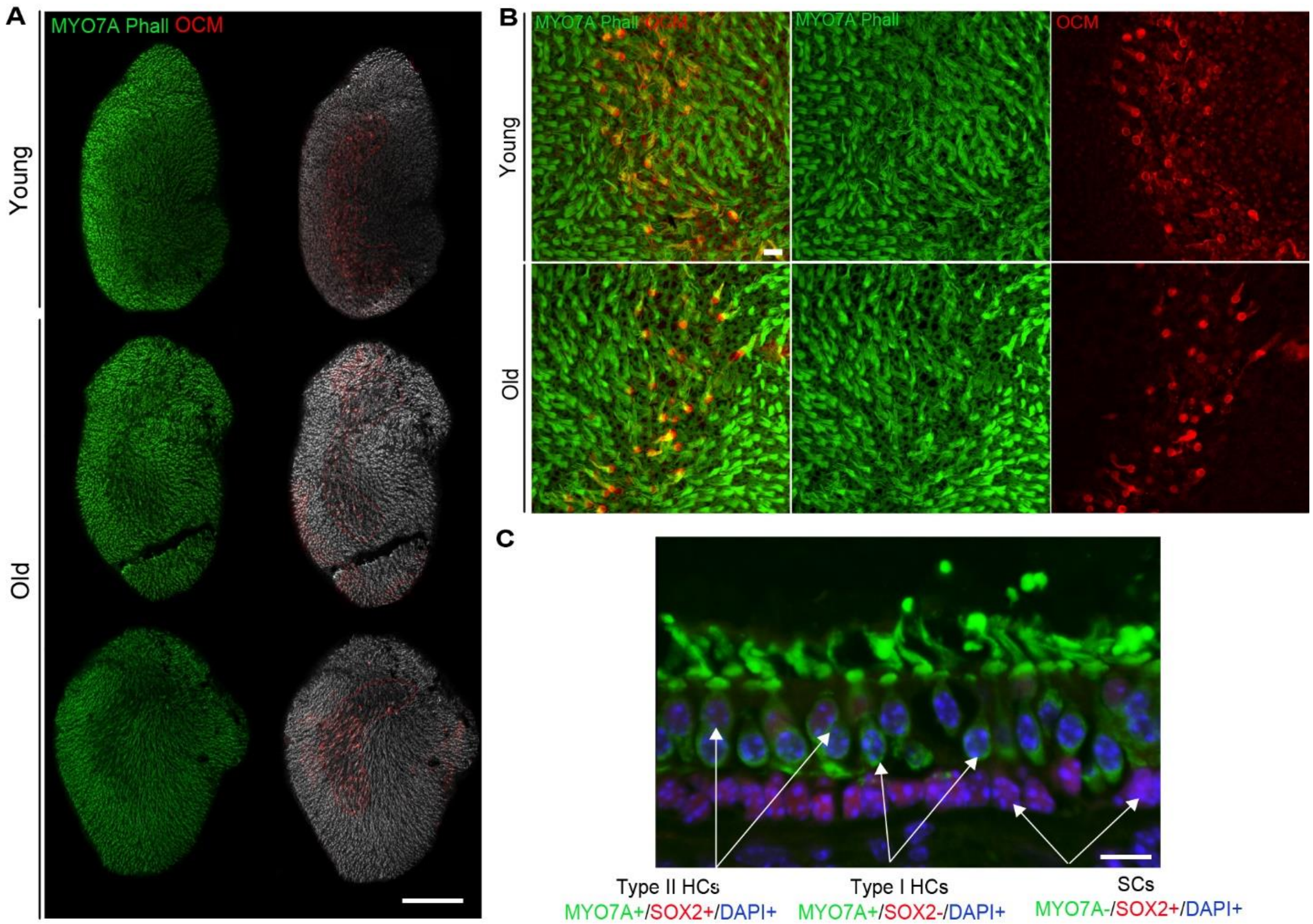

Figure S1. (A and B) Low (20x) and high (60x) magnification images of young and old utricles immunostained with anti-MYO7A, phalloidin, and anti-oncomodulin (OCM) labeling hair cells, hair bundle and striolar region, respectively. Scale bar, 100 µm and 10 µm respectively. **(C)** Orthogonal view of utricle whole mount acquired by Imaris indicating identification of type I (MYO7A+/SOX2-/DAPI+), II HCs (MYO7A+/SOX2+/DAPI+) and supporting cells (SCs) (SOX2+/DAPI+). Scale bar, 10 µm.

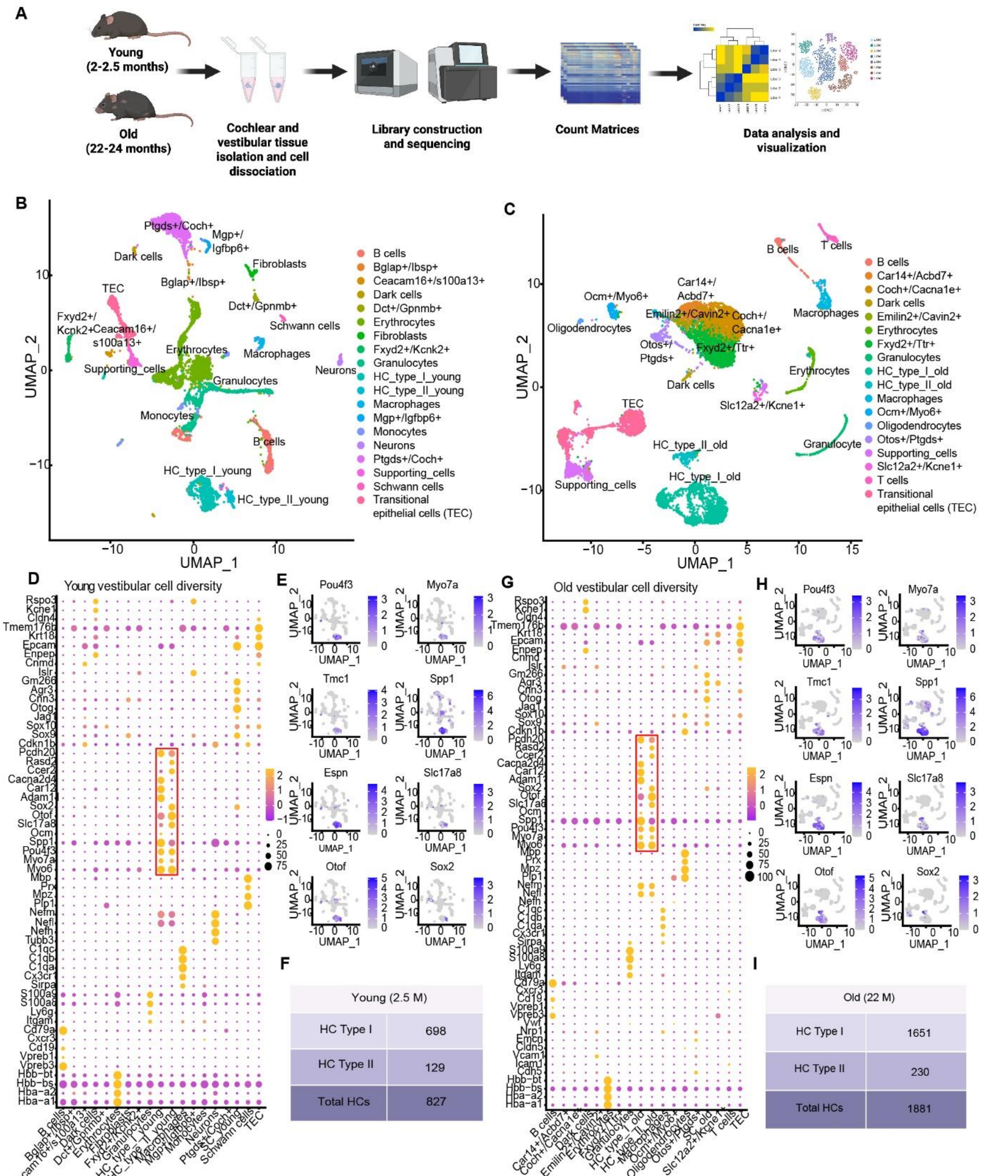

Young (2.5 M)	
HC Type I	698
HC Type II	129
Total HCs	827

Old (22 M)	
HC Type I	1651
HC Type II	230
Total HCs	1881

Figure S2: Vestibular cell type distribution and HC cluster annotation. (A) An illustration of the experimental workflow of scRNA-sequencing, data analysis, and visualization. **(B and C)** Uniform manifold approximation and projection (UMAP) plot showing the distribution of different cell types in young and old vestibular samples, respectively. This dimensional reduction method uses k-nearest neighbors (KNN) and the Euclidean distance algorithm to construct a low-

dimensional graph for visualization. **(D and G)** Dot plots indicating the expression of known marker genes and gene signatures used for cluster annotation. The dot size represents the percentage of cells expressing the genes from each cluster, whereas the color indicates the expression level. Expression levels are normalized via z-score normalization. Thus, the average expression is zero, and positive or negative values indicate expression above or below average. **(E and H)** Feature plots demonstrating the HC-specific pan marker gene expression in the HC clusters identified in the UMAPs. **(F and I)** Number of type I and II HCs identified from young and old vestibular samples for the downstream analysis.

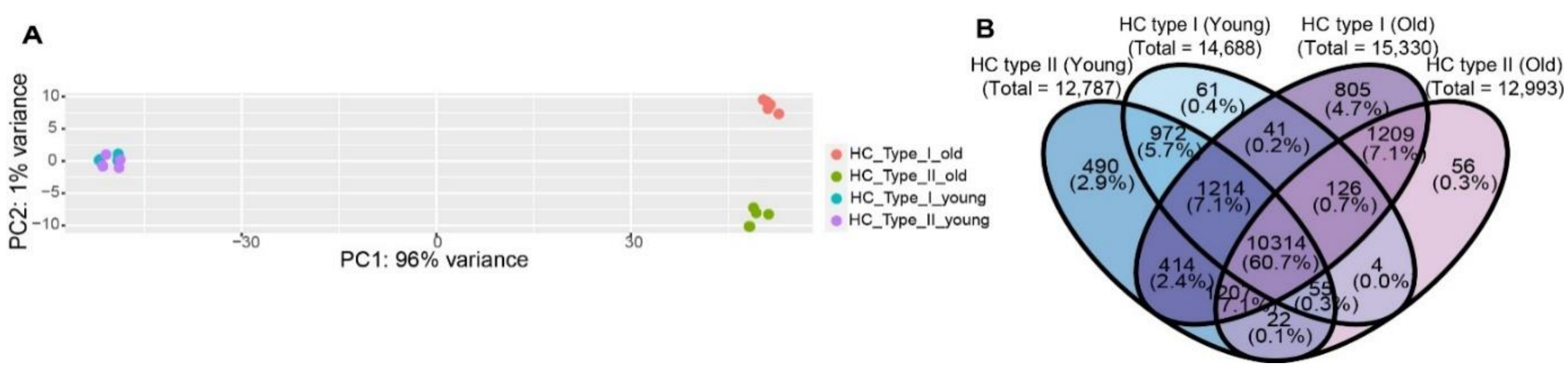

Figure S3: (A) PCA analysis of young and old type I and II HC from the biological replicates. DESeq2 in Seurat was used with the default Wald test, and multiple testing was corrected using the Benjamini-Hochberg method. Variance stabilizing transformation (VST) was applied to assess the variance among different conditions. **(B)** Venn diagram indicating the number of shared and unique genes expressed in type I and II HCs.

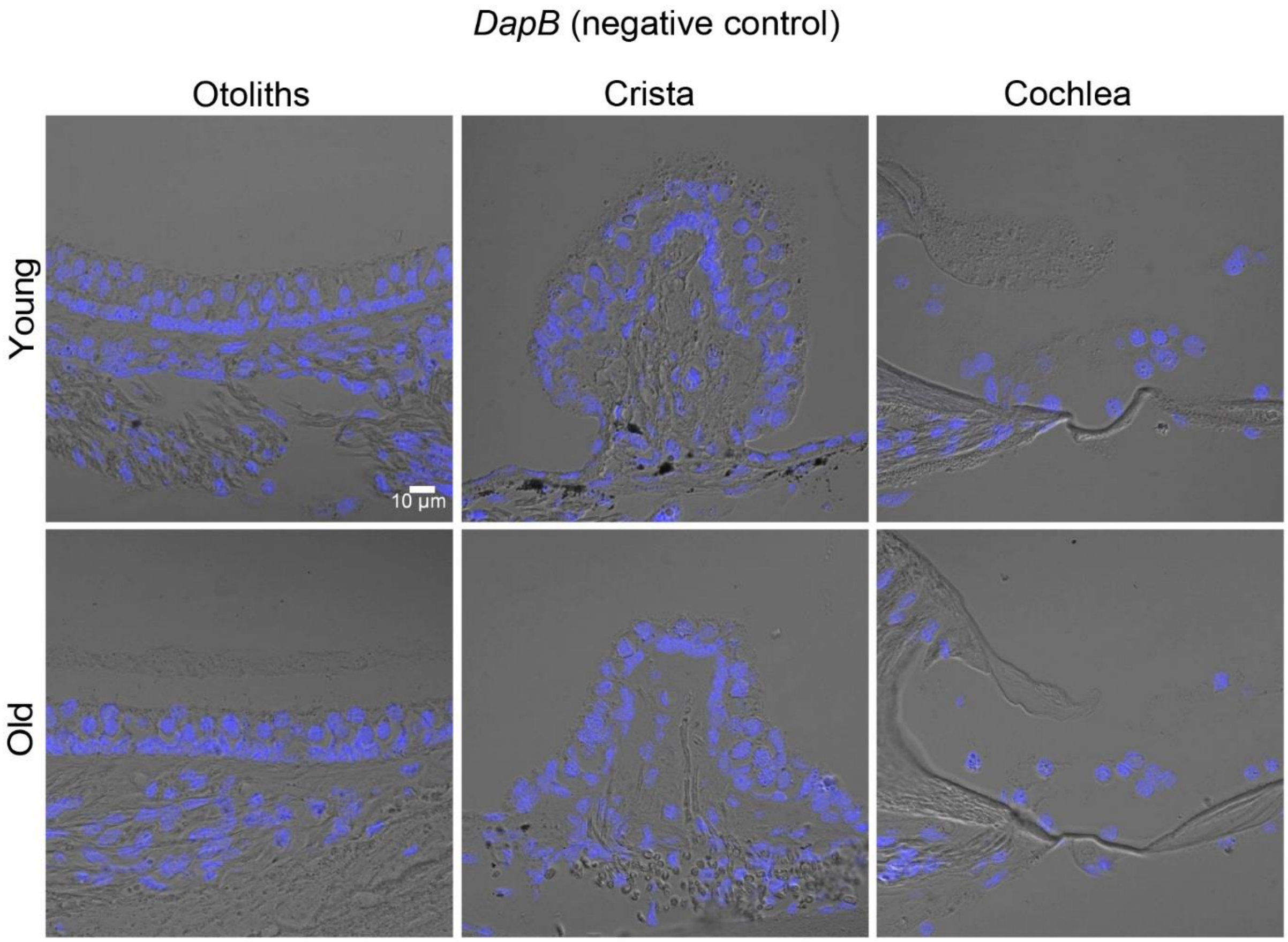

Figure S4: Representative images of *DapB* (negative control) for RNAscope *in situ* hybridization.

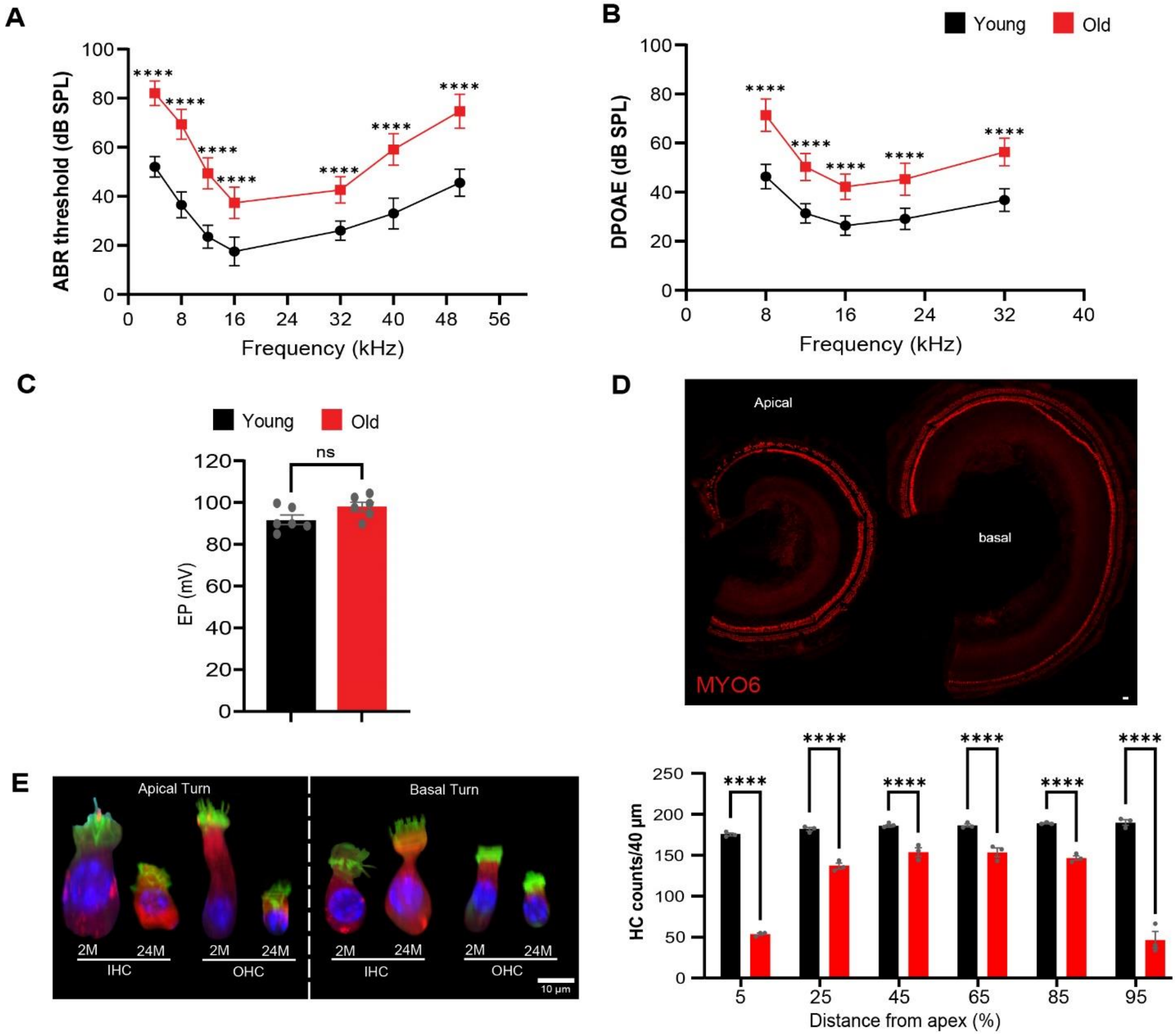

Figure S5: Age-related auditory functional and morphological changes. (**A**) Auditory brainstem response (ABR), **(B)** distortion product otoacoustic emissions (DPOAE) in young (n = 9) and old (n = 17) mice, and **(C)** endocochlear potential (EP) in young (n = 6) and old (n = 6) CBA/J. ABR and DPOAE. Data are shown as SEM, individual data points represent independent biological replicates, ****p<0.0001, ns - non-significant by unpaired t-test and two-way ANOVA Sidak's multiple comparisons test respectively. **(D)** Representative images of the cochlea immunolabeled with anti-MYO6 and the HC quantification along the tonotopic organization. Scale bar 20 µm. Data are shown as SEM, individual data points represent independent biological replicates, ****p<0.0001, ns - non-significant by two-way ANOVA Sidak's multiple comparisons respectively. **(E)** Orthogonal images of individual HCs demonstrating cellular hypertrophy and atrophy in IHC and OHCs from apical and basal turns with aging. Orthogonal images were acquired using the orthoslicer tool in Imaris software, and the background was removed using Adobe Photoshop.

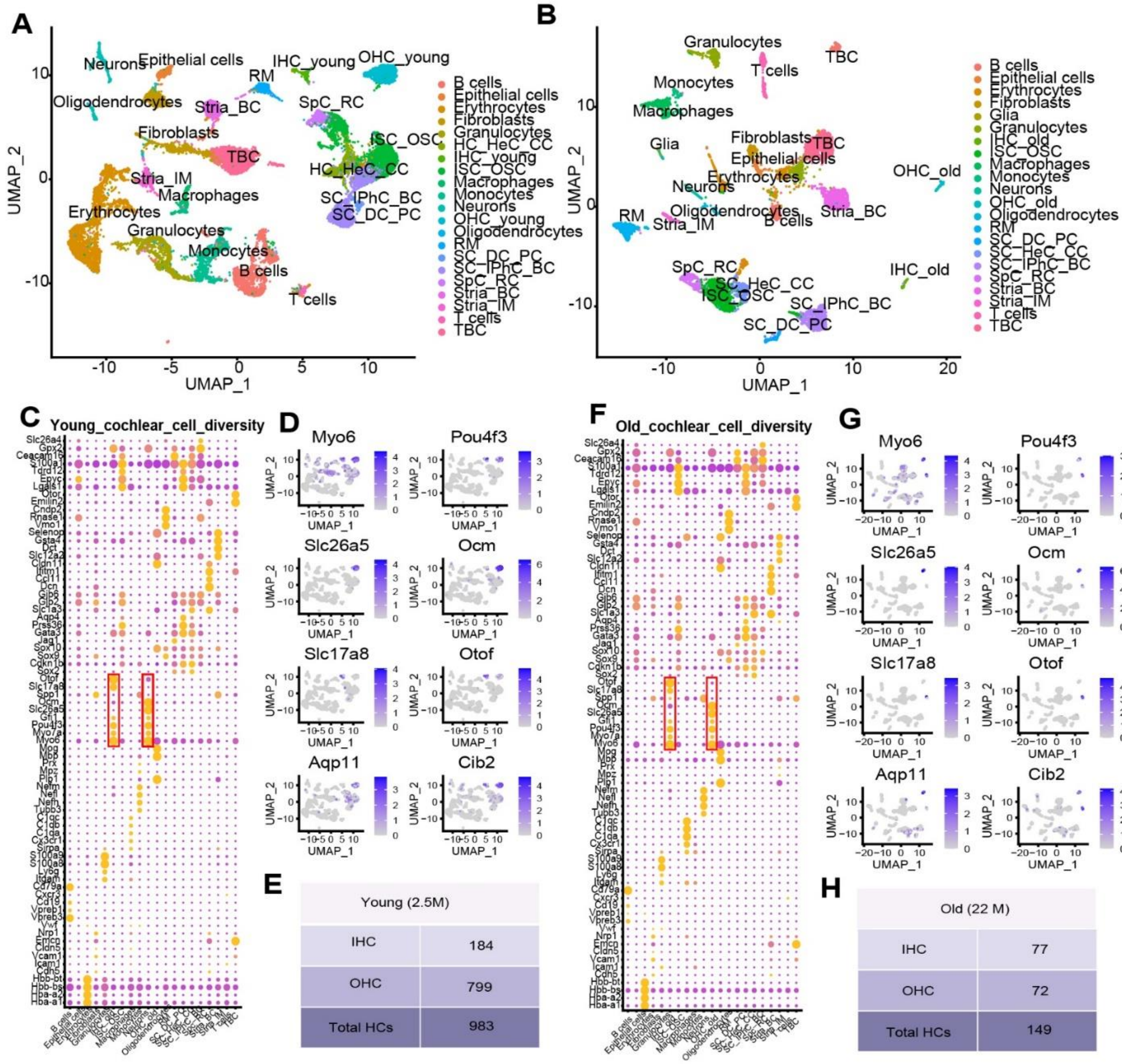

Figure S6: Young (2.5M) and old (22M) cochlear cell type distribution. (**A and B)** Uniform manifold approximation and projection (UMAP) plot shows the distribution of different cell types in young and old cochlear samples. (**C and F)** Dot plots indicating the expression of known marker genes used to identify the cell types and subsequent cluster annotation shown in B and C. The dot size represents the percentage of cells expressing the genes from each cluster, whereas the color indicates the expression level. Expression levels are normalized via z-score normalization. Thus, the average expression is zero, and positive or negative values indicate expression above or below average. (**D and G)** Feature plots demonstrating the HC-specific pan marker gene expression in the HC clusters identified in the UMAPs. (**E and H)** Number of inner and outer HCs identified from young and old cochlear samples for the downstream analysis.

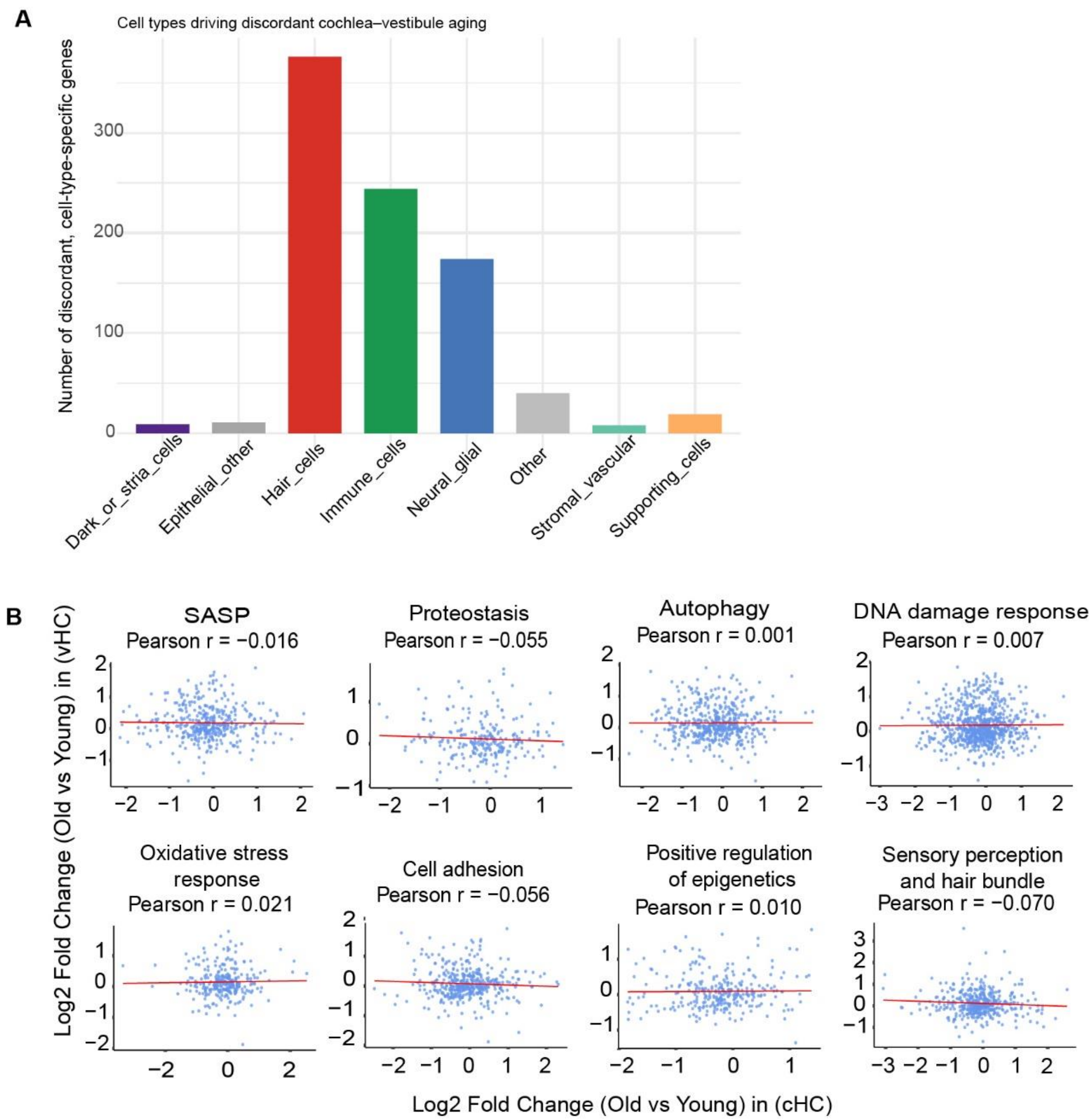

Figure S7: (A) Cell types that drive the highest number of discordant genes contribute to differences in global transcriptomic changes in the cochlea and the vestibular system **(B)** Linear correlation of age-related transcriptomic changes related to aging pathways in cochlear and vestibular HCs.

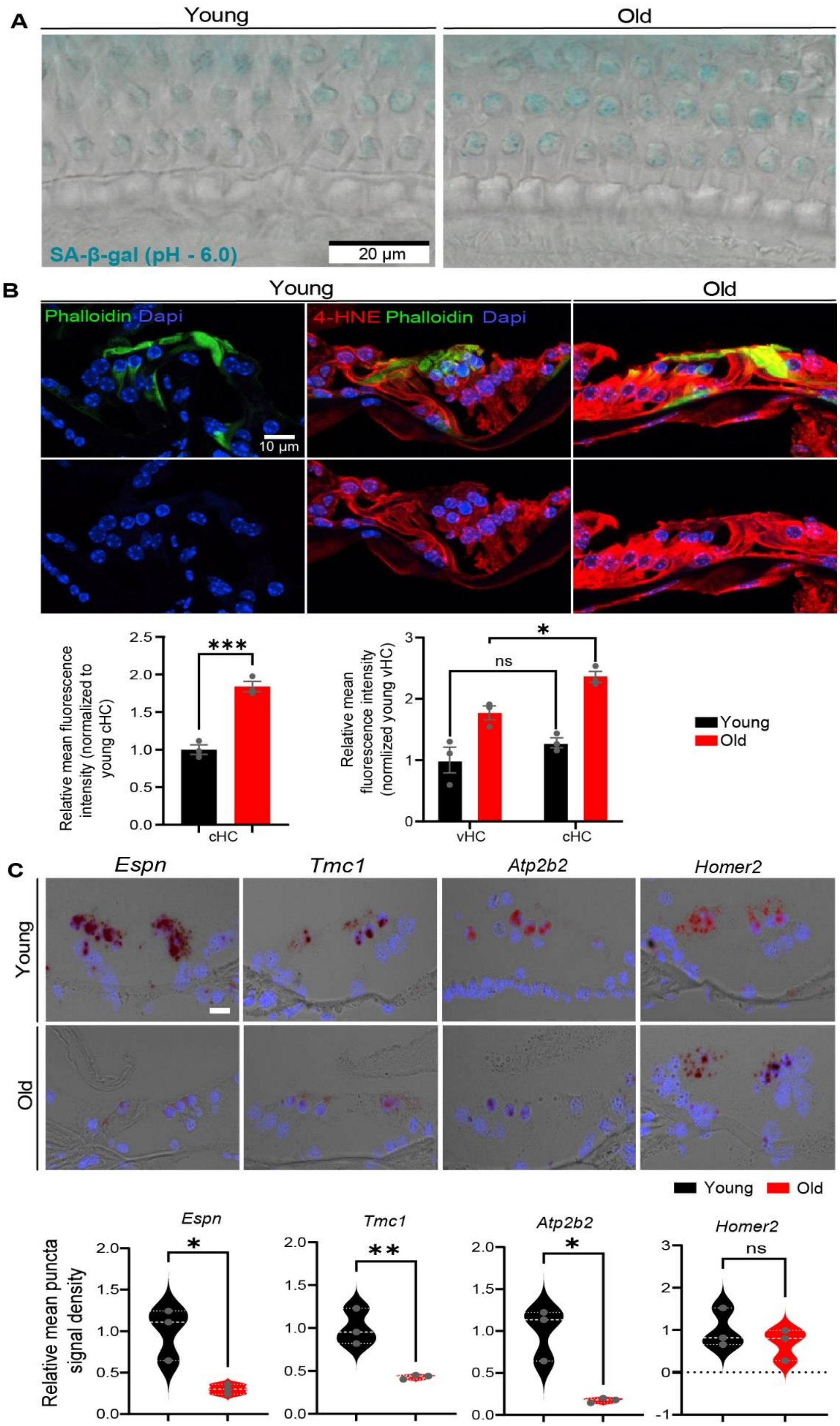
A
Young
Old
SA-β-gal (pH - 6.0)
20 μm
B
Young
Old
Phalloidin Dapi
4-HNE Phalloidin Dapi
10 μm
Relative mean fluorescence intensity (normalized to young cHC)
***
cHC
Relative mean fluorescence intensity (normlized young vHC)
*
ns
vHC
cHC
Young
Old
C
Espn
Tmc1
Atp2b2
Homer2
Young
Old
Young
Old
Relative mean puncta signal density
*
**
*
ns

Figure S8: **(A)** Senescence-associated β-galactosidase (SA-β-gal) activity at pH 6.0 in the cochlear HCs, indicating cellular senescence. **(B)** Representative images of 4-HNE expression in the cochlear HCs and quantifications. Left panel shows the relative mean fluorescence intensity of old cHCs normalized to young, while the right panel shows relative mean fluorescence intensity of young and old cHCs normalized to young vHCs. Both vestibular and cochlear samples were processed and immunostained at the same time to allow direct comparison. Mean intensity was quantified using FIJI. Data shown as mean ± SEM, ns-non-significant, *p <0.05, ***p <0.001 by unpaired (left) and paired (right) t-test. **(C)** RNAscope *in situ* hybridization of some key genes identified from the comparative analysis and their quantifications (n=3 per age group). Scale bar, 5 μm. Puncta signal density was quantified for each gene using FIJI. Data shown as mean ± SEM, ns-non-significant, individual data points represent independent biological replicates, *p <0.05, **p <0.01 by unpaired Student's t-test.

Table S3: Antibodies and RNAscope probes

Antibody	Manufacturer	Catalog Number	Dilution
**Primary:**			
Anti-ESPN	Protein tech	20717-1-AP	1:200
Anti-CCDC39	Sigma Prestige Antibodies	HPA035564	1:200
Anti- CCDC40	Bioss	bs-8091R	1:200
Anti- MYO6	Proteus	25-6791	1:200
Anti- MYO7A	Proteus	25-6790	1:200
Anti-acetylated-β-Tubulin	Sigma Millipore	T6793	1:200
Anti-SOX2	Invitrogen	14-9811-82	1:300
Anti-4-HNE	Abcam	ab46545	1:100
Anti-Oncomodulin	Swant	OMG4	1:200
**Secondary:**			
Phalloidin FlourTM 488	Invitrogen	A12379	1:200
Phalloidin FlourTM Plus 405	Invitrogen	A30104	1:200
Goat anti-rabbit Fluor 568	Invitrogen	A11011	1:200
Donkey anti-mouse Fluor 568	Invitrogen	A31570	1:200
Goat anti-rabbit Fluor 488	Invitrogen	A32731	1:200
Goat anti-mouse Fluor 488	Invitrogen	A11001	1:200
**RNAscope Probes**	**Manufacturer**	**Catalog Number**	**Dilution**
Espn	Advanced Cell Diagnostics (ACD) Biotechne	1061141	N/A
Tmc1		520911	
Atp2b2		1262061	
Fbxo2		524151	
Pou4f3		1740841	
Homer2		581231	
Gjb2		51881	
Hspb1		488361	
DapB		310043	
