# Supplementary material for: Molecular and Cellular Hallmarks of Age‐Related Vestibular Hair Cell Degeneration
Source: Adv Sci (Weinh). 2026 Jun 26:e76340. Online ahead of print. doi: 10.1002/advs.76340 (PMC13337082; doi:10.1002/advs.76340)

Supporting information

Figure 1 source data

1A-C

Young	Threshold
#1	-16.5
#2	-13.5
#3	-13.5
#4	-13.5
#5	-13.5
#6	-16.5
#7	-10.5
#8	-16.5
Old	-4.5
#1	-1.5
#2	-4.5
#3	-4.5
#4	-4.5
#5	-4.5
#6	-10.5
#7	1.5
#8	-7.5

1D

Young	mV
#1	10
#2	11
#3	9
#4	8
#5	10
#6	18
Old	
#1	10
#2	9
#3	17
#4	14
#5	9
#6	11

1E

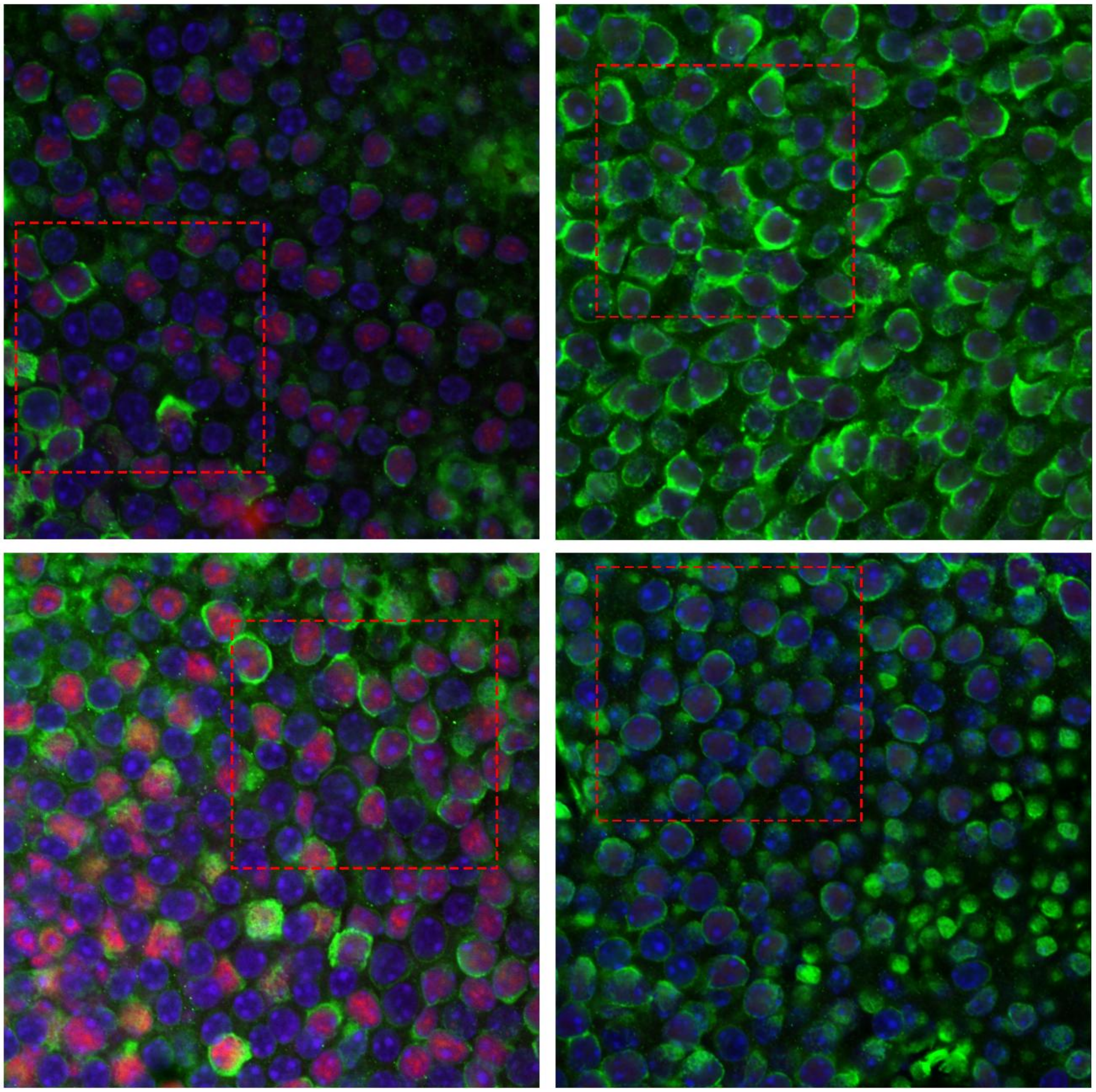

1F

Young	Total striolar HCs	Total extrastriolar HCs	Old	Total striolar HCs	Total extrastriolar HCs
#1	220	178	#1	220	259
#2	249	238	#2	255	238
#3	176	191	#3	201	191
#4	203	206	#4	182	206

Young	striolar		Young	extrastriolar	
	type I	type II		type I	type II
#1	113	115	#1	103	154
#2	158	166	#2	118	134
#3	126	59	#3	94	84
Old	type I	type II	Old	type I	type II
#1	107	105	#1	75	105
#2	91	89	#2	132	104
#3	174	81	#3	160	91

1G

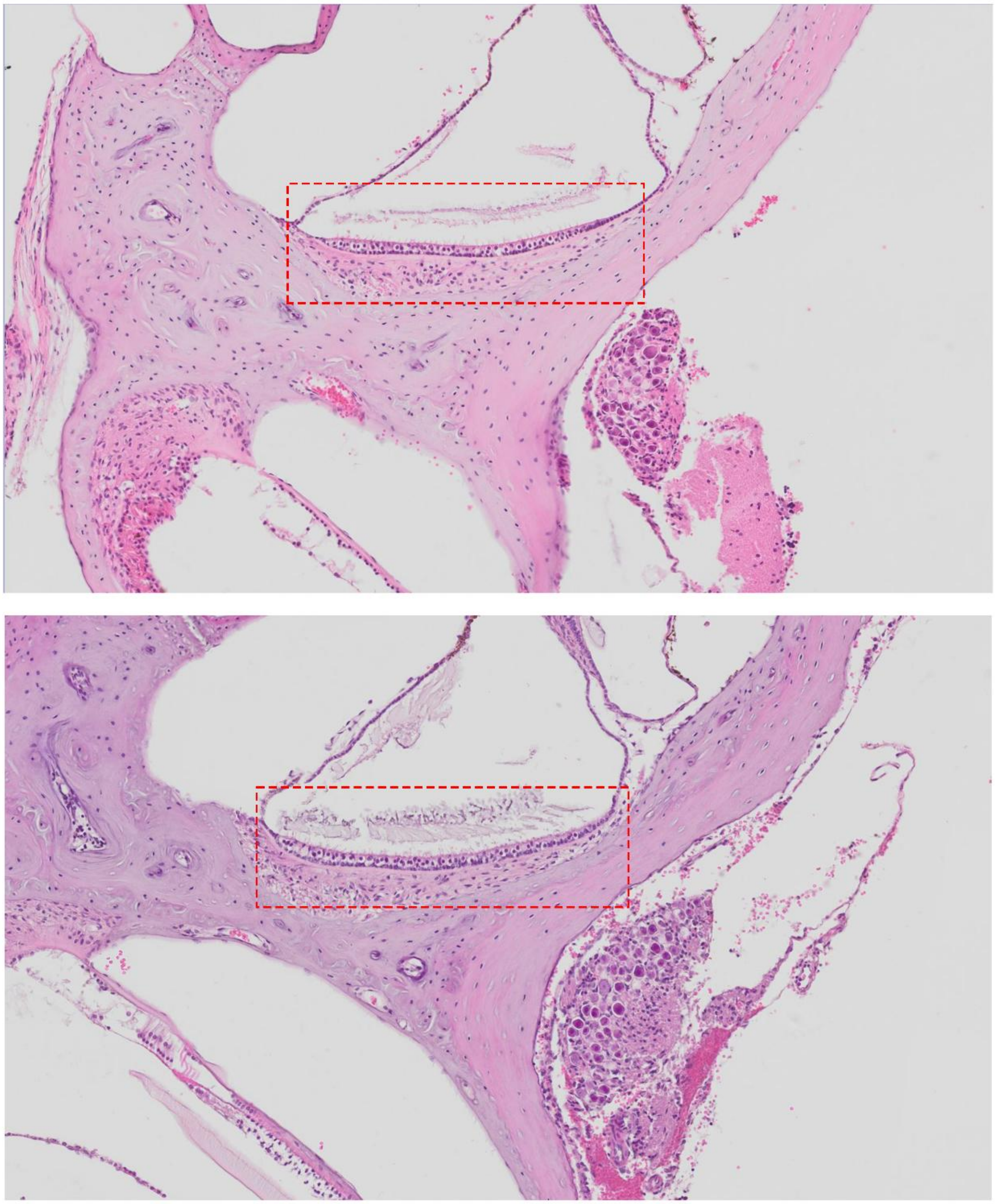

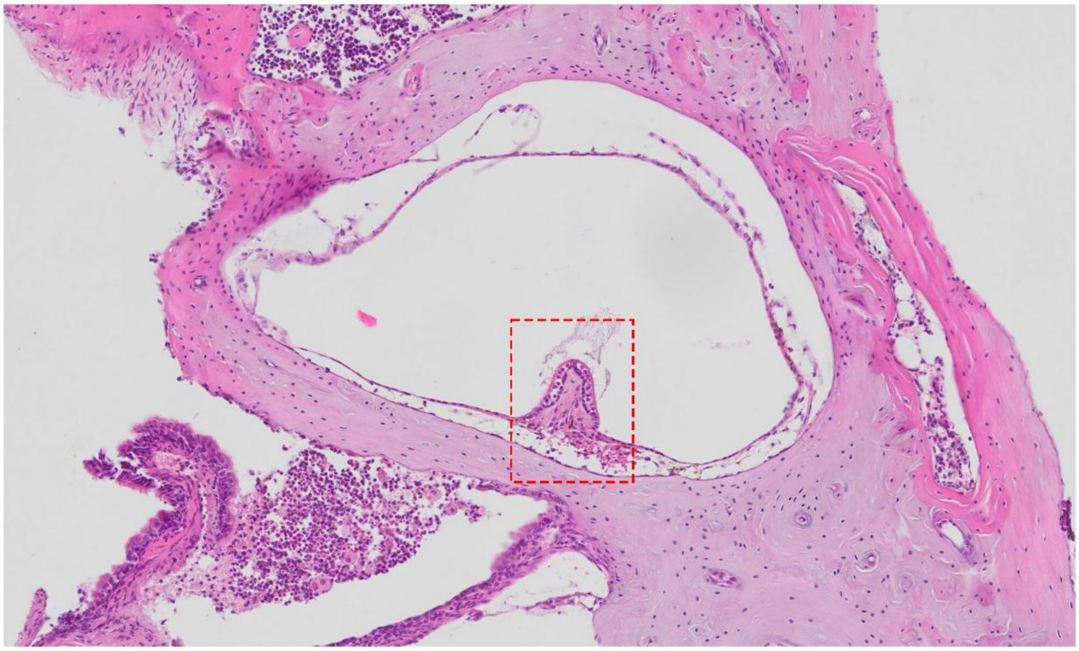

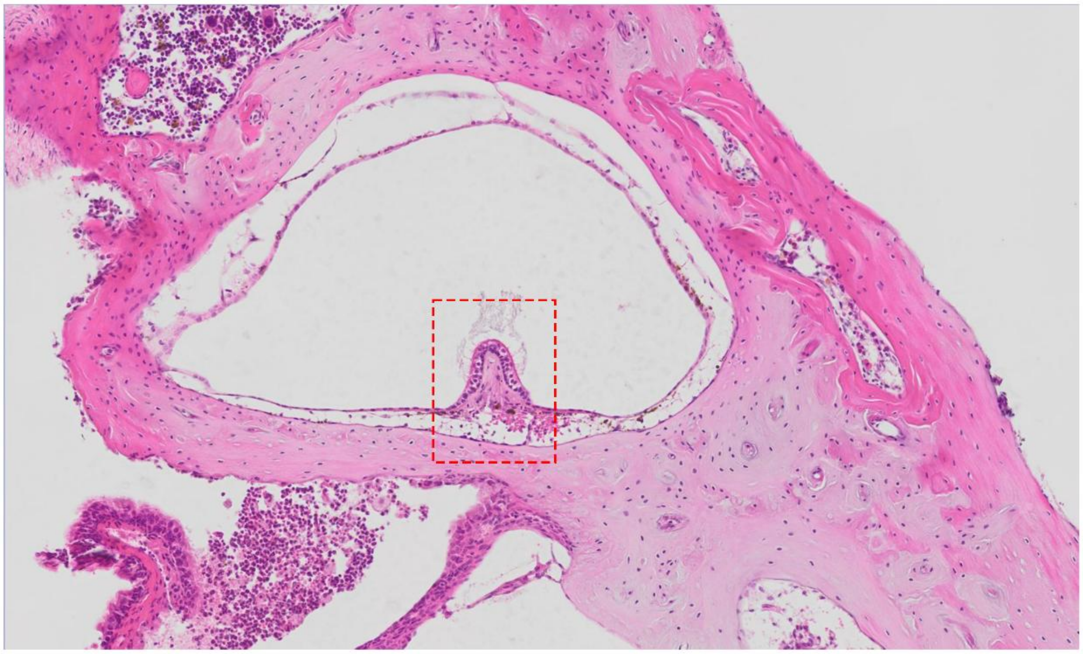

1H

Young	saccule		crista	
	type I	type II	type I	type II
#1	11.80	7.87	14.16	7.07
#2	15.35	9.05	13.76	8.79
#3	11.80	7.65	15.54	8.94
Old	type I	type II	type I	type II

#1	11.58	7.85	12.25	8.48
#2	12.66	9.05	10.85	6.72
#3	12.99	7.65	12.00	6.96

Figure 2 source data

2A

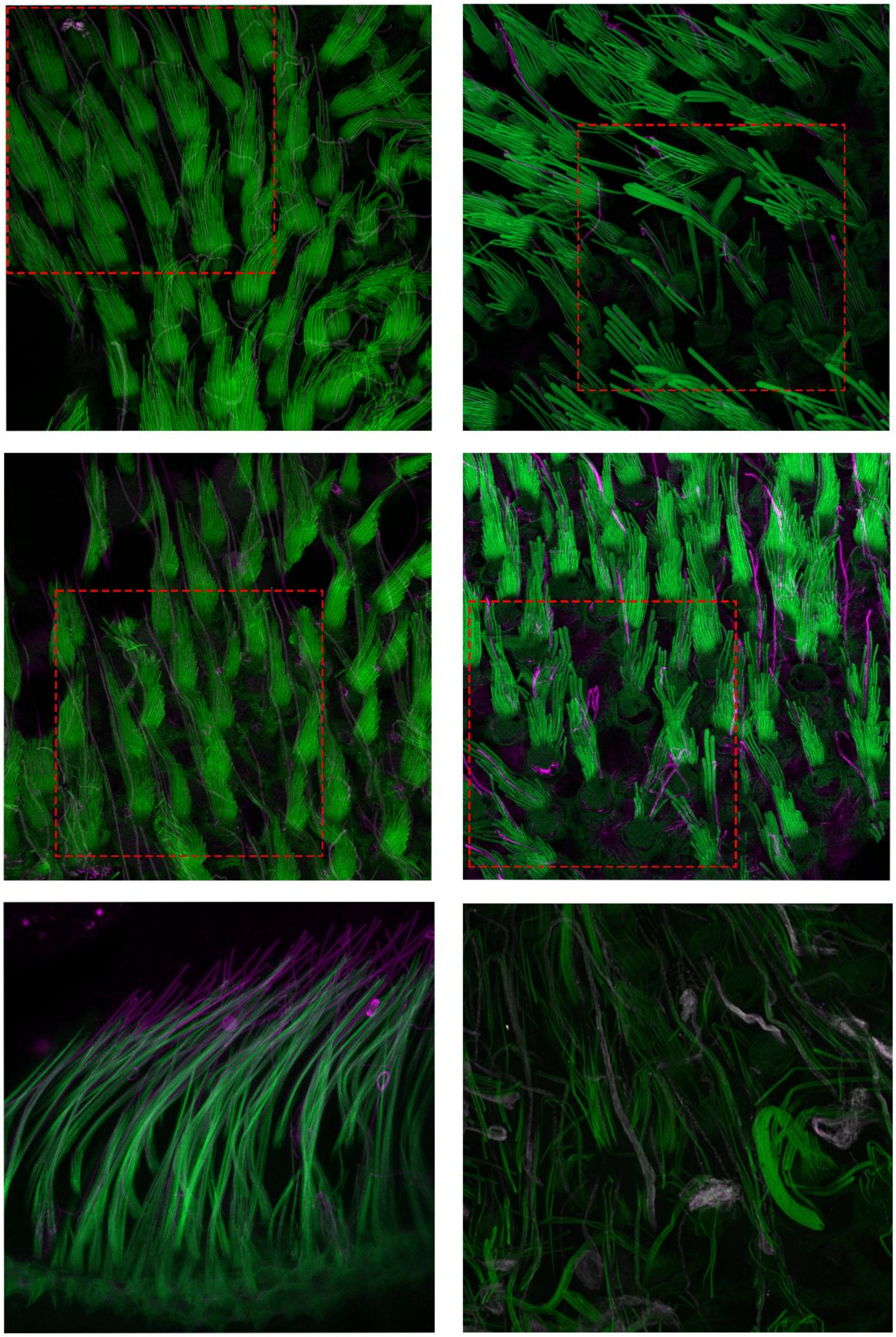

Note: The black regions within the young striolar and extarstriolar images is a result of uneven areas that was not fully captured within the acquired super-resolution z-stack not bundle or hair cell loss.

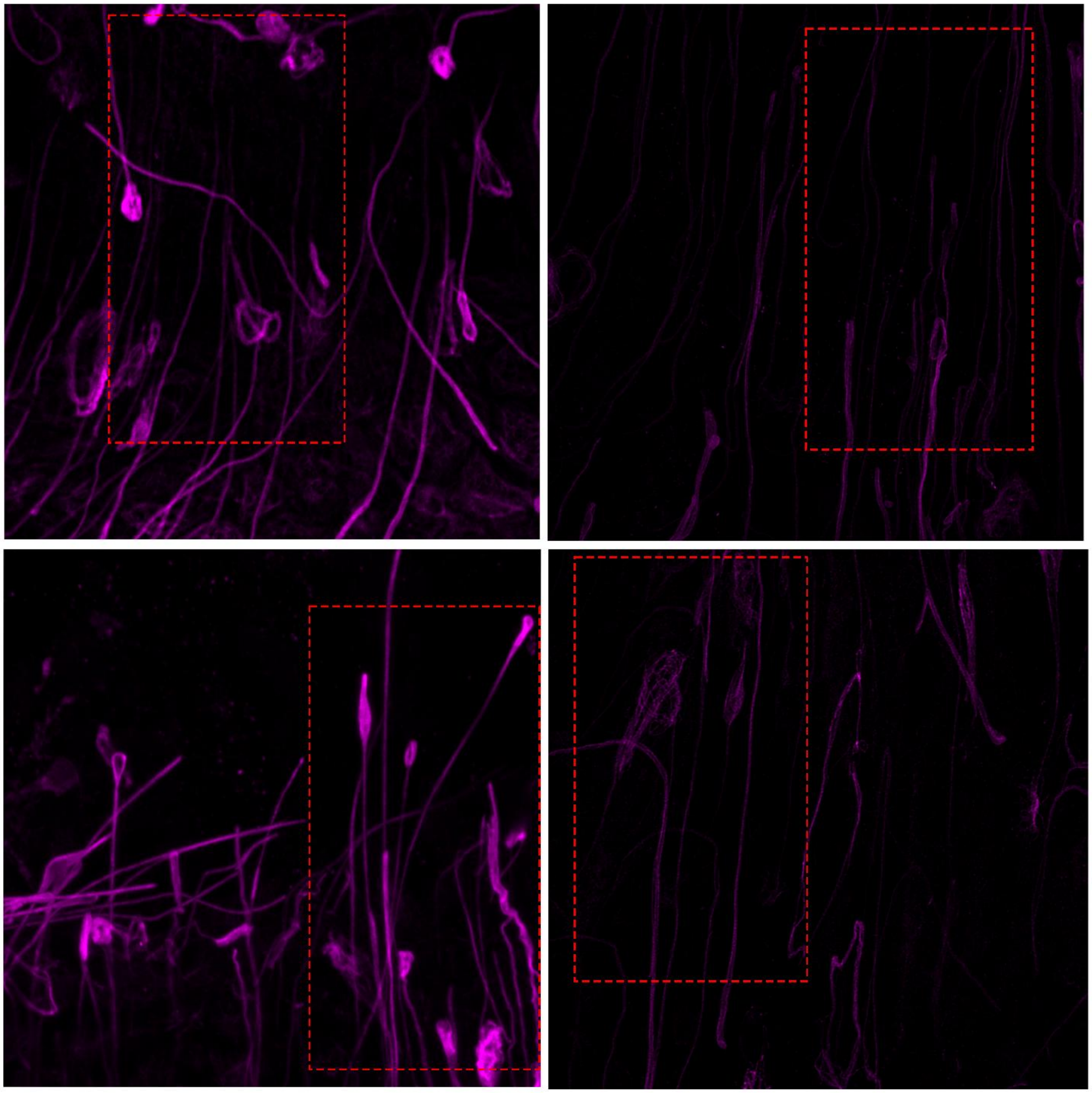

2B

	Extrastriolar	Total bundles/HCs	Degenerating bundles
Young	#1	47	0
	#2	119	0
	#3	114	2
	#4	81	2
	#5	96	2
Old	#1	178	47
	#2	102	23
	#3	103	20
	#4	52	22
	#5	50	31
	Striolar	Total bundles/HCs	Degenerating bundles
Young	#1	116	2
	#2	89	3
	#3	61	1
	#4	40	3
	#5	38	1
Old	#1	160	54
	#2	29	14
	#3	35	15
	#4	53	16
	#5	86	20
	Crista	Total bundles/HCs	Degenerating bundles
Young	#1	63	0
	#2	116	4
	#3	98	1
	#4	45	1
Old		Total bundles/HCs	Degenerating bundles
	#1	80	21
	#2	74	27
	#3	82	33
	#4	57	18

2C
Note: Cells marked by arrows around the edge of the tissue are not bundleless HCs.

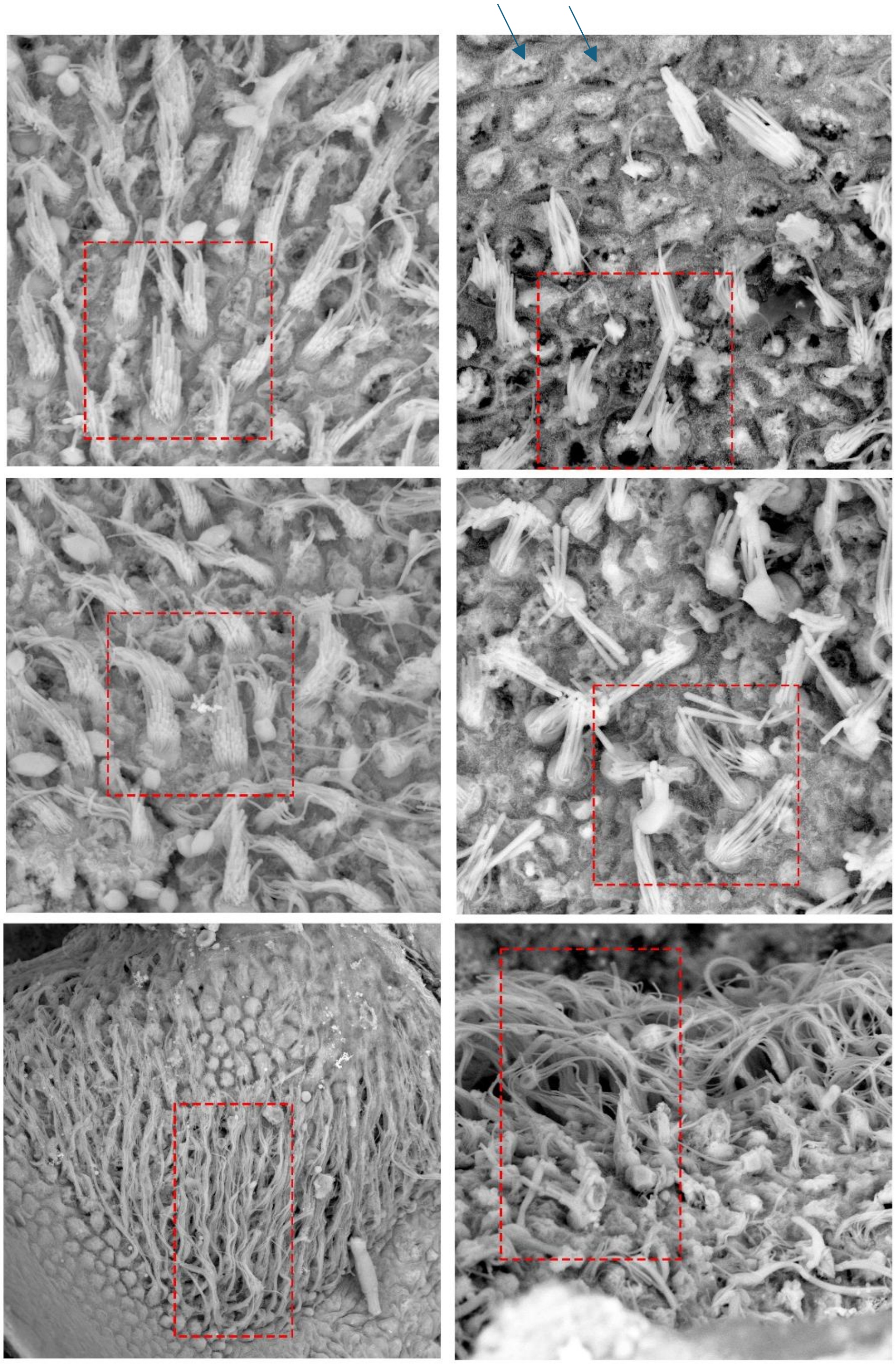

2D, E

Striolar		
Young	stereocilia	length
#1	36.833	10.160
#2	33.200	9.640
#3	24.200	10.320
#4	18.200	10.280
Old		
#1	10.000	8.254
#2	6.800	6.920
#3	15.875	8.989
Extrastriolar		
Young	stereocilia	length
#1	39.250	9.175
#2	38.000	9.680
#3	35.143	9.530
Old		
#1	16.500	7.182
#2	13.857	6.457
#3	16.000	5.152
#4	13.667	7.176
Crista		
Young	stereocilia	length
#1	28.500	23.400
#2	33.500	36.667
#3	25.200	35.388
Old		
#1	10.700	16.385
#2	15.571	25.000
#3	10.400	15.582

2F

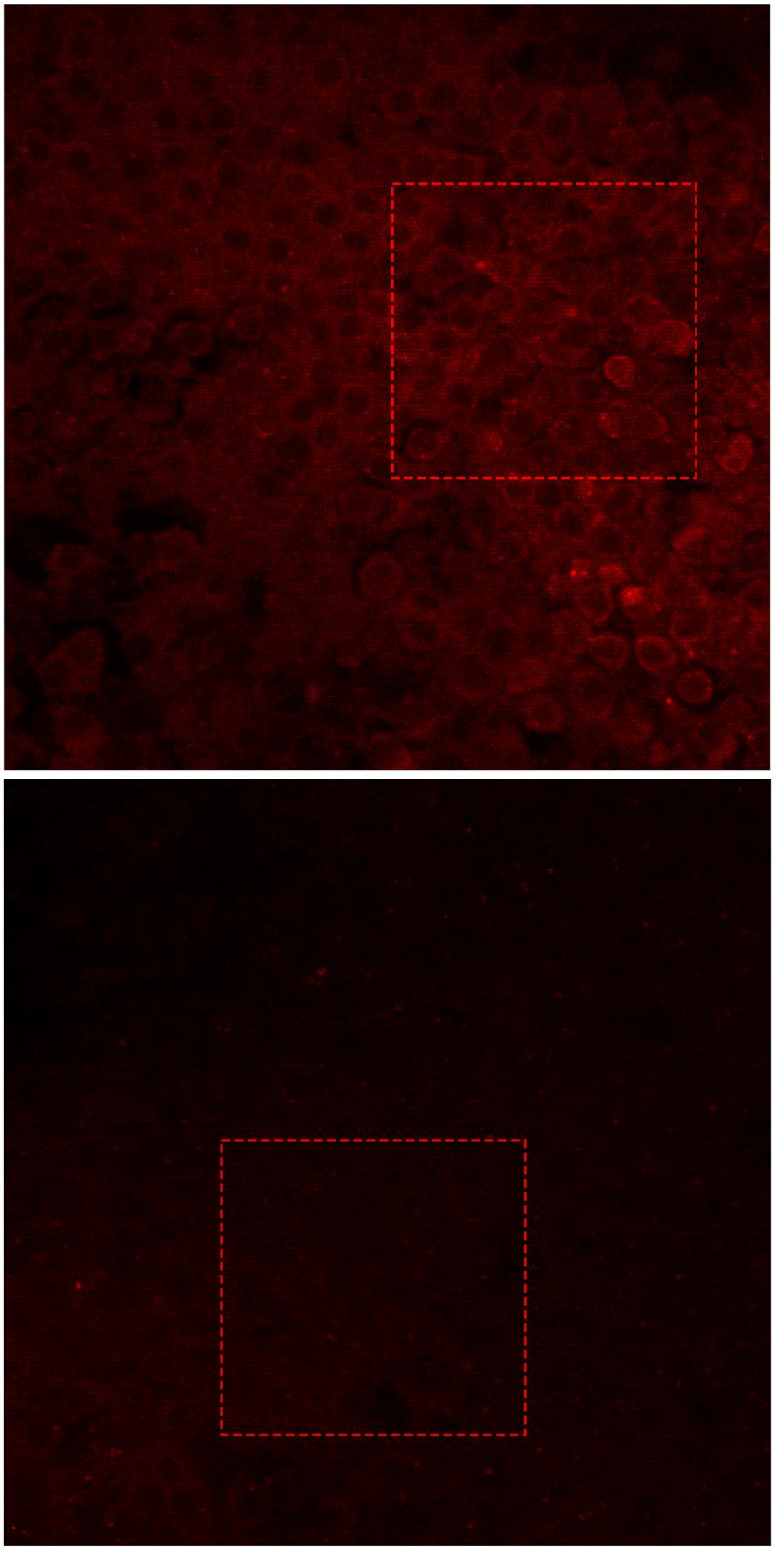

	Utricle	Crista
Young	Mean intensity	Mean intensity
#1	65.37775	83.0145
#2	49.60875	33.99625
#3	37.37925	55.15425
#4	33.94925	55.0235
#5	59.045	29.88625
Old	Mean Intensity	Mean intensity
#1	17.25975	13.4615
#2	16.19275	16.3
#3	8.951	8.06475
#4	15.15	17.4295
#5	5.8075	14.954

2G

	Young	Old
#1	0.82	0.32
#2	0.43	0.37
#3	0.91	0.22
#4	0.79	0.2
#5	0.61	0.16

Figure 3 source data

SASP

Gene	GWEIGHT	HCI_young	HCII_young	HCI_old	HCII_old
EWEIGHT		1	1	1	1
H3f3a	1	-0.541306	-0.456909	0.497678	0.500537
Prdx1	1	-0.026517	-0.806285	0.381609	0.451194
Ybx1	1	-0.363452	-0.61822	0.534809	0.446862
Mif	1	-0.552632	-0.443608	0.470169	0.526071
Cxcl14	1	-0.385982	-0.595046	0.401832	0.579197
Ypel3	1	-0.589083	-0.402102	0.48968	0.501506
Prdx2	1	-0.5988	-0.390267	0.493193	0.495874
Sod1	1	-0.556888	-0.439402	0.482246	0.514044
Raf1	1	0.643453	0.308684	-0.612816	-0.339321

Cdk4	1	-0.056567	0.791941	-0.590445	-0.144929
Rb1cc1	1	-0.494516	-0.505306	0.512101	0.487721
Trp53bp1	1	-0.65371	-0.314529	0.43417	0.534069
Pawr	1	-0.597782	-0.381784	0.397254	0.582312
Cdkn1b	1	-0.409336	-0.471804	0.107673	0.773467
Nos3	1	-0.704851	-0.23362	0.405041	0.533431
Mdm4	1	-0.552884	-0.442538	0.536742	0.45868
Kras	1	-0.541196	-0.452752	0.433114	0.560834
Tbx2	1	-0.497843	-0.020109	-0.296727	0.814679
Jun	1	-0.214131	0.037504	-0.596209	0.772836
Nfe2l2	1	-0.374188	0.362577	-0.597707	0.609318
Fzr1	1	-0.312133	-0.637693	0.325186	0.62464
Mdm1	1	-0.438804	0.8122	-0.384271	0.010875
Bcl2	1	-0.569202	-0.378267	0.678764	0.268706
Pik3r1	1	-0.588697	-0.381704	0.621028	0.349374
Pik3ca	1	-0.56715	-0.422086	0.420829	0.568407
Mapk1	1	-0.444969	-0.550428	0.455129	0.540268
Mapk3	1	-0.410554	-0.580451	0.537189	0.453817
Zmpste24	1	-0.556116	-0.406058	0.658517	0.303656
Pik3r2	1	-0.559008	-0.420367	0.364762	0.614613
Mapk9	1	-0.553579	-0.441387	0.454182	0.540784
Parp1	1	0.175611	-0.857365	0.369962	0.311792
Cdkn1a	1	-0.646896	-0.234407	0.177753	0.70355
Hif1a	1	-0.262549	-0.688145	0.422358	0.528336
B2m	1	-0.225547	-0.696755	0.322696	0.599607
Vegfa	1	0.251478	-0.862887	0.254347	0.357062
Igfbp5	1	-0.499989	-0.499989	0.504682	0.495296
Pten	1	-0.499886	-0.499886	0.484755	0.515016
Prdx3	1	-0.499971	-0.499971	0.492373	0.50757
Nfkb2	1	-0.173477	-0.732975	0.348758	0.557693
E2f2	1	-0.496388	-0.496388	0.581225	0.411552
Mmp9	1	-0.401529	-0.401529	-0.019836	0.822894
Cdk1	1	-0.288675	-0.288675	0.866025	-0.288675
Lmna	1	0.435445	0.546059	-0.614309	-0.367195
Lmnb1	1	0.649509	-0.611009	-0.338675	0.300175
Cdk2	1	0.440281	0.487685	-0.726429	-0.201538
Cdk5	1	0.682467	0.150735	-0.703311	-0.129891
Cdk6	1	0.628515	0.309432	-0.28276	-0.655186
Cdk7	1	0.600163	0.378485	-0.58334	-0.395308
Cdk8	1	0.53174	0.466695	-0.521708	-0.476727

Cdk9	1	0.451075	0.545934	-0.525652	-0.471357
Cdk10	1	0.679294	0.033293	-0.732831	0.020245
Cdk11b	1	-0.583764	-0.402826	0.421635	0.564955
Hira	1	-0.531721	-0.415748	0.255128	0.692341
Asf1a	1	0.240159	0.148877	-0.84432	0.455284
Ubn1	1	-0.595052	-0.391842	0.441517	0.545377
Cabin1	1	-0.510473	-0.488277	0.532926	0.465824
Hp1bp3	1	0.201963	-0.86036	0.362732	0.295664

Oxidative stress response

Gene	GWEIGHT	HCI_young	HCII_young	HCI_old	HCII_old
EWEIGHT		1	1	1	1
Mt1	1	-0.591231	-0.399472	0.487553	0.50315
mt-Co1	1	-0.499886	-0.499886	0.484789	0.514983
mt-Cytb	1	-0.5	-0.5	0.499102	0.500897
mt-Nd1	1	-0.499985	-0.499985	0.494565	0.505405
mt-Nd3	1	-0.418672	-0.575171	0.500835	0.493008
Glrx	1	-0.367118	-0.603818	0.606444	0.364492
Stk24	1	-0.666439	-0.290069	0.562709	0.393799
Sp1	1	-0.704442	-0.240005	0.481397	0.46305
Lonp1	1	-0.701697	-0.238203	0.537683	0.402217
Slc7a11	1	-0.631405	-0.324413	0.618067	0.337751
Plekha1	1	-0.368817	-0.539451	0.170857	0.737411
Rcan2	1	-0.485283	-0.508744	0.42074	0.573287
Sirpa	1	-0.650851	-0.020092	-0.083401	0.754344
Agap3	1	-0.806858	-0.018501	0.349319	0.47604
Ndufa6	1	-0.315492	-0.655117	0.474217	0.496392
Pax2	1	-0.498234	-0.498234	0.438856	0.557612
Ncoa7	1	-0.498685	-0.498685	0.447442	0.549929
Prdx3	1	-0.499971	-0.499971	0.492373	0.50757
Stox1	1	-0.046365	-0.654821	-0.05142	0.752606
Srxn1	1	-0.235669	-0.706798	0.451562	0.490906
Casp3	1	-0.329604	-0.644016	0.450499	0.523121
Net1	1	-0.297264	-0.627608	0.250247	0.674625
Rps3	1	-0.635172	-0.343222	0.481233	0.497162
Ppia	1	-0.500604	-0.498807	0.475467	0.523945
Gpx4	1	-0.546648	-0.448838	0.451785	0.543701
Atp2a2	1	-0.596576	-0.331099	0.692069	0.235607

Kpna4	1	-0.586391	-0.346569	0.241948	0.691012
Atox1	1	-0.612612	-0.368459	0.428508	0.552563
Ralbp1	1	-0.725447	-0.203544	0.444687	0.484304
Apod	1	-0.488115	-0.511629	0.510696	0.489049
Prdx2	1	-0.5988	-0.390267	0.493193	0.495874
Sod1	1	-0.556888	-0.439402	0.482246	0.514044
Ndufa12	1	-0.465896	-0.532965	0.503181	0.495679
Car3	1	-0.394178	-0.591344	0.560989	0.424533
Hnrnpd	1	-0.606389	-0.378399	0.538237	0.446551
Bcl2	1	-0.569202	-0.378267	0.678764	0.268706
Idh1	1	-0.75763	-0.135736	0.511957	0.381409
Nme5	1	-0.360368	-0.622327	0.490893	0.491801
Arl6ip5	1	-0.605906	-0.372247	0.399865	0.578289
Cflar	1	-0.726762	-0.166206	0.295657	0.59731
Fer	1	-0.70005	-0.238785	0.391298	0.547538
Lias	1	-0.717707	-0.214463	0.513155	0.419016
Trap1	1	-0.777761	-0.091206	0.366592	0.502374
Pdcd10	1	-0.250792	-0.694997	0.531303	0.414487
Gstp1	1	-0.247374	-0.692829	0.378436	0.561766
Hmox2	1	-0.367969	-0.616322	0.47927	0.505021
Ndufs8	1	-0.273416	-0.678624	0.551921	0.40012
Nox1	1	0.517083	0.471003	-0.600402	-0.387684
Nox3	1	0.452926	0.417555	-0.087652	-0.78283
Nox4	1	-0.288675	0.866025	-0.288675	-0.288675
Sod3	1	0.451657	0.544572	-0.538185	-0.458044
Cat	1	0.469096	0.499675	-0.659049	-0.309723
Gpx2	1	-0.487821	-0.502143	0.594652	0.395312
Gpx3	1	0.402531	0.579484	-0.590984	-0.391031
Prdx1	1	-0.026517	-0.806285	0.381609	0.451194
Prdx4	1	-0.497525	-0.497525	0.567794	0.427256
Prdx6	1	0.586644	0.394464	-0.393188	-0.58792
Hif1a	1	-0.262549	-0.688145	0.422358	0.528336
Keap1	1	-0.09955	0.443333	-0.777883	0.434101
Nrf1	1	-0.706814	-0.22638	0.549488	0.383706
Atf5	1	0.670388	0.04945	-0.740084	0.020247
Mtf1	1	-0.85635	0.163978	0.339618	0.352754
Fth1	1	-0.499584	-0.499584	0.528433	0.470734
Ftl1	1	-0.54749	-0.449442	0.524161	0.472771
Cox4i1	1	-0.397849	-0.5754	0.622685	0.350563
Cox6a1	1	-0.547128	-0.450532	0.496703	0.500957

Cox7a1	1	-0.274537	-0.60178	0.139391	0.736926
Cyb5r3	1	0.298594	-0.865345	0.309977	0.256774
Fis1	1	-0.555164	-0.441418	0.484972	0.511611
Mfn1	1	-0.84167	0.123025	0.263848	0.454797
Mfn2	1	-0.761441	-0.134261	0.425035	0.470667
Aifm1	1	-0.499977	-0.499977	0.493129	0.506824

Inflammaging

Genes	GWEIGHT	HCI_young	HCII_young	HCI_old	HCII_old
EWEIGHT		1	1	1	1
Ptgds	1	-0.461165	-0.53606	0.535611	0.461614
Gpx2	1	-0.487821	-0.502143	0.594652	0.395312
Gpx1	1	0.341798	0.586818	-0.232303	-0.696314
Stat3	1	0.052296	0.338965	-0.830385	0.439123
Stat1	1	0.531114	0.46002	-0.582535	-0.408599
Ptgs2	1	0.501179	0.481315	-0.359893	-0.622601
Ncf1	1	0.447262	0.475272	-0.733791	-0.188743
Myd88	1	0.542659	0.395478	-0.702593	-0.235544
Mapk8	1	-0.126861	-0.473738	-0.237808	0.838407
Stat6	1	0.612041	0.373445	-0.506048	-0.479438
Crppa	1	0.481315	0.511398	-0.580226	-0.412487
Mapk14	1	-0.523146	-0.456728	0.352749	0.627125
Nfkb1	1	0.12196	-0.792907	0.079213	0.591734
Fadd	1	-0.203859	-0.3964	-0.257164	0.857424
Pla2g6	1	0.677245	-0.293772	-0.628492	0.24502
Irak4	1	0.136316	0.744257	-0.299229	-0.581344
Sod3	1	0.451657	0.544572	-0.538185	-0.458044
Tlr3	1	0.304877	0.541809	-0.780453	-0.066233
Tlr4	1	0.469865	0.399953	-0.087798	-0.78202
Tyk2	1	0.449974	0.435465	-0.114168	-0.771271
Alox12	1	0.379766	0.607216	-0.493491	-0.493491
Tgfb1	1	0.48776	0.492329	-0.349661	-0.630428
Tlr7	1	0.503262	0.493807	-0.44464	-0.552429
Tlr8	1	0.514733	0.485046	-0.49989	-0.49989
Jak1	1	-0.346259	-0.632257	0.460892	0.517624
Nos3	1	-0.704851	-0.23362	0.405041	0.533431
Mapk1	1	-0.444969	-0.550428	0.455129	0.540268
Mapk3	1	-0.410554	-0.580451	0.537189	0.453817
Jak2	1	-0.122815	-0.760791	0.353111	0.530496

Irak1	1	-0.339351	-0.637789	0.470342	0.506798
Rela	1	-0.499776	-0.499776	0.520951	0.4786
Tradd	1	0.23951	-0.757375	-0.083796	0.601661
Nfkb2	1	-0.173477	-0.732975	0.348758	0.557693
Alox15	1	-0.029246	-0.636526	-0.098597	0.764368
Ncf2	1	0.36071	0.621394	-0.463712	-0.518391
Relb	1	-0.351744	0.86269	-0.227993	-0.282953
Pla2g4a	1	0.508564	0.459221	-0.307577	-0.660208
Nos2	1	0.514146	0.479697	-0.420492	-0.573351
Tlr2	1	0.592729	0.39087	-0.413827	-0.569772
Ccl11	1	0.717845	0.207678	-0.381298	-0.544224
Ccl19	1	0.48234	0.502817	-0.371631	-0.613526
Ccl2	1	0.362305	0.620843	-0.491574	-0.491574
Jak3	1	-0.288675	0.866025	-0.288675	-0.288675
Ccl20	1	0.849167	-0.363216	-0.122736	-0.363216
Ptger2	1	0.866025	-0.288675	-0.288675	-0.288675
Cxcl1	1	0.866025	-0.288675	-0.288675	-0.288675
Cxcl2	1	0.714935	-0.468707	0.222479	-0.468707
Ncf4	1	0.696079	-0.474914	0.253749	-0.474914
Cybb	1	0.668938	-0.482261	0.295585	-0.482261
Cxcl3	1	0.828623	-0.394889	-0.038846	-0.394889
Cxcl10	1	0.828623	-0.394889	-0.038846	-0.394889
Ccl8	1	0.548178	-0.498778	0.449379	-0.498778
Cxcl5	1	0.698109	-0.474292	0.250475	-0.474292
Itih2	1	0.818682	-0.011435	-0.447245	-0.360002
Cxcl14	1	-0.385982	-0.595046	0.401832	0.579197
Ccl12	1	-0.288675	-0.288675	0.866025	-0.288675
Il1a	1	-0.288675	-0.288675	0.866025	-0.288675
Il1b	1	-0.497322	0.570405	0.42424	-0.497322
Il5ra	1	0.336577	0.629689	-0.375027	-0.591239
Tnf	1	0	0	0	0
Hif1a	1	-0.262549	-0.688145	0.422358	0.528336
Ifi27l2a	1	-0.016408	0.659247	-0.744801	0.101961
Trem2	1	0.037542	0.663984	0.043982	-0.745507

Proteostasis

Gene	GWEIGHT	HCI_young	HCII_young	HCI_old	HCII_old
EWEIGHT		1	1	1	1
Hspa5	1	-0.31687	0.849482	-0.13189	-0.400723

Usp47	1	0.385146	0.324178	0.142832	-0.852156
Usp1	1	0.763714	-0.040598	-0.638726	-0.084391
Usp15	1	-0.796099	0.021559	0.568661	0.205879
Usp8	1	-0.823184	0.022082	0.422895	0.378207
Ube3c	1	-0.60698	0.731296	-0.273172	0.148857
Dnajb1	1	-0.863287	0.249885	0.261088	0.352314
Dnajc9	1	-0.780761	-0.088161	0.383821	0.4851
Atf6	1	-0.41888	-0.565704	0.591829	0.392756
Dnajb14	1	0.456884	0.540827	-0.47596	-0.521751
Ube2e2	1	0.605994	0.381052	-0.508385	-0.478661
Cul1	1	0.584318	0.407775	-0.505196	-0.486897
Rpn2	1	0.686938	0.263364	-0.415071	-0.535231
Itch	1	0.626844	0.353854	-0.512367	-0.468331
Usp33	1	0.446824	0.53419	-0.360517	-0.620497
Ube2q1	1	0.48985	0.49558	-0.612948	-0.372481
Herc2	1	0.516241	0.422871	-0.707998	-0.231114
Usp20	1	0.44942	0.514372	-0.667631	-0.296161
Hspa2	1	0.299732	0.051043	-0.825785	0.47501
Ubqln4	1	0.323512	0.471895	-0.819793	0.024386
Dnajc24	1	0.502171	0.496312	-0.460409	-0.538074
Fbxo31	1	0.508106	0.487561	-0.562778	-0.432889
Rnf38	1	0.600524	0.383108	-0.558278	-0.425353
Ube2j2	1	0.180078	0.738623	-0.460719	-0.457981
Dnajc5	1	0.636464	0.333946	-0.406	-0.564409
Uchl5	1	0.450404	0.543405	-0.560225	-0.433584
Rnf180	1	0.455748	0.531811	-0.598262	-0.389298
Otulin	1	0.52927	0.46981	-0.493568	-0.505512
Vcpip1	1	0.489719	0.509486	-0.526003	-0.473202
Dnajb9	1	0.509095	0.490668	-0.512218	-0.487545
Usp38	1	0.501451	0.491234	-0.581561	-0.411124
Herc4	1	0.470296	0.52805	-0.470566	-0.52778
Rnft1	1	0.649886	0.297586	-0.331893	-0.615579
Agbl4	1	0.485988	0.475779	-0.287296	-0.674471
Hspb1	1	0.499455	0.499634	-0.529717	-0.469372
Uspl1	1	-0.282558	0.864489	-0.332791	-0.24914
Rnf170	1	0.385504	0.541678	-0.716709	-0.210474
Fbxw8	1	0.325824	0.633976	-0.605008	-0.354793
Trib2	1	0.32116	0.599798	-0.698194	-0.222765
Fbxo8	1	0.475096	0.523269	-0.531641	-0.466724
Fbxo25	1	0.434885	0.558855	-0.447905	-0.545836

Rnf41	1	0.519671	0.479615	-0.48194	-0.517345
Usp5	1	0.460941	0.537582	-0.496549	-0.501975
Usp30	1	0.164983	0.741574	-0.530502	-0.376055
Usp43	1	0.647469	0.092774	-0.756254	0.016011
Eif2ak3	1	0.30376	0.661869	-0.524275	-0.441355
Fbxo10	1	0.401923	0.584391	-0.565739	-0.420575
Tnip2	1	0.485703	0.513607	-0.477393	-0.521917
Laptm5	1	0.514531	0.484937	-0.482051	-0.517417
Fbxo30	1	0.516795	0.431187	-0.25301	-0.694972
Fbxo5	1	0.206472	0.707446	-0.59708	-0.316837
Hspb2	1	0.46889	0.522116	-0.404694	-0.586311
Ptk2b	1	0.187757	0.722941	-0.338264	-0.572434
Hspa4	1	-0.56183	-0.430853	0.44156	0.551124
Hspa4l	1	-0.443519	-0.55293	0.474709	0.521739
Dnajb2	1	-0.599841	-0.385516	0.437432	0.547925
Dnaja1	1	-0.384484	-0.602921	0.469459	0.517945

Autophagy

Genes	GWEIGHT	HCI_young	HCII_young	HCI_old	HCII_old
EWEIGHT		1	1	1	1
Tsc1	1	0.472069	0.518348	-0.400335	-0.590083
Tsc2	1	-0.162547	-0.72256	0.269668	0.615439
Stk38l	1	0.546072	0.440757	-0.594963	-0.391865
Rptor	1	0.483327	0.513941	-0.548577	-0.448691
Atg5	1	0.49753	0.49868	-0.436601	-0.559609
Atg16l2	1	0.468268	0.502299	-0.654723	-0.315845
Rraga	1	0.369871	0.614744	-0.50894	-0.475674
Golga2	1	0.720442	0.118081	-0.659531	-0.178993
Kdm4a	1	0.659695	0.288957	-0.349152	-0.599499
Rheb	1	-0.665679	-0.299428	0.455194	0.509913
Bcl2	1	-0.569202	-0.378267	0.678764	0.268706
Bnip3	1	-0.689569	0.010337	0.722833	-0.043601
Nbr1	1	-0.532894	-0.465531	0.520167	0.478258
Stat3	1	0.052296	0.338965	-0.830385	0.439123
Sqstm1	1	-0.586621	0.777109	0.034822	-0.22531
Sesn1	1	0.241732	0.637613	-0.167284	-0.712061
Ppargc1a	1	0.380927	0.404884	0.044253	-0.830063
Ambra1	1	0.55541	0.44098	-0.48001	-0.516379
Nrbf2	1	0.340444	0.613051	-0.640592	-0.312903
Atg14	1	0.490213	0.497406	-0.382943	-0.604676

Mtor	1	0.499162	0.500835	-0.501498	-0.498499
Akt1	1	0.280585	0.644293	-0.660503	-0.264375
Tecpr2	1	0.342893	0.619206	-0.346527	-0.615571
Slc7a5	1	0.464887	0.492437	-0.682563	-0.274762
Atg9b	1	0.506934	0.477507	-0.368845	-0.615596
Atg7	1	0.576457	0.404027	-0.599296	-0.381188
Atg10	1	0.582704	0.406771	-0.442471	-0.547004
Lepr	1	0.541962	0.428924	-0.325771	-0.645116
Atg4b	1	-0.5377	0.800731	-0.264022	0.000991
Becn1	1	-0.565658	-0.392485	0.296086	0.662057
Prkaa1	1	0.531861	0.454435	-0.603201	-0.383095
Prkaa2	1	0.269925	0.414557	-0.852645	0.168164
Ulk1	1	-0.191165	-0.730018	0.516453	0.40473
Atg13	1	-0.500428	-0.393213	0.134043	0.759597
Zbtb24	1	-0.734225	0.541738	0.369107	-0.17662
Atg9a	1	0.75364	0.096627	-0.600081	-0.250186
Atg16l1	1	0.514629	-0.731787	-0.188124	0.405282
Atg12	1	-0.624075	-0.357683	0.509155	0.472604
Atg3	1	-0.690859	-0.261494	0.49673	0.455624
Atg4a	1	-0.05637	-0.795957	0.430894	0.421433
Fundc1	1	-0.299942	-0.6657	0.464839	0.500802
Pink1	1	-0.039507	-0.617731	0.776317	-0.119079

DNA damage response

Genes	GWEIGHT	HCI_young	HCII_young	HCI_old	HCII_old
EWEIGHT		1	1	1	1
Uchl5	1	0.450404	0.543405	-0.560225	-0.433584
Rpa3	1	0.550768	0.438324	-0.582221	-0.406872
Rad50	1	0.531887	0.46611	-0.529315	-0.468682
Xpc	1	-0.431287	0.845885	-0.286434	-0.128163
Trim28	1	0.843209	-0.12219	-0.444575	-0.276444
Mdm2	1	-0.107273	0.128212	0.686609	-0.707547
Trp53bp2	1	0.656005	0.31336	-0.513221	-0.456144
Apex1	1	0.512368	0.168022	-0.82896	0.14857
Sirt1	1	0.307074	0.660944	-0.47121	-0.496809
H2afx	1	0.789465	-0.010295	-0.580747	-0.198424
Atm	1	0.082634	0.776823	-0.328771	-0.530686
Xrcc1	1	0.526777	0.43789	-0.663258	-0.301408
Atr	1	0.436943	0.305255	-0.840392	0.098195

Trp53bp1	1	-0.65371	-0.314529	0.43417	0.534069
Ercc5	1	0.410289	0.581155	-0.530655	-0.460789
Fancm	1	0.436208	0.551392	-0.588702	-0.398898
Blm	1	0.686364	0.265013	-0.530973	-0.420405
Rad52	1	0.611748	0.359772	-0.375302	-0.596218
Ercc8	1	0.646867	0.308296	-0.354398	-0.600765
Trp53	1	0.556174	0.435642	-0.56313	-0.428686
Mgmt	1	0.426412	0.537839	-0.661028	-0.303223
Ercc2	1	-0.213914	0.86234	-0.338047	-0.310378
Rad9b	1	0.37308	0.561207	-0.701036	-0.233252
Recql5	1	0.614014	0.36719	-0.548821	-0.432382
Mlh1	1	0.631454	0.326908	-0.611786	-0.346577
Xrcc2	1	0.700806	0.204362	-0.622056	-0.283113
Recql	1	0.405595	0.540663	-0.254643	-0.691616
Ddb2	1	0.509688	0.469544	-0.347666	-0.631566
Tigar	1	0.448013	0.524587	-0.326431	-0.64617
Hic1	1	0.473818	0.525316	-0.485333	-0.513801
Eya2	1	0.49709	0.501741	-0.465306	-0.533524
Twist1	1	0.439754	0.49536	-0.218543	-0.716571
Chek1	1	0.614126	0.350665	-0.35111	-0.613682
Chek2	1	0.396332	0.477121	-0.094806	-0.778646
Parp1	1	0.175611	-0.857365	0.369962	0.311792

Epigenetic alterations

Genes	GWEIGHT	HCI_young	HCII_young	HCI_old	HCII_old
EWEIGHT		1	1	1	1
Ezh1	1	-0.32995	0.588395	0.376509	-0.634953
Hdac1	1	0.473793	0.524969	-0.523513	-0.475249
Hdac2	1	0.667095	0.292419	-0.412151	-0.547364
Hdac3	1	-0.289625	-0.671549	0.44051	0.520664
Hdac4	1	-0.575995	-0.414347	0.439685	0.550657
Hdac8	1	-0.285902	-0.652372	0.631142	0.307132
Hdac11	1	0.382887	0.579246	-0.315202	-0.646931
Smarca5	1	-0.61613	-0.368035	0.47415	0.510016
Smarcb1	1	-0.218706	0.832444	-0.495108	-0.118629
Setd1a	1	0.264431	0.543532	-0.796567	-0.011396
Setd1b	1	-0.450681	-0.544141	0.55201	0.442812
Setd2	1	-0.732158	-0.09053	0.653565	0.169123
Dnmt1	1	-0.553656	-0.441381	0.455101	0.539936

Dnmt3b	1	-0.288675	-0.288675	0.866025	-0.288675
Tet1	1	-0.265956	-0.627666	0.18602	0.707602
Tet2	1	-0.83681	0.13134	0.223012	0.482457
Tet3	1	0.676135	0.284558	-0.466184	-0.49451
Kdm5a	1	-0.425338	-0.568226	0.460574	0.53299
Kdm5b	1	-0.37601	-0.608093	0.444048	0.540056
Kdm4a	1	0.659695	0.288957	-0.349152	-0.599499
Kdm4b	1	0.257469	-0.859354	0.217017	0.384869
Kdm4c	1	0.458204	-0.841871	0.12978	0.253887
Kdm6a	1	-0.365296	-0.618476	0.483486	0.500286
Kdm6b	1	0.392701	0.596827	-0.494988	-0.49454
Dnmt3a	1	0.100974	-0.008539	0.65573	-0.748164
Chd1	1	-0.537295	-0.454268	0.414066	0.577497
Chd2	1	-0.838989	0.195427	0.162377	0.481186
Chd3	1	-0.014062	-0.753488	0.64597	0.12158
Chd4	1	-0.526881	-0.472373	0.501399	0.497855
Chd5	1	-0.210757	-0.041557	-0.552821	0.805136
Chd6	1	0.454581	0.537064	-0.577178	-0.414468
Chd7	1	-0.292589	-0.670221	0.452296	0.510513
Chd8	1	0.452982	0.540914	-0.432512	-0.561384
Chd9	1	-0.549155	-0.446062	0.543592	0.451625
Smarcc1	1	-0.634482	-0.33489	0.396819	0.572552
Smarcd1	1	-0.384402	-0.603473	0.486665	0.50121
Smarcd2	1	-0.190384	-0.729328	0.393144	0.526568
Baz1b	1	-0.236995	-0.689659	0.323935	0.602719
Baz2a	1	0.314244	0.650267	-0.400826	-0.563686
Phf2	1	0.394204	0.498772	-0.760436	-0.132539
Phf8	1	-0.204227	-0.723877	0.506513	0.42159
Hat1	1	-0.53298	-0.46563	0.515288	0.483322
Kat6a	1	-0.7524	-0.066411	0.626498	0.192313
Kat6b	1	0.507372	0.491769	-0.527813	-0.471329
Kat2b	1	-0.499795	-0.499795	0.479532	0.520057
Hp1bp3	1	0.201963	-0.86036	0.362732	0.295664
Rbbp4	1	-0.427836	-0.565318	0.450782	0.542372
Rbbp7	1	-0.499603	-0.499603	0.471433	0.527773
Mbd2	1	-0.591659	-0.398674	0.477579	0.512754
Mbd3	1	0.539787	0.458371	-0.485537	-0.512621
Ino80d	1	-0.849838	0.129301	0.333162	0.387375
Ino80b	1	-0.717357	-0.155955	0.23698	0.636331
Ino80c	1	0.044892	-0.787023	0.143909	0.598222

Ino80e	1	-0.238974	-0.704889	0.489004	0.454859
Ssrp1	1	0.316757	0.648968	-0.560943	-0.404782
Anp32e	1	-0.610588	-0.374404	0.46168	0.523312
Vps72	1	-0.69466	-0.253282	0.51869	0.429252
Mllt3	1	-0.559591	-0.434291	0.543572	0.45031
Itgb3bp	1	0.563354	0.426435	-0.420922	-0.568867
Scmh1	1	-0.209211	-0.714063	0.561955	0.361319
Znhit1	1	-0.696445	0.610352	-0.220324	0.306418
Spty2d1	1	0.408778	0.582403	-0.530865	-0.460315
Ctr9	1	-0.821687	0.210916	0.521977	0.088794
Kcnq1ot1	1	-0.68665	-0.244516	0.598228	0.332937
Samd1	1	0.5921	0.38205	-0.366719	-0.607431
Ctcf	1	-0.401119	-0.579473	0.384148	0.596444
Ppm1d	1	-0.239375	-0.702993	0.518471	0.423897
Mphosph8	1	-0.62795	-0.339784	0.58868	0.379053
Setdb2	1	0.485786	0.511164	-0.444767	-0.552183
L3mbtl3	1	0.630646	0.348706	-0.513394	-0.465958
Znfx1	1	-0.143894	-0.690639	0.139698	0.694835
Cenpv	1	-0.427219	-0.567714	0.486573	0.508361
Hmgb1	1	-0.765791	-0.087983	0.283088	0.570687
Apobec1	1	0.477642	0.485048	-0.290032	-0.672658

Intercellular communication

Genes	GWEIGHT	HCI_young	HCII_young	HCI_old	HCII_old
EWEIGHT		1	1	1	1
Gja1	1	0.563823	0.422844	-0.402364	-0.584303
Fat4	1	0.500311	0.499621	-0.508212	-0.49172
Ctnna2	1	0.510461	0.463049	-0.646684	-0.326826
Itgb2	1	0.500557	0.490515	-0.58968	-0.401392
Ncam1	1	0.483121	0.493844	-0.337681	-0.639284
Cldn23	1	0.502449	0.48648	-0.389843	-0.599086
Dscaml1	1	0.674571	0.28467	-0.523465	-0.435776
Cdh5	1	0.49524	0.497516	-0.411425	-0.58133
Cdh3	1	0.502285	0.49771	-0.499997	-0.499997
Gjb2	1	0.517771	0.424364	-0.238669	-0.703466
Cdh18	1	0.44352	0.498011	-0.234084	-0.707447
Tjp3	1	0.589832	0.371672	-0.64155	-0.319954

Itgb8	1	0.50964	0.489967	-0.517015	-0.482592
Jam3	1	0.500416	0.498318	-0.534916	-0.463818
Cdh1	1	0.418649	0.555215	-0.6323	-0.341564
Pcdh17	1	0.479406	0.510592	-0.59353	-0.396468
Cd300a	1	0.465643	0.524222	-0.398882	-0.590983
Pcdh9	1	0.403672	0.522353	-0.202786	-0.723239
Itgav	1	0.596767	0.390503	-0.448774	-0.538496
Cd44	1	0.400903	0.390659	-0.827856	0.036293
Itga8	1	0.488102	0.501269	-0.597296	-0.392075
Itga9	1	0.511875	0.469732	-0.357463	-0.624144
Itgb1	1	0.029982	0.316427	-0.820883	0.474474
Ptprf	1	0.478805	-0.678905	-0.280617	0.480717
Cdh15	1	0.245977	0.596943	-0.083897	-0.759023
Ptprg	1	0.31623	0.638858	-0.343824	-0.611264
Cdh11	1	0.326944	0.270801	-0.865055	0.267309
Nlgn2	1	0.391424	0.456965	-0.051309	-0.79708
Ptprk	1	0.453442	0.542824	-0.539724	-0.456542
Pkp1	1	0.445071	0.539972	-0.380301	-0.604742
Pcdhgb5	1	0.470218	0.500158	-0.315007	-0.655369
Itga4	1	0.421891	0.460508	-0.773334	-0.109066
Cdh24	1	0.429301	0.45418	-0.772763	-0.110717
Cd151	1	0.472223	0.409144	-0.773251	-0.108116
Cldn10	1	0.543552	0.41322	-0.673443	-0.283329
Pecam1	1	0.371704	0.587596	-0.311662	-0.647638
Dsc2	1	0.440759	0.55592	-0.498339	-0.498339
Pcdhga10	1	0.44976	0.539962	-0.585363	-0.404359
Pcdhga4	1	0.46389	0.519322	-0.365592	-0.61762
Nlgn1	1	0.363654	0.579258	-0.262082	-0.68083
Dsp	1	0.178007	0.724495	-0.585722	-0.316781
Pkp3	1	0.25833	0.584825	-0.764936	-0.07822
Pcdhgc4	1	0.144639	0.727248	-0.248731	-0.623156
Cd276	1	-0.06521	0.734062	-0.675907	0.007055

Figure 4 source data

4A

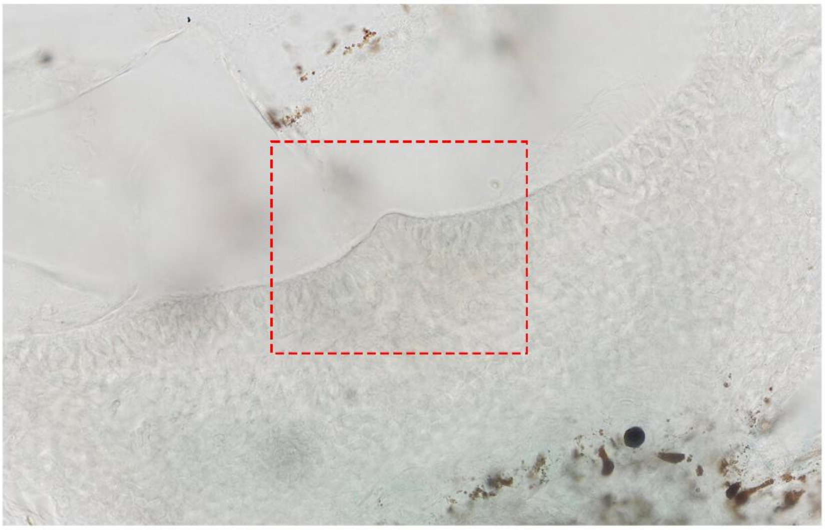

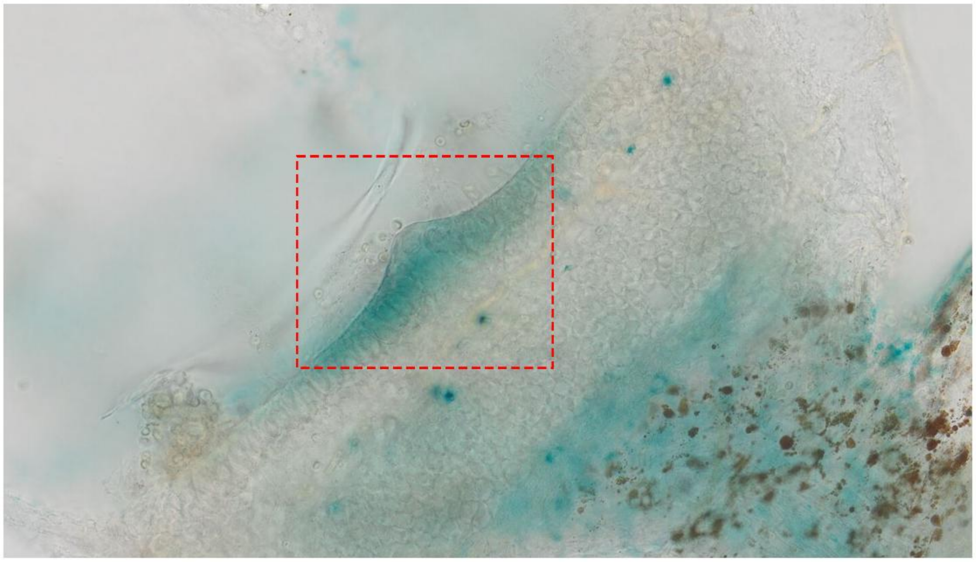

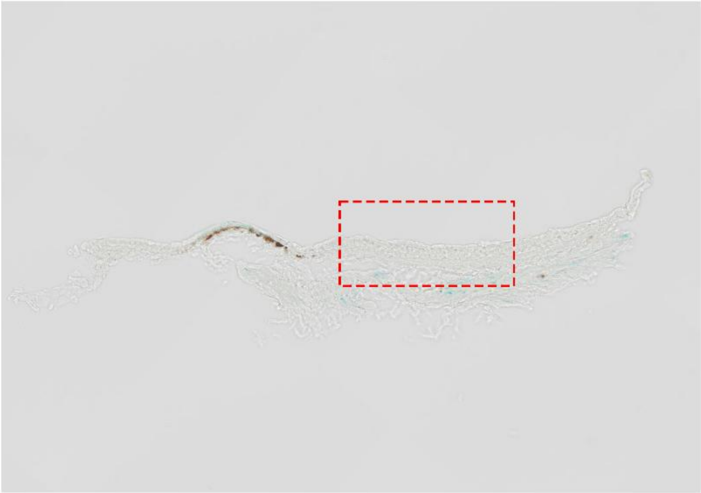

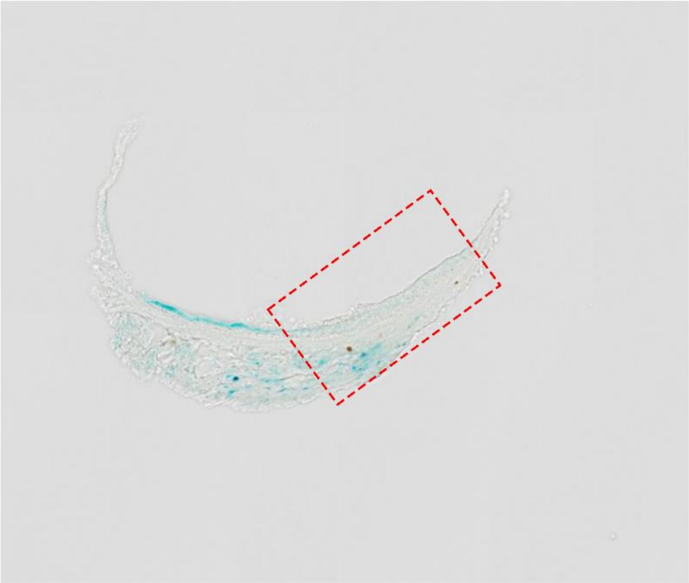

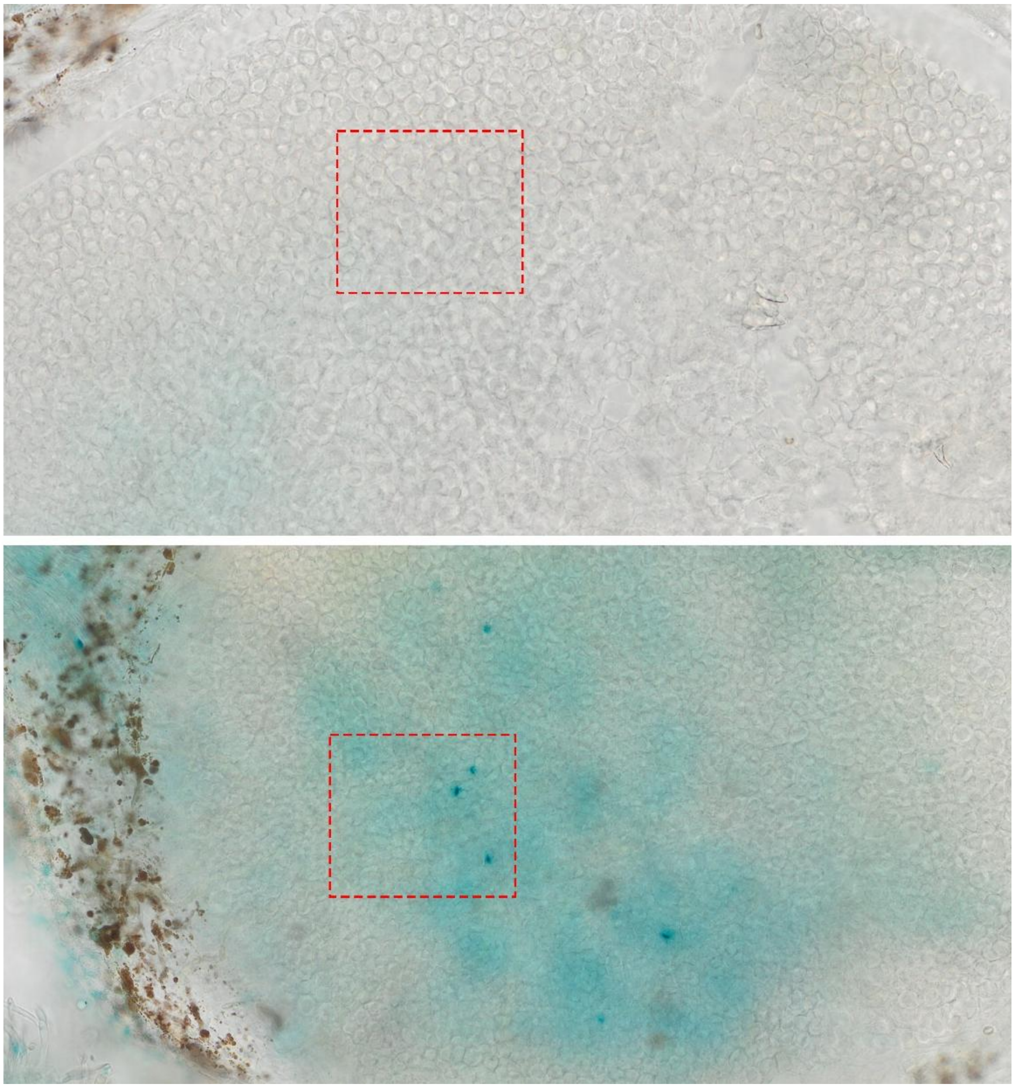

4B

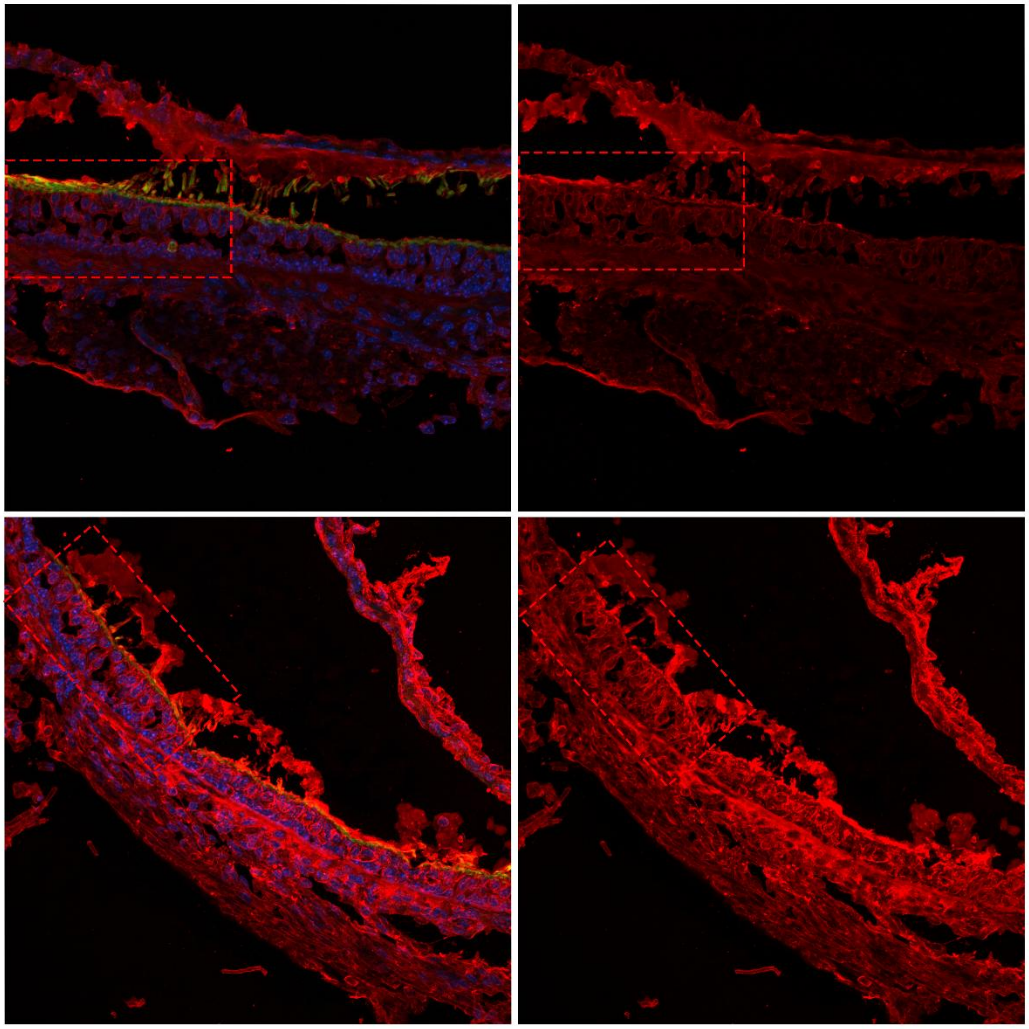

4C

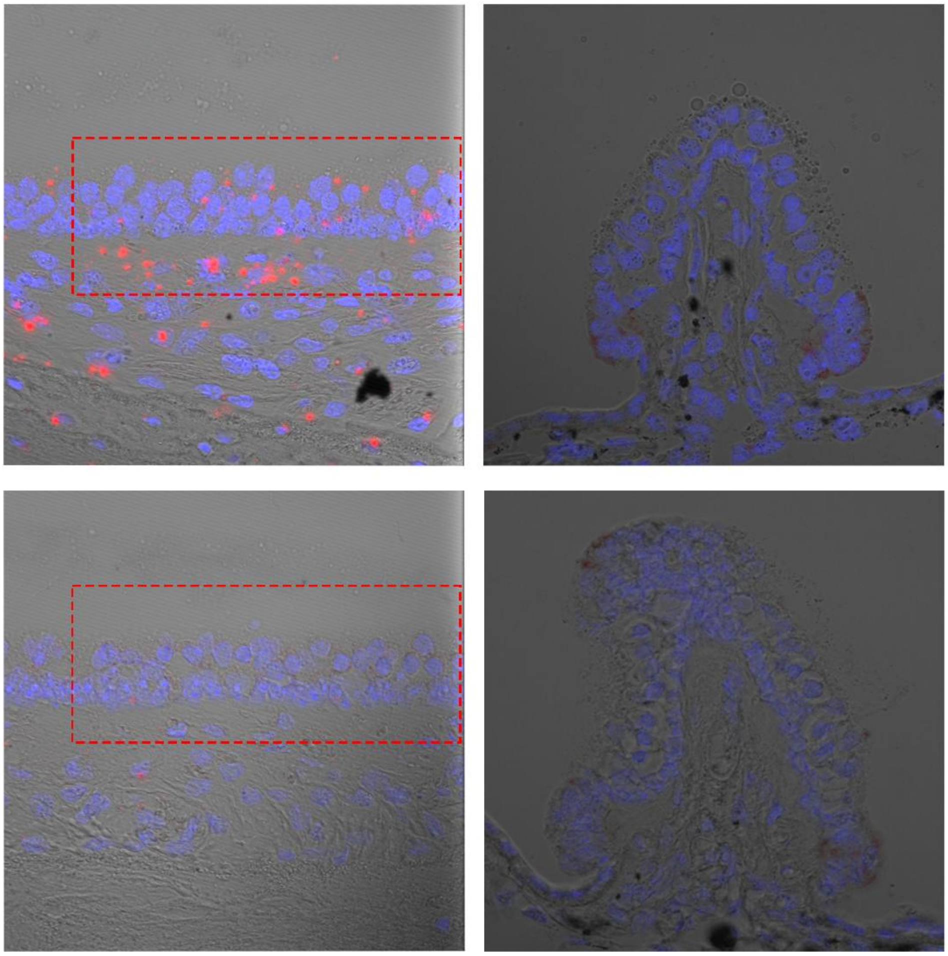

4D

	Otoliths	Crista
Young	Puncta signal density	Puncta signal density
#1	0.05559177	0.052121763
#2	0.05845874	0.107174652
#3	0.02726436	0.08166559
Old		
#1	0.01734852	0.048756586
#2	0.01719048	0.027460463
#3	0.01374565	0.017205879

4E

	Young	Old
	Mean Intensity	Mean Intensity

#1	67.332	113.156
#2	79.154	94.061
#3	36.229	116.175

Figure 5 source data

gene	p_val	p_val_adj	comparison
Cib2	2.04E-07	0.00354984	Young_vs_Old
Fbxo2	3.65E-40	6.34E-36	Young_vs_Old
Nek2	5.82E-13	1.01E-08	Young_vs_Old
Atxn7l3b	3.90E-29	6.78E-25	Young_vs_Old
Lrrc10b	1.88E-24	3.26E-20	Young_vs_Old
Slc17a8	3.33E-11	5.78E-07	Young_vs_Old
Tuba1a	8.90E-19	1.55E-14	Young_vs_Old
1700001C02Rik	2.08E-12	3.61E-08	Young_vs_Old
Lrrc73	6.92E-12	1.20E-07	Young_vs_Old
Cfap126	2.44E-20	4.23E-16	Young_vs_Old
Slc25a4	1.05E-22	1.82E-18	Young_vs_Old
Kncn	1.24E-16	2.15E-12	Young_vs_Old
Rsph1	1.05E-17	1.83E-13	Young_vs_Old
Espn	4.04E-40	7.01E-36	Young_vs_Old
Slc9a3r2	2.64E-11	4.58E-07	Young_vs_Old
Ttc21a	1.78E-15	3.09E-11	Young_vs_Old
Kif21a	4.24E-12	7.37E-08	Young_vs_Old
Arf5	1.58E-09	2.75E-05	Young_vs_Old
Purb	3.21E-17	5.57E-13	Young_vs_Old
1700024G13Rik	3.14E-11	5.45E-07	Young_vs_Old
Prdx5	2.31E-10	4.01E-06	Young_vs_Old
Jund	9.56E-12	1.66E-07	Young_vs_Old
Cst6	1.13E-06	0.01967025	Young_vs_Old
Tbc1d7	1.07E-07	0.00186618	Young_vs_Old
Pvalb	1.04E-06	0.01798989	Young_vs_Old
Tle5	8.22E-07	0.01427874	Young_vs_Old
Wdr66	0.00020758	1	Young_vs_Old
Negr1	1.17E-08	0.00020282	Young_vs_Old
Lsm4	2.33E-10	4.05E-06	Young_vs_Old
Pmvk	1.17E-08	0.00020358	Young_vs_Old
2410004P03Rik	5.62E-08	0.00097672	Young_vs_Old
Flywch2	1.27E-09	2.21E-05	Young_vs_Old

Ush1c	2.54E-09	4.41E-05	Young_vs_Old
1700016K19Rik	5.01E-10	8.70E-06	Young_vs_Old
Myl6	5.52E-13	9.59E-09	Young_vs_Old
Otud3	1.41E-08	0.00024466	Young_vs_Old
Nedd8	8.68E-12	1.51E-07	Young_vs_Old
Dynll2	1.61E-12	2.80E-08	Young_vs_Old
Rab2a	1.41E-08	0.00024523	Young_vs_Old
1110017D15Rik	3.93E-14	6.83E-10	Young_vs_Old
Gm30191	5.01E-08	0.00087096	Young_vs_Old
Tubb4b	4.14E-10	7.19E-06	Young_vs_Old
Elof1	9.73E-11	1.69E-06	Young_vs_Old
Morn2	1.23E-09	2.14E-05	Young_vs_Old
Homer2	9.62E-06	0.16708527	Young_vs_Old
Ap2b1	2.33E-08	0.000404	Young_vs_Old
Actb	4.35E-08	0.00075626	Young_vs_Old
Uaca	1.73E-09	3.00E-05	Young_vs_Old
Wbp1	2.46E-09	4.28E-05	Young_vs_Old
Ccdc30	2.76E-10	4.79E-06	Young_vs_Old
Ttll3	1.63E-10	2.84E-06	Young_vs_Old
Gsn	9.86E-09	0.00017134	Young_vs_Old
Zfp106	2.81E-09	4.89E-05	Young_vs_Old
Edf1	1.75E-11	3.04E-07	Young_vs_Old
Calm1	5.51E-26	9.56E-22	Young_vs_Old
Rgs9	6.69E-06	0.11626395	Young_vs_Old
Tekt2	8.47E-07	0.01470773	Young_vs_Old
1110008P14Rik	1.64E-06	0.02842818	Young_vs_Old
Dctn3	2.69E-07	0.00466486	Young_vs_Old
Llph	1.70E-06	0.02959716	Young_vs_Old
Hsp90ab1	6.00E-20	1.04E-15	Young_vs_Old
Lmod1	5.43E-08	0.00094345	Young_vs_Old
Lmo1	2.73E-06	0.04735546	Young_vs_Old
Erp29	8.83E-08	0.00153425	Young_vs_Old
Ccdc81	0.00152157	1	Young_vs_Old
Anp32b	3.28E-05	0.57053073	Young_vs_Old
Rbm24	4.92E-07	0.00854599	Young_vs_Old
Serf1	8.16E-11	1.42E-06	Young_vs_Old
Chrac1	6.80E-09	0.00011818	Young_vs_Old
Spop	3.29E-07	0.00572276	Young_vs_Old
Nudc	7.13E-11	1.24E-06	Young_vs_Old
Mrpl54	6.83E-06	0.11872555	Young_vs_Old

Drc7	8.42E-06	0.1462018	Young_vs_Old
Pou4f3	5.26E-10	9.14E-06	Young_vs_Old
Sfr1	1.72E-08	0.00029845	Young_vs_Old
Cfap46	0.00026167	1	Young_vs_Old
Evl	5.49E-08	0.00095439	Young_vs_Old
Vgll4	7.81E-09	0.00013573	Young_vs_Old
Lrrc51	1.47E-06	0.02548771	Young_vs_Old
Pdcd4	2.89E-06	0.0501664	Young_vs_Old
Gtf2i	1.68E-06	0.02913358	Young_vs_Old
Mns1	1.91E-07	0.00331197	Young_vs_Old
Ccdc85b	3.38E-07	0.00587012	Young_vs_Old
Stard10	9.90E-05	1	Young_vs_Old
1700094D03Rik	9.52E-07	0.01653466	Young_vs_Old
Madd	6.40E-05	1	Young_vs_Old
Saraf	1.74E-05	0.30170226	Young_vs_Old
Map4k4	3.47E-07	0.00602259	Young_vs_Old
Bex3	5.22E-05	0.90603619	Young_vs_Old
Fam104a	4.42E-06	0.07669784	Young_vs_Old
Atp5d	7.64E-08	0.00132664	Young_vs_Old
Tex52	1.54E-05	0.26698051	Young_vs_Old
Rab14	1.31E-06	0.02276611	Young_vs_Old
Cct7	1.34E-06	0.02319268	Young_vs_Old
Smap2	3.11E-05	0.54034015	Young_vs_Old
Otof	3.58E-05	0.62186813	Young_vs_Old
Pifo	1.56E-07	0.00270549	Young_vs_Old
Calml4	2.79E-13	4.85E-09	Young_vs_Old
Znhit1	4.22E-07	0.00733101	Young_vs_Old
Arhgdig	6.68E-05	1	Young_vs_Old
Knop1	3.88E-05	0.67438267	Young_vs_Old
Slco3a1	1.93E-05	0.33449045	Young_vs_Old
Bbof1	0.00020105	1	Young_vs_Old
Kifap3	6.43E-06	0.11166172	Young_vs_Old
Smdt1	1.66E-08	0.00028914	Young_vs_Old
Pgls	7.06E-05	1	Young_vs_Old
Usf2	1.94E-05	0.33727034	Young_vs_Old
Gnas	1.11E-07	0.00193143	Young_vs_Old
Dnajb2	2.66E-05	0.46285807	Young_vs_Old
St3gal1	0.0003092	1	Young_vs_Old
Mrpl18	1.95E-06	0.03392164	Young_vs_Old
Hyou1	1.43E-05	0.24782637	Young_vs_Old

Kcp	2.24E-06	0.03891115	Young_vs_Old
Hydin	0.00026772	1	Young_vs_Old
Supt6	6.18E-06	0.10736582	Young_vs_Old
Hebp2	1.66E-06	0.02882741	Young_vs_Old
Mtus2	9.50E-06	0.16506589	Young_vs_Old
Lamtor4	4.60E-06	0.07992785	Young_vs_Old
Auts2	5.08E-05	0.88324801	Young_vs_Old
Ndufb9	2.48E-06	0.04309852	Young_vs_Old
1110032A03Rik	1.38E-05	0.23896199	Young_vs_Old
Fads2	2.40E-05	0.4176057	Young_vs_Old
Ube2k	0.00012665	1	Young_vs_Old
Map1lc3a	2.39E-06	0.04159129	Young_vs_Old
Ccdc181	0.00033102	1	Young_vs_Old
St13	2.50E-06	0.04338064	Young_vs_Old
Nf2	0.00085389	1	Young_vs_Old
Ssu72	0.00026608	1	Young_vs_Old
Ccdc40	2.85E-06	0.04955181	Young_vs_Old
Sys1	4.33E-06	0.0752636	Young_vs_Old
4833439L19Rik	7.01E-06	0.12169258	Young_vs_Old
Cabp2	1.84E-11	3.20E-07	Young_vs_Old
Rundc3a	3.30E-05	0.5738734	Young_vs_Old
Dap	8.70E-06	0.15112689	Young_vs_Old
Nfic	2.92E-05	0.5067483	Young_vs_Old
Car7	0.0014065	1	Young_vs_Old
Dnajb11	5.09E-08	0.0008844	Young_vs_Old
Pebp1	4.13E-08	0.0007176	Young_vs_Old
Stk39	0.00018204	1	Young_vs_Old
2300009A05Rik	1.30E-05	0.22511773	Young_vs_Old
Cdkn2d	7.32E-05	1	Young_vs_Old
Ctxn1	0.00561083	1	Young_vs_Old
0610012G03Rik	0.00018013	1	Young_vs_Old
Casc4	1.77E-05	0.30708462	Young_vs_Old
Ccdc114	0.00255272	1	Young_vs_Old
Chd7	0.00335931	1	Young_vs_Old
Zdhhc16	0.00788769	1	Young_vs_Old
Ik	2.09E-05	0.36268245	Young_vs_Old
Mlf2	0.00192915	1	Young_vs_Old
Gadd45g	0.0517959	1	Young_vs_Old
Calm2	1.02E-17	1.77E-13	Young_vs_Old
Zfp664	0.00019341	1	Young_vs_Old

Pitpna	1.37E-05	0.23768913	Young_vs_Old
Hras	0.00010212	1	Young_vs_Old
Lpgat1	1.48E-07	0.00256811	Young_vs_Old
Etfb	2.65E-05	0.45984258	Young_vs_Old
Spp1	5.76E-10	1.00E-05	Young_vs_Old
Lhx3	0.00014462	1	Young_vs_Old
Smpx	1.48E-06	0.02569696	Young_vs_Old
Serinc1	3.91E-05	0.67863112	Young_vs_Old
Canx	0.00043663	1	Young_vs_Old
Gls	0.00045333	1	Young_vs_Old
Ebf1	5.20E-05	0.90314886	Young_vs_Old
2310009B15Rik	5.40E-06	0.09375979	Young_vs_Old
Pcbp2	3.57E-05	0.61995153	Young_vs_Old
Rufy3	2.32E-06	0.04029047	Young_vs_Old
Tusc3	8.73E-05	1	Young_vs_Old
Rpl36	1.19E-09	2.06E-05	Young_vs_Old
Etnk1	7.10E-06	0.12339521	Young_vs_Old
Rsph4a	0.00029461	1	Young_vs_Old
Laptm4a	0.00268093	1	Young_vs_Old
Trim36	5.82E-05	1	Young_vs_Old
Pde5a	0.00028374	1	Young_vs_Old
Rpl21	3.27E-05	0.56722119	Young_vs_Old
Rpl17	0.00026548	1	Young_vs_Old
Gpr155	4.57E-05	0.7943551	Young_vs_Old
Tspan12	2.64E-06	0.04583079	Young_vs_Old
Rpl36a	0.00125529	1	Young_vs_Old
Rpl37a	5.02E-09	8.71E-05	Young_vs_Old
1700012P22Rik	3.94E-06	0.06839662	Young_vs_Old
Nptn	3.53E-06	0.06124399	Young_vs_Old
Rpl30	1.46E-09	2.54E-05	Young_vs_Old
Cab39l	2.57E-05	0.44603844	Young_vs_Old
Elfn1	0.00083229	1	Young_vs_Old
Arl6	4.52E-06	0.07859894	Young_vs_Old
Hnrnph1	3.43E-06	0.05950388	Young_vs_Old
Gm47283	0.00024207	1	Young_vs_Old
Ano3	7.55E-06	0.13115912	Young_vs_Old
Rpl10	0.00046591	1	Young_vs_Old
Gm32742	0.03075987	1	Young_vs_Old
Cyp2g1	1.77E-08	0.0003069	Young_vs_Old
Rps27	1.98E-05	0.34368281	Young_vs_Old

Srebf1	3.06E-08	0.00053094	Young_vs_Old
Rps24	2.00E-11	3.47E-07	Young_vs_Old
Lars2	0.07506698	1	Young_vs_Old
Ptn	0.00110874	1	Young_vs_Old
Mindy4b-ps	2.61E-06	0.0453716	Young_vs_Old
Rpl37	8.77E-11	1.52E-06	Young_vs_Old
Phip	1.82E-07	0.00315744	Young_vs_Old
mt-Atp6	9.05E-08	0.0015717	Young_vs_Old
Gas5	3.35E-06	0.05818892	Young_vs_Old
Gm10260	2.65E-07	0.00460728	Young_vs_Old
Rpl22l1	1.40E-09	2.43E-05	Young_vs_Old
mt-Nd5	7.19E-07	0.01249843	Young_vs_Old
mt-Co2	1.05E-12	1.83E-08	Young_vs_Old
Rps15a	3.93E-14	6.82E-10	Young_vs_Old
Rpl39	6.38E-13	1.11E-08	Young_vs_Old
Rps2	2.88E-17	5.00E-13	Young_vs_Old
mt-Cytb	4.92E-19	8.54E-15	Young_vs_Old
Plat	3.10E-08	0.00053907	Young_vs_Old
Txnip	1.51E-08	0.00026218	Young_vs_Old
Rps3a1	1.16E-19	2.02E-15	Young_vs_Old
Gm4791	1.21E-08	0.00020996	Young_vs_Old
Apoe	1.57E-07	0.00272827	Young_vs_Old
Rpl35	6.44E-28	1.12E-23	Young_vs_Old
mt-Nd3	1.32E-16	2.30E-12	Young_vs_Old
mt-Co1	2.06E-28	3.58E-24	Young_vs_Old
mt-Co3	1.96E-32	3.40E-28	Young_vs_Old
mt-Nd4l	1.70E-27	2.96E-23	Young_vs_Old
S100a13	0.12256792	1	Young_vs_Old
S100b	1.06E-14	1.83E-10	Young_vs_Old
Uba52	2.86E-26	4.97E-22	Young_vs_Old
Atp1a2	1.16E-12	2.01E-08	Young_vs_Old
Coch	2.47E-24	4.29E-20	Young_vs_Old
Mt1	1.38E-53	2.40E-49	Young_vs_Old
Apod	5.93E-24	1.03E-19	Young_vs_Old
Otos	8.82E-18	1.53E-13	Young_vs_Old
Ptgds	8.24E-49	1.43E-44	Young_vs_Old
Ptgds	8.24E-49	1.43E-44	Old_vs_Young
Otos	8.82E-18	1.53E-13	Old_vs_Young
Apod	5.93E-24	1.03E-19	Old_vs_Young
Mt1	1.38E-53	2.40E-49	Old_vs_Young

Coch	2.47E-24	4.29E-20	Old_vs_Young
Atp1a2	1.16E-12	2.01E-08	Old_vs_Young
Uba52	2.86E-26	4.97E-22	Old_vs_Young
S100b	1.06E-14	1.83E-10	Old_vs_Young
S100a13	0.12256792	1	Old_vs_Young
mt-Nd4l	1.70E-27	2.96E-23	Old_vs_Young
mt-Co3	1.96E-32	3.40E-28	Old_vs_Young
mt-Co1	2.06E-28	3.58E-24	Old_vs_Young
mt-Nd3	1.32E-16	2.30E-12	Old_vs_Young
Rpl35	6.44E-28	1.12E-23	Old_vs_Young
Apoe	1.57E-07	0.00272827	Old_vs_Young
Gm4791	1.21E-08	0.00020996	Old_vs_Young
Rps3a1	1.16E-19	2.02E-15	Old_vs_Young
Txnip	1.51E-08	0.00026218	Old_vs_Young
Plat	3.10E-08	0.00053907	Old_vs_Young
mt-Cytb	4.92E-19	8.54E-15	Old_vs_Young
Rps2	2.88E-17	5.00E-13	Old_vs_Young
Rpl39	6.38E-13	1.11E-08	Old_vs_Young
Rps15a	3.93E-14	6.82E-10	Old_vs_Young
mt-Co2	1.05E-12	1.83E-08	Old_vs_Young
mt-Nd5	7.19E-07	0.01249843	Old_vs_Young
Rpl22l1	1.40E-09	2.43E-05	Old_vs_Young
Gm10260	2.65E-07	0.00460728	Old_vs_Young
Gas5	3.35E-06	0.05818892	Old_vs_Young
mt-Atp6	9.05E-08	0.0015717	Old_vs_Young
Phip	1.82E-07	0.00315744	Old_vs_Young
Rpl37	8.77E-11	1.52E-06	Old_vs_Young
Mindy4b-ps	2.61E-06	0.0453716	Old_vs_Young
Ptn	0.00110874	1	Old_vs_Young
Lars2	0.07506698	1	Old_vs_Young
Rps24	2.00E-11	3.47E-07	Old_vs_Young
Srebf1	3.06E-08	0.00053094	Old_vs_Young
Rps27	1.98E-05	0.34368281	Old_vs_Young
Cyp2g1	1.77E-08	0.0003069	Old_vs_Young
Gm32742	0.03075987	1	Old_vs_Young
Rpl10	0.00046591	1	Old_vs_Young
Ano3	7.55E-06	0.13115912	Old_vs_Young
Gm47283	0.00024207	1	Old_vs_Young
Hnrnph1	3.43E-06	0.05950388	Old_vs_Young
Arl6	4.52E-06	0.07859894	Old_vs_Young

Elfn1	0.00083229	1	Old_vs_Young
Cab39l	2.57E-05	0.44603844	Old_vs_Young
Rpl30	1.46E-09	2.54E-05	Old_vs_Young
Nptn	3.53E-06	0.06124399	Old_vs_Young
1700012P22Rik	3.94E-06	0.06839662	Old_vs_Young
Rpl37a	5.02E-09	8.71E-05	Old_vs_Young
Rpl36a	0.00125529	1	Old_vs_Young
Tspan12	2.64E-06	0.04583079	Old_vs_Young
Gpr155	4.57E-05	0.7943551	Old_vs_Young
Rpl17	0.00026548	1	Old_vs_Young
Rpl21	3.27E-05	0.56722119	Old_vs_Young
Pde5a	0.00028374	1	Old_vs_Young
Trim36	5.82E-05	1	Old_vs_Young
Laptm4a	0.00268093	1	Old_vs_Young
Rsph4a	0.00029461	1	Old_vs_Young
Etnk1	7.10E-06	0.12339521	Old_vs_Young
Rpl36	1.19E-09	2.06E-05	Old_vs_Young
Tusc3	8.73E-05	1	Old_vs_Young
Rufy3	2.32E-06	0.04029047	Old_vs_Young
Pcbp2	3.57E-05	0.61995153	Old_vs_Young
2310009B15Rik	5.40E-06	0.09375979	Old_vs_Young
Ebf1	5.20E-05	0.90314886	Old_vs_Young
Gls	0.00045333	1	Old_vs_Young
Canx	0.00043663	1	Old_vs_Young
Serinc1	3.91E-05	0.67863112	Old_vs_Young
Smpx	1.48E-06	0.02569696	Old_vs_Young
Lhx3	0.00014462	1	Old_vs_Young
Spp1	5.76E-10	1.00E-05	Old_vs_Young
Etfb	2.65E-05	0.45984258	Old_vs_Young
Lpgat1	1.48E-07	0.00256811	Old_vs_Young
Hras	0.00010212	1	Old_vs_Young
Pitpna	1.37E-05	0.23768913	Old_vs_Young
Zfp664	0.00019341	1	Old_vs_Young
Calm2	1.02E-17	1.77E-13	Old_vs_Young
Gadd45g	0.0517959	1	Old_vs_Young
Mlf2	0.00192915	1	Old_vs_Young
Ik	2.09E-05	0.36268245	Old_vs_Young
Zdhhc16	0.00788769	1	Old_vs_Young
Chd7	0.00335931	1	Old_vs_Young
Ccdc114	0.00255272	1	Old_vs_Young

Casc4	1.77E-05	0.30708462	Old_vs_Young
0610012G03Rik	0.00018013	1	Old_vs_Young
Ctxn1	0.00561083	1	Old_vs_Young
Cdkn2d	7.32E-05	1	Old_vs_Young
2300009A05Rik	1.30E-05	0.22511773	Old_vs_Young
Stk39	0.00018204	1	Old_vs_Young
Pebp1	4.13E-08	0.0007176	Old_vs_Young
Dnajb11	5.09E-08	0.0008844	Old_vs_Young
Car7	0.0014065	1	Old_vs_Young
Nfic	2.92E-05	0.5067483	Old_vs_Young
Dap	8.70E-06	0.15112689	Old_vs_Young
Rundc3a	3.30E-05	0.5738734	Old_vs_Young
Cabp2	1.84E-11	3.20E-07	Old_vs_Young
4833439L19Rik	7.01E-06	0.12169258	Old_vs_Young
Sys1	4.33E-06	0.0752636	Old_vs_Young
Ccdc40	2.85E-06	0.04955181	Old_vs_Young
Ssu72	0.00026608	1	Old_vs_Young
Nf2	0.00085389	1	Old_vs_Young
St13	2.50E-06	0.04338064	Old_vs_Young
Ccdc181	0.00033102	1	Old_vs_Young
Map1lc3a	2.39E-06	0.04159129	Old_vs_Young
Ube2k	0.00012665	1	Old_vs_Young
Fads2	2.40E-05	0.4176057	Old_vs_Young
1110032A03Rik	1.38E-05	0.23896199	Old_vs_Young
Ndufb9	2.48E-06	0.04309852	Old_vs_Young
Auts2	5.08E-05	0.88324801	Old_vs_Young
Lamtor4	4.60E-06	0.07992785	Old_vs_Young
Mtus2	9.50E-06	0.16506589	Old_vs_Young
Hebp2	1.66E-06	0.02882741	Old_vs_Young
Supt6	6.18E-06	0.10736582	Old_vs_Young
Hydin	0.00026772	1	Old_vs_Young
Kcp	2.24E-06	0.03891115	Old_vs_Young
Hyou1	1.43E-05	0.24782637	Old_vs_Young
Mrpl18	1.95E-06	0.03392164	Old_vs_Young
St3gal1	0.0003092	1	Old_vs_Young
Dnajb2	2.66E-05	0.46285807	Old_vs_Young
Gnas	1.11E-07	0.00193143	Old_vs_Young
Usf2	1.94E-05	0.33727034	Old_vs_Young
Pgls	7.06E-05	1	Old_vs_Young
Smdt1	1.66E-08	0.00028914	Old_vs_Young

Kifap3	6.43E-06	0.11166172	Old_vs_Young
Bbof1	0.00020105	1	Old_vs_Young
Slco3a1	1.93E-05	0.33449045	Old_vs_Young
Knop1	3.88E-05	0.67438267	Old_vs_Young
Arhgdig	6.68E-05	1	Old_vs_Young
Znhit1	4.22E-07	0.00733101	Old_vs_Young
Calml4	2.79E-13	4.85E-09	Old_vs_Young
Pifo	1.56E-07	0.00270549	Old_vs_Young
Otof	3.58E-05	0.62186813	Old_vs_Young
Smap2	3.11E-05	0.54034015	Old_vs_Young
Cct7	1.34E-06	0.02319268	Old_vs_Young
Rab14	1.31E-06	0.02276611	Old_vs_Young
Tex52	1.54E-05	0.26698051	Old_vs_Young
Atp5d	7.64E-08	0.00132664	Old_vs_Young
Fam104a	4.42E-06	0.07669784	Old_vs_Young
Bex3	5.22E-05	0.90603619	Old_vs_Young
Map4k4	3.47E-07	0.00602259	Old_vs_Young
Saraf	1.74E-05	0.30170226	Old_vs_Young
Madd	6.40E-05	1	Old_vs_Young
1700094D03Rik	9.52E-07	0.01653466	Old_vs_Young
Stard10	9.90E-05	1	Old_vs_Young
Ccdc85b	3.38E-07	0.00587012	Old_vs_Young
Mns1	1.91E-07	0.00331197	Old_vs_Young
Gtf2i	1.68E-06	0.02913358	Old_vs_Young
Pdcd4	2.89E-06	0.0501664	Old_vs_Young
Lrrc51	1.47E-06	0.02548771	Old_vs_Young
Vgll4	7.81E-09	0.00013573	Old_vs_Young
Evl	5.49E-08	0.00095439	Old_vs_Young
Cfap46	0.00026167	1	Old_vs_Young
Sfr1	1.72E-08	0.00029845	Old_vs_Young
Pou4f3	5.26E-10	9.14E-06	Old_vs_Young
Drc7	8.42E-06	0.1462018	Old_vs_Young
Mrpl54	6.83E-06	0.11872555	Old_vs_Young
Nudc	7.13E-11	1.24E-06	Old_vs_Young
Spop	3.29E-07	0.00572276	Old_vs_Young
Chrac1	6.80E-09	0.00011818	Old_vs_Young
Serf1	8.16E-11	1.42E-06	Old_vs_Young
Rbm24	4.92E-07	0.00854599	Old_vs_Young
Anp32b	3.28E-05	0.57053073	Old_vs_Young
Ccdc81	0.00152157	1	Old_vs_Young

Erp29	8.83E-08	0.00153425	Old_vs_Young
Lmo1	2.73E-06	0.04735546	Old_vs_Young
Lmod1	5.43E-08	0.00094345	Old_vs_Young
Hsp90ab1	6.00E-20	1.04E-15	Old_vs_Young
Llph	1.70E-06	0.02959716	Old_vs_Young
Dctn3	2.69E-07	0.00466486	Old_vs_Young
1110008P14Rik	1.64E-06	0.02842818	Old_vs_Young
Tekt2	8.47E-07	0.01470773	Old_vs_Young
Rgs9	6.69E-06	0.11626395	Old_vs_Young
Calm1	5.51E-26	9.56E-22	Old_vs_Young
Edf1	1.75E-11	3.04E-07	Old_vs_Young
Zfp106	2.81E-09	4.89E-05	Old_vs_Young
Gsn	9.86E-09	0.00017134	Old_vs_Young
Ttll3	1.63E-10	2.84E-06	Old_vs_Young
Ccdc30	2.76E-10	4.79E-06	Old_vs_Young
Wbp1	2.46E-09	4.28E-05	Old_vs_Young
Uaca	1.73E-09	3.00E-05	Old_vs_Young
Actb	4.35E-08	0.00075626	Old_vs_Young
Ap2b1	2.33E-08	0.000404	Old_vs_Young
Homer2	9.62E-06	0.16708527	Old_vs_Young
Morn2	1.23E-09	2.14E-05	Old_vs_Young
Elof1	9.73E-11	1.69E-06	Old_vs_Young
Tubb4b	4.14E-10	7.19E-06	Old_vs_Young
Gm30191	5.01E-08	0.00087096	Old_vs_Young
1110017D15Rik	3.93E-14	6.83E-10	Old_vs_Young
Rab2a	1.41E-08	0.00024523	Old_vs_Young
Dynll2	1.61E-12	2.80E-08	Old_vs_Young
Nedd8	8.68E-12	1.51E-07	Old_vs_Young
Otud3	1.41E-08	0.00024466	Old_vs_Young
Myl6	5.52E-13	9.59E-09	Old_vs_Young
1700016K19Rik	5.01E-10	8.70E-06	Old_vs_Young
Ush1c	2.54E-09	4.41E-05	Old_vs_Young
Flywch2	1.27E-09	2.21E-05	Old_vs_Young
2410004P03Rik	5.62E-08	0.00097672	Old_vs_Young
Pmvk	1.17E-08	0.00020358	Old_vs_Young
Lsm4	2.33E-10	4.05E-06	Old_vs_Young
Negr1	1.17E-08	0.00020282	Old_vs_Young
Wdr66	0.00020758	1	Old_vs_Young
Tle5	8.22E-07	0.01427874	Old_vs_Young
Pvalb	1.04E-06	0.01798989	Old_vs_Young

Tbc1d7	1.07E-07	0.00186618	Old_vs_Young
Cst6	1.13E-06	0.01967025	Old_vs_Young
Jund	9.56E-12	1.66E-07	Old_vs_Young
Prdx5	2.31E-10	4.01E-06	Old_vs_Young
1700024G13Rik	3.14E-11	5.45E-07	Old_vs_Young
Purb	3.21E-17	5.57E-13	Old_vs_Young
Arf5	1.58E-09	2.75E-05	Old_vs_Young
Kif21a	4.24E-12	7.37E-08	Old_vs_Young
Ttc21a	1.78E-15	3.09E-11	Old_vs_Young
Slc9a3r2	2.64E-11	4.58E-07	Old_vs_Young
Espn	4.04E-40	7.01E-36	Old_vs_Young
Rsph1	1.05E-17	1.83E-13	Old_vs_Young
Kncn	1.24E-16	2.15E-12	Old_vs_Young
Slc25a4	1.05E-22	1.82E-18	Old_vs_Young
Cfap126	2.44E-20	4.23E-16	Old_vs_Young
Lrrc73	6.92E-12	1.20E-07	Old_vs_Young
1700001C02Rik	2.08E-12	3.61E-08	Old_vs_Young
Tuba1a	8.90E-19	1.55E-14	Old_vs_Young
Slc17a8	3.33E-11	5.78E-07	Old_vs_Young
Lrrc10b	1.88E-24	3.26E-20	Old_vs_Young
Atxn7l3b	3.90E-29	6.78E-25	Old_vs_Young
Nek2	5.82E-13	1.01E-08	Old_vs_Young
Fbxo2	3.65E-40	6.34E-36	Old_vs_Young
Cib2	2.04E-07	0.00354984	Old_vs_Young

Figure 6 source data

6A-C

ID (BP)	Description	p.adjust
GO:0030317	flagellated sperm motility	5.15E-05
GO:0003341	cilium movement	5.15E-05
GO:0097722	sperm motility	5.15E-05
GO:0060294	cilium movement involved in cell motility	8.32E-05
GO:0001539	cilium or flagellum-dependent cell motility	0.000111872

GO:0060285	cilium-dependent cell motility	0.000111872
GO:0044782	cilium organization	0.000144329
GO:0042026	protein refolding	0.000815047
GO:0007018	microtubule-based movement	0.001240979
GO:0007286	spermatid development	0.025249942
GO:0035082	axoneme assembly	0.025249942
GO:0048515	spermatid differentiation	0.032159824
GO:1903320	regulation of protein modification by small protein conjugation or removal	0.0331534
GO:0006457	protein folding	0.0331534
GO:0007605	sensory perception of sound	0.04399092
GO:0031396	regulation of protein ubiquitination	0.044295112
**ID (CC)**	**Description**	**p.adjust**
GO:0032421	stereocilium bundle	9.89E-07
GO:0032420	stereocilium	4.32E-06
GO:0098862	cluster of actin-based cell projections	4.61E-06
GO:0032437	cuticular plate	3.20E-05
GO:0031514	motile cilium	0.000616928
GO:0097729	9+2 motile cilium	0.003784283
GO:0030864	cortical actin cytoskeleton	0.004639083
GO:0005874	microtubule	0.0046578
GO:0098858	actin-based cell projection	0.005200039
GO:0032426	stereocilium tip	0.005772533
GO:0015629	actin cytoskeleton	0.007768532

GO:0036126	sperm flagellum	0.009911794
GO:0030863	cortical cytoskeleton	0.011187656
GO:0043209	myelin sheath	0.022140694
GO:0098993	anchored component of synaptic vesicle membrane	0.034291918
GO:0036064	ciliary basal body	0.037937079
GO:0030672	synaptic vesicle membrane	0.037937079
GO:0099501	exocytic vesicle membrane	0.037937079
GO:0098563	intrinsic component of synaptic vesicle membrane	0.039558434

6D-E

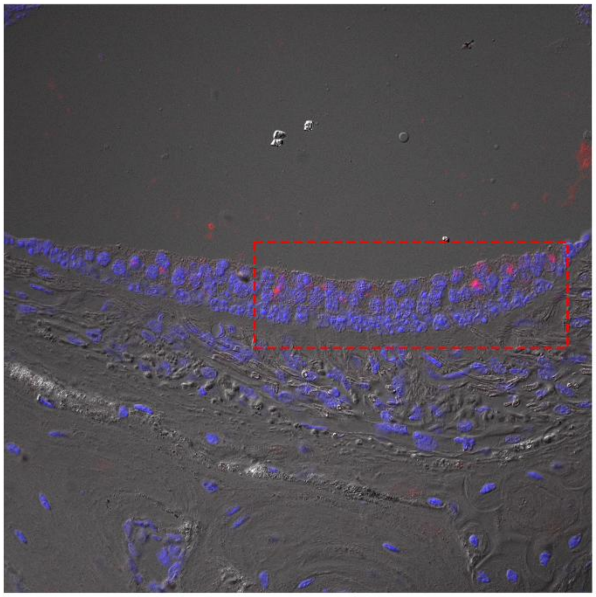

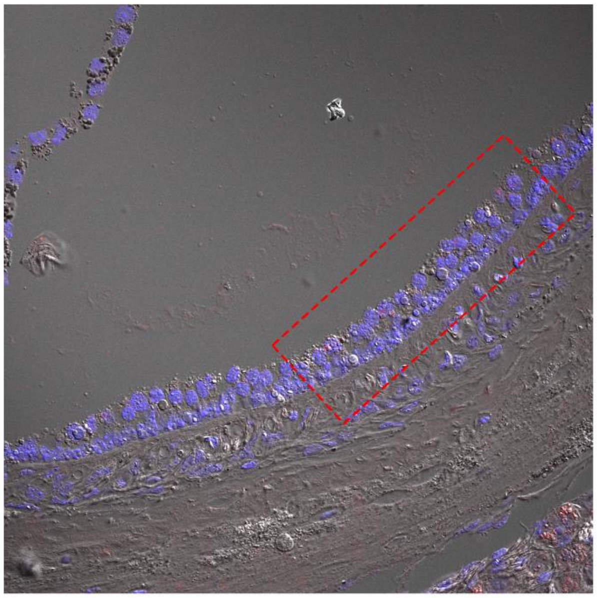

6F, G

Gene	HCI_young	HCII_young	HCI_old	HCII_old
App	1.78930018	0	12.7565563	11.4482851
Mapt	7.00827776	7.37097733	8.51175265	9.5836675
Nefl	7.91727804	8.64594939	11.5137276	11.242895
Apoe	9.12572113	8.76764787	11.509775	10.7849122
Snca	6.66839356	6.37966655	5.04439412	0
Pink1	7.66128424	7.02542901	8.55842071	7.57378167

Lrrk2	3.06512689	4.61498962	2.3219281	0
Tardbp	7.69587996	7.95303175	9.62570884	9.8255778
Fus	8.49042931	9.22971089	10.2609195	10.5434402
C9orf72	7.39266163	7.26473239	7.65105169	9.23484828
Htt	6.89373412	6.57014422	8.3219281	8.7184816

Figure 7 source data

7B

Espn

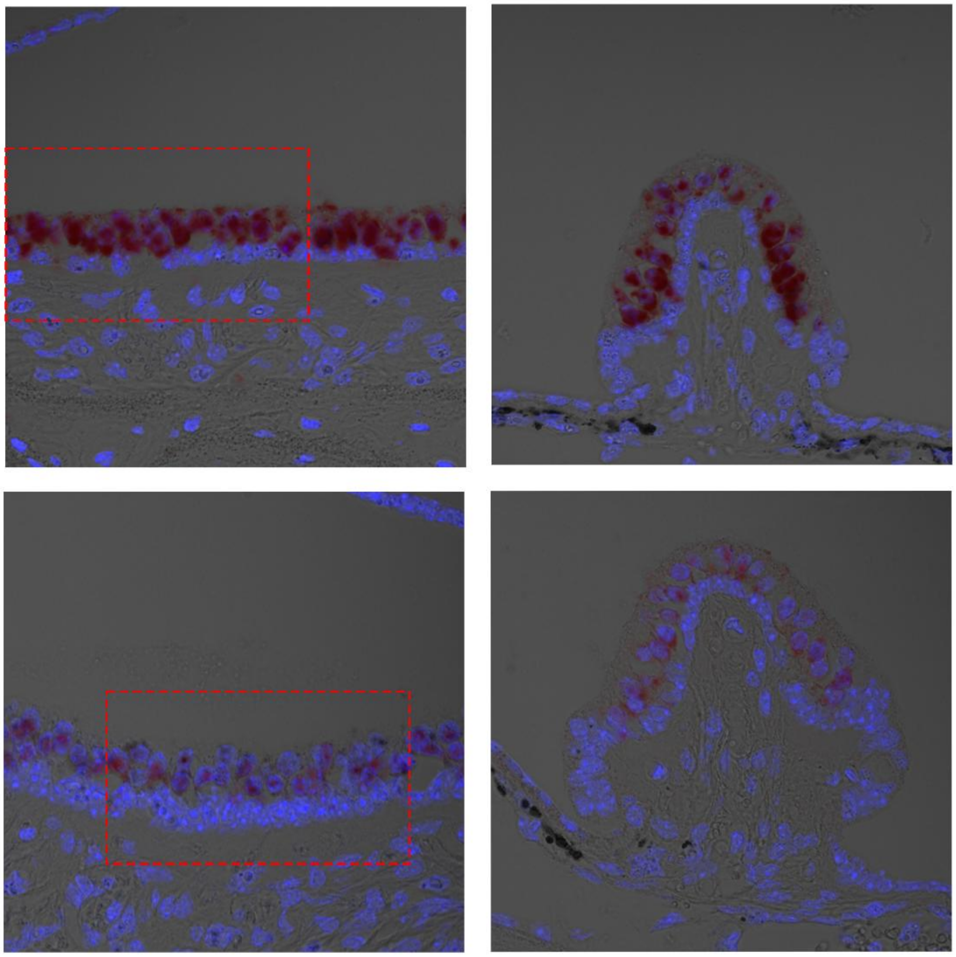

Pou4f3

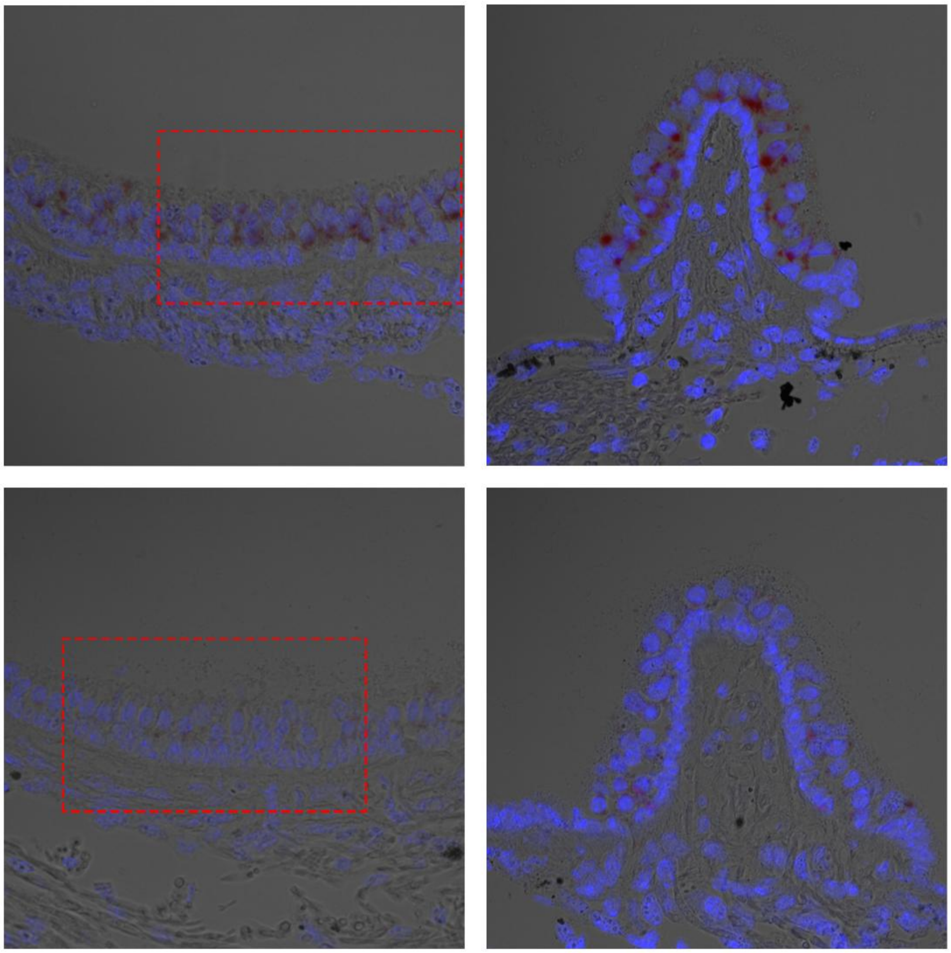

Fbxo2

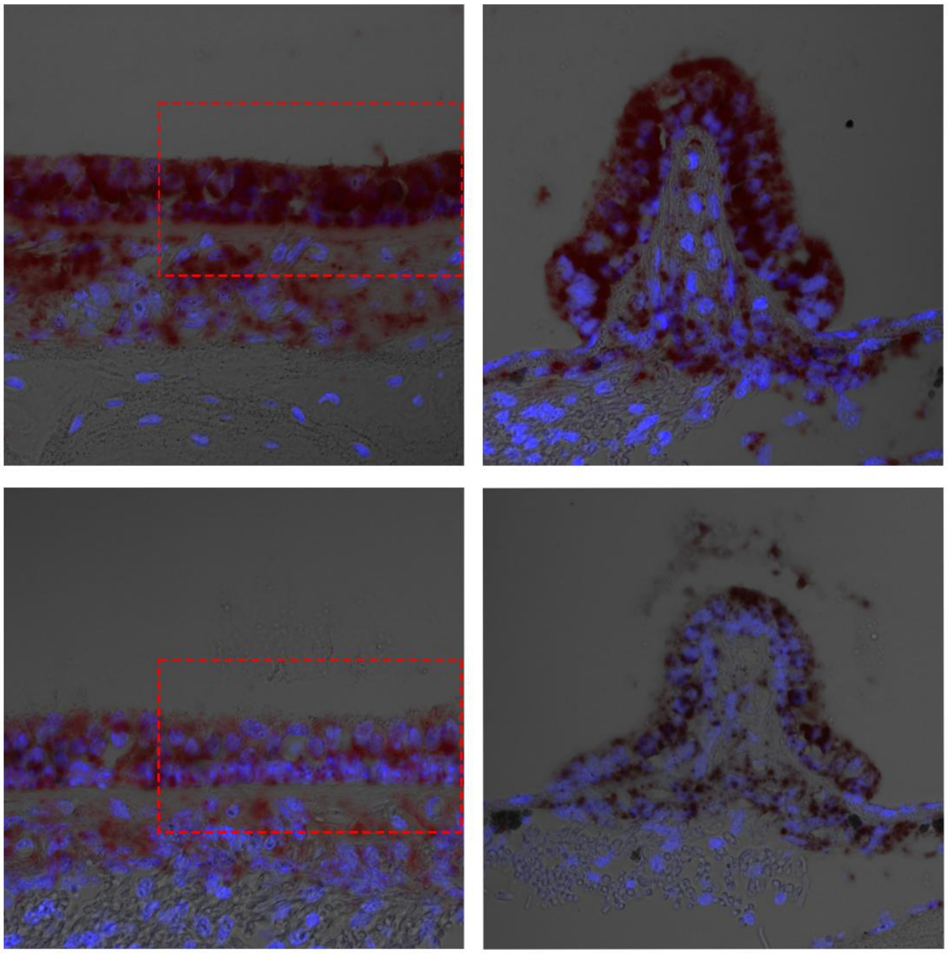

Tmc1

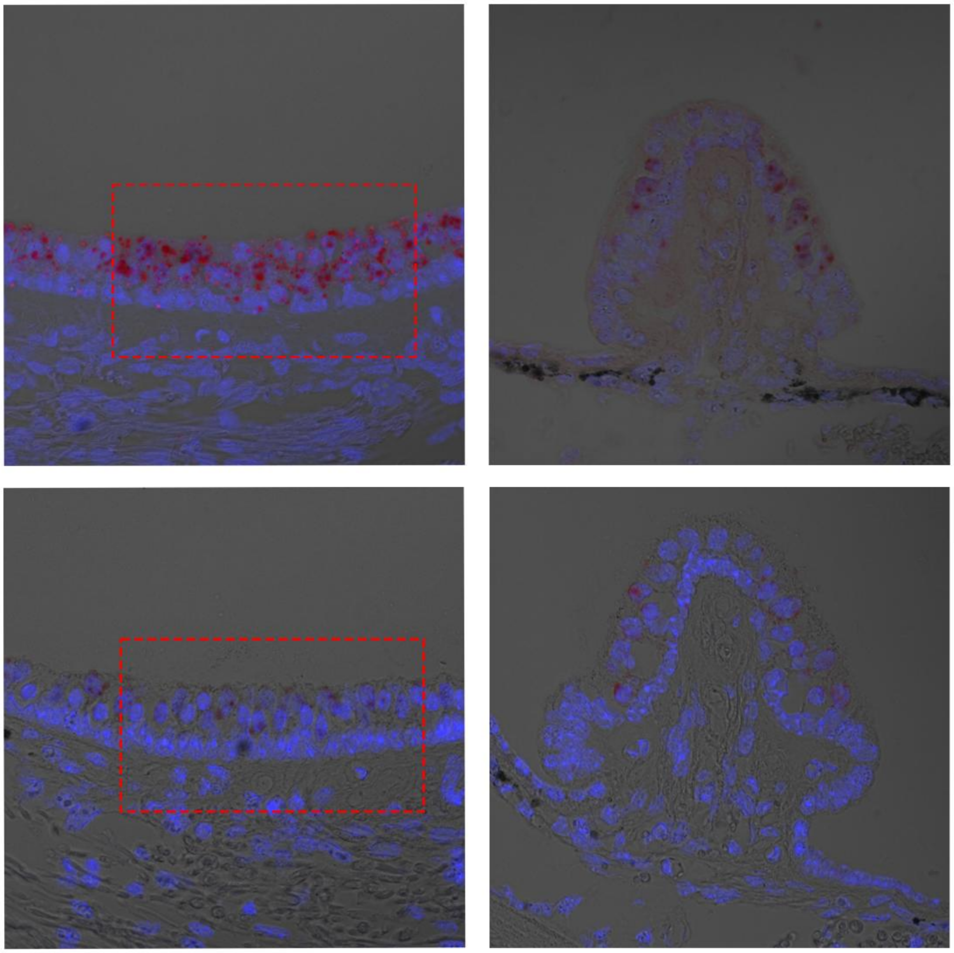

Atp2b2

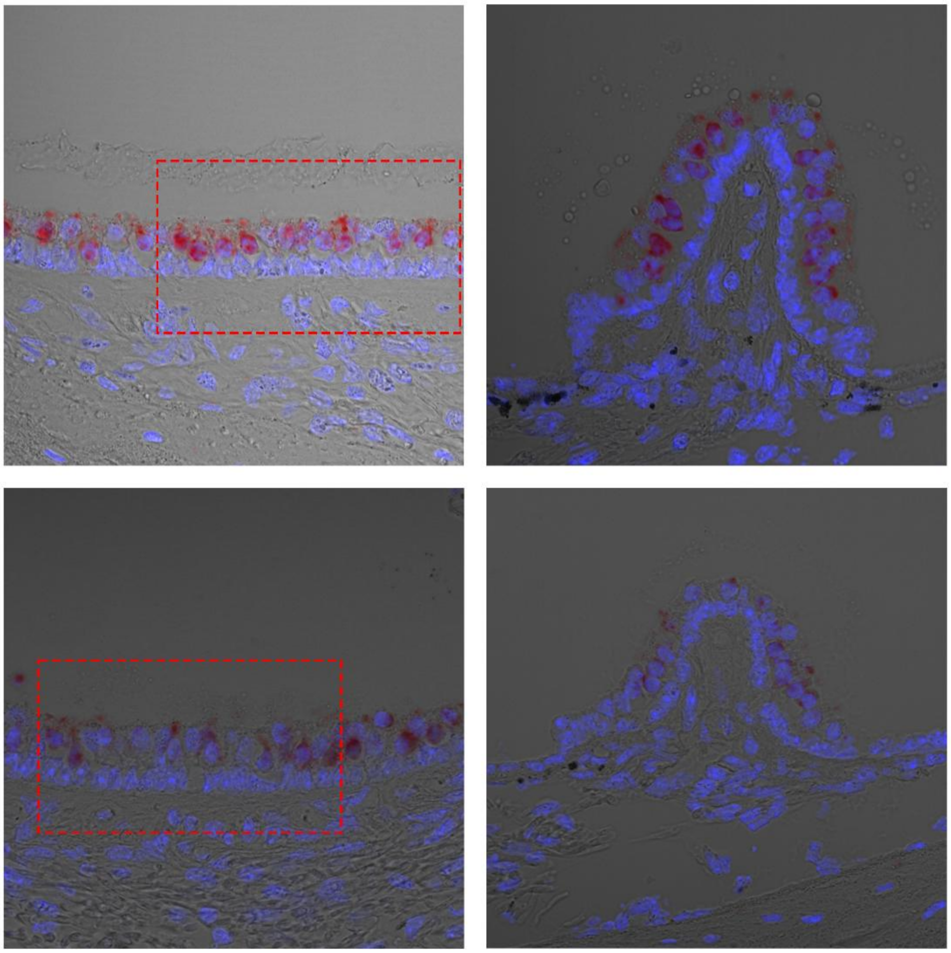

Homer2

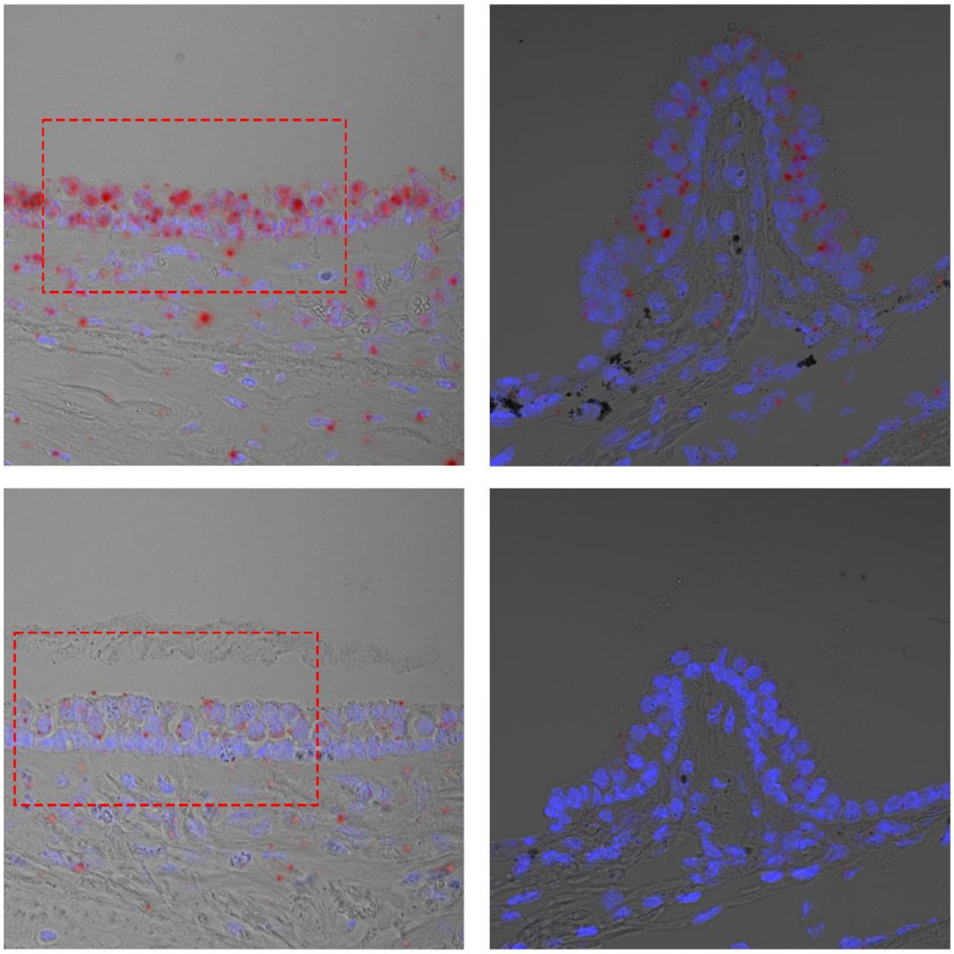

7C

Espn	Otoliths	Crista
Young	Puncta signal density	Puncta signal density
#1	0.58402998	0.22098239
#2	0.47056742	0.33068936
#3	0.60354182	0.05474059
Old		
#1	0.11548344	0.11963809
#2	0.12088185	0.05159453
#3	0.17182083	0.00955635
Tmc1		
Young	Puncta signal density	Puncta signal density

#1	0.34072877	0.09376316
#2	0.26045676	0.04709853
#3	0.23597102	0.12508931
Old		
#1	0.02036379	0.03126892
#2	0.04731654	0.04281369
#3	0.04757407	0.00330469
Fbxo2		
Young	Puncta signal density	Puncta signal density
#1	0.68526433	0.61531395
#2	0.75005623	0.46241856
#3	0.69806989	0.49833867
Old		
#1	0.22977928	0.23007193
#2	0.17982137	0.32770369
#3	0.22010714	0.01157195
Pou4f3		
Young	Puncta signal density	Puncta signal density
#1	0.28389204	0.13478205
#2	0.29322196	0.12583469
#3	0.16251072	0.03747058
Old		
#1	0.00483231	0.02003746
#2	0.04066304	0.02653623
#3	0.06044068	7.8528E-05
Homer2		
Young	Puncta signal density	Puncta signal density
#1	0.09490457	0.24042564
#2	0.21251481	0.24898646
#3	0.23109609	0.10624896
Old		
#1	0.01799522	0.1770617
#2	0.04739053	0.05198467
#3	0.01640841	0.06876277
Atp2b2		

Young	Puncta signal density	Puncta signal density
#1	0.05010143	0.13168217
#2	0.38229283	0.15802487
#3	0.08059971	0.02659285
Old		
#1	0.01131484	0.02138415
#2	0.08033427	0.04217474
#3	0.06403578	0.00366774

7D

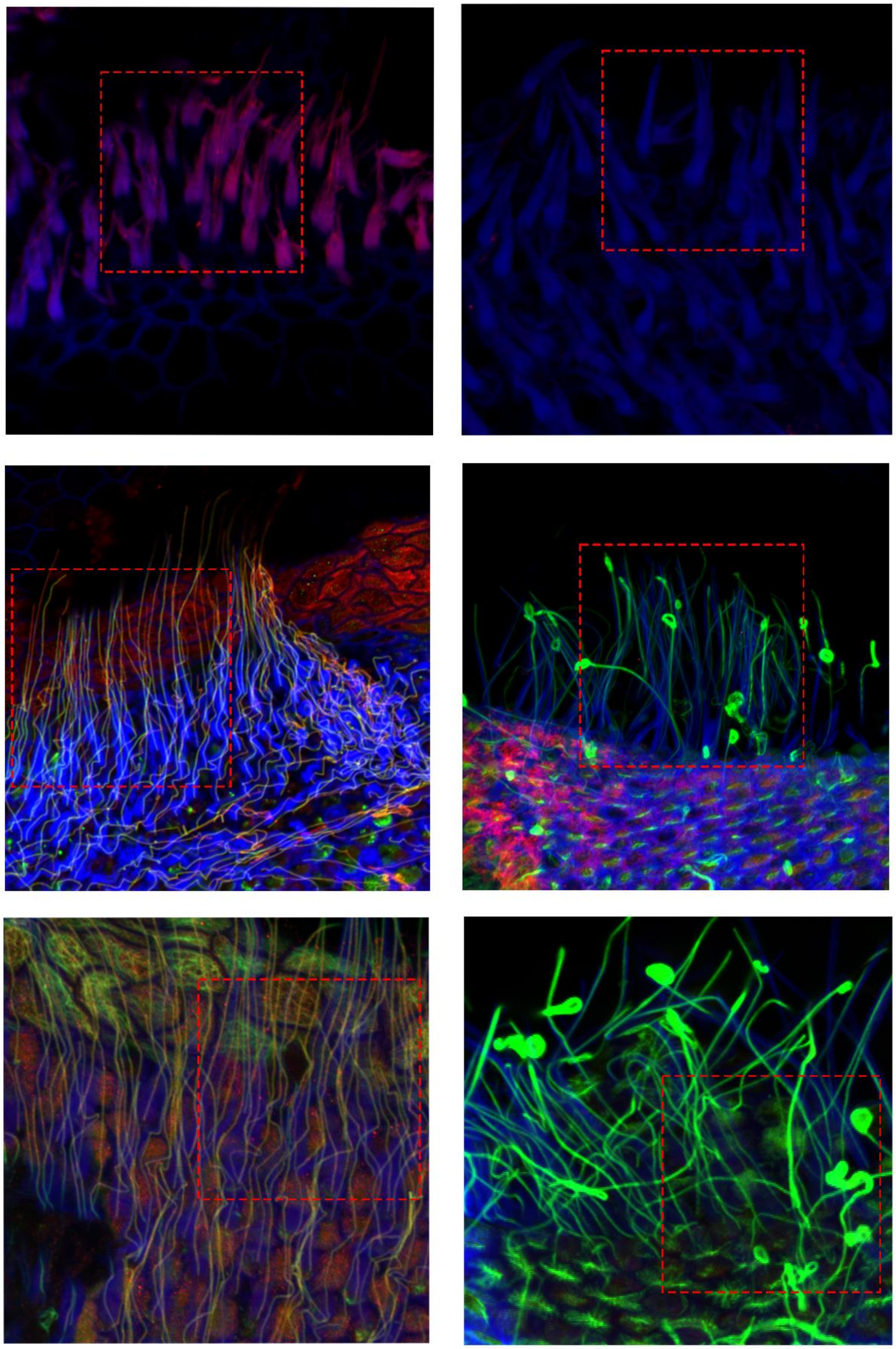

Figure 8 source data

SASP

Gene	Pathway
Pde5A	chronic inflammatory response
Lrrk2	microglial cell activation
Bok	mitochondrial outer membrane permeabilization involved in apoptotic signaling pathway
Bcl2L12	senescence
Zfp13	mitochondrial outer membrane permeabilization involved in apoptotic signaling pathway
Apod	cytokine production involved in inflammatory response
Slc7A11	neutrophil apoptotic process
Pla2G3	histamine secretion by mast cell
Cxcl12	intrinsic apoptotic signaling pathway in dna damage
Fabp4	inflammatory response
Ldlr	astrocyte activation
Arrb2	release of cytochrome c from mitochondria
Nod2	cytokine production involved in inflammatory response
Lrp1	astrocyte activation involved in immune response
Tradd	inflammatory response
Aifm2	apoptotic mitochondrial changes
Plekho2	macrophage apoptotic process
Skil	intrinsic apoptotic signaling pathway in dna damage
Anxa1	neutrophil apoptotic process
Bnip3	mitochondrial fragmentation involved in apoptotic process
Bcl2L11	mitochondrial membrane permeability involved in apoptotic process
Eya2	mitochondrial outer membrane permeabilization
Ccl5	neuroinflammatory response
Ier3	mitochondrial outer membrane permeabilization involved in apoptotic signaling pathway
Hic1	intrinsic apoptotic signaling pathway in dna damage
Casp9	intrinsic apoptotic signaling pathway in dna damage
Stat5A	inflammatory response
Cx3Cr1	microglial cell activation
Ccar2	mitochondrial fragmentation involved in apoptotic process
Map3K1	apoptotic mitochondrial changes
Usp28	intrinsic apoptotic signaling pathway in dna damage by p53 class mediator
Bbc3	release of cytochrome c from mitochondria
Myo5A	reactive gliosis

Erbb4	mitochondrial fragmentation involved in apoptotic process
Cd47	inflammatory response
Zc3H12A	cytokine production involved in inflammatory response
Mif	senescence
Nmi	inflammatory response
Nlrc3	cytokine production involved in inflammatory response
Gpr4	inflammatory response
Bcl2	apoptotic mitochondrial changes
Cd24A	inflammatory antigenic stimulus
Ercc6	intrinsic apoptotic signaling pathway in dna damage
Zeb2	astrocyte activation
Wdr35	release of cytochrome c from mitochondria
Brca2	intrinsic apoptotic signaling pathway in dna damage
Large1	reactive gliosis
Suv39H1	senescence
B2M	senescence
Tbx2	senescence
Pml	intrinsic apoptotic signaling pathway in dna damage
Selenos	macrophage apoptotic process
C2Cd4B	acute inflammatory response
Nfkb1	inflammatory response
Sirpa	cytokine production involved in inflammatory response
Prkn	mitochondrial fragmentation involved in apoptotic process
Rad9A	intrinsic apoptotic signaling pathway in dna damage
Il16	inflammatory response
Trp53	senescence
Mllt11	release of cytochrome c from mitochondria
Ppif	apoptotic mitochondrial changes
Eef1E1	senescence
Itpkb	neutrophil apoptotic process
Fmc1	apoptotic mitochondrial changes
Ddt	inflammatory response
Rap1Gds1	cytokine production involved in inflammatory response
Mapk3	neuroinflammatory response
Bcl2L2	release of cytochrome c from mitochondria
Vps54	neuroinflammatory response
Zkscan3	senescence
Mapk9	cytokine production involved in inflammatory response
Crip1	intrinsic apoptotic signaling pathway in dna damage
Ep300	intrinsic apoptotic signaling pathway in dna damage by p53 class mediator

Ppm1K	mitochondrial membrane permeability involved in apoptotic process
Rps19	respiratory burst involved in inflammatory response
Setd4	inflammatory response
Ybx1	senescence
Vps13A	neuroinflammatory response
Mtch2	mitochondrial membrane permeability involved in apoptotic process
Pla2G6	release of cytochrome c from mitochondria
Rhot1	mitochondrial outer membrane permeabilization
Slc9A6	glial cell activation
Cuedc2	cytokine production involved in inflammatory response
Dnm1L	mitochondrial fragmentation involved in apoptotic process
Cdkn2D	intrinsic apoptotic signaling pathway in dna damage
Casp3	pyroptotic inflammatory response
Rpl26	intrinsic apoptotic signaling pathway in dna damage by p53 class mediator
Mapk1	neuroinflammatory response
Xpa	intrinsic apoptotic signaling pathway in dna damage
Triap1	release of cytochrome c from mitochondria
Atm	microglial cell activation
Hip1R	mitochondrial outer membrane permeabilization involved in apoptotic signaling pathway
Ankrd42	cytokine production involved in inflammatory response
Hnrnpk	intrinsic apoptotic signaling pathway in dna damage by p53 class mediator
Clock	inflammatory response
Ggct	release of cytochrome c from mitochondria
Tmem161A	intrinsic apoptotic signaling pathway in dna damage
Sirt1	senescence
Prdx2	respiratory burst involved in inflammatory response
Zfp277	senescence
Seh1L	cytokine production involved in inflammatory response
Pias4	intrinsic apoptotic signaling pathway in dna damage
Atad5	intrinsic apoptotic signaling pathway in dna damage
Ghitm	release of cytochrome c from mitochondria
Casp2	intrinsic apoptotic signaling pathway in dna damage
Pbxip1	production of molecular mediator involved in inflammatory response
Morc3	senescence
Fam162A	release of cytochrome c from mitochondria
Pten	senescence
Topors	intrinsic apoptotic signaling pathway in dna damage
Zmpste24	senescence
Polb	intrinsic apoptotic signaling pathway in dna damage

Vps35	mitochondrial fragmentation involved in apoptotic process
Rps3	intrinsic apoptotic signaling pathway in dna damage
Akt3	senescence
Ypel3	senescence
Tpt1	intrinsic apoptotic signaling pathway in dna damage
S100A9	inflammatory response
Mef2C	macrophage apoptotic process
Ccn4	inflammatory response
S100A8	inflammatory response
Calhm2	microglial cell activation
Il17Ra	inflammatory response
Abcc1	inflammatory response
Egfr	astrocyte activation
Ccr2	inflammatory response
Fcer1G	type i hypersensitivity
Fnip2	intrinsic apoptotic signaling pathway in dna damage
Zfp36	cytokine production involved in inflammatory response
Igf1	neuroinflammatory response
Bik	apoptotic mitochondrial changes
Trim32	intrinsic apoptotic signaling pathway in dna damage
Adcy8	neuroinflammatory response
Abcd1	cytokine production involved in inflammatory response
Tyrobp	microglial cell activation involved in immune response
Ei24	intrinsic apoptotic signaling pathway in dna damage
Fzr1	senescence
Dagla	neuroinflammatory response
Pik3Cd	neutrophil apoptotic process
Shisa5	intrinsic apoptotic signaling pathway in dna damage by p53 class mediator
Itgam	microglial cell activation
Hmox1	cytokine production involved in inflammatory response
Camk2N1	inflammatory response
Nptx1	mitochondrial fragmentation involved in apoptotic process
Naglu	astrocyte activation
Gna14	inflammatory response
C1Qa	astrocyte activation
Uaca	intrinsic apoptotic signaling pathway in dna damage
Mtor	senescence
Prkaa2	release of cytochrome c from mitochondria
Gpx1	release of cytochrome c from mitochondria
Kras	senescence

Mpv17L	mitochondrial outer membrane permeabilization involved in apoptotic signaling pathway
Jun	microglial cell activation
Gprc5B	inflammatory response
Timm50	release of cytochrome c from mitochondria
Sirt6	senescence
St6Gal1	macrophage apoptotic process
Fem1A	inflammatory response
Adam17	production of molecular mediator involved in inflammatory response
Abl1	senescence
Pik3Cb	neutrophil apoptotic process
Ptpn6	cytokine production involved in inflammatory response
Dap3	apoptotic mitochondrial changes
Akt1	apoptotic mitochondrial changes
Nek4	senescence
Prkdc	senescence
Nr3C1	neuroinflammatory response
Ttc1	neuroinflammatory response
Ninj1	inflammatory response
Atg3	mitochondrial fragmentation involved in apoptotic process
Sod2	apoptotic mitochondrial changes
Tnip1	inflammatory response
Fis1	mitochondrial fragmentation involved in apoptotic process
Ilk	senescence
Park7	acute inflammatory antigenic stimulus
Bpgm	neuroinflammatory response
Per1	cytokine production involved in inflammatory response
Mul1	mitochondrial outer membrane permeabilization involved in apoptotic signaling pathway
Psen1	astrocyte activation
Pnp	apoptotic mitochondrial changes
Hspd1	apoptotic mitochondrial changes
Parl	release of cytochrome c from mitochondria
Msh2	intrinsic apoptotic signaling pathway in dna damage
Bnip3L	mitochondrial outer membrane permeabilization
Terf2	senescence
Bax	apoptotic mitochondrial changes
Slc25A4	apoptotic mitochondrial changes
App	astrocyte activation
Opa1	release of cytochrome c from mitochondria

Gsk3B	mitochondrial outer membrane permeabilization involved in apoptotic signaling pathway
Gclm	apoptotic mitochondrial changes
Prkaa1	release of cytochrome c from mitochondria
Cebpb	inflammatory response
Cxcr2	neutrophil apoptotic process
Ttbk1	astrocyte activation
C3	type iia hypersensitivity
Ptger4	inflammatory response
Traf3Ip2	cytokine production involved in inflammatory response
Zbp1	inflammatory response
Lipa	respiratory burst involved in inflammatory response
Steap3	intrinsic apoptotic signaling pathway in dna damage by p53 class mediator
Trem2	astrocyte activation
Gch1	senescence
Mmp9	release of cytochrome c from mitochondria
Gbp2	pyroptotic inflammatory response
Pde2A	inflammatory response
Plscr3	release of cytochrome c from mitochondria
Il33	microglial cell activation involved in immune response
Mmp8	microglial cell activation
Tlr2	microglial cell activation
Plaur	release of cytochrome c from mitochondria
Sfn	release of cytochrome c from mitochondria
Tlr3	microglial cell activation
Naif1	mitochondrial membrane permeability involved in apoptotic process
Sphk1	microglial cell activation
Rbl1	senescence
Il1B	astrocyte activation
Trpv4	inflammatory response
Cx3Cl1	microglial cell activation
E2F1	intrinsic apoptotic signaling pathway in dna damage
Il17Rc	cytokine production involved in inflammatory response
Arg2	senescence
Map3K8	inflammatory response
Ephb2	cytokine production involved in inflammatory response
Ackr3	intrinsic apoptotic signaling pathway in dna damage
Nol3	mitochondrial membrane permeability involved in apoptotic process
Fzd9	mitochondrial outer membrane permeabilization involved in apoptotic signaling pathway
Casp12	inflammatory response

Fbxo5	senescence
Syk	serotonin secretion by platelet
Kcnn4	microglial cell activation
Nkg7	inflammatory response
Smo	astrocyte activation
Wnt5A	inflammatory response
Bcl6	senescence
Adcy1	neuroinflammatory response
Ppp2R2B	mitochondrial fragmentation involved in apoptotic process
Csf1R	microglial cell activation
Tlr4	microglial cell activation
Gsdmd	inflammatory response
Ccr5	fever generation
Chil3	production of molecular mediator involved in inflammatory response
Ephx2	prostaglandin production involved in inflammatory response
Casp8	pyroptotic inflammatory response
Lpl	inflammatory response
Il17Rb	inflammatory response
Gpsm3	inflammatory response
Knl1	intrinsic apoptotic signaling pathway in dna damage by p53 class mediator
Fcgr3	type i hypersensitivity
Il17D	cytokine production involved in inflammatory response
Alox5Ap	acute inflammatory response
Hrk	release of cytochrome c from mitochondria
Bid	apoptotic mitochondrial changes
Zdhhc5	pyroptotic inflammatory response
Stap1	microglial cell activation
Hk2	apoptotic mitochondrial changes
Twist1	senescence
Tmem102	mitochondrial outer membrane permeabilization involved in apoptotic signaling pathway
Ncf1	respiratory burst involved in inflammatory response
Tgfb1	inflammatory response
Tnfrsf1A	inflammatory response
Tmem109	intrinsic apoptotic signaling pathway in dna damage by p53 class mediator
Grn	astrocyte activation involved in immune response
Snap23	histamine secretion by mast cell
Ctsc	microglial cell activation
Syt11	microglial cell activation
Ppp2Cb	apoptotic mitochondrial changes

Hipk2	intrinsic apoptotic signaling pathway in dna damage by p53 class mediator
Ulk4	neuroinflammatory response
Ager	astrocyte activation
Msh6	intrinsic apoptotic signaling pathway in dna damage
Pdcd4	inflammatory response
Phlda3	intrinsic apoptotic signaling pathway in dna damage by p53 class mediator
Hspa1A	mitochondrial outer membrane permeabilization involved in apoptotic signaling pathway
Lyn	neuroinflammatory response
Pik3R1	intrinsic apoptotic signaling pathway in dna damage
Itgb1	reactive gliosis
Mlh1	intrinsic apoptotic signaling pathway in dna damage
Napepld	inflammatory response
Them4	mitochondrial membrane permeability involved in apoptotic process
Pawr	senescence
Il18	inflammatory response
Cdk6	senescence
Bcl3	intrinsic apoptotic signaling pathway in dna damage by p53 class mediator
Stat3	inflammatory response
Cdkn1A	intrinsic apoptotic signaling pathway in dna damage by p53 class mediator
Nuak1	senescence
Ets1	inflammatory response
Aif1	microglial cell activation
Mdk	inflammatory response
Moap1	release of cytochrome c from mitochondria
Bad	release of cytochrome c from mitochondria
Nr1D1	astrocyte activation
Kdm1A	intrinsic apoptotic signaling pathway in dna damage by p53 class mediator
Ckmt1	mitochondrial outer membrane permeabilization involved in apoptotic signaling pathway
Aifm1	apoptotic mitochondrial changes
Vamp7	histamine secretion by mast cell
Mcl1	apoptotic mitochondrial changes
Casp6	release of cytochrome c from mitochondria
Cdip1	intrinsic apoptotic signaling pathway in dna damage by p53 class mediator
Bcl2L1	apoptotic mitochondrial changes
Tmem14A	mitochondrial outer membrane permeabilization involved in apoptotic signaling pathway
Cd44	neutrophil apoptotic process
Bmpr1A	senescence
Fads2	arachidonate metabolite production involved in inflammatory response

Lgals1	inflammatory response
Appl1	cytokine production involved in inflammatory response
Cd81	inflammatory antigenic stimulus
Appl2	cytokine production involved in inflammatory response
Ifi35	inflammatory response
Higd1A	release of cytochrome c from mitochondria
Cd200	neuroinflammatory response
Nek7	inflammatory response
Vamp8	histamine secretion by mast cell
Slc25A5	mitochondrial outer membrane permeabilization involved in apoptotic signaling pathway
Zfp385A	intrinsic apoptotic signaling pathway in dna damage by p53 class mediator
Prelid1	release of cytochrome c from mitochondria
Ywhaz	histamine secretion by mast cell
Psmd10	release of cytochrome c from mitochondria
Snw1	intrinsic apoptotic signaling pathway in dna damage by p53 class mediator
Rsl1D1	senescence
Hspa4	microglial cell activation
Abcd2	cytokine production involved in inflammatory response
Hif1A	cytokine production involved in inflammatory response
Dyrk2	intrinsic apoptotic signaling pathway in dna damage by p53 class mediator
Camp	prostaglandin production involved in inflammatory response
Cd74	intrinsic apoptotic signaling pathway in dna damage by p53 class mediator
Snca	microglial cell activation
Mbd4	intrinsic apoptotic signaling pathway in dna damage
Snx4	histamine secretion by mast cell
Plk2	senescence
Pomgnt1	reactive gliosis
Psen2	neuroinflammatory response
Mfn2	release of cytochrome c from mitochondria
Slc35F6	mitochondrial outer membrane permeabilization involved in apoptotic signaling pathway
Rhot2	mitochondrial outer membrane permeabilization
Bak1	mitochondrial outer membrane permeabilization involved in apoptotic signaling pathway
Ticam1	cytokine production involved in inflammatory response
Slc7A2	nitric oxide production involved in inflammatory response
Stat5B	inflammatory response
Ddit4	intrinsic apoptotic signaling pathway in dna damage by p53 class mediator
Pycard	inflammatory response
Mapk13	inflammatory response

Aen	intrinsic apoptotic signaling pathway in dna damage by p53 class mediator
Hmga1	inflammatory cell apoptotic process
Icmt	senescence
Smad3	release of cytochrome c from mitochondria
Map3K7	cytokine production involved in inflammatory response
Chid1	cytokine production involved in inflammatory response
Bmf	release of cytochrome c from mitochondria
Nfkbiz	inflammatory response
Pink1	release of cytochrome c from mitochondria
Pim2	apoptotic mitochondrial changes
Fxn	release of cytochrome c from mitochondria
Plscr1	intrinsic apoptotic signaling pathway in dna damage
Bloc1S2	mitochondrial outer membrane permeabilization
Nacc2	intrinsic apoptotic signaling pathway in dna damage
Nupr1	neuroinflammatory response
Extl3	cytokine production involved in inflammatory response
Ripk1	inflammatory response
Atp7A	release of cytochrome c from mitochondria
Hyal2	inflammatory response
Prkca	inflammatory response
Siva1	mitochondrial outer membrane permeabilization involved in apoptotic signaling pathway
Gsk3A	mitochondrial outer membrane permeabilization involved in apoptotic signaling pathway
Gclc	mitochondrial outer membrane permeabilization involved in apoptotic signaling pathway
Il4Ra	production of molecular mediator involved in inflammatory response
Rela	cytokine production involved in inflammatory response
Clu	microglial cell activation
Bap1	cytokine production involved in inflammatory response
Dusp10	respiratory burst involved in inflammatory response
Pomt2	reactive gliosis
Nampt	senescence
Ezh2	cytokine production involved in inflammatory response
Uri1	intrinsic apoptotic signaling pathway in dna damage by p53 class mediator
Pnpt1	senescence
Vdac2	mitochondrial outer membrane permeabilization
Myd88	cytokine production involved in inflammatory response
Kpna6	cytokine production involved in inflammatory response
Htra2	intrinsic apoptotic signaling pathway in dna damage
Pld3	cytokine production involved in inflammatory response

Dhx9	inflammatory response
Lmna	release of cytochrome c from mitochondria
Usp47	intrinsic apoptotic signaling pathway in dna damage
Acaa2	mitochondrial membrane permeability involved in apoptotic process
Rps27L	intrinsic apoptotic signaling pathway in dna damage by p53 class mediator
Ppp2R5C	intrinsic apoptotic signaling pathway in dna damage by p53 class mediator
Fbh1	intrinsic apoptotic signaling pathway in dna damage
Sbno1	neuroinflammatory response
Atf2	mitochondrial membrane permeability involved in apoptotic process
Ctss	inflammatory response
Jak2	microglial cell activation
Mapk14	cytokine production involved in inflammatory response
Hipk1	intrinsic apoptotic signaling pathway in dna damage by p53 class mediator
Bag6	intrinsic apoptotic signaling pathway in dna damage by p53 class mediator
Zdhhc9	pyroptotic inflammatory response
Jtb	apoptotic mitochondrial changes
Adam8	acute inflammatory response
Ikbkb	cytokine production involved in inflammatory response
Nfkbia	inflammatory response
Rnf7	apoptotic mitochondrial changes
Ptgs2	neuroinflammatory response
Ccr1	neuroinflammatory response
Gbp3	pyroptotic inflammatory response
Lbp	respiratory burst involved in inflammatory response
Tslp	inflammatory response
Cd96	cytokine production involved in inflammatory response
Brca1	intrinsic apoptotic signaling pathway in dna damage
Fcgr2B	neutrophil apoptotic process
Camk2A	mitochondrial membrane permeability involved in apoptotic process
Bcl2A1B	release of cytochrome c from mitochondria
Nlrp3	inflammatory response

Proteostasis

Gene	Pathway
Nacad	unfolded protein binding
Dnajb13	unfolded protein binding
Bok	perkmediated unfolded protein response
Cav1	misfolded protein
Optn	unfolded protein

Lonrf2	misfolded protein binding
Klhl15	nuclear protein quality control by the ubiquitinproteasome system
Tmem67	unfolded protein binding
Hspb8	unfolded protein
Pmp22	unfolded protein
Wfs1	endoplasmic reticulum unfolded protein response
Cryab	protein refolding
Dnaja3	unfolded protein binding
Serp1	endoplasmic reticulum unfolded protein response
Ncoa3	nuclear protein quality control by the ubiquitinproteasome system
Fbxo6	unfolded protein
Dnajb3	unfolded protein binding
Elp6	unfolded protein
Wdr83Os	protein folding chaperone
B2M	protein refolding
Derl3	endoplasmic reticulum unfolded protein response
Abcb10	mitochondrial unfolded protein response
Selenos	endoplasmic reticulum unfolded protein response
Edem1	unfolded protein
Ube2W	misfolded protein
Pdcl3	protein folding chaperone
Prkn	unfolded protein
Trap1	unfolded protein binding
Ufl1	ire1mediated unfolded protein response
Nfe2L2	endoplasmic reticulum unfolded protein response
Vbp1	unfolded protein binding
Dnajb12	misfolded protein
Atad3A	perkmediated unfolded protein response
Canx	unfolded protein binding
Yod1	endoplasmic reticulum unfolded protein response
Jkamp	unfolded protein
Creb3	endoplasmic reticulum unfolded protein response
Epg5	unfolded protein
Tmbim4	endoplasmic reticulum unfolded protein response
Ptges3	unfolded protein binding
Ube4B	unfolded protein binding
Eif2Ak3	endoplasmic reticulum unfolded protein response
Tram1	unfolded protein
Ndufaf1	unfolded protein binding

Hspa8	protein refolding
Ufd1	misfolded protein
Sirt1	endoplasmic reticulum unfolded protein response
Tsc1	protein folding chaperone
Akirin2	nuclear protein quality control by the ubiquitinproteasome system
Scg5	unfolded protein binding
Nudcd2	unfolded protein binding
Tor1A	misfolded protein binding
Derl2	endoplasmic reticulum unfolded protein response
Herpud2	endoplasmic reticulum unfolded protein response
Dnlz	protein folding chaperone
Pfdn5	unfolded protein binding
Xbp1	endoplasmic reticulum unfolded protein response
Pofut2	protein folding
Atf6B	endoplasmic reticulum unfolded protein response
Atf4	endoplasmic reticulum unfolded protein response
Sdf2	protein refolding
Ube2J2	unfolded protein
Nck2	perkmediated unfolded protein response
Vcp	misfolded protein
Akt3	perkmediated unfolded protein response
Tcp1	unfolded protein binding
Cdk5Rap3	endoplasmic reticulum unfolded protein response
Tmem129	unfolded protein
Wipf1	protein folding chaperone
Rhbdd1	unfolded protein
Edem2	endoplasmic reticulum unfolded protein response
Ppp1R15A	endoplasmic reticulum unfolded protein response
Mkks	unfolded protein binding
Ern 1.00	unfolded protein
Hspa2	protein refolding
Derl1	misfolded protein
Dnajc18	misfolded protein
Aip	unfolded protein binding
Dab2Ip	unfolded protein
Stt3B	unfolded protein
Vapb	endoplasmic reticulum unfolded protein response
Rara	nuclear protein quality control by the ubiquitinproteasome system
Bag5	protein refolding

Fkbp8	protein folding chaperone
Dnajb11	unfolded protein binding
Nudcd3	unfolded protein binding
Ric8B	protein folding chaperone
Cdc37L1	unfolded protein binding
Dnaja1	protein refolding
Rpap2	perkmediated unfolded protein response
Akt1	perkmediated unfolded protein response
Eif2Ak4	endoplasmic reticulum unfolded protein response
Mesd	protein folding chaperone
Ddit3	endoplasmic reticulum unfolded protein response
Dnajc3	endoplasmic reticulum unfolded protein response
Atf3	endoplasmic reticulum unfolded protein response
Dnaja4	protein refolding
Tmem33	ire1mediated unfolded protein response
Dnajb14	misfolded protein
Hyou1	atpdependent protein folding chaperone
Erlec1	endoplasmic reticulum unfolded protein response
Hspa5	endoplasmic reticulum unfolded protein response
Stub1	misfolded protein
Tmtc4	endoplasmic reticulum unfolded protein response
Snrnp70	protein refolding
Eif2Ak2	endoplasmic reticulum unfolded protein response
Hspd1	mitochondrial unfolded protein response
Uggt1	unfolded protein binding
Cops5	ire1mediated unfolded protein response
Nudc	unfolded protein binding
Dnajc10	ire1mediated unfolded protein response
Hspa4L	unfolded protein
Os9	endoplasmic reticulum unfolded protein response
Dnajb6	unfolded protein binding
Khsrp	protein folding chaperone
Ptpn2	perkmediated unfolded protein response
Parp6	endoplasmic reticulum unfolded protein response
Ubr4	cytoplasm protein quality control by the ubiquitinproteasome system
Upf2	unfolded protein
Akt2	perkmediated unfolded protein response
Npm1	unfolded protein binding
Anp32E	protein folding chaperone

Parp16	endoplasmic reticulum unfolded protein response
Tmed2	perkmediated unfolded protein response
Grpel1	unfolded protein binding
Pfdn6	unfolded protein binding
Cul3	nuclear protein quality control by the ubiquitinproteasome system
Pdia6	ire1mediated unfolded protein response
Dnajb9	ire1mediated unfolded protein response
Serp2	endoplasmic reticulum unfolded protein response
Lyrm7	protein folding chaperone
Ccnd1	endoplasmic reticulum unfolded protein response
Hspa1B	protein refolding
Hspb2	protein refolding
Hsbp1L1	heat acclimation
Zmynd10	protein folding chaperone
Hspb1	unfolded protein
Casp12	endoplasmic reticulum unfolded protein response
Igtp	perkmediated unfolded protein response
Pacrg	unfolded protein
Bag3	unfolded protein
Hspe1-Rs1	unfolded protein binding
Hspa1A	unfolded protein
Cct6B	unfolded protein binding
Ric8A	protein folding chaperone
Pik3R1	endoplasmic reticulum unfolded protein response
Cdc123	protein folding chaperone
Eif2S1	perkmediated unfolded protein response
Pias2	endoplasmic reticulum unfolded protein response
Hsph1	unfolded protein
Tapbp	protein folding chaperone
Hspe1	unfolded protein binding
Bfar	ire1mediated unfolded protein response
Hsbp1	heat acclimation
Ric3	protein folding chaperone
Serpinh1	unfolded protein binding
Ptpn1	endoplasmic reticulum unfolded protein response
Hypk	protein folding chaperone
Dnajb1	unfolded protein binding
Faf2	unfolded protein
Cul5	endoplasmic reticulum unfolded protein response

Cdc37	unfolded protein binding
Cct2	unfolded protein binding
Ubxn4	unfolded protein
Hsp90Ab1	heat
Cct7	unfolded protein binding
Cct6A	unfolded protein binding
Dnajb2	protein refolding
Ahsa1	unfolded protein binding
Cct3	unfolded protein binding
Naca	unfolded protein binding
St13	unfolded protein binding
Creb3L4	unfolded protein
Hsf1	unfolded protein
Cct8	unfolded protein binding
Cct4	unfolded protein binding
Clpx	unfolded protein binding
Pfdn1	unfolded protein binding
Cct5	unfolded protein binding
Crebrf	endoplasmic reticulum unfolded protein response
Hsp90Aa1	unfolded protein
Hspa4	atpdependent protein folding chaperone
Tomm20	unfolded protein binding
Daxx	unfolded protein
Grpel2	unfolded protein binding
Dffa	protein folding chaperone
Cd74	protein folding chaperone
Creb3L1	endoplasmic reticulum unfolded protein response
Thbs1	unfolded protein
Hspb6	protein refolding
Ermp1	endoplasmic reticulum unfolded protein response
Mfn2	unfolded protein
Atxn3	misfolded protein
Abca7	perkmediated unfolded protein response
Syvn1	unfolded protein
Pdcl	protein refolding
Rnf126	cytoplasm protein quality control by the ubiquitinproteasome system
Sdf2L1	misfolded protein
Ficd	ire1mediated unfolded protein response
Tm7Sf3	unfolded protein

Uggt2	unfolded protein binding
Tbl2	endoplasmic reticulum unfolded protein response
Heatr3	unfolded protein binding
Herpud1	endoplasmic reticulum unfolded protein response
Tbce	unfolded protein binding
Hspa13	protein refolding
Atf6	endoplasmic reticulum unfolded protein response
Zpr1	protein folding chaperone
Amfr	endoplasmic reticulum unfolded protein response
Aplf	protein folding chaperone
Dnaja2	protein refolding
Hdac6	misfolded protein
Parp8	endoplasmic reticulum unfolded protein response
Clu	misfolded protein
Pdrg1	unfolded protein binding
Hsp90B1	unfolded protein binding
Ubr5	cytoplasm protein quality control by the ubiquitinproteasome system
Edem3	endoplasmic reticulum unfolded protein response
Creb3L2	unfolded protein
Pfdn4	unfolded protein binding
Rxra	nuclear protein quality control by the ubiquitinproteasome system
Calr	unfolded protein binding
Srsf10	unfolded protein binding
Erp44	unfolded protein
Qrich1	endoplasmic reticulum unfolded protein response
Eif2A	endoplasmic reticulum unfolded protein response
Manf	atf6mediated unfolded protein response
Gorasp2	unfolded protein
Hspa9	protein refolding
Bag6	misfolded protein binding
Pet100	unfolded protein binding
Tmbim6	unfolded protein
Ddrgk1	ire1mediated unfolded protein response
Pfdn2	unfolded protein binding
Hspa14	protein refolding
Dnajb4	unfolded protein binding
Shq1	unfolded protein binding
Nck1	perkmediated unfolded protein response
Rnf7	endoplasmic reticulum unfolded protein response

Ccdc47	protein folding chaperone
Stc2	endoplasmic reticulum unfolded protein response
Hpcal4	heat
Chac1	unfolded protein

Autophagy

Gene	Pathway
Irgm1	autophagosome assembly
Lrrk2	autophagy
Slc7A5	autophagy
Bok	autophagy
Dcn	autophagy
Usp13	autophagy
Vps25	macroautophagy
Iigp1	autophagosome assembly
Mapt	autophagy
Atg16L2	autophagosome assembly
Tigar	autophagy
Nod2	autophagy
Optn	autophagy
Sesn2	lipophagy
Lix1L	autophagosome maturation
Snx30	mitophagy
Bnip3	mitophagy
Bcl2L11	autophagy in er overload
Mid2	autophagy
Traf6	autophagosome assembly
Rab33B	autophagosome assembly
Foxk1	autophagy
Hspb8	aggrephagy
Pmp22	autophagy
Prkd1	autophagy
Mtm1	autophagosome assembly
Atg4B	aggrephagy
Myo5A	macroautophagy
Cptp	autophagy
Lepr	autophagy
Anxa7	autophagy

Rnf213	xenophagy
Zc3H12A	autophagy
Pex12	pexophagy
Bcl2	autophagy
Fez2	autophagosome assembly
Smcr8	autophagy
Srebf1	mitophagy
Vhl	autophagy
Scoc	macroautophagy
Atg4C	aggrephagy
Elp6	autophagosome maturation
Rpgr	autophagy
Kat8	autophagy
Hdac10	autophagy
Sbf2	autophagy
Ap5Z1	autophagosome assembly
Ubqln2	autophagosome assembly
Npc1	autophagy
Trim13	macroautophagy
Pex2	pexophagy
Irgm2	autophagosome assembly
Stk38L	autophagy
Sh3Glb1	autophagosome assembly
Fbxl4	autophagy of mitochondrion
Rnf185	autophagy
Prkn	autophagy
Vps51	autophagy
Rab19	autophagosome assembly
Ufl1	autophagy
Trp53	autophagy
Rab39B	autophagy
Vps16	autophagosome maturation
Tomm7	type 2 mitophagy
Poldip2	macroautophagy
Arsb	autophagy
Golga2	autophagy
Pikfyve	autophagosome assembly
C9Orf72	autophagosomelysosome fusion
Rnf166	autophagy
Yod1	macroautophagy

Mapk3	autophagosome assembly	
Epg5	autophagosome maturation	
Tollip	autophagy	
Trim21	autophagosome assembly	
Sptlc1	lipophagy	
Wdr47	autophagy	
Zkscan3	autophagy	
Ehmt2	autophagosome assembly	
Stx12	autophagosome assembly	
Pip4K2C	autophagosomelysosome fusion	
Ep300	autophagosome assembly	
Ube3C	autophagosome assembly	
Nprl2	autophagy	
Rragc	autophagosome assembly	
Scarb2	autophagy	
Vps13A	autophagy	
Tbc1D14	autophagy	
Usp33	autophagosome assembly	
Sirt2	autophagy	
Nbr1	macroautophagy	
Fnbp1L	autophagy	
Map2K1	autophagy	
Atg16L1	autophagosome assembly	
Svip	autophagy	
Arl8B	autophagosomelysosome fusion	
Dnm1L	mitophagy	
Trim23	autophagy	
Ubqln1	aggrephagy	
Hspa8	autophagy	
Mcoln1	autophagosome maturation	
Vps41	autophagy	
Zfyve26	autophagosome organization	
Xpa	autophagy	
Nipsnap2	autophagy	
Atm	autophagosome assembly	
Depdc5	autophagy	
Pjvk	pexophagy	
Pip4K2B	autophagosomelysosome fusion	
Sirt1	macroautophagy	
Lypla1	aggrephagy	

Rab33A	autophagosome assembly	
Tsc1	macroautophagy	
Vdac1	type 2 mitophagy	
Chmp2A	autophagosome maturation	
Rab12	autophagy	
Atp6V0A1	macroautophagy	
Trp53Inp1	autophagosome assembly	
Snapin	autophagosome maturation	
Kcmf1	protein targeting to vacuole involved in autophagy	
Rragb	autophagy	
Csnk2A1	aggrephagy	
Tmem39A	autophagy	
Herc1	autophagy	
Aup1	lipophagy	
Ubxn2B	autophagosome assembly	
Nprl3	autophagy	
Pik3R2	autophagy	
Xbp1	autophagy	
Atp2A2	autophagosome assembly	
Vps4A	autophagosome maturation	
Wdr45B	autophagosome assembly	
Tom1	autophagosomelysosome fusion	
Dnajc16	autophagy	
Clec16A	autophagy	
Oma1	mitophagy	
Cltc	autophagy	
Zmpste24	autophagy	
Tcirg1	autophagosome assembly	
Snx14	autophagosome maturation	
Ccny	autophagy	
Htt	aggrephagy	
Vcp	autophagosome maturation	
Diaph3	autophagosomelysosome fusion	
S100A9	autophagy	
Usp36	macroautophagy	
S100A8	autophagy	
Irf8	autophagy	
Snx7	mitophagy	
Rptor	autophagosome assembly	
Irgq	autophagy	

Trim32	autophagosome assembly
Tm9Sf1	autophagy
Cdk5Rap3	reticulophagy
Ambra1	autophagosome assembly
Trim8	autophagy
Gpr137B	autophagy
Ei24	autophagy
Ikbkg	macroautophagy
Gramd1A	autophagy
Fez1	autophagosome assembly
Atg101	autophagosome assembly
Tgfbrap1	autophagy
Pik3C2B	autophagosome organization
Capn10	autophagy of mitochondrion
Hmox1	macroautophagy
Mlst8	autophagy
Naglu	autophagy
Rab23	autophagosome assembly
Nrbf2	autophagy
Wipi2	autophagosome assembly
Vps13D	mitophagy
Mtor	autophagosome assembly
Phf23	autophagy
Prkaa2	autophagy
Pptc7	mitophagy
Gpr137	autophagy
Ubqln4	autophagy
Fbxo7	autophagy of mitochondrion
Trim12C	autophagy
Fyco1	autophagosome maturation
Vps33A	autophagosome maturation
Atp13A2	autophagosome organization
Acer2	autophagy
Retreg2	autophagy
Btrc	autophagosome assembly
Wdr45	autophagosome assembly
Wdfy3	aggrephagy
Fkbp8	mitophagy
Abl1	autophagy
Snf8	macroautophagy

Gabarapl1	autophagosome assembly	
Vps37C	macroautophagy	
Usp20	autophagy	
Wipi1	autophagosome assembly	
Cttn	mitophagy	
Rragd	autophagy	
Pik3Cb	autophagy	
Supt5	macroautophagy	
Rubcn	autophagy	
Mtmr3	autophagosome assembly	
Lrsam1	autophagy	
Akt1	autophagy	
Uvrag	autophagosome maturation	
Rab7	autophagosome assembly	
Nipsnap3B	mitophagy	
Washc1	autophagy	
Rab2A	autophagosomelysosome fusion	
Tbk1	autophagy	
Snap29	autophagosome maturation	
Nhlrc1	autophagy	
Kdm4A	autophagy	
Tpcn1	autophagy	
Nsfl1C	autophagosome assembly	
Ddit3	autophagy in er overload	
Mtcl1	autophagy	
Ulk2	autophagosome assembly	
Chmp4B	autophagosome maturation	
Dap	autophagy	
Usp7	autophagosome assembly	
Cln3	autophagosome maturation	
Stam	macroautophagy	
Stub1	chaperonemediated autophagy	
Atg3	autophagosome assembly	
Dnm2	autophagy	
Snrnp70	chaperonemediated autophagy	
Park7	autophagy	
Mul1	type 2 mitophagy	
Psen1	autophagosome assembly	
Calm3	autophagosome membrane docking	
Cisd1	autophagy	

Tmem150B	autophagy
Pacs2	autophagosome assembly
Keap1	autophagy
Chmp1B	autophagosome maturation
Vps18	autophagy
Parl	mitophagy
Ubr4	autophagy
Chmp6	autophagosome maturation
Rab3Gap1	autophagosome assembly
Ubxn6	macroautophagy
Dcaf12	autophagy
Spg11	autophagosome organization
Bnip3L	macroautophagy
Slc25A4	mitophagy
Tmem41B	autophagosome assembly
Eif4G2	autophagy
Pip4K2A	autophagosomelysosome fusion
Gsk3B	autophagy
Nrbp2	macroautophagy
Timm23	type 2 mitophagy
Tex264	autophagy
Ubxn2A	autophagosome assembly
Usp10	autophagy
Tbc1D5	autophagy
Prkaa1	autophagosome assembly
Trappc4	autophagy
Ift20	autophagosome assembly
Csnk1A1	autophagosome assembly
Rbx1	autophagosome assembly
Gabarapl2	autophagosome assembly
Itgb4	autophagy
Trem2	autophagy
Chmp4C	autophagosome maturation
Eva1A	autophagy
Gfap	chaperonemediated autophagy
Tlr2	xenophagy
Stbd1	autophagy
Inhba	autophagy
Kdr	macroautophagy
Gpsm1	macroautophagy

Plekhf1	autophagy	
Armc3	autophagy	
Ephb2	autophagosome assembly	
Ptpn22	autophagy	
Syk	autophagosome assembly	
Smo	autophagy	
Igtp	autophagosome assembly	
Rnf152	autophagy	
Rasip1	autophagy	
Ap4M1	autophagosome assembly	
Plk3	chaperonemediated autophagy	
Deptor	autophagosome assembly	
Trp53Inp2	autophagosome assembly	
Ulk1	autophagosome assembly	
Bid	autophagy in er overload	
Tpcn2	autophagy	
Mapk15	autophagy	
Hk2	type 2 mitophagy	
Ift88	autophagosome assembly	
Bag3	autophagosome assembly	
Adrb2	autophagosome maturation	
Wdr24	autophagy	
Atg10	autophagosome assembly	
Uba5	reticulophagy	
Fbxw7	mitophagy	
Wdr81	aggrephagy	
Syt11	autophagy	
Klhl22	autophagy	
Dapl1	autophagy	
Ager	autophagy	
Nod1	autophagy	
Atg7	autophagosome assembly	
Wdr41	autophagy	
Ripk2	xenophagy	
Arsa	autophagy	
Tfeb	autophagy	
Sesn3	macroautophagy	
Ptk2	autophagy	
Bcas3	autophagy	
Dapk1	autophagy	

Lyn	autophagy	
Fundc1	autophagy	
Map1Lc3A	autophagosome assembly	
Itgb1	autophagy	
Eif2S1	mitophagy	
Atg4A	aggrephagy	
Stat3	autophagy	
Vps37B	macroautophagy	
Wdfy4	autophagy	
Ormdl3	autophagy	
Acbd5	autophagy	
Lamp2	autophagosome maturation	
Ilrun	macroautophagy	
Calm2	autophagosome membrane docking	
Moap1	autophagy	
Gopc	autophagy	
Rab2B	macroautophagy	
Ube2A	mitophagy	
Stam2	macroautophagy	
Sqstm1	aggrephagy	
Hmgb1	autophagy	
Mcl1	autophagy	
Znrf1	autophagosomelysosome fusion	
Chmp3	autophagosome maturation	
Eif2Ak1	mitophagy	
Chmp5	autophagosome maturation	
Ufc1	reticulophagy	
Rraga	autophagosome assembly	
Chuk	autophagy	
Creg1	autophagy	
Tspo	mitophagy	
Cdc37	type 2 mitophagy	
Fundc2	autophagy of mitochondrion	
Rock1	autophagy	
Scfd1	autophagosome assembly	
Tecpr1	autophagosome maturation	
Arfip2	autophagy	
Cul1	autophagosome assembly	
Camkk2	autophagy of mitochondrion	
Tbc1D12	autophagosome assembly	

Map1Lc3B	autophagosome assembly	
Cisd2	autophagy	
Calm1	autophagosome membrane docking	
Atg5	aggrephagy	
Vps4B	autophagosome maturation	
Slc25A46	autophagy of mitochondrion	
Phb2	mitophagy	
Wdr6	autophagy	
Vti1A	autophagy	
Gabarap	autophagosome assembly	
Bc004004	autophagy	
Setd2	autophagosome assembly	
Rab1A	autophagosome assembly	
Atg12	autophagosome assembly	
Pex10	pexophagy	
Vamp8	autophagosome maturation	
Gnai3	macroautophagy	
Rab1B	autophagosome assembly	
Rab8A	autophagy	
Slc25A5	mitophagy	
Mtdh	autophagy	
Lamtor1	autophagosome assembly	
Cdk16	autophagy	
Vti1B	macroautophagy	
Rab43	autophagosome assembly	
Lgals8	autophagy	
Tbc1D25	autophagy	
Foxo3	autophagy	
Efnb1	autophagosome assembly	
Hif1A	macroautophagy	
Tbc1D17	autophagy	
Eif4G1	autophagy	
Rb1Cc1	autophagosome assembly	
Gusb	autophagy	
Snca	chaperonemediated autophagy	
Synpo2	autophagosome assembly	
Flcn	autophagy	
Ralb	autophagosome assembly	
Snx4	autophagosome assembly	
Epm2A	autophagosome assembly	

Tnfaip3	autophagy
Sec22B	autophagosome assembly
Plk2	autophagy
Fbxl2	autophagy
Mfn2	autophagy
Spata33	autophagy
Pik3R4	autophagosome maturation
Chmp7	autophagosome maturation
Usp30	autophagy of mitochondrion
Mfsd8	autophagosome maturation
Ticam1	autophagy
Trim65	autophagy
Smurf1	autophagy
Pycard	autophagy
Ulk3	autophagosome assembly
1600014C10Rik	autophagy
Rab3Gap2	macroautophagy
Hmga1	autophagy
Zfp418	microautophagy
Atg2B	autophagosome assembly
Rmc1	autophagy
Map3K7	autophagy
Atg2A	autophagosome assembly
Atg4D	aggrephagy
Bmf	autophagy
Pink1	autophagy
Rnf31	xenophagy
Atg14	autophagosome assembly
Vps39	autophagosomelysosome fusion
Retreg3	autophagy
Pim2	macroautophagy
Plekhm1	autophagosomelysosome fusion
Atf6	autophagy
Plekhm2	autophagosomelysosome fusion
Vps13C	type 2 mitophagy
Plaa	macroautophagy
Atg9A	autophagosome assembly
Ctsd	autophagosome assembly
Elavl1	autophagosome size
Arhgap26	mitophagy

Bcl2L13	mitophagy
Nupr1	autophagosome assembly
Larp1	macroautophagy
Ctsa	chaperonemediated autophagy
Pik3C3	autophagosome assembly
Ercc4	autophagy
Mapk8	autophagy
Qsox1	macroautophagy
Stx17	autophagosome maturation
Gsk3A	autophagy
Lrpprc	autophagy
Zranb1	autophagosome assembly
Vps11	autophagy
Hdac6	aggrephagy
Acvr2A	autophagy
Becn1	autophagosome assembly
Pdcd6Ip	macroautophagy
Ogt	mitophagy
Dram2	autophagy
Pafah1B2	macroautophagy
Clu	protein targeting to lysosome involved in chaperonemediated autophagy
Rnf41	autophagy
Sesn1	macroautophagy
Kat5	aggrephagy
Gaa	glycophagy
Pex5	pexophagy
Pik3C2A	autophagosome organization
Wac	macroautophagy
Nampt	autophagy
Retreg1	autophagy
Rps6Kb1	autophagosome assembly
Endog	autophagy
Chmp2B	autophagosome maturation
Chmp1A	autophagosome maturation
Zdhhc12	chaperonemediated autophagy
Sh3Bp4	autophagy
Ufm1	reticulophagy
Vmp1	autophagosome assembly
Rnf5	autophagy

Abi2	mitophagy
Foxk2	autophagy
Htra2	type 2 mitophagy
Wnk1	autophagy
Tsc2	macroautophagy
Snx18	autophagosome assembly
Znrf2	autophagosomelysosome fusion
Zfyve1	macroautophagy
Hap1	autophagy
Tmem208	autophagy
Rheb	autophagosome assembly
Trim27	autophagy
Mtmr9	autophagy
Huwe1	type 2 mitophagy
Lrba	mitophagy
Atg13	autophagosome assembly
Foxo1	autophagy
Tmbim6	autophagy
Ddrgk1	reticulophagy
Rab24	autophagy
Tmem59	autophagy
Ikbkb	autophagy
Nipsnap1	autophagy
Emc6	autophagosome assembly
Dele1	mitophagy
Ate1	protein targeting to vacuole involved in autophagy
Sptlc2	lipophagy
Stk11	autophagy
Srpx	autophagy
Fn1	autophagy

DNA damage response/repair

**Gene**	**Pathway**
Tex15	dna damage response
Bok	intrinsic apoptotic signaling pathway in dna damage
Plk5	dna damage response
Bcl2L12	intrinsic apoptotic signaling pathway in dna damage by p53 class mediator
Stk33	mitotic dna damage checkpoint signaling

Smarcd3	doublestrand break repair
Eepd1	dna repair
Cxcl12	intrinsic apoptotic signaling pathway in dna damage
Macrod1	dna damage response
Neil2	baseexcision repair
Yap1	dna damage response
Mapt	dna damage response
Tigar	dna damage response
Wdr76	dna damage response
Mgmt	dna alkylation repair
Kif22	dna repair
Pot1B	protection from nonhomologous end joining at telomere
Sesn2	dna damage response signal transduction by p53 class mediator
Taf9	dna repair
Foxm1	dna damage response
Palb2	dna damage response
Sprtn	dna damage response
Riox1	dna repair
Skil	intrinsic apoptotic signaling pathway in dna damage
Nabp1	dna damage response
Parp3	dna damage response
Klhl15	doublestrand break repair via homologous recombination
Ung	baseexcision repair
Brip1	dna damage response
Bcl2L11	intrinsic apoptotic signaling pathway in dna damage
Tnks1Bp1	doublestrand break repair
Actr5	dna damage response
Cdc45	doublestrand break repair via breakinduced replication
Npas2	dna damage response
Eya2	dna damage response
Ier3	dna damage response
Hic1	intrinsic apoptotic signaling pathway in dna damage
Casp9	dna damage response
Traf6	dna damage response
Xrcc2	dna damage response
Cdc14B	dna damage response
Samhd1	dna damage response
Rnaseh2B	mismatch repair
Ccar2	dna damage response
Ints7	dna damage checkpoint signaling

Rpa3	baseexcision repair
Usp22	dna repair
Iffo1	doublestrand break repair via nonhomologous end joining
Clspn	dna damage checkpoint signaling
Usp28	dna damage checkpoint signaling
Bbc3	dna damage response
Ap5S1	dna damage response
Ercc8	dna damage response
Zcwpw1	doublestrand break repair involved in meiotic recombination
Zc3H12A	dna damage response
Eme2	dna damage response
Mif	dna damage response signal transduction by p53 class mediator
Nhej1	dna damage response
Xrcc6	dna damage response
Exosc10	dna damage response
Endov	dna repair
Rpa1	baseexcision repair
Wrnip1	dna damage response
Bcl2	dna damage response
Ercc6	baseexcision repair
Ctc1	dna damage response
Tonsl	dna damage response
Lig1	baseexcision repair
Foxo4	mitotic g2 dna damage checkpoint signaling
Fbxo6	dna damage response
Ddb2	dna damage response
Brca2	dna damage response
Tdp2	dna damage response
Uba7	dna damage response
Suv39H1	dna damage response
Fancb	dna damage response
Spred1	dna damage response signal transduction by p53 class mediator
Smug1	baseexcision repair
Hdac10	dna damage response
Bach1	dna repair
Hdac3	dna repairdependent chromatin remodeling
Ap5Z1	dna damage response
Spire2	doublestrand break repair
Dclre1B	dna damage response
Pml	dna damage response signal transduction by p53 class mediator

Rad17	dna damage checkpoint signaling
Rad51C	dna damage response
Smarcal1	dna damage response
Rfc4	dna repair
Rif1	dna damage response
Prdm9	doublestrand break repair involved in meiotic recombination
Hells	doublestrand break repair
Polg	baseexcision repair
Ube2W	dna damage response
Fto	dna alkylation repair
Tank	dna damage response
Immp2L	dna damage response
Ercc2	dna damage response
Fem1B	dna damage checkpoint
Recql	dna repair
Rad9A	dna damage checkpoint signaling
Ufl1	dna damage checkpoint signaling
Trp53	dna damage response
Hmga1B	baseexcision repair
Cdk7	dna damage response
Cenps	dna damage response
Xrcc4	baseexcision repair
Wdr4	dna damage response
Shprh	dna damage response
Mcm4	doublestrand break repair via breakinduced replication
Ascc1	dna alkylation repair
Rrm1	dna repair
Eef1E1	dna damage response signal transduction by p53 class mediator
Poldip2	dna damage response
Primpol	dna damage response
Sgk1	dna damage response
Rfc5	dna repair
Cbl	dna damage response
Nme3	dna repair
Atad3A	dna damage response
Brd4	dna damage response
Mbtps1	mitotic g2 dna damage checkpoint signaling
Slf1	dna damage response
Mbtps2	mitotic g2 dna damage checkpoint signaling
Pak1	dna damage response

Actr2	doublestrand break repair via homologous recombination
Mapk3	dna damage response
Rrm2B	dna damage response
Rnf4	dna damage response
Rad21	dna damage response
Smarcad1	dna damage response
Actl6A	dna damage response
Prmt6	dna damage response
Bcl2L2	intrinsic apoptotic signaling pathway in dna damage
Usp45	dna repair
Ing3	doublestrand break repair via homologous recombination
Hpf1	dna damage response
Polg2	dna repair
Ing4	dna damage response signal transduction by p53 class mediator
Crip1	intrinsic apoptotic signaling pathway in dna damage
Trim39	mitotic g2 dna damage checkpoint signaling
Smc5	dna damage response
Vps72	doublestrand break repair via homologous recombination
Ercc1	dna damage response
Ep300	intrinsic apoptotic signaling pathway in dna damage by p53 class mediator
Gtf2H5	dna damage response
Atxn7	dna repair
Dpf2	doublestrand break repair
Xiap	dna damage response
Abraxas1	dna damage response
Sfpq	dna damage response
Usp9X	dna alkylation repair
Xab2	dna damage response
Rbbp8	dna damage response
Kat2B	dna repair
Mtch2	intrinsic apoptotic signaling pathway in dna damage
Usp1	dna damage response
Eny2	dna repair
Ube2D3	dna damage response
Cbx3	dna damage response
Xrcc5	dna damage response
Cdkn2D	dna synthesis involved in dna repair
Casp3	dna damage response
Rpl26	dna damage response signal transduction by p53 class mediator

Rad9B	dna damage checkpoint signaling
Mapk1	dna damage response
Smg1	dna damage response
Taf10	dna repair
Nfat5	dna damage response
Zfyve26	dna damage response
Xpa	baseexcision repair
Smarca2	doublestrand break repair
Ube2B	dna damage response
Phf13	dna damage response
Triap1	dna damage response signal transduction by p53 class mediator
Uimc1	dna damage response
Atm	dna damage checkpoint signaling
Grb2	signal transduction in dna damage
Slx1B	dna damage response
Hnrnpk	intrinsic apoptotic signaling pathway in dna damage by p53 class mediator
Morf4L1	dna damage response
Babam2	dna damage response
Clock	dna damage checkpoint signaling
Tmem161A	intrinsic apoptotic signaling pathway in dna damage
Sirt1	dna damage response
Pcna	baseexcision repair gapfilling
Sem1	doublestrand break repair via homologous recombination
Rad50	dna damage response
Prmt1	dna damage response
Fh1	dna damage response
Nabp2	dna damage response
Cry1	dna damage checkpoint
Csnk2A1	doublestrand break repair
Foxn3	mitotic g2 dna damage checkpoint signaling
Pias4	dna damage response
Atad5	dna damage response
Ppp4R3B	dna damage response
Ascc3	dna alkylation repair
Yju2	dna damage response signal transduction by p53 class mediator
Casp2	dna damage response
Cfap410	dna damage response
Ssrp1	dna damage response
Myo6	dna damage response signal transduction by p53 class mediator

Taok1	dna damage response
Brsk1	dna damage response
Mcrs1	dna damage response
Nsmce4A	dna damage response
Rbbp4	dna damage response
Topors	dna damage response
Zmpste24	dna damage response
Tfpt	dna damage response
Polb	baseexcision repair
Marf1	doublestrand break repair
Rps3	baseexcision repair
Commd1	nucleotideexcision repair
Taf7	dna repair
Fus	doublestrand break repair via homologous recombination
Rpa2	baseexcision repair
Otub1	dna damage response
Vcp	dna damage response
Ddx5	dna damage response signal transduction by p53 class mediator
Dclre1A	dna damage response
Dyrk1A	doublestrand break repair via homologous recombination
Mdm2	dna damage response signal transduction by p53 class mediator
Rad52	dna damage response
Oard1	dna damage response
Tpt1	intrinsic apoptotic signaling pathway in dna damage
Pttg1	dna damage response
Zfp365	doublestrand break repair via homologous recombination
Mcm6	doublestrand break repair via breakinduced replication
Egfr	dna repair
Rptor	dna damage response
Fnip2	dna damage response
Dpf1	doublestrand break repair
Sox4	dna damage response signal transduction by p53 class mediator
Hdgfl2	dna damage response
Trim32	intrinsic apoptotic signaling pathway in dna damage
Spindoc	dna damage response
Chd1L	dna damage response
Polk	dna damage response
Cdk5Rap3	mitotic g2 dna damage checkpoint signaling
Mcm8	dna damage response
Fancc	dna damage response

Shld3	dna damage response
Cep63	dna damage checkpoint signaling
Ei24	intrinsic apoptotic signaling pathway in dna damage
Ikbkg	dna damage response
Fzr1	dna damage response
Fancg	dna damage response
Banf1	dna repair
Mrnip	dna damage response
Kmt5C	dna repair
Zswim7	dna damage response
Shisa5	intrinsic apoptotic signaling pathway in dna damage by p53 class mediator
Dgkz	mitotic g1 dna damage checkpoint signaling
Slf2	dna damage response
Pole2	errorprone translesion synthesis
Cradd	dna damage response
Pold2	dna damage response
Parp1	baseexcision repair
Gnl1	dna damage response
Pold1	baseexcision repair gapfilling
Gins4	doublestrand break repair via breakinduced replication
Hmox1	intrinsic apoptotic signaling pathway in dna damage
Fen1	baseexcision repair
Rnf111	dna damage response
Rnf8	dna damage response
Taf5L	dna repair
Mgme1	dna damage response
Mlst8	dna damage response
Crebbp	doublestrand break repair via homologous recombination
Fbxo45	dna damage response
Rbbp5	dna damage response
Uaca	intrinsic apoptotic signaling pathway in dna damage
Mms22L	dna damage response
Mtor	dna damage response
Ercc5	baseexcision repair ap site formation
Polm	dna damage response
Ubqln4	dna damage response
Gins2	doublestrand break repair via breakinduced replication
Alkbh8	dna damage response
Rnf138	dna damage response

Aktip	postreplication repair
Nek1	dna damage response
Epc2	dna damage response
Hmces	dna damage response
Acer2	dna damage response
Faap20	dna damage response
Upf1	dna repair
Rbm38	dna damage response signal transduction by p53 class mediator
Setd7	dna damage response
Zbtb1	dna damage response
Sirt7	dna damage response
Ppm1D	dna damage response signal transduction by p53 class mediator
Btg2	dna damage response
Ppp1Ca	doublestrand break repair via alternative nonhomologous end joining
Rint1	mitotic g2 dna damage checkpoint signaling
Baz1B	dna damage response
Bod1L	dna damage response
Sirt6	baseexcision repair
Chchd4	mitochondrial dna repair
Smarce1	doublestrand break repair
Gtf2H1	dna damage response
Tlk2	dna damage response
Abl1	dna damage response
Smarca4	doublestrand break repair
Sde2	mitotic g1 dna damage checkpoint signaling
Top3B	dna repair
Wdr48	dna damage response
Mettl3	dna damage response
Supt16	dna damage response
Ino80	dna damage response
Rbm24	dna damage response
Ercc3	dna damage response
Ppp4R2	doublestrand break repair
Dyrk1B	dna damage response
Htatsf1	dna damage response
Rnf168	dna damage checkpoint signaling
Rexo4	dna repair
Emsy	dna damage response
Ube2E2	dna damage response

Arid1A	doublestrand break repair
Akt1	dna damage response
Uvrag	dna damage response
Eif2Ak4	dna damage checkpoint signaling
Csnk2A2	dna damage response
Topbp1	broken chromosome clustering
Nek4	dna damage response
Sfr1	dna damage response
Prkdc	dna damage response
Flywch1	dna damage response
Fancl	dna damage response
Mms19	dna damage response
Jmy	dna damage response
Cdc5L	dna damage checkpoint signaling
Rnaseh2C	mismatch repair
Stk38	dna damage checkpoint signaling
Nsd2	doublestrand break repair via nonhomologous end joining
Nop53	dna damage response
Cep164	dna damage response
Usp7	dna alkylation repair
Atrx	dna damage response
Cinp	dna damage response
Rad23A	dna damage response
Smyd2	dna damage response signal transduction by p53 class mediator
Stub1	dna damage response
Pwwp3A	dna damage response
Sod2	intrinsic apoptotic signaling pathway in dna damage
Ddx1	doublestrand break repair
Zbtb7A	dna damage response
Zdhhc16	dna damage response
Ptpn11	dna damage checkpoint signaling
Psme4	dna damage response
Park7	dna damage response
Actr8	dna damage response
Herc2	dna damage response
Syf2	mitotic g2 dna damage checkpoint signaling
Psen1	dna damage response
Pole3	dna damage response
Pnp	dna repair
Pttg1Ip	dna damage response signal transduction by p53 class mediator

Cebpg	dna repair
Chd4	doublestrand break repair via homologous recombination
Smc1A	dna damage response
Nipbl	dna damage response
Prpf19	dna damage checkpoint signaling
Eya4	dna damage response
Tfap4	dna damage response signal transduction by p53 class mediator
Tlk1	dna damage response
Uba1	dna damage response
Yy1	dna damage response
Msh2	dna damage response
Terf2	protection from nonhomologous end joining at telomere
Bax	dna damage response
Msh3	dna damage response
Tdg	baseexcision repair
Kin	dna damage response
Kdm2A	doublestrand break repair via nonhomologous end joining
Nrde2	dna damage response
Ppp4R3A	dna damage response
Gtf2H2	dna damage response
Npm1	dna repair
Taf6	dna repair
Smarcc2	doublestrand break repair
Xpc	dna damage response
Top2B	doublestrand break repair via nonhomologous end joining
Ppp1R10	mitotic dna damage checkpoint
Macrod2	dna damage response
Rbbp6	dna damage response
Ruvbl2	dna damage response
Tex264	dna damage response
Smarcc1	doublestrand break repair
Ndufs6	dna damage response signal transduction by p53 class mediator
Polr2I	transcriptioncoupled nucleotideexcision repair
Bcl7A	doublestrand break repair
Usp10	dna damage response
Rbx1	baseexcision repair ap site formation via deaminated base removal
Rad51	dna damage response
Dtx3L	dna damage checkpoint signaling
Steap3	intrinsic apoptotic signaling pathway in dna damage by p53 class mediator

Rad54L	dna damage response
Sfn	intrinsic apoptotic signaling pathway in dna damage
Cdk1	dna damage response
Chaf1A	dna damage response
Cdkn3	dna damage response
Uhrf1	dna damage response
Helb	dna damage response
Egln3	dna damage response
Ccnd1	dna damage response
Zfp668	dna repair
Gnb1L	dna damage checkpoint signaling
Zranb3	dna damage response
E2F1	dna damage checkpoint signaling
Neil1	baseexcision repair
Ndrg1	dna damage response signal transduction by p53 class mediator
Parp9	dna damage checkpoint signaling
Gen1	dna damage response
Poln	dna damage response
Ackr3	intrinsic apoptotic signaling pathway in dna damage
Fbxo5	dna damage response
Fam111A	dna damage response
Dna2	baseexcision repair
Aste1	doublestrand break repair via homologous recombination
Sp100	dna damage response signal transduction by p53 class mediator
Bcl6	dna damage response
Fbxo4	dna damage checkpoint
Ube2T	dna damage response
Wrn	baseexcision repair
Top3A	dna repair
Knl1	dna damage response
Top2A	dna damage response
Plk3	dna damage response
Ccdc13	dna damage response
Faap24	dna damage response
Mcm2	doublestrand break repair via breakinduced replication
Dclre1C	dna damage response
Cdk2	dna damage response
Bid	dna damage response
Slx4	dna damage response
Mapk15	dna damage response

Twist1	dna damage response signal transduction by p53 class mediator
Mapkapk2	dna damage response
Pclaf	dna damage response
Trip13	doublestrand break repair
Mutyh	baseexcision repair
Lig4	baseexcision repair
Smc4	single strand break repair
Mc1R	uvdamage excision repair
Ncoa6	dna damage response
Rhno1	dna damage checkpoint signaling
Tnfrsf1A	intrinsic apoptotic signaling pathway in dna damage
Tmem109	intrinsic apoptotic signaling pathway in dna damage by p53 class mediator
Ascc2	dna damage response
Map3K20	dna damage checkpoint signaling
Pola1	dna repair
Fgf10	dna repair
Fbxw7	dna damage response
Pidd1	dna damage response
Gadd45A	dna damage response
Hmgn1	pyrimidine dimer repair by nucleotideexcision repair
Hipk2	dna damage response
Ager	dna damage response
Msh6	dna damage response
Nfatc2	dna damage response
Phlda3	intrinsic apoptotic signaling pathway in dna damage by p53 class mediator
Armt1	dna damage response
Pot1A	doublestrand break repair via nonhomologous end joining
Cbx5	dna damage response
Supt20	dna repair
Hspa1A	dna repair
Elof1	dna damage response
Cip2A	broken chromosome clustering
Lyn	dna damage checkpoint signaling
Pik3R1	intrinsic apoptotic signaling pathway in dna damage
Alkbh7	dna damage response
Mtrex	dna damage response
Vrk2	dna damage response
Nsmce1	dna damage response
Mlh1	dna damage response

Mdc 1.00	dna damage response
Shld1	dna damage response
Cdk6	dna damage response
Mad2L2	dna damage response
Bcl3	dna damage response
Dcun1D5	dna damage response
Cdkn1A	dna damage response
4931406C07Rik	dna damage response
Uba6	dna damage response
Prkcd	dna damage response
Poli	dna damage response
Hinfp	dna damage checkpoint signaling
Taok3	dna damage response
Nuak1	dna damage response
Plk1	doublestrand break repair
Rfwd3	dna damage response
Mpnd	doublestrand break repair
Cdk5	dna damage response
Cetn2	dna damage response
Mpg	baseexcision repair
Moap1	intrinsic apoptotic signaling pathway in dna damage
Bad	intrinsic apoptotic signaling pathway in dna damage
Ruvbl1	dna damage response
Ino80C	dna damage response
Kat7	dna damage response
Znhit1	dna damage response signal transduction by p53 class mediator
Ube2A	dna damage response
Xrn2	dna repair
Exo5	dna damage response
Atxn7L3	dna repair
Kdm1A	dna repairdependent chromatin remodeling
Smarca5	dna damage response
Pold4	dna damage response
Nfrkb	dna damage response
Morf4L2	dna damage response
Mcts1	dna damage response
Tti1	dna damage checkpoint
Gfi1	dna damage response
Hmgb1	baseexcision repair
Wdhd1	dna repair

Smc6	dna damage response
Mcl1	dna damage response
Actb	doublestrand break repair
Cdkn2Aip	dna damage response
Msl2	dna damage response
Parg	dna damage response
Cdip1	intrinsic apoptotic signaling pathway in dna damage by p53 class mediator
Lig3	baseexcision repair
Sgf29	dna repair
Bcl2L1	intrinsic apoptotic signaling pathway in dna damage
Axin2	mismatch repair
Fmr1	dna damage response
Rad51B	dna damage response
Dgcr8	dna damage response
Zfp830	mitotic dna damage checkpoint signaling
Pms1	mismatch repair
Uchl5	dna damage response
Sf3B5	dna repair
Pnkp	dna damage response
Cd44	dna damage response signal transduction by p53 class mediator
Hus1	dna damage checkpoint signaling
Xrcc1	baseexcision repair
Vav3	dna damage response
Cib1	dna damage response
Ttc5	dna damage response
Bclaf1	dna damage response
Nedd4	dna damage response
Vcpip1	dna damage response
Yeats4	doublestrand break repair via homologous recombination
Chchd6	dna damage response
Nthl1	baseexcision repair
Actl6B	doublestrand break repair
Kmt5A	doublestrand break repair via homologous recombination
Zmat3	dna damage response
Ube2V2	dna doublestrand break processing
Faap100	dna damage response
Stxbp4	dna damage response
Pcbp4	dna damage response signal transduction by p53 class mediator
Bccip	dna damage response

Atmin	dna damage response
Phf1	dna damage response
Usp3	dna damage response
Ino80B	dna damage response
Neurl4	mitochondrial dna repair
Usp16	dna damage response
Ube2N	dna damage response
Nucks1	dna damage response
Apex1	baseexcision repair
Swi5	dna damage response
Setd2	dna damage response
Cops3	dna damage response signal transduction by p53 class mediator
Hsf1	dna damage response
Cyren	dna damage response
Pias1	dna damage response
Gigyf2	mitotic g1 dna damage checkpoint signaling
Babam1	dna damage response
Setx	dna damage response
Zfp385A	dna damage response
Rad1	dna damage checkpoint signaling
Mnat1	dna repair
Smc3	dna damage response
Dynll1	dna damage response
Cul4A	baseexcision repair ap site formation via deaminated base removal
Hltf	dna damage response
Bcl7C	doublestrand break repair
Ube2V1	errorfree postreplication dna repair
Psmd10	dna damage response signal transduction by p53 class mediator
Rmi1	doublestrand break repair via homologous recombination
Kmt5B	dna repair
Snw1	intrinsic apoptotic signaling pathway in dna damage by p53 class mediator
Cbx1	dna damage response
Mbtd1	doublestrand break repair via homologous recombination
Meaf6	doublestrand break repair via homologous recombination
Zmynd8	doublestrand break repair via homologous recombination
Apbb1	dna damage response
Foxo3	dna damage response signal transduction by p53 class mediator
Pms2	dna damage response
Dek	doublestrand break repair

Dyrk2	dna damage response
Bcl7B	doublestrand break repair
Foxp1	dna damage response
Vrk3	dna damage response
Pole	baseexcision repair gapfilling
Cd74	dna damage response signal transduction by p53 class mediator
Zgrf1	dna damage response
Mbd4	dna damage response
Mastl	dna damage response
Vrk1	dna damage response
Smc2	single strand break repair
Creb3L1	mitotic g2 dna damage checkpoint signaling
Nsmce2	dna damage response
Apex2	baseexcision repair
Fancm	dna damage response
Rpain	dna repair
Alkbh3	dna alkylation repair
Fancf	dna damage response
Senp3	doublestrand break repair via homologous recombination
Dot1L	dna damage checkpoint signaling
Ddx11	dna damage response
Eya3	dna damage response
Ccdc117	dna repair
Bak1	intrinsic apoptotic signaling pathway in dna damage
Rps6Ka6	dna damage response signal transduction by p53 class mediator
Rad18	dna damage response
Fan1	dna damage response
Paxip1	dna damage response
Men1	dna damage response
Swsap1	dna damage response
Ddit4	intrinsic apoptotic signaling pathway in dna damage by p53 class mediator
Pycard	intrinsic apoptotic signaling pathway in dna damage by p53 class mediator
Rnf126	doublestrand break repair via homologous recombination
Aen	dna damage response
Atrip	dna damage checkpoint signaling
Hmga1	baseexcision repair
Rnf169	dna damage response
Ppp4C	doublestrand break repair via homologous recombination
Sirt4	dna damage response

Map3K7	signal transduction in dna damage
Recql5	dna damage response
Paxx	dna damage response
Blm	dna damage response
Exd2	dna damage response
Ttf2	dna repair
Gtf2H3	dna damage response
Spdya	dna damage response
Tada3	dna repair
Rfc3	dna repair
Dcaf1	baseexcision repair ap site formation via deaminated base removal
Eya1	dna damage response
Polh	dna damage response
Smchd1	dna damage response
Rtel1	dna damage response
Mrgbp	doublestrand break repair via homologous recombination
Shld2	dna damage response
Mcm7	dna damage response
Kat2A	dna repair
Setmar	dna doublestrand break processing
Stk19	dna damage response
Hdac9	dna repair
Taf2	dna repair
Terf2Ip	protection from nonhomologous end joining at telomere
Supt7L	dna repair
Tdp1	dna damage response
Smarcd2	doublestrand break repair
Ogg1	baseexcision repair
Dpf3	doublestrand break repair
Plscr1	intrinsic apoptotic signaling pathway in dna damage
Donson	dna damage checkpoint signaling
Nacc2	intrinsic apoptotic signaling pathway in dna damage
Peli1	dna damage response
Mus81	dna damage response
Ep400	dna repair
Alkbh1	dna damage response
Skp2	dna doublestrand break processing
Cdc7	doublestrand break repair via breakinduced replication
Mre11A	dna damage response
Pogz	dna damage response

Nupr1	intrinsic apoptotic signaling pathway in dna damage by p53 class mediator
Rev3L	dna damage response
Trp53Bp1	dna damage checkpoint signaling
Cdk9	dna damage response
Nsmce3	dna damage response
Fbxo31	dna damage response
Taf5	dna repair
Ash2L	dna damage response
Aplf	dna damage response
Uvssa	dna damage response
Ercc4	dna damage response
Mapk8	doublestrand break repair
Poll	baseexcision repair gapfilling
Gtf2H4	dna damage response
Zbtb4	dna damage response
Trrap	doublestrand break repair via homologous recombination
Ino80D	dna damage response
Spidr	dna damage response
Epc1	doublestrand break repair via homologous recombination
Rfc2	dna repair
Chd2	dna damage response
Zbtb38	dna damage response
Nbn	dna damage checkpoint signaling
Tada1	dna repair
Helq	dna damage response
Ccnk	dna damage response
Ercc6L2	dna damage response
Mlh3	dna damage response
Trim28	dna repair
Mta1	doublestrand break repair
Inip	dna damage response
Phf10	doublestrand break repair
Clu	intrinsic apoptotic signaling pathway in dna damage
Tipin	dna damage checkpoint signaling
Fanca	dna damage response
Taf6L	dna repair
Kat5	dna damage response
Sf3B3	dna repair
Rad23B	dna damage response

Ubr5	dna damage response
Wac	dna damage response
Susd6	dna damage response
Setd1A	dna damage response
Rfc1	dna repair
Brat1	dna damage response
Zbtb40	dna damage response
Alkbh2	dna alkylation repair
Asf1A	dna damage response
Atr	dna damage checkpoint signaling
Mcm9	dna damage response
Ints3	dna damage response
Uri1	intrinsic apoptotic signaling pathway in dna damage by p53 class mediator
Endog	dna damage response
Dyrk3	dna damage response signal transduction by p53 class mediator
Cul4B	dna damage response
Wrap53	dna damage response
Smarcb1	doublestrand break repair
Mdm4	dna damage response signal transduction by p53 class mediator
Ccng1	mitotic g2 dna damage checkpoint signaling
Trip12	dna damage response
Spire1	doublestrand break repair
Nudt16L1	doublestrand break repair via nonhomologous end joining
Pold3	dna damage response
Taok2	dna damage response
Aptx	dna damage response
Arid2	doublestrand break repair
Htra2	intrinsic apoptotic signaling pathway in dna damage
Etaa1	dna damage response
Dhx9	dna repair
Usp47	baseexcision repair
Brcc3	dna damage response
Parp2	baseexcision repair
Rps27L	dna damage response signal transduction by p53 class mediator
Ddb1	baseexcision repair ap site formation via deaminated base removal
D7Ertd443E	dna damage response
Ppp2R5C	dna damage response signal transduction by p53 class mediator
Fbh1	dna damage response
Taf12	dna repair

C1Qbp	dna damage response
Huwe1	baseexcision repair
Rnaseh2A	mismatch repair
Rev1	dna damage response
Atf2	dna damage response
Acd	protection from nonhomologous end joining at telomere
Fam168A	baseexcision repair
Morc2A	dna damage response
Mapk14	dna damage checkpoint signaling
Foxo1	dna damage response
Hipk1	intrinsic apoptotic signaling pathway in dna damage by p53 class mediator
Apc	dna damage response
Tiprl	dna damage checkpoint signaling
Rad51D	dna damage response
Bag6	intrinsic apoptotic signaling pathway in dna damage by p53 class mediator
Otud4	dna alkylation repair
Brd8	doublestrand break repair via homologous recombination
Brd7	doublestrand break repair
Pbrm1	doublestrand break repair
Psmd14	dna damage response
Nono	dna damage response
Dmap1	dna repair
Cenpx	dna damage response
Dele1	dna damage response
Senp2	dna damage response signal transduction by p53 class mediator
Rassf1	dna damage response
Smarcd1	doublestrand break repair
Cbx8	dna repair
Stk11	dna damage response
Spred2	dna damage response signal transduction by p53 class mediator
Brca1	dna damage response
Fancd2	dna damage response
Bcl2A1B	intrinsic apoptotic signaling pathway in dna damage

Oxidative stress response

Gene	Pathway
Cox7A1	mitochondrial electron transport cytochrome c to oxygen

Lrrk2	oxidative stress
Mt3	oxidative stress
Slc7A11	oxidative stress
Ppargc1A	oxidative stress
Cyp1B1	hydrogen peroxide
Trim25	oxidative stress
Sesn2	oxidative stress
Aifm2	oxidative stress
Anxa1	hydrogen peroxide
Bnip3	hydrogen peroxide
Aqp1	hydrogen peroxide
Cygb	removal of superoxide radicals
Slc11A2	oxidative stress
Gpx7	oxidative stress
Glrx	hydrogen peroxidemediated programmed cell death
Prkd1	hydroperoxide
Zfp580	hydrogen peroxide
Nqo1	hydrogen peroxide
Zc3H12A	oxidative stress
Pex12	reactive oxygen species
Foxo4	oxidative stress
Mapk7	hydrogen peroxide
Pml	translation in oxidative stress
Selenos	oxidative stress
Pex2	reactive oxygen species
Iscu	mitochondrial electron transport nadh to ubiquinone
Prdx3	oxidative stress
Sirpa	hydrogen peroxide
Prdx1	removal of superoxide radicals
Prkn	oxidative stress
Trap1	intrinsic apoptotic signaling pathway in hydrogen peroxide
Trp53	reactive oxygen species
Nfe2L2	hydrogen peroxide
Mt-Nd3	mitochondrial electron transport nadh to ubiquinone
Cpe	hydrogen peroxide
Ppif	hydrogen peroxide
Net1	hydrogen peroxide
Nos3	removal of superoxide radicals
Sdhd	mitochondrial electron transport succinate to ubiquinone
Fyn	hydrogen peroxide

Mapk9	reactive oxygen species
Ep300	hydrogen peroxidemediated programmed cell death
Apoa4	removal of superoxide radicals
Pex13	reactive oxygen species
Map2K4	intrinsic apoptotic signaling pathway in hydrogen peroxide
Kat2B	oxidative stress
Mtch2	mitochondrial atp synthesis coupled electron transport
Sirt2	oxidative stress
Sod1	oxidative stress
Mt-Co3	mitochondrial electron transport cytochrome c to oxygen
Arl6Ip5	oxidative stress
Ndufaf1	mitochondrial electron transport nadh to ubiquinone
Pyroxd1	oxidative stress
Hspa8	hydrogen peroxide
Cox7C	mitochondrial electron transport cytochrome c to oxygen
Atm	reactive oxygen species
Smpd3	hydrogen peroxide
Pjvk	programmed cell death in reactive oxygen species
Tmem161A	oxidative stress
Sirt1	hydrogen peroxide
Pnpla8	oxidative stress
Tsc1	oxidative stress
Ncoa7	oxidative stress
Prdx2	oxidative stress
Zfp277	hydrogen peroxide
Stk25	oxidative stress
Pcna	hydrogen peroxide
Trp53Inp1	hydroperoxide
Uqcrc2	mitochondrial electron transport ubiquinol to cytochrome c
Uqcrb	mitochondrial electron transport ubiquinol to cytochrome c
Sdhc	mitochondrial electron transport succinate to ubiquinone
Ndufs8	mitochondrial electron transport nadh to ubiquinone
Mt-Cytb	mitochondrial electron transport ubiquinol to cytochrome c
Pex14	reactive oxygen species
Xbp1	oxidative stress
Atp2A2	oxidative stress
Ndufv3	mitochondrial atp synthesis coupled electron transport
Ndufa12	mitochondrial atp synthesis coupled electron transport
Ndufa7	mitochondrial electron transport nadh to ubiquinone
Atf4	oxidative stress

Uqcr10	mitochondrial electron transport ubiquinol to cytochrome c
Cyc1	mitochondrial electron transport ubiquinol to cytochrome c
Uqcrh	mitochondrial electron transport ubiquinol to cytochrome c
Rps3	hydrogen peroxide
Cycs	mitochondrial electron transport cytochrome c to oxygen
Uqcr11	mitochondrial electron transport ubiquinol to cytochrome c
Cox5B	mitochondrial electron transport cytochrome c to oxygen
Ndufa11	mitochondrial electron transport nadh to ubiquinone
Mdm2	hydrogen peroxide
Rad52	oxidative stress
Uqcrq	mitochondrial electron transport ubiquinol to cytochrome c
Rwdd1	oxidative stress
Slc8A1	reactive oxygen species
Slc25A24	oxidative stress
Myb	hydrogen peroxide
Ptprk	reactive oxygen species
Rhob	hydrogen peroxide
Fancc	oxidative stress
Abcd1	oxidative stress
Lcn2	hydrogen peroxide
Parp1	oxidative stress
Prdx5	oxidative stress
Ern 1.00	hydrogen peroxide
Naglu	oxidative stress
Cat	detoxification of hydrogen peroxide
Bmp4	oxidative stress
Prkaa2	oxidative stress
Gpx1	oxidative stress
Sdhaf2	mitochondrial electron transport succinate to ubiquinone
Stx4A	oxidative stress
Pdk2	reactive oxygen species
Atp13A2	oxidative stress
Fer	reactive oxygen species
Mapkap1	oxidative stress
Sirt6	hydrogen peroxide
Map3K5	hydrogen peroxide
Ndufa8	mitochondrial electron transport nadh to ubiquinone
Chchd4	oxidative stress
Ndufa10	mitochondrial electron transport nadh to ubiquinone
Abl1	hydrogen peroxide

Kdm6B	hydrogen peroxide
Stau2	oxidative stress
Uqcc3	mitochondrial electron transport ubiquinol to cytochrome c
Akt1	hydrogen peroxideinduced neuron intrinsic apoptotic signaling pathway
Dnajc15	mitochondrial electron transport nadh to ubiquinone
Ndufv1	mitochondrial atp synthesis coupled electron transport
Stx2	oxidative stress
Ndufb9	mitochondrial electron transport nadh to ubiquinone
Sod2	oxidative stress
Mt-Nd2	mitochondrial electron transport nadh to ubiquinone
Park7	hydrogen peroxide
Cox4I1	mitochondrial electron transport cytochrome c to oxygen
Ndufb8	mitochondrial electron transport nadh to ubiquinone
Keap1	oxidative stress
Coq9	mitochondrial electron transport nadh to ubiquinone
Rnf146	hydrogen peroxide
Lonp1	oxidative stress
Fut8	oxidative stress
Cox5A	mitochondrial electron transport cytochrome c to oxygen
Dguok	mitochondrial atp synthesis coupled electron transport
Top2B	hydrogen peroxide
Vkorc1L1	oxidative stress
Ndufs6	mitochondrial electron transport nadh to ubiquinone
Prkaa1	hydrogen peroxide
Ccs	removal of superoxide radicals
Cul3	oxidative stress
Rbx1	oxidative stress
Fbln5	removal of superoxide radicals
Prr5L	oxidative stress
Cox4I2	mitochondrial electron transport cytochrome c to oxygen
Mpo	removal of superoxide radicals
Sod3	removal of superoxide radicals
Gch1	removal of superoxide radicals
Mmp2	reactive oxygen species
Gpx8	oxidative stress
Stk26	oxidative stress
Cdk1	hydrogen peroxide
Sphk1	hydrogen peroxide
Met	hydrogen peroxidemediated programmed cell death

Ednra	hydrogen peroxide
Pdgfd	hydrogen peroxide
Hspb1	oxidative stress
Fos	reactive oxygen species
Slc4A11	oxidative stress
Capn1	hydrogen peroxide
Msra	oxidative stress
Bid	mitochondrial atp synthesis coupled electron transport
Axl	hydrogen peroxide
Scly	oxidative stress
Klf4	hydrogen peroxide
Gpr37	reactive oxygen species
Klf2	hydrogen peroxide
Atg7	reactive oxygen species
Pdgfrb	hydrogen peroxide
Plekha1	hydrogen peroxide
Src	hydrogen peroxide
Cox6A2	mitochondrial electron transport cytochrome c to oxygen
Dapk1	hydroperoxide
Gsr	oxidative stress
Vrk2	oxidative stress
Map1Lc3A	hydrogen peroxide
Eif2S1	oxidative stress
Pawr	hydrogen peroxidemediated programmed cell death
Cst3	hydrogen peroxide
Nr4A2	oxidative stress
Tet1	reactive oxygen species
Slc25A14	oxidative stress
Ankzf1	hydrogen peroxide
Prkcd	hydrogen peroxide
Hsph1	intrinsic apoptotic signaling pathway in hydrogen peroxide
Aif1	oxidative stress
Ect2	hydrogen peroxide
Sin3A	tertbutyl hydroperoxide
Pam	oxidative stress
Romo1	reactive oxygen species
Hdac2	hydrogen peroxide
Fads2	oxidative stress
Cul1	oxidative stress
Rack1	intrinsic apoptotic signaling pathway in hydrogen peroxide

Abl2	oxidative stress
Mt-Nd6	mitochondrial electron transport nadh to ubiquinone
Apex1	hydrogen peroxide
Pycr2	oxidative stress
Hsf1	hydrogen peroxide
Pex10	reactive oxygen species
Chchd2	oxidative stress
Ndufv2	mitochondrial electron transport nadh to ubiquinone
Setx	hydrogen peroxide
Cfl1	hydrogen peroxide
Cox7A2L	mitochondrial electron transport cytochrome c to oxygen
Oxr1	hydroperoxide
Bdnf	mitochondrial electron transport nadh to ubiquinone
Foxo3	oxidative stress
Prkra	oxidative stress
Hif1A	oxidative stress
Sdhb	mitochondrial electron transport succinate to ubiquinone
Ndufs2	mitochondrial atp synthesis coupled electron transport
Foxp1	hydrogen peroxidemediated programmed cell death
Snca	oxidative stress
Agap3	reactive oxygen species
Dhfr	removal of superoxide radicals
Tnfaip3	hydrogen peroxide
Car3	intrinsic apoptotic signaling pathway in hydrogen peroxide
Cox8B	mitochondrial electron transport cytochrome c to oxygen
Ermp1	oxidative stress
Aldh3B1	oxidative stress
Etv5	oxidative stress
Pdgfra	reactive oxygen species
Mapk13	hydrogen peroxide
Tbc1D24	oxidative stress
Selenon	oxidative stress
Pink1	oxidative stress
Coq7	oxidative stress
Dld	mitochondrial electron transport nadh to ubiquinone
Fxn	hydrogen peroxide
Mgst1	lipid hydroperoxide
Meak7	oxidative stress
Ogg1	reactive oxygen species
Ndufs1	mitochondrial electron transport nadh to ubiquinone

Stk24	oxidative stress
Oser1	hydrogen peroxide
Ripk1	hydrogen peroxide
Fbxo31	oxidative stress
Atp7A	removal of superoxide radicals
Mapk8	hydrogen peroxide
Pdcd10	intrinsic apoptotic signaling pathway in hydrogen peroxide
G6Pdx	oxidative stress
Pcgf2	hydrogen peroxide
Hdac6	hydrogen peroxide
Becn1	hydrogen peroxide
Ercc6L2	reactive oxygen species
Psap	reactive oxygen species
Rela	hydrogen peroxide
Pex5	reactive oxygen species
Uqcrc1	mitochondrial electron transport ubiquinol to cytochrome c
Ezh2	hydrogen peroxide
Ppia	oxidative stress
Endog	oxidative stress
Rbfox2	hydrogen peroxide
Pnpt1	oxidative stress
Ppp5C	hydrogen peroxide
Cox6A1	mitochondrial electron transport cytochrome c to oxygen
Ndufb6	mitochondrial atp synthesis coupled electron transport
Ddr2	hydrogen peroxidemediated programmed cell death
Arnt	oxidative stress
Mpv17	reactive oxygen species
Htra2	oxidative stress
Sdha	mitochondrial electron transport succinate to ubiquinone
Cox7B	mitochondrial electron transport cytochrome c to oxygen
Mt-Nd1	mitochondrial electron transport nadh to ubiquinone
Txn1	detoxification of hydrogen peroxide
Brf2	oxidative stress
Atf2	oxidative stress
Mt-Nd5	mitochondrial electron transport nadh to ubiquinone
Foxo1	hydrogen peroxide
Stat6	hydrogen peroxide
Ndufc2	mitochondrial electron transport nadh to ubiquinone
Uqcrfs1	mitochondrial electron transport ubiquinol to cytochrome c
Cox7A2	mitochondrial electron transport cytochrome c to oxygen

Ndufs7	mitochondrial electron transport nadh to ubiquinone
Mt-Nd4	mitochondrial electron transport nadh to ubiquinone
Cox8A	mitochondrial electron transport cytochrome c to oxygen
Cbx8	hydrogen peroxide
Srxn1	oxidative stress
Ndufs3	mitochondrial electron transport nadh to ubiquinone
Wnt16	oxidative stressinduced premature senescence
Ccnb1	mitochondrial atp synthesis coupled electron transport
Mgat3	oxidative stress
Trpm2	hydrogen peroxide
Penk	oxidative stress
Fancd2	oxidative stress
Rbm11	oxidative stress

Intercellular communication and cell adhesion

Gene	Pathway
Spp1	integrin binding
Hopx	gap junction assembly
Tnr	integrin binding
Icam4	integrin binding
Apod	focal adhesion assembly
Panx1	gap junction
Cxcl12	integrin binding
Ppm1F	focal adhesion assembly
Itgb3	cellsubstrate junction assembly
Dchs1	cadherin binding
Yap1	bitight junction
Mxra8	bitight junction
Cav1	gap junction assembly
Coro1C	focal adhesion assembly
Prtn3	cellcell junction maintenance
Enpp2	focal adhesion assembly
Jam2	bitight junction
Cldn11	bitight junction
Cyth3	bitight junction
Cldn4	bitight junction
Cd46	cadherin binding
Ccn2	integrin binding

Itgb7	integrin binding
Ptprz1	integrin binding
Ramp2	adherens junction assembly
Cdh4	cellcell junction assembly
Igf2	integrin binding
Nrp1	focal adhesion assembly
Lin7B	bitight junction
Zfp703	adherens junction assembly
Cldn8	bitight junction
Lims1	focal adhesion assembly
Arvcf	cadherin binding
Cdh18	cellcell junction assembly
Synpo	bitight junction
Pmp22	bitight junction
Map3K1	tight junction
Vegfa	focal adhesion assembly
Clmp	bitight junction
Traf4	bitight junction
Jam3	bitight junction
Micall2	bitight junction
Col16A1	focal adhesion assembly
Anxa7	integrin binding
Cdh6	cellcell junction assembly
Rap2B	bitight junction
Itgb2L	integrin binding
Cntnap2	cell junction assembly
Bcl2	focal adhesion assembly
Cldn6	bitight junction
Cdk5R1	cadherin binding
Srf	bitight junction assembly
Vcl	adherens junction assembly
Ptprm	cadherin binding
Gjb2	connexin complex
Itgav	focal adhesion assembly
Gjb6	connexin complex
Usp53	bitight junction
Tesk2	focal adhesion assembly
Dusp3	focal adhesion assembly
Clasp2	focal adhesion assembly
Prkaca	bitight junction assembly

Poldip2	focal adhesion assembly
Wwtr1	bitight junction
Cldn7	bitight junction
Nos3	cadherin binding
Wdpcp	focal adhesion assembly
Dapk3	focal adhesion assembly
Cdh23	cadherin binding
Plpp3	integrin binding
Frmd4B	bitight junction
Wnk3	bitight junction
Sorbs1	focal adhesion assembly
Vapa	bitight junction
Epb41L4B	bitight junction
Cyth1	bitight junction
Mtss1	adherens junction maintenance
Pip5K1A	focal adhesion assembly
Rhoa	apical junction assembly
Cdc42	cell junction assembly
Acvr1	cadherin binding
Rock2	bitight junction assembly
Pten	focal adhesion assembly
Lin7C	bitight junction
Prkcz	bitight junction
Strn	bitight junction
Specc1L	gap junction
P4Hb	integrin binding
Capza1	cell junction assembly
Crb3	bitight junction
Tjp3	bitight junction
Clasp1	focal adhesion assembly
Wnt4	focal adhesion assembly
Frmd5	integrin binding
Ccn4	integrin binding
Lcp1	integrin binding
Egfr	integrin binding
Fermt3	integrin binding
Nphp4	bitight junction
Ptprk	focal adhesion assembly
Ptk2B	focal adhesion assembly
Igf1	integrin binding

Ptprj	focal adhesion assembly
Lama5	integrin binding
Pkp3	desmosome assembly
Cldn23	bitight junction
Cdh24	cellcell junction assembly
Actn1	focal adhesion assembly
Gpnmb	integrin binding
Sympk	bitight junction
Pard3B	bitight junction
Calb2	gap junction
Sgsm3	gap junction
Tbc1D2	cadherin binding
Neo1	cadherin binding
Ubn1	bitight junction
Arhgap17	bitight junction
Pkp2	adherens junction maintenance
Tspan4	integrin binding
Plec	hemidesmosome assembly
Rap2C	bitight junction
Cdk4	bitight junction
Dusp22	focal adhesion assembly
Hdac7	cellcell junction assembly
Tjp2	bitight junction
Rpgrip1L	bitight junction
Ocln	bitight junction
Dst	hemidesmosome assembly
Nf2	integrin binding
Fkrp	filtration diaphragm assembly
Adam15	integrin binding
Shroom2	cellcell junction maintenance
Afdn	adherens junction maintenance
Myh9	focal adhesion assembly
Cib2	integrin binding
Smad7	adherens junction assembly
Whamm	focal adhesion assembly
Ctnnal1	cadherin binding
Fer	adherens junction assembly
Prkci	bitight junction
Ppp1Ca	cadherin binding involved in cellcell adhesion

Ctnna1	apical junction assembly
Mpdz	bitight junction
Adam17	integrin binding
Abl1	focal adhesion assembly
Cldn14	bitight junction
Cttn	focal adhesion assembly
Dlg5	zonula adherens assembly
Coro2B	focal adhesion assembly
Slk	focal adhesion assembly
Ccn1	integrin binding
Ash1L	bitight junction
Pard3	bitight junction
Cdh15	cellcell junction assembly
Tln1	cell junction assembly
Mycbp2	synaptic assembly at neuromuscular junction
Magi1	bitight junction
Rapgef1	cell junction assembly
Nphp1	bitight junction
Plekha7	zonula adherens maintenance
Tjap1	bitight junction
Fbf1	apical junction assembly
Ptpn11	cadherin binding
Ilk	integrin binding
Rab40C	focal adhesion assembly
Psen1	cadherin binding
Actn4	bitight junction assembly
Camsap3	zonula adherens maintenance
Ptpn2	integrin binding
Arhgef2	bitight junction
App	synaptic assembly at neuromuscular junction
Cxadr	bitight junction
Pkn2	apical junction assembly
Gsk3B	integrin binding
Rtn4	cadherin binding
Nisch	integrin binding
Gjd2	connexin complex
Itgb1Bp1	focal adhesion assembly
Ctnnd2	cadherin binding
Itgb4	hemidesmosome assembly

Fbln5	integrin binding
Sema7A	integrin binding
Cdh5	bitight junction
Cldn10	bitight junction
Fermt1	integrin binding
Lin7A	bitight junction
Dbn1	gap junction
Tspan8	integrin binding
Fbln1	integrin binding
Arhgap6	focal adhesion assembly
Itgb5	integrin binding
Gfap	integrin binding
Cldn22	bitight junction
L1Cam	integrin binding
Isg15	integrin binding
Eppk1	bitight junction
Vcam1	integrin binding
Fscn1	cellcell junction assembly
S1Pr3	integrin binding
S1Pr2	integrin binding
Wnk4	bitight junction
Mfge8	integrin binding
Kdr	focal adhesion assembly
Itga9	integrin binding involved in cellmatrix adhesion
Il1B	integrin binding
Cdh13	cellcell junction assembly
Cyth4	bitight junction
Nrg1	integrin binding
Ccnd1	bitight junction
Col3A1	integrin binding
Fgf2	integrin binding
Flot1	cell junction assembly
Cx3Cl1	integrin binding
Atp7B	bitight junction
Vtn	integrin binding
Pecam1	bitight junction assembly
Mmp14	focal adhesion assembly
Ibsp	integrin binding
Itgb2	integrin binding

Cldn3	cellcell junction maintenance
Gjc1	connexin complex
Ndrg1	cadherin binding
Cdh11	cellcell junction assembly
Fxyd5	cadherin binding
Emp2	integrin binding
Dab2	integrin binding
Ephb2	tight junction assembly
Cd177	cellcell junction maintenance
Cd9	integrin binding
Aloxe3	bitight junction assembly
Emilin1	integrin binding involved in cellmatrix adhesion
Amot	bitight junction
Syk	integrin binding
Itgb8	integrin binding
Ptprt	cadherin binding
Acvrl1	bitight junction assembly
Csf1R	cellcell junction maintenance
Cdh1	bitight junction assembly
Igsf5	bitight junction
S100A10	focal adhesion assembly
Inava	adherens junction maintenance
Rhod	focal adhesion assembly
Lamb2	integrin binding
Marveld2	bitight junction
Lamb1	integrin binding
Fmn1	focal adhesion assembly
Mpz	cellcell junction maintenance
Pdia4	integrin binding
Mapk15	bitight junction
Tnc	integrin binding
Prkch	bitight junction assembly
Luzp1	bitight junction
Ctnnd1	bitight junction
Grhl2	bitight junction assembly
Frmd4A	bitight junction
Itga6	integrin binding
Amotl1	bitight junction
Whrn	paranodal junction maintenance

Myo9A	cell junction assembly
Dlg3	bitight junction
F11R	bitight junction
Epb41L5	focal adhesion assembly
Ptk2	focal adhesion assembly
Src	focal adhesion assembly
Cldnd1	bitight junction
Lyn	integrin binding
Fermt2	adherens junction maintenance
Itgb1	bitight junction assembly
Npnt	integrin binding
Rhoc	apical junction assembly
Dmtn	focal adhesion assembly
Ctnna2	cadherin binding
Ptn	integrin binding
Rac1	bitight junction assembly
Rap1A	cell junction assembly
Tln2	integrin binding
Magi2	bitight junction
Fam107A	focal adhesion assembly
Cdh2	cellcell junction assembly
Ect2	bitight junction
Arl 2.00	bitight junction assembly
Hmgb1	integrin binding
Actb	tight junction
Epcam	bitight junction
Pard6G	bitight junction
Ank3	bitight junction
Rab13	bitight junction
Cul5	focal adhesion assembly
Iqgap1	focal adhesion assembly
Tbcd	bitight junction
Actg1	focal adhesion assembly
Marveld3	bitight junction
Tjp1	adherens junction maintenance
Rock1	bitight junction assembly
Limch1	focal adhesion assembly
Pkp4	cellcell junction assembly
Peak1	focal adhesion assembly
Tgfbr1	bitight junction

Map4K4	focal adhesion assembly
Myo1C	bitight junction assembly
Cd81	integrin binding
Cldn9	bitight junction
Pak2	adherens junction assembly
Cldn12	bitight junction
Timp2	integrin binding
Magi3	bitight junction
Rcc2	focal adhesion assembly
Cyth2	bitight junction
Sdc4	focal adhesion assembly
Cfl1	focal adhesion assembly
Ocel1	bitight junction
Mtdh	bitight junction
Jup	desmosome assembly
Kifc3	zonula adherens maintenance
Pard6A	cellcell junction maintenance
Lgals8	integrin binding
Macf1	focal adhesion assembly
Cd2Ap	cadherin binding
Vasp	bitight junction
Mmp24	cadherin binding
Phldb2	focal adhesion assembly
Flcn	cellcell junction assembly
Tns1	cellsubstrate junction assembly
Fgf1	integrin binding
Dlc1	focal adhesion assembly
Ccdc85C	bitight junction
Thbs1	integrin binding
Adam9	integrin binding
Pxn	focal adhesion assembly
Sh3Bp1	bitight junction
Gpm6B	focal adhesion assembly
Smad3	focal adhesion assembly
P2Rx4	cadherin binding
Nfasc	septate junction
Lrp12	integrin binding
Cgnl1	bitight junction
Cgn	bitight junction
Gnpat	paranodal junction assembly

Rhpn1	focal adhesion assembly
Ankrd28	focal adhesion assembly
Patj	bitight junction
Prkca	desmosome assembly
Bcr	focal adhesion assembly
Lsr	bitight junction
Pdcd6Ip	bitight junction
Slc39A9	bitight junction assembly
Ildr1	bitight junction
Ctnnb1	bitight junction
F2R	cellcell junction maintenance
Rapgef2	bitight junction
Utrn	integrin binding
Lamc1	hemidesmosome assembly
Bmpr2	cadherin binding
Ppia	integrin binding
Wdr1	apical junction assembly
Numb	cadherin binding
Mpp7	bitight junction
Dmd	integrin binding
Amotl2	bitight junction
Calr	integrin binding
Vmp1	cell junction assembly
Epb41L3	paranodal junction maintenance
Taok2	focal adhesion assembly
Abi2	zonula adherens assembly
Anp32A	integrin binding
Pdpk1	focal adhesion assembly
Dlg1	bitight junction
Sipa1L3	tritight junction
Ybx3	gap junction
Itga3	integrin binding
Gfra1	integrin binding
Hipk1	adherens junction assembly
Rap1B	cell junction assembly
Apc	bitight junction
Ophn1	cell junction assembly
Adam8	integrin binding
Cd151	integrin binding

Pdzd11	synaptic assembly at neuromuscular junction
Pard6B	bitight junction
Rnf7	focal adhesion assembly
Ccn3	gap junction
Pof1B	bitight junction
Olfm4	cadherin binding
Fbn1	integrin binding
Des	gap junction
Fn1	cellsubstrate junction assembly
Itgbl1	integrin binding
Ildr2	bitight junction
Fzd5	bitight junction

Epigenetics

**Gene**	**Pathway**
Mycn	autosome genomic imprinting
Tex15	dna methylationdependent constitutive heterochromatin formation
Smarcd3	nucleosome disassembly
Nap1L2	nucleosome assembly
Bahd1	heterochromatin formation
Loxl2	heterochromatin organization
Ftx	gene expression via chromosomal cpg island demethylation
Apobec3	transposable element silencing
Pcgf5	random inactivation of x chromosome
Phf19	epigenetic gene expression
Nap1L5	nucleosome assembly
Zcwpw1	epigenetic gene expression
Exosc10	random inactivation of x chromosome
Oip5	pericentric heterochromatin organization
Hat1	subtelomeric heterochromatin formation
Rrp8	rdna heterochromatin formation
Suv39H1	epigenetic programming in the zygotic pronuclei
Kat8	transcription initiationcoupled chromatin remodeling
Hdac3	epigenetic gene expression
Rif1	gene expression epigenetic
Hells	dna methylationdependent constitutive heterochromatin formation
Trp53	transcription initiationcoupled chromatin remodeling

Setdb2	heterochromatin organization
Tsix	transcription initiationcoupled chromatin remodeling
Kdm1B	transcription initiationcoupled chromatin remodeling
Shprh	nucleosome assembly
Rnf2	epigenetic gene expression
Xist	inactivation of paternal x chromosome by genomic imprinting
Smyd3	nucleosome assembly
Jpx	random inactivation of x chromosome
Rbm15B	dosage compensation by inactivation of x chromosome
Mta2	genomic imprinting
Tspyl1	nucleosome assembly
Smarcad1	heterochromatin formation
Mecp2	epigenetic gene expression
Ing3	chromatin organization
Ehmt2	dna methylationdependent constitutive heterochromatin formation
Vps72	transcription initiationcoupled chromatin remodeling
Dubr	gene expression via chromosomal cpg island methylation
Ep300	transcription initiationcoupled chromatin remodeling
Upf3B	random inactivation of x chromosome
Pik3Ca	autosome genomic imprinting
Sirt2	epigenetic gene expression
Cbx3	heterochromatin formation
Tasor	constitutive heterochromatin formation
Mllt3	chromatin organization
Smarca2	nucleosome assembly
Glmn	epigenetic gene expression
Hnrnpk	random inactivation of x chromosome
Sirt1	heterochromatin formation
Zfp445	epigenetic programing of female pronucleus
Dnajc9	nucleosome assembly
H3F3B	pericentric heterochromatin formation
Ssrp1	nucleosome assembly
Tspyl4	nucleosome assembly
Bmi1	dna methylationdependent constitutive heterochromatin formation
N6Amt1	transcription initiationcoupled chromatin remodeling
Prmt3	transcription initiationcoupled chromatin remodeling
Rbbp4	nucleosome assembly
H1F0	nucleosome assembly

Atf7Ip	dna methylationdependent constitutive heterochromatin formation
Zmpste24	gene expression via chromosomal cpg island demethylation
Tut4	transposable element silencing by mrna destabilization
Dyrk1A	heterochromatin formation
Hnrnpu	chromatin organization
Setd5	chromatin organization
Ing2	gene expression epigenetic
Egr1	gene expression via chromosomal cpg island demethylation
Lmnb1	heterochromatin formation
Tspyl5	nucleosome assembly
Apobec2	gene expression via chromosomal cpg island demethylation
Tspyl2	nucleosome assembly
Spen	random inactivation of x chromosome
Ubn1	nucleosome assembly
Tet3	gene expression via chromosomal cpg island demethylation
Sart3	nucleosome assembly
Ndn	genomic imprinting
Gnas	genomic imprinting
Cdyl	random inactivation of x chromosome
Rnf8	epigenetic gene expression
Prmt7	genomic imprinting
Hdac7	epigenetic gene expression
Spty2D1	nucleosome assembly
Mettl23	epigenetic programing of male pronucleus
Mthfr	heterochromatin organization
Hdac11	epigenetic gene expression
Nap1L4	nucleosome assembly
Rbbp5	transcription initiationcoupled chromatin remodeling
Arid4B	genomic imprinting
Gpx1	epigenetic gene expression
Kmt2B	dna methylationdependent constitutive heterochromatin formation
Kmt2A	transcription initiationcoupled chromatin remodeling
Phf2	transcription initiationcoupled chromatin remodeling
Padi2	transcription initiationcoupled chromatin remodeling
Dnmt3A	autosome genomic imprinting
L3Mbtl3	transcription initiationcoupled chromatin remodeling
Smyd5	gene expression epigenetic
Upf1	random inactivation of x chromosome
Setd7	heterochromatin organization

Sirt7	transcription initiationcoupled chromatin remodeling
Ppm1D	dna methylationdependent constitutive heterochromatin formation
Mbd3	dna methylationdependent constitutive heterochromatin formation
Chrac1	nucleosome assembly
Sirt6	gene expression epigenetic
Hdac1	dna methylationdependent constitutive heterochromatin formation
Smarce1	nucleosome disassembly
Mphosph8	constitutive heterochromatin formation
Tlk2	chromatin organization
Smarca4	transcription initiationcoupled chromatin remodeling
Mettl3	dosage compensation by inactivation of x chromosome
Supt16	nucleosome assembly
Rnf168	epigenetic gene expression
Anp32B	nucleosome assembly
Arid1A	nucleosome disassembly
Mtf2	epigenetic gene expression
Ubtf	rdna heterochromatin formation
Nr3C1	transcription initiationcoupled chromatin remodeling
Rlim	random inactivation of x chromosome
Wbp2	transcription initiationcoupled chromatin remodeling
Supt6	nucleosome organization
Ncor2	random inactivation of x chromosome
Upf3A	random inactivation of x chromosome
Tet2	chromosomal 5methylcytosine dna demethylation oxidation pathway
Atad2B	transcription initiationcoupled chromatin remodeling
Kdm5A	facultative heterochromatin formation
Arid4A	genomic imprinting
Usp7	gene expression via chromosomal cpg island methylation
Atrx	nucleosome assembly
Lin54	nucleosome organization
Kmt2E	epigenetic gene expression
Baz2A	dna methylationdependent constitutive heterochromatin formation
Chd1	nucleosome organization
Pcgf3	random inactivation of x chromosome
Jarid2	facultative heterochromatin formation
Pole3	nucleosome assembly

Cebpg	nucleosome disassembly
Tlk1	chromatin organization
Brd2	nucleosome assembly
App	gene expression epigenetic
Tdg	epigenetic gene expression
Kcnq1Ot1	genomic imprinting
Glyr1	transcription initiationcoupled chromatin remodeling
Kdm2A	transcription initiationcoupled chromatin remodeling
Nrde2	regulatory ncrnamediated heterochromatin formation
Cenpv	pericentric heterochromatin formation
Npm1	nucleosome assembly
Smarcc2	nucleosome disassembly
Kat6B	nucleosome assembly
Gsk3B	autosome genomic imprinting
Rbm15	dosage compensation by inactivation of x chromosome
Smarcc1	nucleosome disassembly
Rlf	heterochromatin formation
Ythdc1	dosage compensation by inactivation of x chromosome
Hp1Bp3	nucleosome assembly
Smarca1	heterochromatin formation
Mecom	heterochromatin organization
Chaf1A	nucleosome assembly
Uhrf1	heterochromatin formation
Tdrd12	transposable element silencing by pirnamediated dna methylation
Airn	autosome genomic imprinting
Spi1	transcription initiationcoupled chromatin remodeling
Cdkn1C	genomic imprinting
Dnmt3B	autosome genomic imprinting
Apobec1	gene expression via chromosomal cpg island demethylation
Bcl6	heterochromatin formation
Prdm16	heterochromatin organization
Sox9	nucleosome assembly
Meg3	epigenetic programing of female pronucleus
Mcm2	nucleosome assembly
Cdk2	heterochromatin formation
Sphk2	transcription initiationcoupled chromatin remodeling
Baz1A	nucleosome assembly
Uty	heterochromatin formation
Klf2	epigenetic gene expression

Cbx5	heterochromatin formation
Scmh1	heterochromatin formation
Padi4	nucleosome assembly
Atad2	transcription initiationcoupled chromatin remodeling
Kat6A	nucleosome assembly
Mov10	transposable element silencing
Tet1	gene expression via chromosomal cpg island demethylation
Naa60	nucleosome assembly
Nasp	nucleosome assembly
Ubr2	heterochromatin formation
Eed	facultative heterochromatin formation
Kat7	transcription initiationcoupled chromatin remodeling
Mcrip1	gene expression epigenetic
Znhit1	transcription initiationcoupled chromatin remodeling
Hira	nucleosome assembly
Mis18A	heterochromatin formation
Kdm1A	transcription initiationcoupled chromatin remodeling
Smarca5	nucleosome assembly
Resf1	transposable element silencing by heterochromatin formation
Grwd1	nucleosome assembly
Hmgb1	heterochromatin formation
Wdhd1	pericentric heterochromatin organization
Tnrc18	transposable element silencing by heterochromatin formation
Ctr9	gene expression epigenetic
Msl2	epigenetic gene expression
Sgf29	transcription initiationcoupled chromatin remodeling
Sin3A	heterochromatin formation
Kmt2D	heterochromatin formation
Rb1	heterochromatin formation
Ezh1	constitutive heterochromatin formation
Set	nucleosome assembly
Hdac2	heterochromatin formation
Lrif1	dosage compensation by inactivation of x chromosome
Lcor	transcription initiationcoupled chromatin remodeling
Phf1	gene expression epigenetic
Hdac4	epigenetic gene expression
Trim37	gene expression epigenetic
Apex1	gene expression via chromosomal cpg island demethylation
Setd2	nucleosome organization
Dicer1	pericentric heterochromatin formation

Ehmt1	dna methylationdependent constitutive heterochromatin formation
Dpy30	transcription initiationcoupled chromatin remodeling
Cggbp1	epigenetic gene expression
Asf1B	nucleosome assembly
Cbx1	heterochromatin formation
Znfx1	regulatory ncrnamediated heterochromatin formation
Pphln1	constitutive heterochromatin formation
Axin1	epigenetic programming in the zygotic pronuclei
Eif1	random inactivation of x chromosome
Nap1L1	nucleosome assembly
Daxx	nucleosome assembly
Rsf1	nucleosome assembly
Suv39H2	epigenetic programming in the zygotic pronuclei
Lmnb2	heterochromatin formation
Spin1	transposable element silencing by pirnamediated dna methylation
Zfp335	epigenetic gene expression
Mki67	chromatin organization
Dot1L	subtelomeric heterochromatin formation
Alkbh4	gene expression epigenetic
Setdb1	heterochromatin organization
Men1	transcription initiationcoupled chromatin remodeling
Usp21	transcription initiationcoupled chromatin remodeling
Rbm14	transcription initiationcoupled chromatin remodeling
Bend3	dna methylationdependent constitutive heterochromatin formation
Letmd1	transcription initiationcoupled chromatin remodeling
Mcm3Ap	nucleosome organization
Zfp273	transposable element silencing
Prmt5	gene expression via chromosomal cpg island methylation
Smchd1	autosome genomic imprinting
Tut7	transposable element silencing by mrna destabilization
Pcid2	gene expression epigenetic
Setmar	gene expression epigenetic
Mettl4	gene expression epigenetic
Hdac9	gene expression epigenetic
Smarcd2	nucleosome disassembly
Ogg1	gene expression via chromosomal cpg island demethylation
Samd1	transcription initiationcoupled chromatin remodeling
Alkbh1	gene expression epigenetic

Ash2L	transcription initiationcoupled chromatin remodeling
Mbd2	dna methylationdependent constitutive heterochromatin formation
Epc1	chromatin organization
Gsk3A	autosome genomic imprinting
Chd2	nucleosome organization
Hdac6	gene expression epigenetic
Wdr5	transcription initiationcoupled chromatin remodeling
Crebzf	gene expression epigenetic
Nap1L3	nucleosome assembly
Trim28	dna methylationdependent constitutive heterochromatin formation
Mta1	gene expression epigenetic
Phf8	rdna heterochromatin formation
Kat5	chromatin organization
Bap1	heterochromatin formation
Dcaf13	epigenetic programming in the zygotic pronuclei
Hdac5	epigenetic gene expression
Ubr5	heterochromatin boundary formation
Setd1A	transcription initiationcoupled chromatin remodeling
Asf1A	nucleosome assembly
Ezh2	constitutive heterochromatin formation
Mbd1	dna methylationdependent constitutive heterochromatin formation
H3F3A	pericentric heterochromatin formation
Lbr	random inactivation of x chromosome
Smarcb1	transcription initiationcoupled chromatin remodeling
Tasor2	gene expression epigenetic
Dnmt1	dna methylationdependent constitutive heterochromatin formation
Hdac8	heterochromatin formation
Trip12	heterochromatin boundary formation
Uhrf2	gene expression via chromosomal cpg island methylation
Arid2	nucleosome disassembly
Ctcf	dna methylationdependent constitutive heterochromatin formation
Lsm 11.00	chromatin organization
Lmna	heterochromatin formation
Ddb1	epigenetic programming in the zygotic pronuclei
Trmt112	transcription initiationcoupled chromatin remodeling
Trim27	gene expression epigenetic

Atf2	transcription initiationcoupled chromatin remodeling
Morc2A	constitutive heterochromatin formation
Brd7	transcription initiationcoupled chromatin remodeling
Suz12	facultative heterochromatin formation
Smarcd1	transcription initiationcoupled chromatin remodeling
Tpr	heterochromatin formation
Cbx8	heterochromatin formation
Brca1	gene expression via chromosomal cpg island methylation
Zdbf2	epigenetic programming of gene expression

Sensory perception and hair cell functions

**Gene**	**Pathway**
Slc52A3	sensory perception of sound
Pou4F2	sensory perception of sound
Sptbn4	sensory perception of sound
Kcnq3	sensory perception of sound
Fhod1	stress fiber assembly
Rab40B	actin filament bundle assembly
Lpar1	stress fiber assembly
Rapgef3	stress fiber assembly
Ppm1F	stress fiber assembly
Arhgap28	stress fiber assembly
Otos	sensory perception of sound
Apoa1	stress fiber assembly
Tprn	auditory receptor cell stereocilium organization
Slit2	actin filament polymerization
Cdc42Ep3	actin filament polymerization
Tmeff2	stress fiber assembly
Ccn2	stress fiber assembly
Dock4	stereocilium
Nrp1	stress fiber assembly
Scin	actin filament capping
Kank2	actin filament polymerization
Kiss1R	stress fiber assembly
Col2A1	sensory perception of sound
Synpo	actin filament bundle assembly
Spns2	sensory perception of sound
Dcdc2A	kinocilium
Ssh3	actin filament polymerization

Map3K1	actin filament bundle assembly
Rgs4	actin filament organization
Shank1	actin filament bundle assembly
Wfs1	sensory perception of sound
Bbs2	stereocilium
Ttc8	inner ear receptor cell stereocilium organization
Baiap2L1	actin filament polymerization
Cyfip1	arp23 complexmediated actin nucleation
Cd47	stress fiber assembly
Arfip1	arp23 complexmediated actin nucleation
Tbx18	sensory perception of sound
Espnl	stereocilium
Hexb	sensory perception of sound
Cemip	sensory perception of sound
Myadm	actin filament polymerization
Ptprq	stereocilium
Large1	sensory perception of sound
Fam107B	sensory perception of sound
Arhgef10L	stress fiber assembly
Arap1	stress fiber assembly
Gjb2	sensory perception of sound
S1Pr1	stress fiber assembly
Gjb6	sensory perception of sound
Usp53	sensory perception of sound
Prkn	actin filament bundle assembly
Pcdh15	auditory receptor cell stereocilium organization
Lrp2	sensory perception of sound
Arpin	actin nucleation
Clasp2	stress fiber assembly
Asap3	stress fiber assembly
Ppip5K2	sensory perception of sound
Tesk1	stress fiber assembly
Bag4	actin filament polymerization
Rpl38	sensory perception of sound
C9Orf72	actin filament organization
Pak1	stress fiber assembly
Braf	stress fiber assembly
Washc4	arp23 complexmediated actin nucleation
Cotl1	actin filament polymerization
Flii	barbedend actin filament capping

Inppl1	actin filament organization
Dnm1	sensory perception of sound
Fbxo11	sensory perception of sound
Grxcr1	auditory receptor cell stereocilium organization
Lrig2	sensory perception of sound
Mpp1	stereocilium
Scarb2	sensory perception of sound
Cdh23	auditory receptor cell stereocilium organization
Capza2	actin filament capping
Abitram	actin filament polymerization
Cfl2	actin filament depolymerization
Nckap1	actin filament polymerization
Pik3Ca	actin filament depolymerization
Arpc5L	actin filament polymerization
Slc12A2	detection of mechanical stimulus involved in sensory perception of sound
Sod1	auditory receptor cell stereocilium organization
Kank3	actin filament polymerization
Cdkn2D	sensory perception of sound
Casp3	sensory perception of sound
Add3	barbedend actin filament capping
Mtss1	actin filament bundle assembly
Hip1R	actin filament polymerization
Pjvk	stereocilium maintenance
Grb2	actin filament polymerization
Fgfr1	sensory perception of sound
Rhoa	actin filament polymerization
Tsc1	stress fiber assembly
Washc2	arp23 complexmediated actin nucleation
Pfn2	actin filament polymerization
Cdc42	stress fiber assembly
Bbs4	actin filament polymerization
Rock2	stress fiber assembly
Myo6	sensory perception of sound
Pik3R2	actin filament polymerization
Specc1L	actin filament depolymerization
Arf1	arp23 complexmediated actin nucleation
Eps8	stereocilium
Capza1	actin filament capping
Nck2	actin filament polymerization

Rnh1	arp23 complexmediated actin nucleation
Clasp1	stress fiber assembly
Diaph3	stereocilia tiplink density
Wnt4	stress fiber assembly
Tub	sensory perception of sound
Hes5	inner ear receptor cell stereocilium organization
P2Rx2	sensory perception of sound
Coro1A	stereocilium tip
Col1A1	sensory perception of sound
Aqp4	sensory perception of sound
Ptk2B	actin filament polymerization
Fhod3	actin filament polymerization
Flna	actin filament bundle assembly
Vill	actin filament capping
Strc	auditory receptor cell stereocilium organization
Thrb	sensory perception of sound
Calb2	stereocilium
Kcnq4	sensory perception of sound
Elmod3	stereocilium maintenance
Col11A2	sensory perception of sound
Mrtfa	actin filament polymerization
Pvalb	stereocilium
Sh3Pxd2B	stress fiber assembly
Mkks	kinociliary basal body
Barhl1	sensory perception of sound
Kcnma1	sensory perception of sound
Chrna10	detection of mechanical stimulus involved in sensory perception of sound
Ush1C	inner ear receptor cell stereocilium organization
Scrib	auditory receptor cell stereocilium organization
Pgap1	sensory perception of sound
Ssh2	actin filament polymerization
Mlst8	actin filament polymerization
Nf2	stress fiber assembly
Baiap2L2	actin filament polymerization
Ccdc50	sensory perception of sound
Ppp1R9A	stress fiber assembly
Myh14	sensory perception of sound
Mtor	actin filament polymerization
Gpx1	sensory perception of sound

Lmod1	pointedend actin filament capping
Hexa	sensory perception of sound
Cdc42Ep4	actin filament polymerization
Shroom2	actin filament depolymerization
Pou4F3	sensory perception of sound
Myh9	actin filament capping
Stx4A	stereocilium
Cib2	stereocilium
Whamm	actin nucleation
Bin1	actin filament polymerization
Fer	actin filament polymerization
Homer2	stereocilium
Tmie	sensory perception of sound
Washc5	actin nucleation
Myo7A	auditory receptor cell stereocilium organization
Slc4A7	stereocilium
Abl1	stress fiber assembly
Cttn	actin filament polymerization
Triobp	auditory receptor cell stereocilium organization
Naa80	actin polymerization or depolymerization
Stmn1	stress fiber assembly
Ift27	inner ear receptor cell stereocilium organization
Espn	stereocilium
Coro2B	stress fiber assembly
Add1	barbedend actin filament capping
Cacna1D	sensory perception of sound
Atp2B2	auditory receptor cell stereocilium organization
Twf2	stereocilium
Cyfip2	actin filament polymerization
Tmem63B	sensory perception of sound
Ppfia1	stress fiber assembly
Ppm1E	stress fiber assembly
Washc1	actin filament polymerization
Tmod3	pointedend actin filament capping
Ddit3	sensory perception of sound
Tmod2	pointedend actin filament capping
Wasf2	stress fiber assembly
Dbnl	barbedend actin filament capping
Kif3A	inner ear receptor cell stereocilium organization
Trip11	inner ear receptor cell stereocilium organization

Sod2	sensory perception of sound
Ccdc88A	stress fiber assembly
Tmtc4	sensory perception of sound
Ankrd24	auditory receptor cell stereocilium organization
Nipbl	sensory perception of sound
Six1	sensory perception of sound
Eya4	sensory perception of sound
Minar2	inner ear receptor cell stereocilium organization
Fscn2	stereocilium
Kank1	actin filament polymerization
Lima1	actin filament depolymerization
Dstn	actin filament depolymerization
Zswim6	stereocilium
Arpc3	actin filament polymerization
Pafah1B1	stereocilium
Grxcr2	auditory receptor cell stereocilium organization
Arpc2	actin filament polymerization
Loxhd1	stereocilium
Arf6	actin filament polymerization
Pls1	auditory receptor cell stereocilium organization
Morn4	stereocilium
Itgb1Bp1	stress fiber assembly
Tomt	sensory perception of sound
Spry2	sensory perception of sound
Ift20	inner ear receptor cell stereocilium organization
Ror1	sensory perception of sound
Spta1	actin filament capping
Ptger4	stress fiber assembly
Prkcq	stress fiber assembly
Tectb	sensory perception of sound
Pdlim4	stress fiber assembly
Hpn	detection of mechanical stimulus involved in sensory perception of sound
Hes1	inner ear receptor cell stereocilium organization
Tmod4	pointedend actin filament capping
Daam2	actin filament polymerization
Dbn1	actin filament bundle assembly
Hcls1	actin filament polymerization
Arhgap6	stress fiber assembly
Cthrc1	inner ear receptor cell stereocilium organization

Ceacam16	stereocilium tip
Tlr2	actin filament polymerization
Slitrk6	sensory perception of sound
Arhgef5	stress fiber assembly
Npr 2.00	sensory perception of sound
Met	stress fiber assembly
Otog	inner ear receptor cell stereocilium organization
Ccl11	actin filament polymerization
Otogl	inner ear receptor cell stereocilium organization
Epyc	sensory perception of sound
Cx3Cl1	actin filament bundle assembly
Mbp	sensory perception of sound
Pecam1	actin filament polymerization
Avil	actin filament capping
Nckap1L	actin filament polymerization
Add2	barbedend actin filament capping
Lhfpl3	sensory perception of sound
Slc1A3	sensory perception of sound
Gmfg	arp23 complexmediated actin nucleation
Sema5A	actin filament depolymerization
Fzd4	sensory perception of sound
Col11A1	detection of mechanical stimulus involved in sensory perception of sound
Tbx1	sensory perception of sound
Plek	actin filament bundle assembly
Cdh1	sensory perception of sound
Evl	barbedend actin filament capping
Cdc42Ep5	actin filament polymerization
Map1A	sensory perception of sound
S100A10	stress fiber assembly
Slc17A8	sensory perception of sound
Plekhg2	actin filament polymerization
Rgcc	stress fiber assembly
Baiap2	actin filament polymerization
Cdc42Ep1	actin filament polymerization
Otoa	sensory perception of sound
Marveld2	sensory perception of sound
Srrm4	sensory perception of sound
Arhgef10	stress fiber assembly
Sox2	detection of mechanical stimulus involved in sensory perception of sound

Capn1	actin filament polymerization
Snx9	actin filament polymerization
Fmn1	actin filament polymerization
Eps8L1	stereocilium tip
Rhpn2	stress fiber assembly
Tmod1	pointedend actin filament capping
Piezo1	stereocilium
Kptn	stereocilium
Ift88	inner ear receptor cell stereocilium organization
Sobp	sensory perception of sound
Tmsb15B2	actin filament polymerization
Cdkn1B	sensory perception of sound
Vangl2	inner ear receptor cell stereocilium organization
Prex1	actin filament polymerization
Rsph9	kinocilium
Enpp1	sensory perception of sound
Plekhh2	actin filament depolymerization
Gsn	actin filament capping
Epha1	stress fiber assembly
Whrn	auditory receptor cell stereocilium organization
F11R	stress fiber assembly
Kit	detection of mechanical stimulus involved in sensory perception of sound
Pik3R1	stress fiber assembly
Fermt2	stress fiber assembly
Pls3	stereocilium
Tgfb3	stress fiber assembly
Rhoc	stereocilium
Dmtn	actin filament capping
Vezt	stereocilia ankle link complex
Ctnna2	arp23 complexmediated actin nucleation
Cabp2	sensory perception of sound
Sorbs3	stress fiber assembly
Prkcd	actin filament polymerization
Svil	barbedend actin filament capping
Rac1	kinocilium
Wasf1	arp23 complexmediated actin nucleation
Fchsd1	actin filament polymerization
Xirp2	actin filament organization
Actr3	actin filament polymerization

Mtpn	barbedend actin filament capping
Twf1	barbedend actin filament capping
Prkce	actin filament polymerization
Otof	sensory perception of sound
Id1	actin filament bundle assembly
Capg	actin filament capping
Myo3A	auditory receptor cell stereocilium organization
Pfn1	actin filament bundle assembly
Washc3	arp23 complexmediated actin nucleation
Actg1	stress fiber assembly
Cecr2	inner ear receptor cell stereocilium organization
Tjp1	stress fiber assembly
Rasa1	actin filament polymerization
Pkhd1L1	stereocilium coat
Limch1	stress fiber assembly
Arfip2	arp23 complexmediated actin nucleation
Chd7	sensory perception of sound
Lhfpl4	sensory perception of sound
Kncn	kinocilium
Hey1	inner ear receptor cell stereocilium organization
Myo1C	stereocilium
Gba2	actin filament polymerization
Capzb	actin filament capping
Tmsb4X	actin filament polymerization
Pak2	stress fiber assembly
Sptan1	actin filament capping
Sptbn1	actin filament capping
Kank4	actin filament polymerization
Arpc5	actin filament polymerization
Nav2	sensory perception of sound
Limk1	actin filament bundle assembly
Tmc1	stereocilium
Sdc4	inner ear receptor cell stereocilium organization
Cfl1	actin filament bundle assembly
Tpm1	actin filament capping
Arfgef1	actin filament polymerization
Brk1	arp23 complexmediated actin nucleation
Gsdme	sensory perception of sound
Axin1	sensory perception of sound
Pak3	actin filament polymerization

Gmfb	arp23 complexmediated actin nucleation
Carmil3	arp23 complexmediated actin nucleation
Calb1	stereocilium
Vasp	actin filament polymerization
Pou3F4	sensory perception of sound
Coch	sensory perception of sound
Tsku	inner ear receptor cell stereocilium organization
Pdxp	actin filament depolymerization
Phldb2	stress fiber assembly
Swap70	actin filament depolymerization
Synpo2	actin filament bundle assembly
Dlc1	stress fiber assembly
Fat4	inner ear receptor cell stereocilium organization
Ocm	stereocilium
Carmil2	barbedend actin filament capping
Clrn2	auditory receptor cell stereocilium organization
Pomgnt1	sensory perception of sound
Pxn	stress fiber assembly
Inpp5K	stress fiber assembly
Sh3Bp1	actin filament depolymerization
Cdc42Ep2	actin filament polymerization
Pycard	actin filament polymerization
Adcy6	stereocilium
Ush1G	inner ear receptor cell stereocilium organization
Coro1B	arp23 complexmediated actin nucleation
Alms1	inner ear receptor cell stereocilium organization
Sptb	actin filament capping
Smad3	stress fiber assembly
Diaph1	sensory perception of sound
Carmil1	barbedend actin filament uncapping
Pdzd7	auditory receptor cell stereocilium organization
Mcoln3	stereocilium membrane
Fchsd2	stereocilium
Tacstd2	stress fiber assembly
Wasf3	arp23 complexmediated actin nucleation
Ripor2	auditory receptor cell stereocilium organization
Cgnl1	stress fiber assembly
Chrnb2	sensory perception of sound
Tiam1	kinocilium
Mks1	inner ear receptor cell stereocilium organization

Chrna9	detection of mechanical stimulus involved in sensory perception of sound
Rhpn1	stress fiber assembly
Scn8A	sensory perception of sound
Rictor	actin filament polymerization
Ssh1	actin filament polymerization
Psap	sensory perception of sound
Arhgef18	stress fiber assembly
Adgrv1	inner ear receptor cell stereocilium organization
Lats1	actin filament polymerization
Arhgap18	actin filament polymerization
Wdr1	actin filament depolymerization
Arhgap35	actin polymerization or depolymerization
Tmprss3	sensory perception of sound
Eps8L2	stereocilium
Ndufs4	sensory perception of sound
Mpv17	sensory perception of sound
Sec24B	auditory receptor cell stereocilium organization
Lhfpl5	auditory receptor cell stereocilium organization
Abi2	arp23 complexmediated actin nucleation
Rdx	stereocilium
Pick1	arp23 complexmediated actin nucleation
Atp8B1	stereocilium
Jag2	sensory perception of sound
Dlg1	actin filament polymerization
Trim27	actin nucleation
Clic5	auditory receptor cell stereocilium organization
Serpinb6A	sensory perception of sound
Ap1Ar	arp23 complexmediated actin nucleation
Hax1	actin filament organization
Rest	auditory receptor cell stereocilium organization
Clrn1	auditory receptor cell stereocilium organization
Ikzf2	sensory perception of sound
Nck1	actin filament polymerization
Cdc14A	kinociliary basal body
Crym	sensory perception of sound
Lrig1	sensory perception of sound
Kcnq1	detection of mechanical stimulus involved in sensory perception of sound
D130043K22Rik	sensory perception of sound

Figure S1 source data

S1A, B

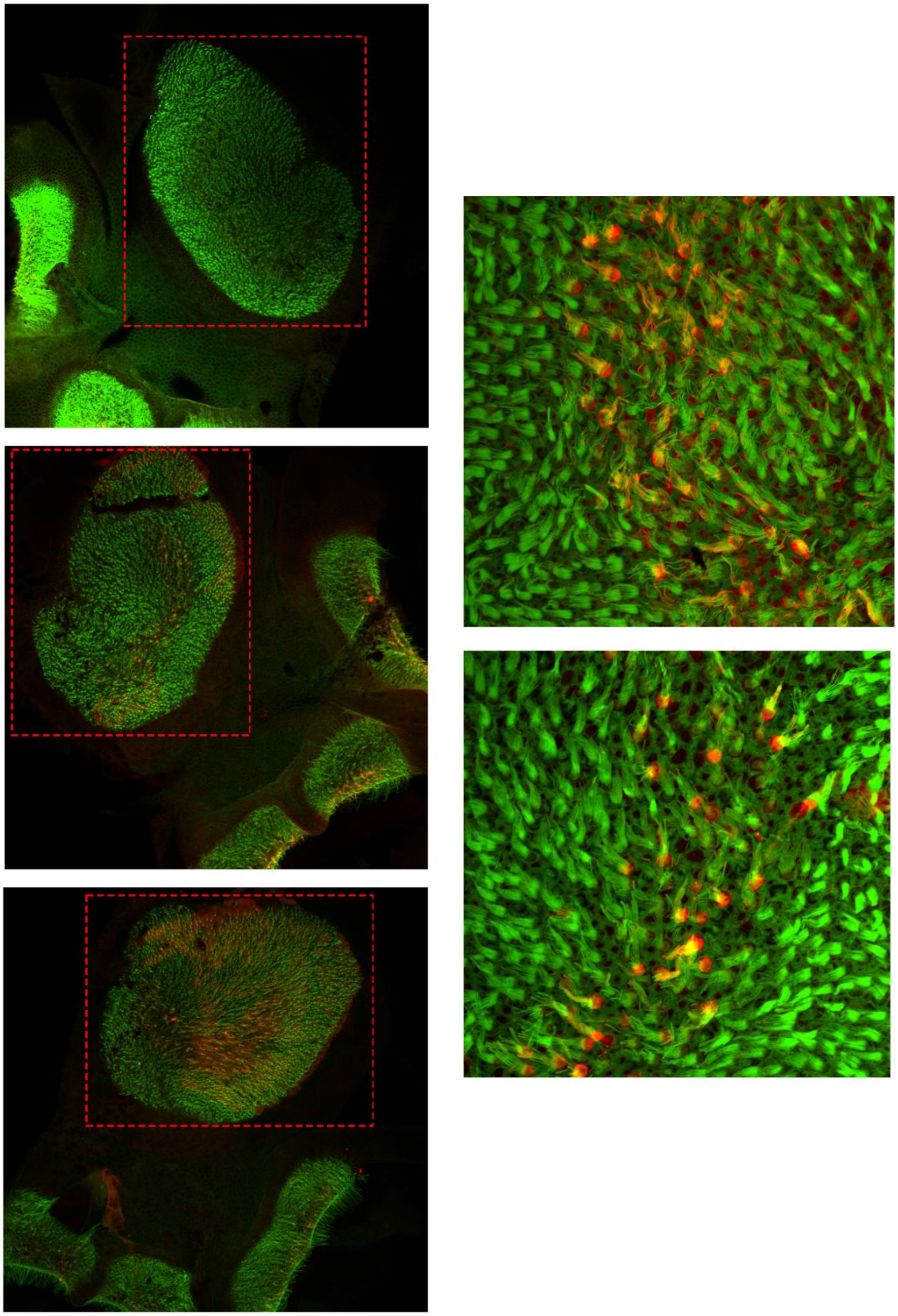

S1C

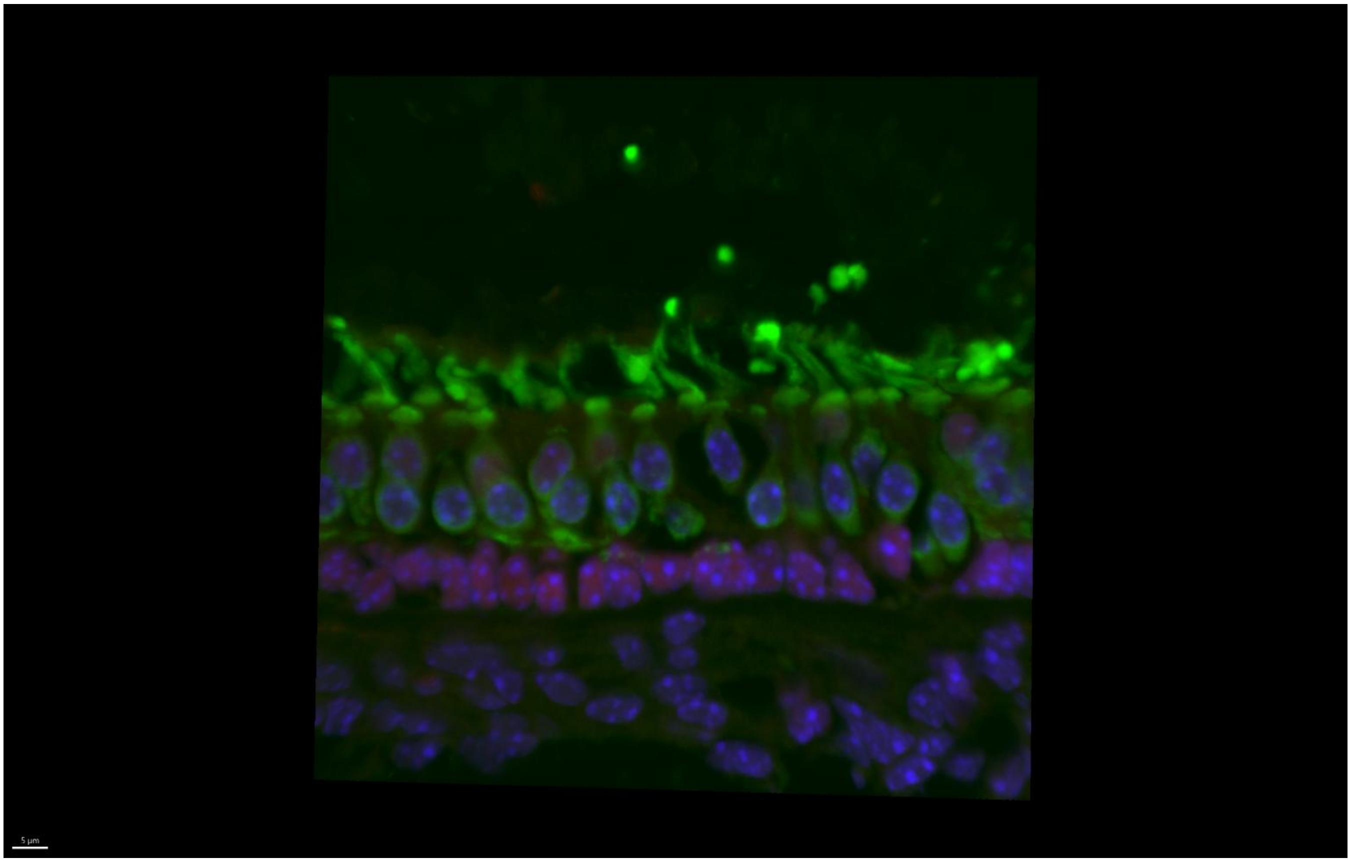

Figure S4 source data

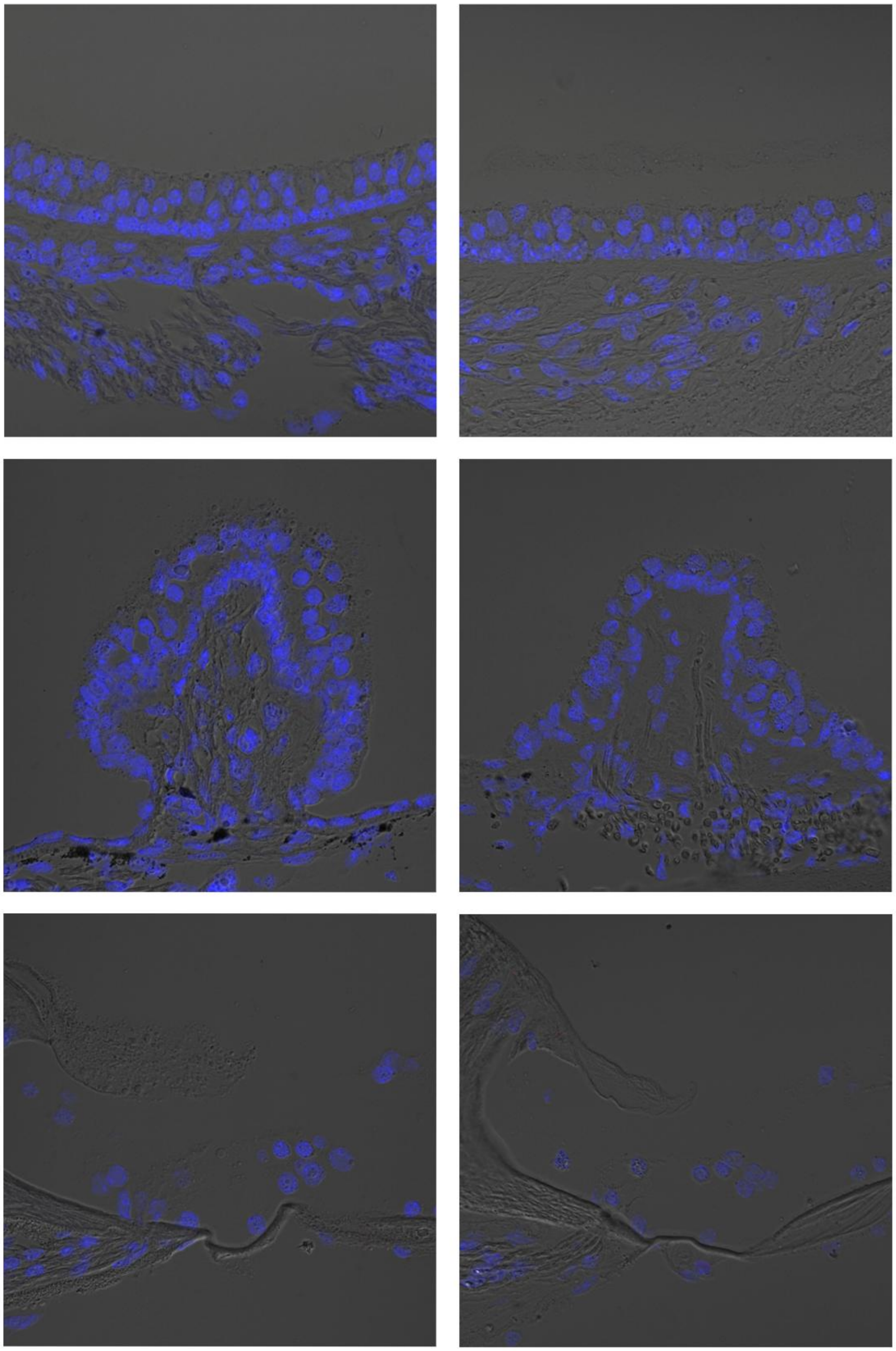

Figure S5 source data

S5A

Young	4	8	12	16	22	32	40	50
#1	60	40	30	20	25	30	40	45
#2	55	30	20	10	20	25	30	45
#3	50	30	30	20	20	25	30	50
#4	45	30	25	30	10	20	25	45
#5	50	40	30	20	20	30	30	45
#6	50	40	20	10	20	30	40	45
#7	55	40	20	15	20	25	30	40
#8	50	35	20	15	15	20	25	35
#9	55	35	20	20	25	30	40	55
#10	50	45	20	15	20	25	40	50
#11	55	35	20	15	15	25	40	50
Old								
#1	90	75	55	35	40	50	65	80
#2	85	80	60	50	45	50	60	80
#3	80	75	50	40	40	50	60	75
#4	90	80	60	45	45	60	75	90
#5	85	75	55	45	45	55	65	80
#6	90	80	55	45	40	55	65	85
#7	85	70	55	40	45	50	60	75
#8	80	70	50	30	30	40	60	85

S5B

Young	8K	12k	16k	22k	32k
#1	50	40	30	35	40
#2	40	30	30	30	35
#3	40	30	30	30	35
#4	40	30	20	20	30
#5	50	35	30	30	40
#6	50	30	20	30	45
#7	50	30	25	30	35
#8	45	30	25	25	30
#9	45	35	30	35	40

#10	55	30	25	30	40
#11	45	25	25	25	35
Old					
#1	75	50	45	50	60
#2	80	60	50	55	65
#3	80	55	50	55	65
#4	70	55	45	45	55
#5	80	55	45	45	55
#6	80	55	45	50	60
#7	80	60	50	50	60
#8	75	50	40	40	50

S5C

Young	mV
#1	85
#2	90
#3	89
#4	98
#5	90
#6	100
Old	
#1	90
#2	98
#3	95
#4	105
#5	100
#6	103

S5D

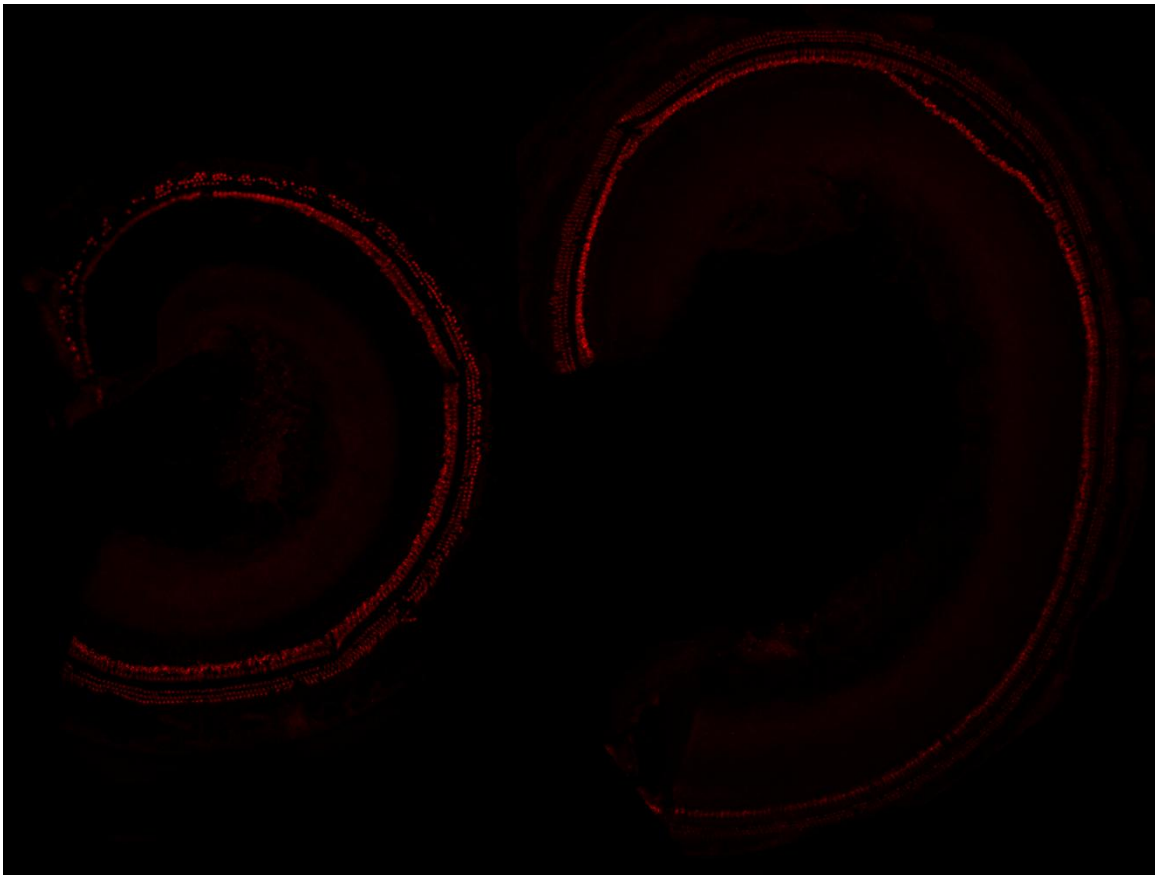

Young IHCs	5%	15%	25%	35%	45%	55%	65%	75%	85%	95%
#1	42		41		41		42		43	42
#2	43		43		43		44		45	48
#3	40		43		44		45		42	44
OHCs										
#1	139		139		146		145		147	143
#2	134		142		142		142		145	148
#3	135		144		146		147		150	150
Old IHCs	5%	15%	25%	35%	45%	55%	65%	75%	85%	95%
#1	23		90		120		119		108	41
#2	19		95		104		120		112	20
#3	14		106		118		101		100	24
OHCs										
#1	32		42		42		38		38	25
#2	36		41		40		40		40	14
#3	37		38		37		42		42	16

S5E

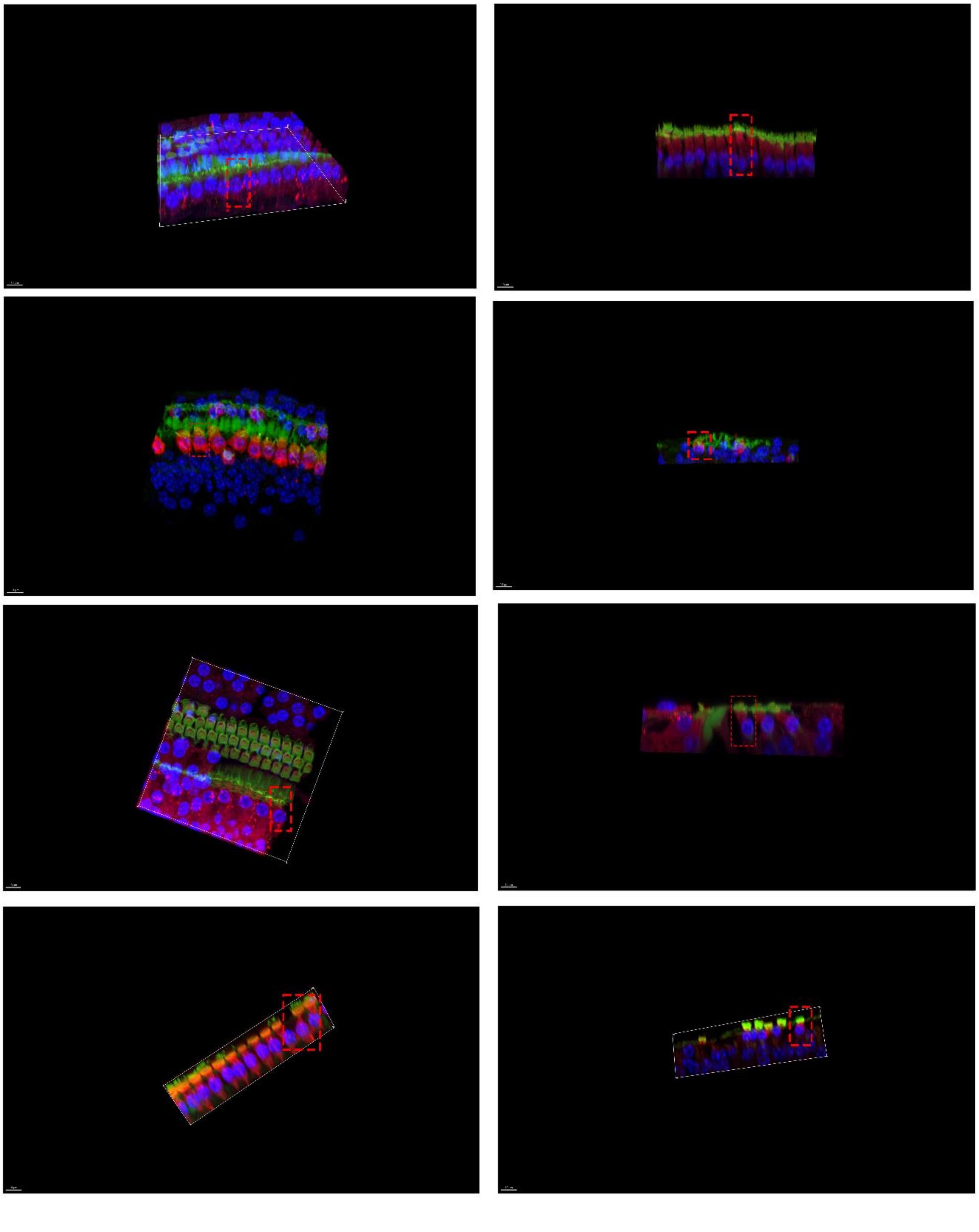

Figure S8 source data

S8A

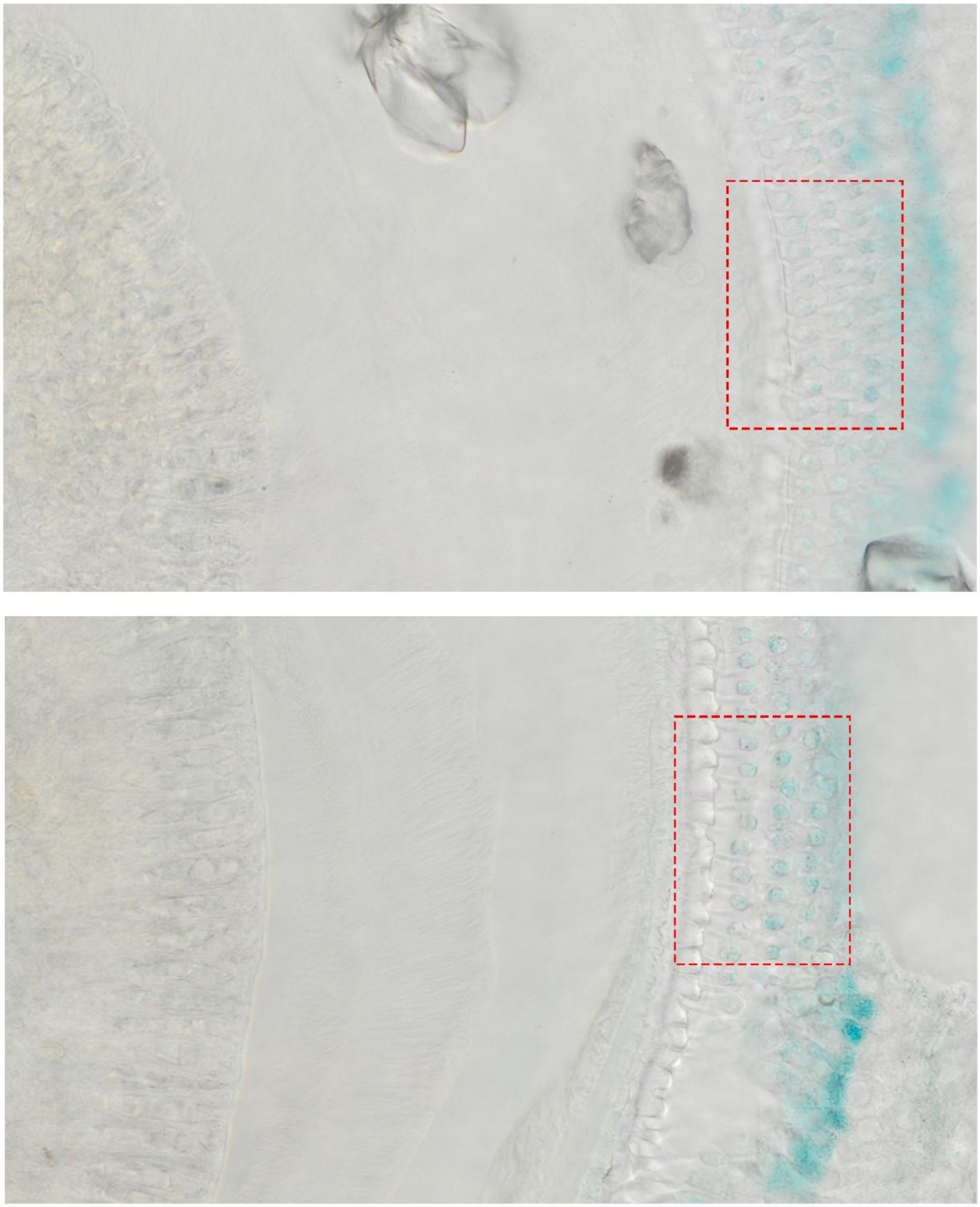

S8B

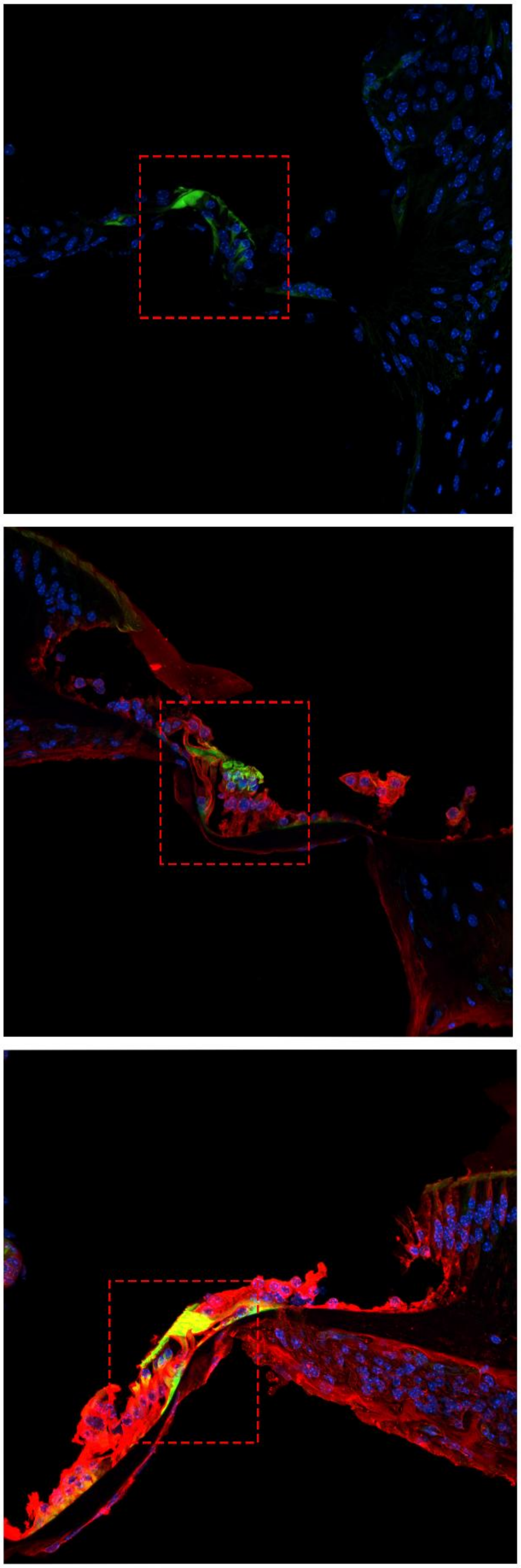

S8C

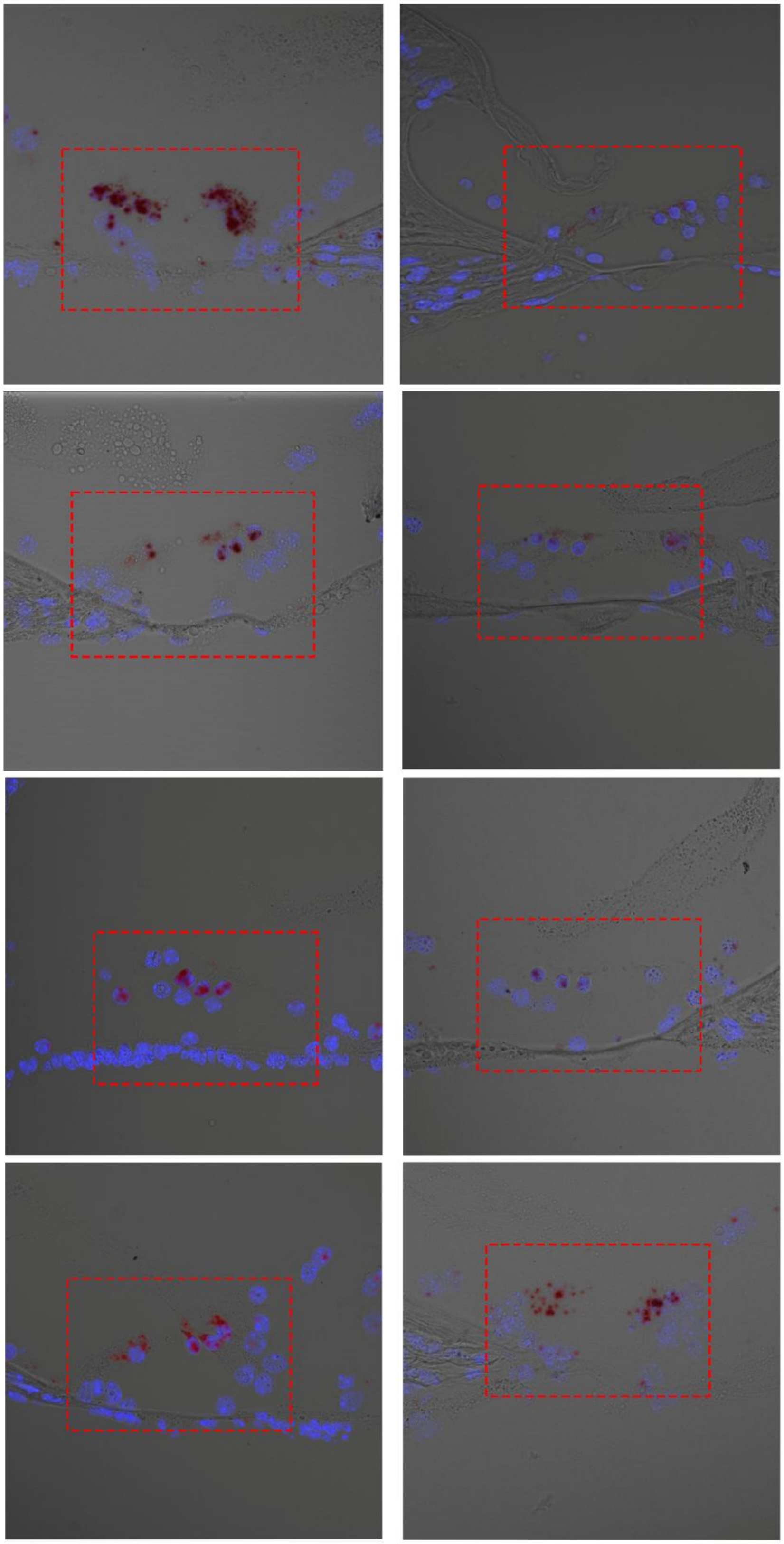

Supplement: Supplementary file 4 — Supporting File 4: advs76340‐sup‐0004‐Data.zip. [file ADVS-9999-e76340-s004.zip › Supporting information_data.pdf]
